# Redshifting TADF Emission With a Bora‐Triptycene Lewis Acid

**DOI:** 10.1002/anie.3353986

**Published:** 2026-09-26

**Authors:** Mathew J. Cross, Emily L. Hanover, Arnaud Osi, Eli Zysman‐Colman, Michael J. Ingleson

**Affiliations:** ^1^ EaStCHEM School of Chemistry University of Edinburgh Edinburgh UK; ^2^ Organic Semiconductor Centre EaStCHEM School of Chemistry University of St Andrews St. Andrews UK

**Keywords:** bora‐triptycene, fluorescence, Lewis acid, MR‐TADF, TADF

## Abstract

Binding of a Lewis acid to an emitter molecule is a versatile method to redshift the emission. This approach is underexplored for thermally activated delayed fluorescence (TADF) materials. In this study, we report that the addition of a Lewis acid can induce large (>100 nm) bathochromic shifts in the emission in sulfone‐based donor–acceptor TADF (DA‐TADF) emitters and for the first time in multi‐resonant TADF (MR‐TADF) emitters, whilst retaining TADF behaviour. This is achieved through the binding of a 9‐sulfonium‐10‐bora‐triptycene (BTripS) Lewis acid to the fluorophore carbonyl or sulfone moiety to form bench‐stable Lewis adducts. In contrast, when using B(C_6_F_5_)_3_ as the Lewis acid, the generated adducts rapidly decompose on exposure to water to form H_2_O‐B(C_6_F_5_)_3_. This work demonstrates that the formation of bench‐stable Lewis adducts has significant potential as a method to redshift the emission of two popular classes of TADF compounds. This provides a new approach to address the current challenge of accessing longer‐wavelength‐emitting MR‐TADF materials.

## Graphical abstract

Bathochromic emission shifting of donor–acceptor and multi‐resonant TADF emitters is achieved by adduct formation with a thia‐bora‐triptycene Lewis acid. TADF properties are retained on adduct formation whilst delivering > 100 nm redshift of the emission.

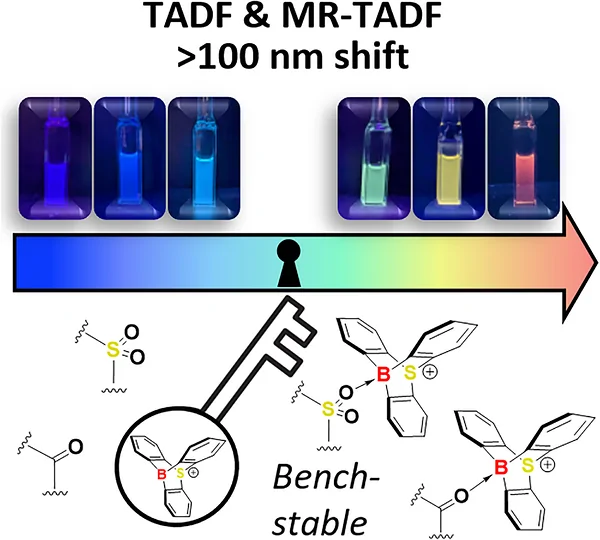

## 1 Introduction

Thermally activated delayed fluorescence (TADF) materials research has seen an explosion of activity over the past 15 years, primarily driven by their promise as emitters for organic light‐emitting diodes (OLEDs) [1, 2, 3]. Beyond OLEDs, this class of emitter has seen increasing use in other fields, such as sensing, bioimaging and photocatalysis [4, 5], exploiting the coupled singlet and triplet excited states and the relatively long radiative lifetimes. TADF is enabled by a small energy gap between the *S*_1_ and *T*_1_ states (Δ*E*_ST_), and an endothermic reverse intersystem crossing (RISC) that permits triplet excitons to be converted into singlets. The magnitude of Δ*E*_ST_ is correlated with that of the exchange integral, meaning spatial separation of the HOMO and LUMO is a required structural design element for TADF materials. The most common design is a strongly twisted donor–acceptor architecture (DA‐TADF) that limits conjugation between these two moieties (e.g., Figure 1a). A second design is based on p‐ and n‐doped polycyclic aromatic hydrocarbons, termed multi‐resonant TADF (MR‐TADF, e.g., **DiKTa**, Figure 1a). In this class, the small Δ*E*_ST_ is achieved by the complementary electron density distribution in the excited state compared to the ground state that is controlled by the distinct mesomeric effects of the dopants. Many of the acceptor units used in TADF emitters contain Lewis basic sites like the oxygen atoms of the sulfone and carbonyl moieties in the examples shown in Figure 1a [6, 7, 8]. They can therefore interact with Lewis acids to form adducts. How Lewis adduct formation impacts the TADF properties of emitters containing these acceptor units has not been assessed to date. Furthermore, to the best of our knowledge there have been no optoelectronic studies into Lewis adducts formed between an MR‐TADF material and a Lewis acid [9].

**FIGURE 1:**
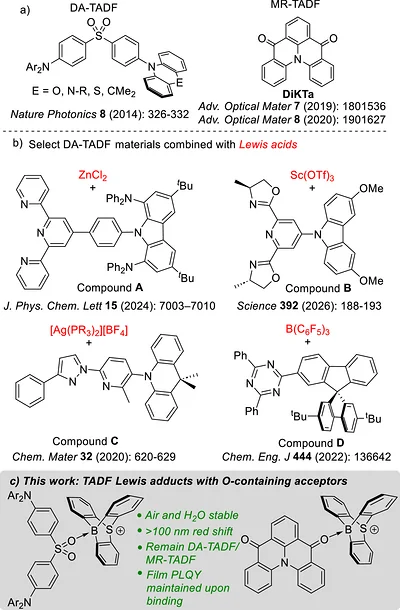
(a) Exemplar DA‐TADF and MR‐TADF materials. (b) Examples of Lewis acid‐coordinated DA‐materials exhibiting redshifted emission compared to their parent. (c) This work highlights the first Lewis adducts of DA‐TADF and MR‐TADF emitters with 9‐sulfonium‐10‐bora‐triptycene.

The small number of optoelectronic studies into the impact of Lewis acid binding on DA‐TADF materials, to our knowledge, all involve N‐heterocycle acceptor units (e.g., Figure 1b), with the majority of these involving chelating units to strengthen Lewis acid binding [10, 11, 12, 13]. From these studies, Lewis adduct formation generally leads to a deeper LUMO, a stabilised *S*_1_ energy and a smaller Δ*E*_ST_. Compound **D** is noteworthy in the context of this work as it contains a weakly Lewis basic monodentate binding site (thus is related to the compounds in this study) [13]. On mixing **D** with commercial B(C_6_F_5_)_3_ (termed BCF herein), a considerable reduction in the Δ*E*_ST_ was observed (from 0.59 eV in the neat film of the parent to 0.08 eV for the parent doped with 10 wt% BCF film, respectively). This turned on TADF, while also resulting in a significant redshift in the emission [13]. However, it is established from frustrated Lewis pair chemistry that the formation of dative bonds between BCF and hindered N‐heterocycles, such as 2,4,6‐triphenylpyridine, is endergonic (see Section S1.8.1) [14, 15, 16, 17]. Therefore, the origin of the observed bathochromic shift and change in Δ*E*_ST_ is unlikely to be due to Lewis adduct formation between the triazine unit in **D** and BCF as ascribed by the authors. An alternative, more likely possibility is that **D** interacted with the water adduct of BCF, H_2_O‐BCF, via a hydrogen‐bonding interaction (e.g., triazine(N)···H─O(H)···BCF). H_2_O‐BCF is a strong Brønsted acid comparable in p*K*_a_ to HCl [18], and the interaction of an emitter with a Brønsted acid has been previously reported to induce a bathochromic shift in the delayed emission of DA‐TADF materials [19, 20].

The reactivity between **D** and BCF/H_2_O‐BCF discussed above highlights that identifying the product arising from the combination of a Lewis acid with a TADF material must accompany optoelectronic studies for valid structure–property relationships to be extracted. This should include determining the product's stability, especially towards water (including the trace amounts present in organic solvents). Photophysical studies at “standard” concentrations (e.g., 1 × 10^−5^ M) require water‐stable Lewis adducts. However, bench‐stable Lewis adducts of TADF materials remains underexplored, particularly for MR‐TADF emitters, and it is unclear whether delayed emission will be conserved in MR‐TADF‐Lewis acid adducts. Should it be the case, then Lewis acid addition to MR‐TADF materials would become a viable strategy to promote long‐wavelength delayed emission.

Herein, we report a combined theoretical, chemical, and optoelectronic study into the binding of boron‐based Lewis acids to DA‐TADF and MR‐TADF materials containing diaryl‐sulfone and ‐ketone Lewis basic sites, respectively. Initial studies using purified BCF as the Lewis acid revealed: (i) the Lewis adducts were highly sensitive to water; (ii) the Lewis adduct formed with BCF resulted in a much larger redshift in the emission wavelength relative to that observed when the same TADF material was combined with the Brønsted acid H_2_O‐BCF. This led to the development of a second generation of Lewis adducts using a different boron‐based Lewis acid, a 9‐sulfonium‐10‐bora‐triptycene. Using this Lewis acid, adducts with both diaryl‐sulfone‐ and ‐ketone‐containing TADF materials were generated and found to be water‐stable. Furthermore, the emission of the Lewis adducts derived from both DA‐ and MR‐TADF materials was strongly redshifted by >100 nm and, importantly, retained the TADF of the parent. This work therefore demonstrates for the first time how bench‐stable Lewis adducts of DA‐TADF and MR‐TADF materials and a Lewis acid can be formed that retain their delayed emission while having a dramatic redshift in emission relative to their parent emitter.

## 2 Results and Discussion

For this study, four TADF emitters were chosen: the diphenylamine (DPA) and 9,9‐dimethyl‐9,10‐dihydroacridine (DMAC) diphenylsulfone (DPS) derivatives, **DPA‐DPS** and **DMAC‐DPS**, as representative DA‐TADF emitters (**1** and **2**, respectively), and **DiKTa** (**3**) and the mixed carbonyl/sulfone compound **QPO** (**4**) as representative MR‐TADF emitters [6, 8, 21, 22].

### 2.1 Lewis Adducts With B(C_6_F_5_)_3_

Initial studies to form Lewis adducts used BCF. First, it was crucial to confirm whether an emitter‐BCF Lewis adduct forms and if so, to understand the stability of this adduct. Upon combination of **2** with thoroughly dried BCF under strictly anhydrous conditions, the colourless solution turned to pale yellow. The in situ ^1^H NMR spectra (Figure S60) showed broadened signals, but there were no significant chemical shift changes, while the ^11^B and ^19^F NMR spectra showed resonances close to that reported for free BCF (Figures S61 and S62). This indicates weak and reversible adduct formation, with an equilibrium favouring free BCF and **2** over the Lewis adduct (Figure 2). Using BCF in the presence of water also led to minimal changes in the NMR spectra of **2** (Figure S64) and, importantly, no changes in the emission profiles (Figure S77) compared with those for pure **2**. This indicates that BCF (wet or dry) is not an appropriate Lewis acid to use with sulfone‐containing emitters in this context.

**FIGURE 2:**
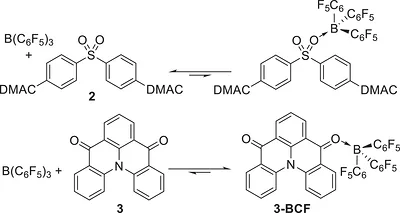
Reversible Lewis adduct formation between TADF emitters **2** and **3** with BCF.

The reaction of equimolar dried BCF with **3** under anhydrous conditions resulted in clear formation of a Lewis adduct, with ^11^B and ^19^F NMR spectra consistent with **3‐BCF** (Figure 2). However, at 298 K the ^13^C{^1^H} NMR spectrum of **3‐BCF** showed only one carbonyl resonance and the ^1^H NMR spectrum exhibited only six aromatic resonances (Figure S36). Upon cooling to 235 K, the number of resonances increased to the expected two ^13^C carbonyl resonances and eleven ^1^H resonances for **3‐BCF,** with the resonances of **3‐BCF**, **3,** and BCF all observed at this temperature (Figures S45 and S48). This indicates that BCF exchange between both carbonyls of **3** is rapid at ambient temperature and that there is an equilibrium in solution under these conditions favouring the Lewis adduct **3‐BCF** at room temperature. The formation of **3‐BCF** resulted in a 102 nm bathochromic shift of the emission maximum, *λ*_PL_, from 452 nm for **3** to 554 nm for **3‐BCF** at 1 × 10^−2^ M in toluene solution (Figure S76). Notably, **3‐BCF** was unstable to hydrolysis. The reaction of **3‐BCF** with water, forming H_2_O‐BCF and **3** (which themselves interact—vide infra), was observed by multinuclear NMR spectroscopy upon opening dry‐CDCl_3_ solutions of **3‐BCF** to air (Figures S69 and S70). Furthermore, using ratios of H_2_O:**3‐BCF** that mimic those used to perform photophysical measurements at more dilute concentrations (e.g., 1 × 10^−5^ M of fluorophore in a solvent containing 100 ppm of H_2_O, which equates to 5 × 10^−3^ M), a rapid reaction was observed between **3‐BCF** and water (Figures S69 and S70). It should be noted that solutions containing **3** and H_2_O‐BCF also displayed a bathochromic shift of *λ*_PL_ relative to **3** (Figures S72 and S76), though this was much less than the redshift observed for **3‐BCF**. This redshift of the emission of **3** in the presence of H_2_O‐BCF is related to that reported for **3** in the presence of excess trifluoroacetic acid [19], and indicates that H_2_O‐BCF can engage in sufficiently strong hydrogen bonding to weakly Lewis basic sites like the carbonyl groups of **3** to perturb its photophysical properties. This initial study with BCF highlights the complexity in studying Lewis adducts that are not bench stable. Motivated by the considerable bathochromic shift observed in the emission of the Lewis adduct **3‐BCF**, bench‐stable Lewis adducts between the sulfone/carbonyl containing TADF materials **1–4** and a different Lewis acid were thus sought.

### 2.2 Lewis Adducts With a Bora‐Triptycene: DA‐TADF Compounds

Berionni and co‐workers previously reported that the cationic Lewis acid 9‐sulfonium‐10‐bora‐triptycene (BtripS) was capable of coordinating to the oxo‐site of weakly Lewis‐basic sulfones, such as [N(SO_2_CF_3_)_2_]^–^ ([NTf_2_]^–^), to form bench‐stable adducts [23, 24, 25]. This makes BtripS an attractive Lewis acid to form Lewis adducts with TADF materials containing weakly Lewis basic sites, such as **1–4**. Following the reported methodology [25], Lewis adducts between sulfone‐containing fluorophores **1** and **2** and BtripS were synthesised and isolated in yields of 34% and 29% as their [B(C_6_F_5_)_4_]^–^ salts (**1a** and **2a**) (Figure 3). Importantly, **1a** and **2a** showed no evidence of decomposition upon prolonged exposure to excess water by ^1^H NMR spectroscopy (Figures S66 and S67). The addition of two equiv. of BtripS to **2** led to a complex mixture (by in situ multinuclear NMR spectroscopy) and only **2a** could be isolated on work‐up.

**FIGURE 3:**
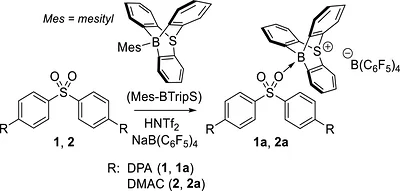
The synthesis of Lewis adducts **1a** and **2a** from **1** and **2**.

Lewis‐adduct formation with the sulfone units in **1** and **2** further polarises the ^δ+^S═O^δ−^ bond. This increases the overlap between the S═O σ^*^ orbital and the extended π system of the emitter, leading to an increase in the acceptor strength (Figures S98–S101) [26]. Density functional theory (DFT) calculations (PBE0/6‐31G(d,p), SMD: toluene, Figures 4 and S106) were performed. This included a range of conformers of **2** and **2a**, as the DMAC donor can adopt both a planar or a puckered conformation that then impacts the dihedral angle with the adjacent phenylene bridge, producing quasi‐equatorial and quasi‐axial conformers, respectively (Figure S113) [27]. The lowest‐energy conformer for **2** (by 0.5 kcal/mol) was where both DMAC were quasi‐equatorial, whereas for **2a** it contained one quasi‐equatorial and one quasi axial DMAC (lowest energy by 2.8 kcal/mol, which equates to this conformer being >99% in solution at 298 K). Note that the different relative conformer energies for **2** versus **2a** are attributed to the significant steric bulk of the BTripS unit (see Section S1.9). These calculations revealed a narrowing of the HOMO–LUMO gap (Δ*E*) of the lowest‐energy conformer of 0.3 eV for **1a** (vs. **1**) and 0.43 eV for **2a** (vs. **2**), while conserving the spatial distributions of the electron density in these orbitals. Time‐dependent DFT calculations at the same level of theory revealed that the *S*_1_ energy is stabilised for **1a** and **2a** relative to **1** and **2** by 0.24 and 0.46 eV, respectively, while the *S*_0_–*S*_1_ transitions maintain a non‐zero oscillator strength (*f*) (Figure S107). Note, the different degree of twisting of the donor NAr_2_ unit (Figure S112), from the phenylene unit of DPS **1** (*θ* ≈ 31°) to **1a** (*θ* ≈ 22°) and **2** (*θ* ≈ 94°) to **2a** (*θ* ≈ 84° for the quasi‐equatorial‐DMAC unit wherein the HOMO is located), impacts how delocalised the HOMO is and thus the extent of HOMO stabilisation upon Lewis acid binding, resulting in the varying magnitude of the change in Δ*E* and *S*_1_ values (Figure 4). Notably, these calculations showed minimal change to the Δ*E*_ST_ values on Lewis adduct formation (ΔΔ*E*_ST_ = 0.02 eV for **1/1a** and 0.04 eV for **2/2a**), implying that both the *S*_1_ and *T*_1_ long‐range charge transfer (LRCT) states are similarly affected by Lewis acid coordination.

**FIGURE 4:**
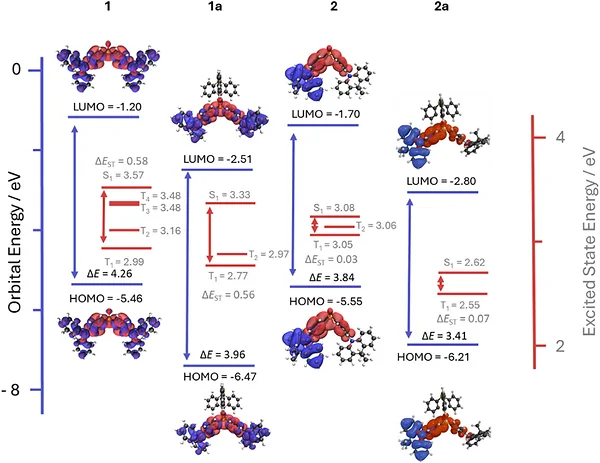
Computed ground‐state (HOMO and LUMO; blue) and excited‐state (red) energies of **1**, **1a**, **2** and **2a**, calculated at the PBE0/6‐31G(d,p) (SMD toluene) level of theory. The images are the natural transition orbitals of the *S*_1_ (top) and *T*_1_ (bottom) (isovalue = 0.02).

With compounds **1**, **1a**, **2,** and **2a** in hand, their HOMO and LUMO energy levels were inferred from cyclic voltammetry (CV) and differential pulse voltammetry (DPV) experiments performed in DCM (Figure S83 and Table 1). No reduction waves were observed for compounds **1** and **2** within the solvent window, whereas a first irreversible reduction event was observed for **1a** and **2a** at −1.66 and −1.29 V, respectively, versus SCE. These processes correspond to LUMO energies of −2.68 and −3.05 eV, respectively, in line with the DFT calculations. Oxidation waves at *E*_ox_ of 1.00, 1.33, 0.82 and 0.91 V versus SCE were detected, implying HOMO energies of −5.34, −5.69, −5.16 and −5.25 eV for **1**, **1a**, **2** and **2a**, respectively. The observed trend in the stabilisation of the HOMO is also in line with that calculated by DFT.

**TABLE 1:** Selected optoelectronic properties for compounds **1–3** and **1a–3a**.

	Solution state						1 wt% in PMMA				
	*λ*_PL_ (nm)^a^	FWHM (nm)^a^	∆*E*_ST_ (eV)^b^	*Φ*_PL_ (%)^c^	*E*_ox_ (V)^d^	*E*_red_ (V)^d^	*λ*_PL_ (nm)^e^	FWHM (nm)^e^	∆*E*_ST_ (eV)^f^	*Φ*_PL_ (%)^g^	*τ*_p_ (ns)^h^/*τ*_d_ (µs) ^i^
**1**	400	50	0.43	54	1.00	—	401	65	0.50	30	1.8/—
**1a**	523	121	0.31	46	1.33	−1.66	452	91	0.40	32	3.2/5546
**2**	466	80	—	33	0.82	—	458	89	0.06	39	12.4/38.0
**2a**	653	173	0.01	2	0.91	−1.29	538	137	—	33	12.0/0.998
**3**	451	28	0.19	77	—	−1.32	468	49	0.20	13	3.1/353.7
**3a**	562	91	0.18	62	—	−0.45	553	96	—	20	10.1/13904.9

^a^ In dilute toluene solution at 293 K (*λ*_exc_ = 340 (**1**, **2**, **2a** and **3**), 360 (**2a**), or 450 (**3a**) nm).

^b^ Determined from the onsets of the SS PL and delayed emission spectra (1–9 ms) in 2‐MeTHF at 77 K (*λ*_exc_ = 340 (**1**, **2**, **2a** and **3**), 360 (**1a**) and 450 (**3a**) nm).

^c^ In degassed dilute toluene solution at 293 K (*λ*_exc_ = 340 (**1**, **2**, **2a** and **3**), 360 (**1a**) and 450 (**3a**) nm).

^d^ In DCM with 0.1 M [*^n^* Bu_4_N][PF_6_], relative to SCE [28, 29].

^e^ *λ*_exc_ = 320 (**2**), 335 (**1**), 340 (**1a**, **3a**), 360 (**2a**) and 330 (**3**) nm.

^f^ Determined from the onsets of the SS PL and delayed emission spectra (1–9 ms) (*λ*_exc_ = 320 (**2**), 330 (**3**), 335 (**1**), 340 (**1a** and **3a**) and 360 (**2a**) nm).

^g^ Measured using an integrating sphere.

^h^ Measured by TCSPC (*λ*_exc_ = 375 nm).

^i^ Measured by MCS (*λ*_exc_ = 320 (**2**), 330 (**3**), 335 (**1**) and 340 (**1a** and **3a**) nm) and TCSPC (*λ*_exc_ = 375 (**2a**) nm).

The photophysical properties in dilute toluene solution of **1** and **2** and their Lewis adducts **1a** and **2a** were investigated next. The lowest energy absorption band, corresponding to the *S*_0_→*S*_1_ transition of **1** and **2**, is weakly bathochromically shifted in **1a** and **2a** (from 350 to 367 nm and from 370 to 425 nm, respectively, Figure S78). The *λ*_PL_ values (400 nm for **1** and 466 nm for **2**) redshift much more strongly upon adduct formation to *λ*_PL_ = 523 and 653 nm for **1a** and **2a**, respectively.

PL solvatochromism studies reveal positive solvatochromism for all four compounds that is consistent with LRCT excited states (Figure S80), with the magnitude of the solvatochromism decreasing upon adduct formation. Lippert–Mataga plot analysis for all four compounds (Figure S95) showed that the relationship between the Stokes shift and the orientation polarisability of the solvent is linear and positive [30, 31, 32]. The gradient of the lines of best fit for the two Lewis adducts is smaller than those of the respective parent emitters, implying that the adducts have a smaller change in transition dipole moment (for *S*_0_ vs. *S*_1_) relative to their parent emitters, consistent with calculations (Figure S110). Adduct formation resulted in a decrease in the photoluminescence quantum yield (*Φ*_PL_) from 54% to 46% (**1** and **1a**) and 33% to 2% (**2** and **2a**), a trend that is in line with the energy gap law. The ∆*E*_ST_, experimentally determined from the difference in energy between the onsets of the steady‐state (SS)‐PL and gated emission spectra at 77 K in 2‐MeTHF glass (Figure S84), decreases from 0.43 eV for **1** to 0.31 eV for **1a**. The onset of both the SS‐PL and gated emission was found to be 3.06 eV for compound **2**, suggesting a ∆*E*_ST_ value of 0 eV, though this has been reported in the literature to be 0.09 eV in toluene [6]. For **2a**, this value was found to be 0.01 eV (see section S1.9). Time‐resolved photoluminescence (TRPL) measurements in dilute degassed toluene solution (Figure S81) reveal no delayed emission for all of the compounds. The prompt PL lifetimes, τ_P_, are 2.5, 8.3, and 12.4 ns and monoexponential for **1**, **1a** and **2**, with **2a** showing a biexponential lifetime of 33.4 ns.

To mitigate non‐radiative decay, we next explored the photophysical properties of these compounds as drop‐casted 1 wt% doped films in PMMA (Table 1; Figures 5 and S86–S90). A bathochromic shift also was observed in PMMA on Lewis adduct formation (from 401 to 466 nm for **1** to **1a**, and 458 to 538 nm for **2** to **2a**), although the magnitude of this shift is smaller in the film compared to that in toluene. Like that observed in 2‐MeTHF glass, the measured ∆*E*_ST_ decreased from **1** to **1a** (0.50–0.40 eV); however, the value for **2a** in PMMA could not be accurately determined (Figures S85 and S86). Interestingly, the *Φ*_PL_ in PMMA for both Lewis adducts was comparable to their parent compounds, despite the large bathochromic shift, 30% and 32% for **1** and **1a**, respectively, and 39% and 33% for **2** and **2a**, respectively. Note that the *Φ*_PL_ value for **2a** is significantly higher in PMMA than that observed in toluene (see Table S2 and associated discussion). Temperature‐dependent TRPL measurements revealed multiexponential decay kinetics for all of the compounds. Compounds **1** and **2** have average prompt PL lifetimes, *τ*_p, avg_ of 1.8 and 12.4 ns, respectively, with **2** also having an average delayed lifetime, *τ*_d, avg_, of 38.0 µs. The delayed lifetime contribution decreased in intensity upon cooling, consistent with TADF. Due to the large ∆*E*_ST_ of **1** (0.50 eV), no delayed emission was observed. The TRPL measurements reveal that **1a** and **2a** each have a biexponential *τ*_p, avg_ (*τ*_p, avg_ = 3.2 and 12.0 ns, respectively) and monoexponential delayed PL lifetime (*τ*_d_ = 5546 and 0.998 µs, respectively) (Figure 5). For **2a**, this represents a significant decrease in delayed lifetime compared to that of parent **2** (from 38.0 to 0.998 µs for **2** and **2a**, respectively), which we primarily attribute to the smaller Δ*E*_ST_ for **2a**. At 77 K the delayed emission is suppressed in both **1a** and **2a**, confirming that TADF is retained upon adduct formation of these oxo‐containing DA‐TADF systems, akin to the N‐heterocycle‐containing TADF materials shown in Figure 1b.

**FIGURE 5:**
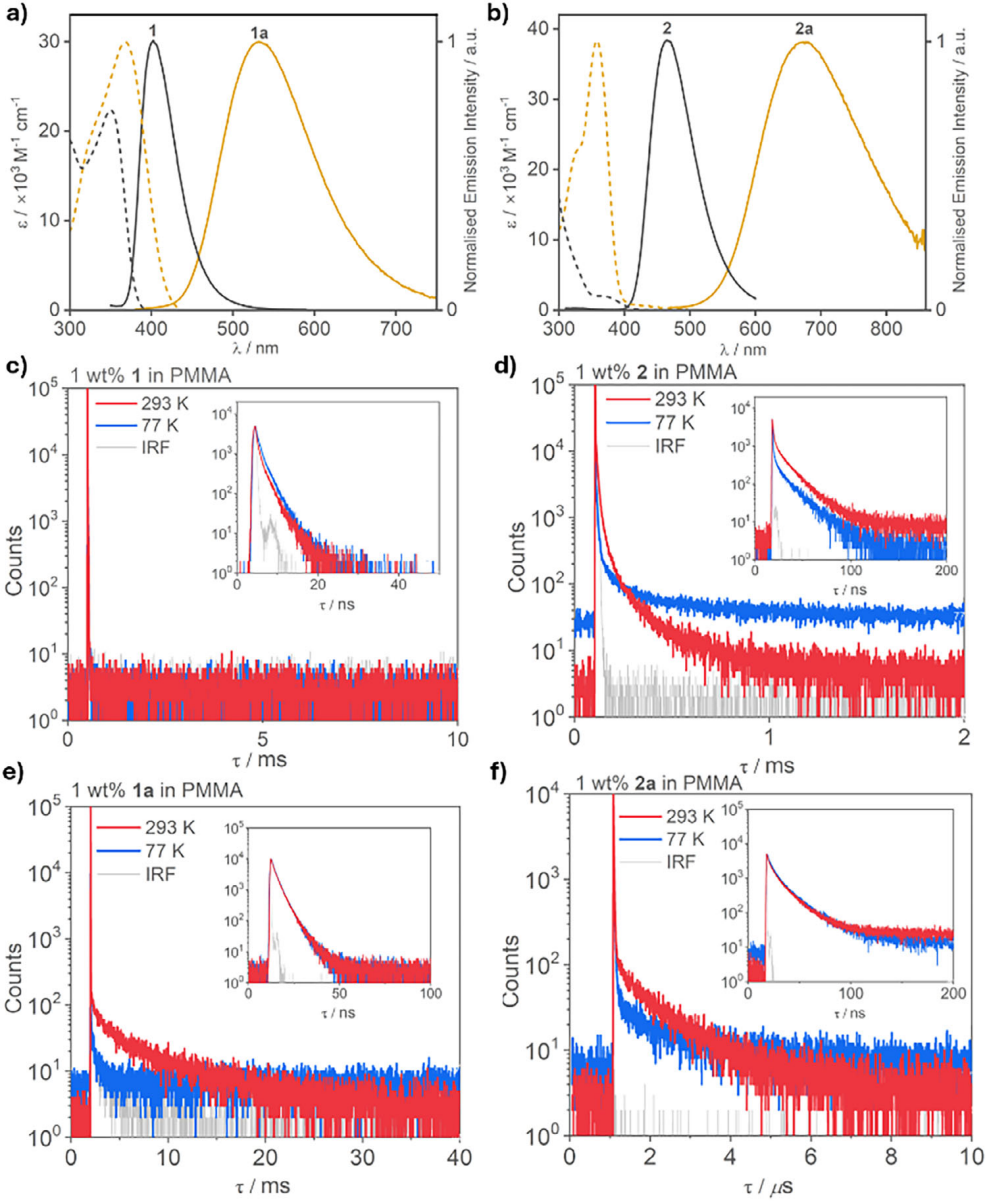
Top: absorption (dashed) and normalised PL (solid) spectra of the parent (black) and Lewis adduct (orange) of (a) **1** (*λ*_exc_  = 340 nm) and **1a** (*λ*_exc_  = 340 nm); (b) **2** (*λ*_exc_  = 366 nm) and **2a** (*λ*_exc_  = 340 nm). Bottom: time‐resolved PL decays of 1 wt% emitter in PMMA films of (c) **1** and (d) **2** (e) **1a** (f) **2a**. The prompt fluorescence lifetimes (and delayed emission lifetime of **2a**) were measured by TCSPC (*λ*_exc_  = 375 nm), whereas the delayed emission lifetimes were measured by MCS (*λ*_exc_  = 335 nm (**1**, **1a**), 320 nm (**2**)).

### 2.3 Lewis Adducts With a Bora‐Triptycene: MR‐TADF Compounds

We next turned our attention to how the photophysics of MR‐TADF emitters would be affected upon Lewis acid coordination. **DiKTa** (**3**) and **QPO** (**4**) were converted into adducts **3a** and **4a** using the same methodology used to form **1a**/**2a**. Compounds **3a/4a** were isolated in yields of 30 and 25%, respectively (Figure 6). Note that the addition of two equiv. of BtripS to **3** or **4** led to a complex mixture (by in situ multinuclear NMR spectroscopy) and only the mono‐adduct (**3a** or **4a**) could be isolated on work‐up from these mixtures. Compound **3a** contains a carbonyl‐bound BtripS, as determined by NMR spectroscopy (two new ^13^C carbonyl resonances are observed at 178.0 and 179.8 ppm, Figure S31). The two different oxygen Lewis basic sites in **4** (sulfone and carbonyl) allowed for the proclivity of BtripS binding to be determined. While DFT calculations predicted that the carbonyl‐bound adduct (**4a_1_**) is 9.8 kcal/mol more stable than the sulfone‐bound isomer (**4a_2_**), the Lewis adducts formed in an approximate 3:2 mixture of the sulfone (**4a_2_**) to carbonyl‐bound (**4a_1_**) BtripS‐bound isomers, respectively (Figure 6). This indicates that the formation of these BtripS adducts occurs under kinetic control and that adduct formation is irreversible. Single crystals of **4a_2_** were isolated in small quantities from the mixture of isomers, enabling X‐ray diffraction studies that confirmed the structural assignment. The solid‐state structure of **4a_2_** represents a rare example of a crystallographically characterised neutral sulfone bound to a Lewis acid. Comparing the S═O bond lengths in **4a_2_**, the uncoordinated S═O has a bond length of 1.4291(12) Å, while the S═O–BtripS lengthens to 1.4910(12) Å. Analogous S═O bond elongation was observed in the only other reported boron‐based Lewis acid bound neutral sulfone [33]. As with **1a** and **2a**, Lewis adducts **3a** and **4a** are stable in solution upon prolonged exposure to air and excess water (by NMR spectroscopy, see Figures S68 and S71).

**FIGURE 6:**
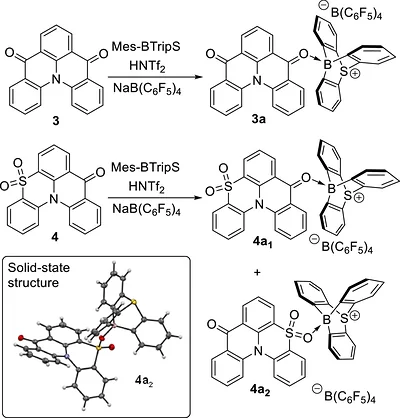
The synthesis of Lewis adducts **3a** and **4a** from **3** and **4**. Note: **4a** is isolated as a 3:2 mixture of the carbonyl (**4a_1_**) and sulfone (**4a_2_**) bound Lewis adducts. Inset bottom, solid‐state structure of **4a_2_**, ellipsoids at 50% probability level and with the [B(C_6_F_5_)_4_]^–^ anion omitted for clarity.

It has been demonstrated previously that TD‐DFT calculations do not provide an accurate prediction for the excited‐state energies of MR‐TADF compounds [34]. Therefore, for **3/3a** and **4/4a** these were calculated at the ADC(2)/cc‐pVDZ level of theory, in the gas phase (Figure 7a). There is a significant stabilisation of both the *S*_1_ (from 3.24 to 2.59 eV) and *T*_1_ (from 2.97 to 2.30 eV) states in the Lewis adducts similar to those calculated for the DA‐TADF compounds. This is driven by a large stabilisation in the LUMO on binding of the Lewis acid. Regarding the two isomers of **4a**, **4a_1_** has the more stabilised orbital and state energies, with an *S*_1_ energy 0.61 eV lower than that of **4a_2_**. The ∆*E*_ST_ values for **3a**, **4a_1_** and **4a_2_** (∆*E*_ST_ = 0.25–0.32 eV) are comparable to their parent emitters.

**FIGURE 7:**
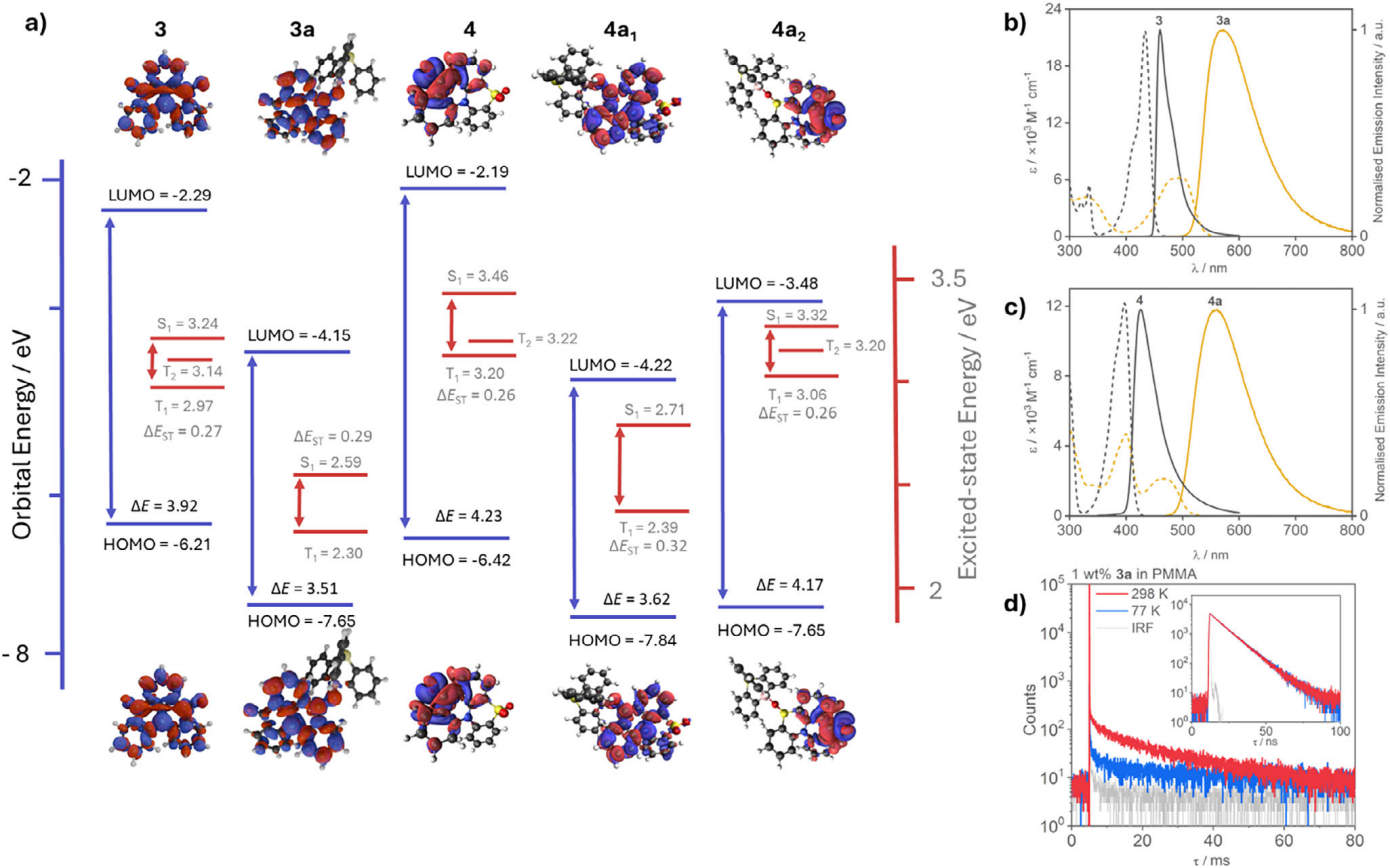
(a) Computed ground‐state (blue calculated at the PBE0/6‐31G(d,p) (SMD toluene) level of theory) and excited‐state energies (red calculated at the ADC(2)/cc‐pVDZ level of theory in gas phase) of **3**, **3a**, **4, 4a_1_** and **4a_2_**. The images are the natural transition orbitals of the *S*_1_ (top) and *T*_1_ (bottom) (isovalue = 0.02). (b) Absorption (dashed) and normalised PL (solid) spectra of **3** (*λ*_exc_  = 378 nm, grey) and **3a** (*λ*_exc_  = 450 nm, orange). (c) Absorption (dashed) and normalised PL (solid) spectra of **4** (*λ*_exc_ = 360 nm, grey) and **4a** (*λ*_exc_ = 450 nm, orange). (d) Time‐resolved PL decays of 1 wt% doped films of **3a**. Prompt PL decay (inset) measured by TCSPC (*λ*_exc_ = 375 nm), and delayed PL decay measured by MCS (*λ*_exc_  = 330 (**3**) or 340 nm (**3a**)).

CV and DPV experiments did not display any oxidation waves within the solvent window for **3/3a** and **4/4a** (Figure S83). Reduction waves were detected for all compounds, with a first irreversible reduction event observed for **3** and **3a** at −1.32 and −0.45 V, respectively, versus SCE. These correspond to LUMO energies of −3.05 eV for **3** (reported in the literature to be −3.11 eV, recorded in MeCN) [7] and −3.90 eV for **3a**; a stabilisation of 0.85 eV. While a reduction process was observed for **4** at −1.42 V versus SCE (LUMO: −2.93 eV, literature value −2.93 eV, recorded in DCM), for **4a** two reduction processes were detected at −0.42 and −0.97 V versus SCE, corresponding to LUMO energies of −3.92 and −3.37 eV [21]. These have been assigned to the reduction of isomers **4a_1_** and **4a_2_**, respectively, based on the relative LUMO energies from the DFT calculations (Figure 7a).

The lowest‐energy absorption band for **3** at *λ*_abs_ 434 nm in dilute toluene solution, assigned to the *S*_0_→*S*_1_ transition, is bathochromically shifted in **3a** to 500 nm. As with the DA‐TADF emitters, the PL spectrum of **3a** at *λ*_PL_ of 562 nm is strongly redshifted compared to that of **3** at *λ*_PL_ of 451 nm. This behaviour is conserved in drop‐casted 1 wt% doped films in PMMA (from 468 to 553 nm for **3** and **3a**, respectively). Despite the large redshift in the PL, a relatively small decrease in *Φ*_PL_ was observed in toluene (from 77% to 62%). MR‐TADF emitters, such as **3**, typically display a small positive PL solvatochromism as a result of the SRCT nature of the emissive excited state [35, 36]. A SRCT emissive excited state is assigned based in part on the PL solvatochromism behaviour of **3a**, which in fact displays an attenuated PL solvatochromism compared to **3**. The Lippert–Mataga plots (Figure S96) are consistent with the calculations (Figure S111), which indicate a smaller change in the transition dipole moment between the *S*_0_ and *S*_1_ states for **3a** relative to **3**. TRPL measurements in degassed toluene of **3** and **3a** reveal no delayed emission, which is not unusual for MR‐TADF compounds possessing moderate ∆*E*_ST_ [37, 38]. An increase in *τ*_P_ from 4.5 ns (**3**) to 10.4 ns (**3a**) was recorded. When doped at 1 wt% in PMMA, a 95 nm redshift was observed from **3** to **3a**. TRPL measurements show an increase in *τ*_p_ (from *τ*_p, avg_ = 3.1 to *τ*_p_ = 10.2 ns), and a significant increase in delayed lifetime *τ*_d_ (from *τ*_d, avg_ = 353.7 to *τ*_d_ = 13905 µs. The difference in the delayed lifetimes is attributed to the presence of an intermediate *T*_2_ state in **3** that can mediate RISC; in contrast, for **3a,** where the *T*_2_ state is much higher in energy than the *S*_1_ state, and so does not participate in the RISC channels (for further discussion and *k*_isc_/*k*_risc_ values see Table S3 and S4 and associated text). Crucially, temperature‐dependent TRPL measurements evidence that delayed emission is suppressed at 77 K, consistent with TADF (Figures 7d and S90).

For **4a**, while ADC(2) calculations predicted two distinct emission bands, one for each isomer (Figure 7a and S109) only one emission band was observed (toluene; *λ*_PL_ = 551 nm, *Φ*_PL_ = 44%, film; *λ*_PL_ = 530 nm, *Φ*_PL_ = 30%). As the calculated oscillator strength for the *S*_0_–*S*_1_ transition of **4a_1_** (0.24) is considerably larger than that of **4a_2_** (0.01), the observation of only one band is potentially due to the emission being dominated by **4a_1_**. However, due to the presence of the two inseparable isomers of **4a**, it is not appropriate to discuss the photophysics of **4a** at length, beyond noting that the mixture of the two Lewis adduct isomers has TADF character and emission that has been significantly redshifted relative to **4** (see Figure 7), and that the delayed emission is suppressed at 77 K (Figure S93). Thus, Lewis acid binding to MR‐TADF materials **3** and **4** is an effective method for redshifting emission, while retaining TADF character.

## 3 Conclusion

A detailed study of the optoelectronic properties of four Lewis adducts derived from TADF fluorophores (both DA‐ and MR‐) is presented, enabled by using a thia‐bora‐triptycene (BTripS) Lewis acid. Importantly, the formed dative bonds between BTripS and the TADF emitters containing diaryl‐sulfone and ‐carbonyl Lewis basic groups are stable to hydrolysis. This is in contrast to the analogous Lewis adducts formed from combining B(C_6_F_5_)_3_ with these fluorophores. The Lewis adducts involving B(C_6_F_5_)_3_ were sensitive to even trace water, rapidly forming H_2_O‐B(C_6_F_5_)_3_. This stability (to H_2_O) disparity highlights the importance of unambiguously characterising the products from combining Lewis basic TADF materials with Lewis acids. This is particularly important when the by‐product from hydrolysis is a strong Brønsted acid, as is the case with H_2_O‐B(C_6_F_5_)_3_, as these can also induce a redshift in emission, albeit lower in magnitude relative to that from Lewis adduct formation.

In each of the four cases studied herein, Lewis adduct formation using the BTripS Lewis acid leads to large (> 100 nm) redshifts in emission driven by dramatic LUMO stabilisation, which occurs while preserving a sufficiently narrow *S*_1_–*T*_1_ energy gap. These Lewis adducts therefore all retain the TADF properties of the parent fluorophores while displaying improved photoluminescence quantum yields in the solid‐state. Notably, the short‐range charge transfer character of the carbonyl‐based emitters (e.g., **DiKTa, 3**) is retained upon Lewis adduct formation. Lewis acid binding therefore offers a new approach to access longer emission wavelength MR‐TADF compounds, which is currently a considerable outstanding challenge in the field [39, 40, 41, 42, 43].

## Author Contributions

**Mathew J. Cross**: conceptualization, methodology, investigation, validation, visualization, writing – original draft, writing – review and editing, formal analysis and data curation. **Emily L. Hanover**: conceptualization, methodology, investigation, validation, visualization, writing – review and editing, writing – original draft, formal analysis and data curation. **Arnaud Osi**: conceptualization, methodology and investigation. **Eli Zysman‐Colman**: conceptualization, methodology, resources, validation, supervision, writing – original draft, funding acquisition, writing – review and editing and validation. **Michael J. Ingleson**: conceptualization, methodology, validation, funding acquisition, writing – original draft, writing – review and editing, supervision and resources.

## Conflicts of Interest

The authors declare no conflicts of interest.

## Supporting information




**Supporting File 1**: anie74951‐sup‐0001‐SuppMat.pdf.

**Supporting File 2**: anie74951‐sup‐0002‐SuppMat.cif.

**Supporting File 3**: anie74951‐sup‐0003‐SuppMat.xyz.

## Data Availability

The data that support the findings of this study are openly available^[^ ^44^ ^]^ in Pure at https://doi.org/10.17630/1839aab6‐ac39‐4d60‐8376‐ca7e7f8c2e9e, reference number 336134752.
