# Supplementary material for: Redshifting TADF Emission With a Bora‐Triptycene Lewis Acid
Source: Angew Chem Int Ed Engl. 2026 Sep 26;65(46):e3353986. doi: 10.1002/anie.3353986 (PMC13749305; doi:10.1002/anie.3353986)
Supplement: Supplementary file 1 [file ANIE-65-e3353986-s003.pdf]

Electronic supporting information for manuscript:

Red-shifting TADF emission with a bora-triptycene Lewis acid

M. J. Cross[a†], E. L. Hanover[ab†], A. Osi[a], E. Zysman-Colman*[b], M. J. Ingleson*[a]

[a] EaStCHEM School of Chemistry, University of Edinburgh, Edinburgh, EH9 3FJ, United Kingdom. E-mail: Michael.ingleson@ed.ac.uk

[b] Organic Semiconductor Centre, EaStCHEM School of Chemistry, University of St Andrews, St. Andrews, KY16 9ST, United Kingdom. E-mail: eli.zysman-colman@st-andrews.ac.uk

† These authors contributed equally to this work

Contents

1. Experimental 3
1.1 General procedures 3
1.2 Cyclic voltammetry 4
1.3 Photophysical analysis procedures 4
Fitting of time-resolved photoluminescence measurements 5
1.3 Syntheses 7
  1.3.1 The synthesis of 1a (DPA-DPS-BtripS [B(C_6F_5)$_4$]) 7
  1.3.2 The synthesis of 2a (DMAC-DPS-BtripS [B(C_6F_5)$_4$]) 10
  1.3.3 The synthesis of 3a (DiKTa-BtripS [B(C_6F_5)$_4$]) 13
  1.3.4 The in-situ NMR-scale synthesis of 3-BCF (DiKTa-BCF) 15
  1.3.5 The synthesis of 4a (QPO-BtripS [B(C_6F_5)$_4$]) 16
1.4 NMR spectra 19
  1.4.1 NMR spectra of 1a (DPA-DPS-BtripS [B(C_6F_5)$_4$]) 19
  1.4.2 NMR spectra of 2a (DMAC-DPS-BtripS [B(C_6F_5)$_4$]) 24
  1.4.3 NMR spectra of 3a (DiKTa-BtripS [B(C_6F_5)$_4$]) 29
  1.4.4 NMR spectra of 3-BCF (DiKTa-BCF) 34
  1.4.5 NMR spectra of 4a (QPO-BtripS [B(C_6F_5)$_4$]) 42
1.5 NMR experiments 47
  1.5.1 Attempted adduct formation with 2 (DMAC-DPS) and BCF 47
  1.5.2 Low-temperature ^{1}H NMR spectrum of 3-BCF (DiKTa-BCF) 50
  1.5.3 1a (DPA-DPS BtripS [B(C_6F_5)$_4$]) stability 51
  1.5.4 2a (DMAC-DPS BtripS [B(C_6F_5)$_4$]) stability 52
  1.5.5 3a (DiKTa BtripS [B(C_6F_5)$_4$]) stability 53
  1.5.6 3-BCF (DiKTa-BCF) stability 54
  1.5.7 4a (QPO BtripS [B(C_6F_5)$_4$]) stability 56

1.5.8 A comparison of Lewis vs Brønsted acid effects on DiKTa (3-BCF) 57
1.6. Crystallography 60
1.6.1 Crystallographic details 60
1.6.2 Crystallographic data tables 61
1.7 Photophysical characterisation 62
1.7.6 3-BCF (DiKTa-BCF) 62
1.7.7 2 (DMAC-DPS) + H_2O-BCF 63
1.7.8 BtripS adducts 1 – 4 and 1a – 4a 64
1.8 Computational details 81
1.8.1 1,3,5-Triphenyltriazine BCF binding calculations 81
1.8.2 Lewis acid bathochromic shift model orbital calculations 82
1.8.3 DMAC DPS BF_3 MO and NBO calculations 82
1.8.4 DiKTa BF_3 MO and NBO calculations 85
1.8.5 TDDFT analysis 87
1.8.6 TADF emitters 1a & 2a 87
1.8.7 MR-TADF emitters 3a, 3-BCF & 4a 88
1.9 Conformer Analysis for 2 and 2a and discussion of ΔE_{ST} for 2a 91
1.10 References 95

1. Experimental

1.1 General procedures

Manipulations under inert atmospheres were performed under argon (glovebox) or nitrogen (Schlenk line) using standard techniques. Glassware was oven dried at 120 °C prior to use. Pentane, DCM, Et_2O were dried on a Grubbs'-type solvent purification system and stored under nitrogen over 3 Å molecular sieves. *Ortho*-difluorobenzene was dried by vacuum distillation over CaH_2, then degassed by three successive freeze-pump-thaw cycles and stored under nitrogen over 3 Å molecular sieves. EtOAc and petroleum ether (40-60) for column chromatography were purchased as technical grade and used as supplied. THF-d_8 was dried by vacuum distillation from CaH_2, refluxed over potassium metal for 12 hours and stored under vacuum over a potassium mirror, from which the solvent was then vacuum transferred into each NMR sample. $CDCl_3$ was dried by vacuum distillation over CaH_2 and stored under nitrogen over 3 Å molecular sieves. $HNTf_2$, potassium metal and CaH_2 were used as supplied, without further purification. Precursors $NaB(C_6F_5)_4$,[1] mes-BtripS,[2] DPA-DPS (**1**),[3] DMAC-DPS (**2**),[4] DiKTa (**3**),[5] and QPO (**4**)[6] were prepared according to literature procedures. Tris(pentafluorophenyl)borane (BCF) dried according to the reported procedure.[7] Analysis by ^{11}B and ^{19}F NMR spectroscopy was used to confirm free BCF had been formed (or there was a fast exchange equilibrium favouring free BCF).

Column chromatography was performed on Teledyne Isco CombiFlash® 100 instrument using Teledyne Redisep® columns.

NMR spectra were recorded on Bruker Avance III 500 MHz, Bruker PRO 500 MHz or Bruker Ultrashield 600 MHz NMR spectrometers at 298 K unless otherwise specified. ^{1}H NMR spectra recorded in deuterated solvents were referenced to the residual protio-solvent signals. ^{10}B, ^{11}B and ^{19}F spectra were indirectly referenced to the deuterated lock solvent. All ^{13}C NMR spectra were recorded with ^{1}H decoupling. All chemical shifts (δ) are reported in ppm and coupling constants (J) are reported in Hz.

Mass spectrometry ESI-MS was recorded on a Bruker solariX 12T FT-ICR. ESI samples were first dissolved in CH_2Cl_2 and run in MeOH carrier solvent. MALDI samples were dissolved in CH_2Cl_2 and mixed with a 2,5-dihydroxy benzoic acid matrix (prepared as 20 mg/mL with 0.1% formic acid).

1.2 Cyclic voltammetry was performed on an Electrochemical Analyzer potentiostat model 620E from CH Instruments at a sweep rate of 100 mV/s. Samples were prepared as dichloromethane (DCM) solutions, which were degassed by sparging with DCM-saturated N_2 gas for 5 minutes prior to measurements. All measurements were performed using 0.1 M DCM solution of tetra-n-butylammonium hexafluorophosphate ($[^nBu_4N]PF_6$). An Ag/Ag^+ electrode was used as the reference electrode, a glassy carbon electrode as the working electrode and a platinum wire as the counter electrode. The redox potentials are reported relative to a saturated calomel electrode (SCE) with a ferrocenium/ferrocene (Fc^+/Fc) redox couple as the internal standard (0.46 V vs SCE).[8] Differential pulse voltammetry (DPV) was conducted with an increment potential of 0.01 V and a pulse amplitude, width, and period of 50 mV, 0.06, and 0.5 s, respectively.

1.3 Photophysical analysis procedures

Optically dilute solutions of concentrations on the order of 10^{-4} to 10^{-6} M were prepared in either spectroscopic or HPLC grade solvents for absorption and emission analyses. Absorption spectra were recorded in toluene at room temperature on a Shimadzu UV-2600 double beam spectrophotometer with a 1 cm quartz cuvette. Water content as supplied in the HPLC grade toluene is 100 ppm, corresponding to ~ 5×10^{-3} M. Molar absorptivity determination was verified by linear regression analysis of values obtained from four or more independent solutions at varying concentrations ranging from 10^{-4} M to 10^{-6} M and the R^2 value for the linear fit of the dataset surpassing 0.98. Steady-state photoluminescence (PL) and excitation spectra and time-resolved PL decays were recorded at 298 K using an Edinburgh Instruments FS5 spectrofluorometer.

For solution-state PL quantum yield (Φ_{PL}) measurements, degassed solutions were prepared via three freeze-pump-thaw cycles and spectra were taken using custom-made Schlenk quartz cuvettes in spectroscopic grade toluene. Φ_{PL}s were determined using the optically dilute method in which three or more sample solutions with absorbances between 0.10 and 0.01 were measured.[9] The Beer-Lambert law was found to remain linear at the concentration of the solutions. For each sample, linearity between absorption and emission intensity was verified through linear regression analysis with the R^2 value for the linear fit of the dataset surpassing 0.98. The Φ_{PL} of the sample, Φ_{PL}, was determined using $\Phi_{PL} = \Phi_r * \frac{A_r}{A_s} * \frac{I_s}{I_r} * \frac{n_s^2}{n_r^2}$, where A is the

absorbance at the excitation wavelength, *I* is the integrated area under the corrected emission curve and *n* is the refractive index of the solvent, with subscripts '*s*' and '*r*' representing sample and reference respectively. Φ_r is the absolute quantum yield of the external reference; either quinine sulfate (Φ_r = 54.6% in 1 M H_2SO_4, aerated), $[Ru(bpy)_3]^{2+}$ (Φ_r = 1.8% in MeCN, aerated) or rhodamine 6G (Φ_r = 94% in EtOH, aerated).[9] Individual relative Φ_{PL} values were calculated for each solution and the values reported represent the slope obtained from the linear fit of these results. The experimental uncertainty in the Φ_{PL} values is conservatively estimated to be 10%, though we have found that statistically we can reproduce Φ_{PL} values to within 3% relative error.

Thin films, either neat or doped in a host matrix, were spin-coated on a quartz substrate (for Φ_{PL} measurements) or a sapphire substrate (for time-resolved PL, temperature-dependent emission and film ΔE_{ST} measurements) from benzene. Spin coating was performed at a spin speed of 1250 rpm for 60 s. Φ_{PL} of the films was measured using an integrating sphere (Edinburgh Instruments FS5, SC30 module) first in air then in N_2 by purging the integrating sphere with N_2 for 2 minutes. Time-resolved PL measurements were carried out using the multi-channel scaling (MCS) and time-correlated single-photon counting (TCSPC) techniques. Samples were excited by a pulsed laser at 375 nm and kept in a vacuum of < 8 × 10^{-4} mbar.

The singlet and triplet state energies in 2-MeTHF glass and in doped film were determined from the onset values of the steady-state PL (SS PL) and delayed emission spectra at 77 K. The singlet-triplet energy gap (ΔE_{ST}) was estimated from the difference in energy of the onsets of these spectra. For steady-state PL and delayed emission spectra, samples were excited by a xenon lamp (EI FS5, SC-70). Delayed emission spectra were measured with a time-gated window of 1-9 ms with a xenon flashlamp operating at 100 Hz.

Fitting of time-resolved photoluminescence measurements

Time-resolved PL measurements were fitted to a sum exponentials decay model with chi-squared (χ^2) values between 1 and 2, using the Fluoracle software. Each component of the decay is assigned a weight (w_i) which is the contribution of the emission from each component to the total emission.

The average lifetime was then calculated using the following:

Two exponential decay model: $\tau_{AVG} = \tau_1 w_1 + \tau_2 w_2$

weights defined as $w_1 = \frac{A1\tau_1}{A1\tau_1 + A2\tau_2}$ and $w_2 = \frac{A2\tau_2}{A1\tau_1 + A2\tau_2}$

A1 and A2 are the pre-exponential factors of each component.

Three exponential decay model: $\tau_{AVG} = \tau_1 w_1 + \tau_2 w_2 + \tau_3 w_3$

weights defined as $w_1 = \frac{A1\tau_1}{A1\tau_1 + A2\tau_2 + A3\tau_3}$, $w_2 = \frac{A2\tau_2}{A1\tau_1 + A2\tau_2 + A3\tau_3}$ and $w_3 = \frac{A3\tau_3}{A1\tau_1 + A2\tau_2 + A3\tau_3}$

A1, A2 and A3 are the pre-exponential factors of each component.

Kinetics rate constant calculations: The kinetics constants calculated following the literature:

$$k_p = \frac{1}{\tau_p}$$

$$k_d = \frac{1}{\tau_d}$$

$$k_r^S = k_p \Phi_{PF}$$

$$k_{nr}^S = k_p \frac{\Phi_{PF}}{\Phi_{PLQY}} (1 - \Phi_{PLQY})$$

$$k_{RISC} = \frac{\Phi_{PL}}{2} * \frac{\Phi_{PL}(1 - \Phi_{PF}) + \Phi_{DF} + \Phi_{PF}(1 - \Phi_{PL})}{\Phi_{PF}(1 - \Phi_{PF})}$$

$$k_{ISC} = \frac{\left[k_p(1 - \Phi_{PF}) - k_d \Phi_{PF}\right]\Phi_{PL} + k_p \Phi_{PF}\Phi_{DF} + \left[k_p \Phi_{PF}^2 (1 - \Phi_{PL}) + k_d \Phi_{DF}\Phi_{PL}\right]}{2\Phi_{PF}\Phi_{PL}}$$

Where Φ_{PF} and Φ_{DF} are the prompt and delayed efficiencies.

1.3 Syntheses

1.3.1 The synthesis of 1a (DPA-DPS-BtripS [$B(C_6F_5)_4$])

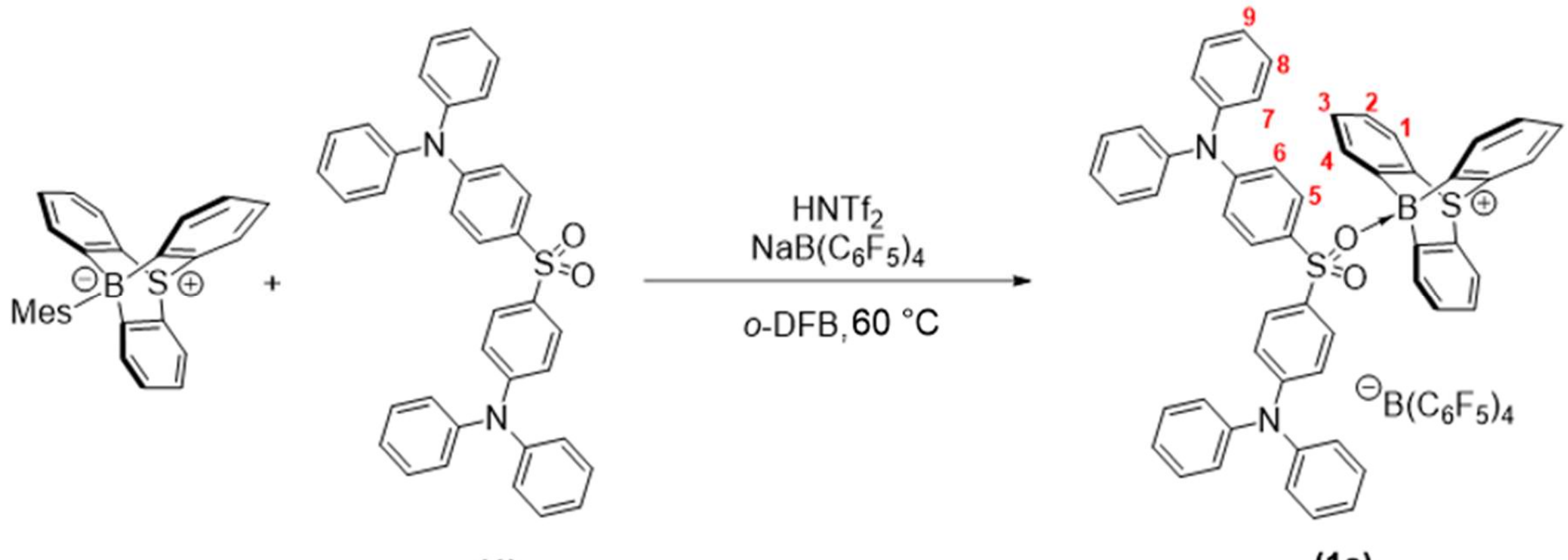

*Notice: Compound (**1a**) is prone to decomposition upon exposure to sources of UV light in solution.*

Under an atmosphere of nitrogen, 0.200 g (0.38 mmol) of DPA-DPS (**1**), 0.124 g (0.32 mmol) of Mes-BtripS, 0.091 g (0.32 mmol) of $HNTf_2$ and 0.225 g (0.32 mmol) of $NaB(C_6F_5)_4$ were added to an ampoule and then suspended in 2 cm^3 of *ortho*-difluorobenzene with stirring. This reaction mixture was heated to 60 °C for 16 hours, during which the colour changed from pale-yellow to green, before cooling to room temperature. After cooling, the solvent was removed *in-vacuo* and the solids were purified by column chromatography on silica with an eluent of EtOAc/pet ether, where the solvent ratio was varied from 30% to 70% EtOAc. The fractions containing the green product (R_f = ~ 0.1 in 1:1 EtOAc:pet ether) were collected and the solvent removed *in-vacuo*, leaving a green solid. This solid was then dried under a high-vacuum (10^{-3} mbar) to give **1a** as a green powder (0.160 g, 34% yield).

To obtain **1a** free of all trace contaminants (e.g., very low quantities of entrained solvent, visible by NMR spectroscopy) the following procedure was used. The previously obtained **1a** was run through a second silica chromatography column, with an eluent of EtOAc/pet ether, where the solvent ratio was varied from 30% to 70% EtOAc. The fractions containing **1a** were collected and the solvent removed in-vacuo. The green solids obtained were then dissolved in 2 cm^3 of $CHCl_3$ and filtered, where the green filtrate was evaporated to dryness *in-vacuo*. The solid obtained was then dissolved in ~ 1 cm^3 of Et_2O and triturated with pentane. The green precipitate formed was collected via canula filtration, washed with more pentane and dried *in-vacuo* to remove all residual solvent. 0.119 g of **1a** was obtained as a green powder (25% yield).

All attempts to crystallise **1a** were unsuccessful.

^1H NMR (500 MHz, THF-d_8, 298 K): δ 8.11 (4H, d, 9.2 Hz, H-5), 7.98 (3H, dd, 7.6 Hz, 0.8 Hz, H-1), 7.76 (3H, dd, 7.4 Hz, 1.1 Hz, H-4), 7.43 (8H, dd, 8 Hz, 7.7 Hz, H-8), 7.33 (3H, td, 7.4 Hz, 1.0 Hz, H-3), 7.30 - 7.26 (12H, overlapping m, H-7 & 9), 7.22 (3H, td, 7.6 Hz, 1.3 Hz, H-2), 7.10 (4H, d, 9.2 Hz, H-6).

^{13}C NMR (125.7 MHz, THF-d_8, 298 K): 155.6, 154.3, 149.3 (dm, $^1J_{CF}$ = 250 Hz), 146.0, 139.2 (dm, $^1J_{CF}$ = 245 Hz), 137.4 (dm, $^1J_{CF}$ = 253 Hz), 133.0, 131.9, 131.2, 130.9, 130.0, 129.7, 128.2, 127.8, 126.9, 126.2, 118.9. $[B(C_6F_5)_4]^-$ B–C not observed

^{19}F NMR (470.4 MHz, THF-d_8, 298 K): δ – 132.7 (8F, dd, 16 Hz, 7 Hz, $[B(C_6F_5)_4]^-$), – 165.2 (4F, t, 21.0 Hz, $[B(C_6F_5)_4]^-$), – 168.63 (8H, t, 21.0 Hz, $[B(C_6F_5)_4]^-$).

^{11}B NMR (160.4 MHz, THF-d_8, 298 K): δ –16.6 (1B, s, $[B(C_6F_5)_4]^-$).

^{10}B NMR (53.7 MHz, THF-d_8, 298 K): δ –16.6 (1B, s, $B(C_6F_5)_4^-$). A broad low-intensity signal was also observed ~ 4 ppm, possibly corresponding to S=O-BtripS.

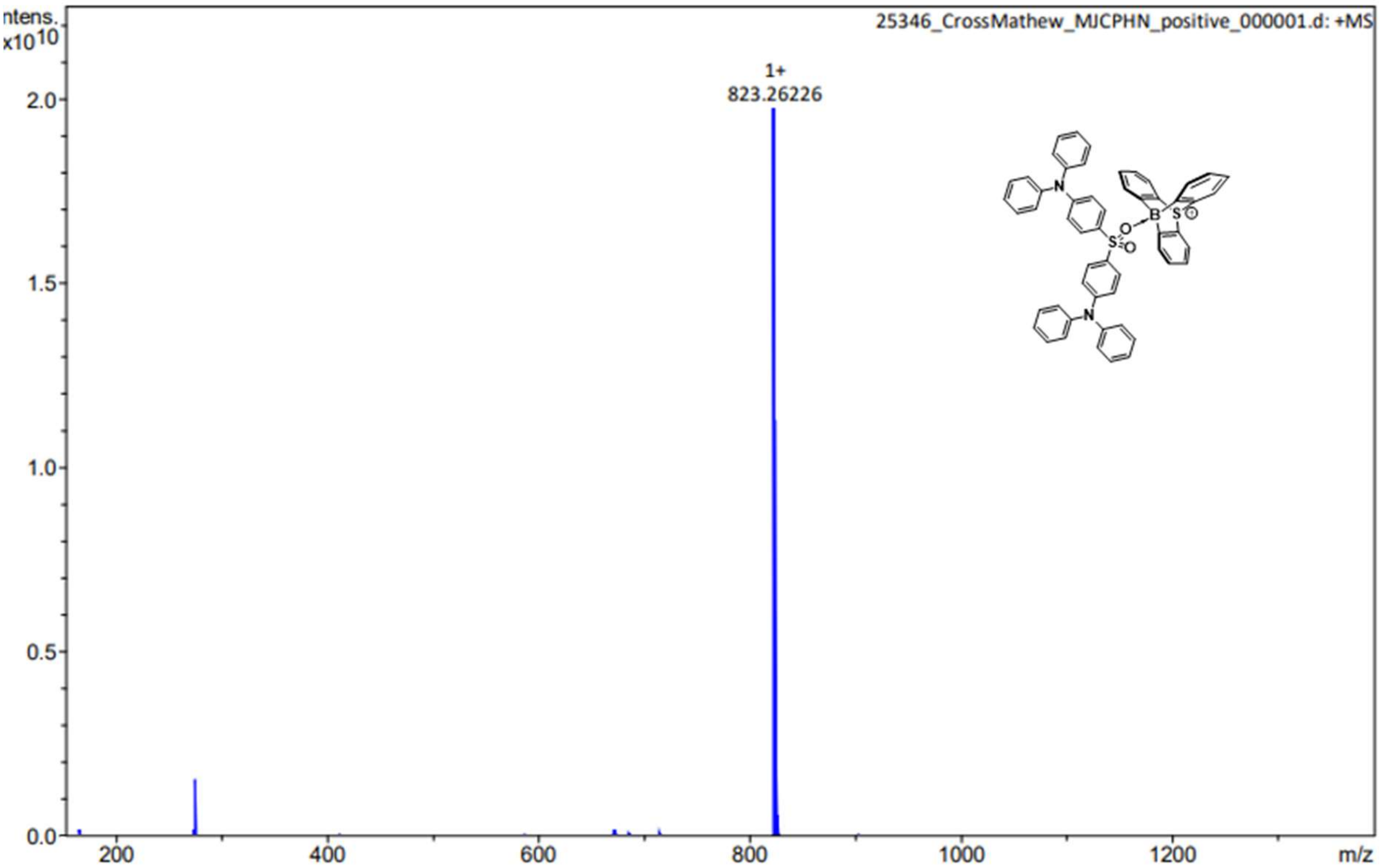

Figure S1. The ESI+ mass spectrum of **1a** showing the molecular ion $[M]^+$ at *m/z* = 823.26 (predicted 823.26). Recorded in MeOH.

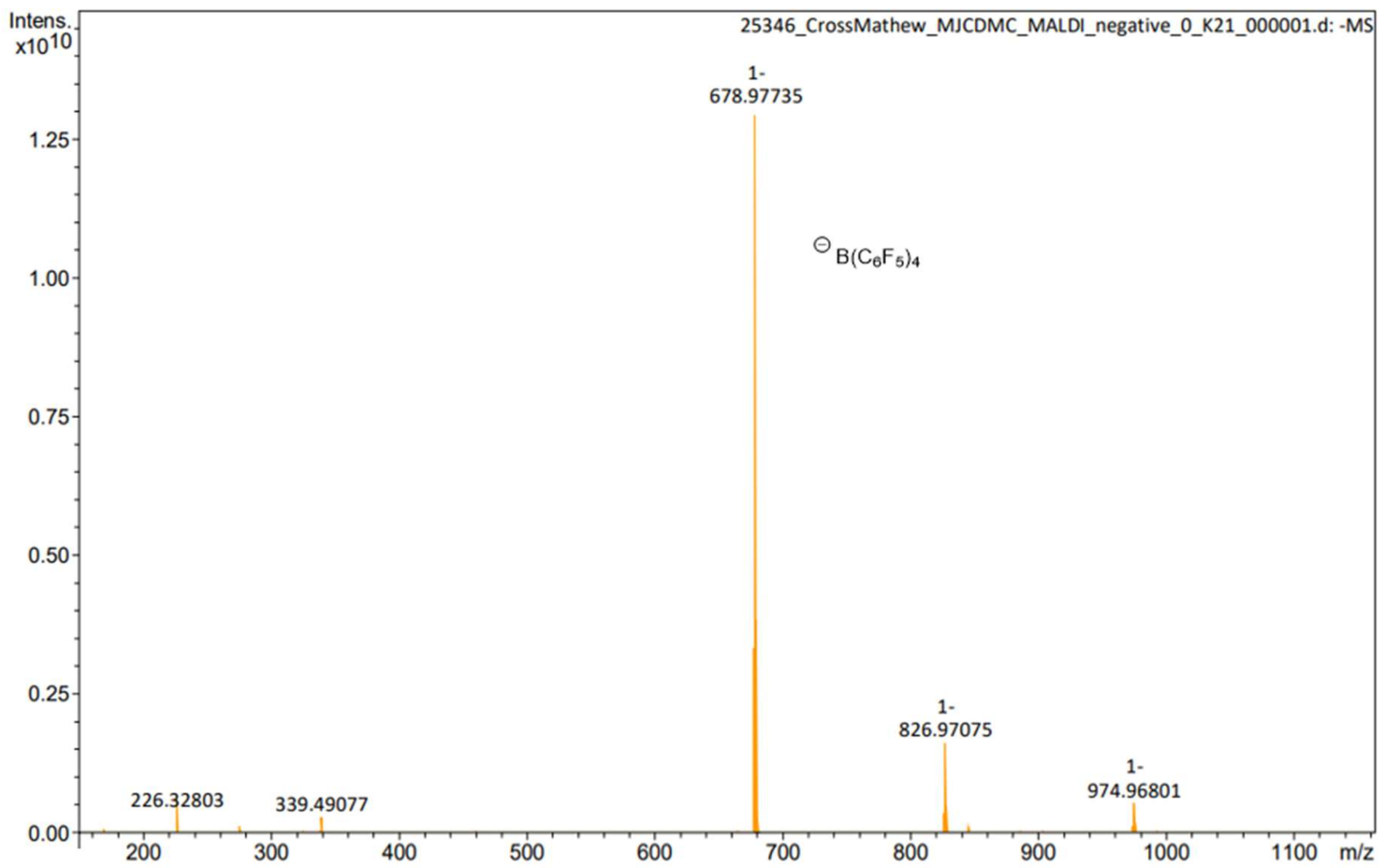

Figure S2. The MALDI- mass spectrum of **1a** showing the anion $[B(C_6F_5)_4]^-$ at *m/z* = 678.98 (predicted 678.98).

1.3.2 The synthesis of 2a (DMAC-DPS-BtripS [$B(C_6F_5)_4$])

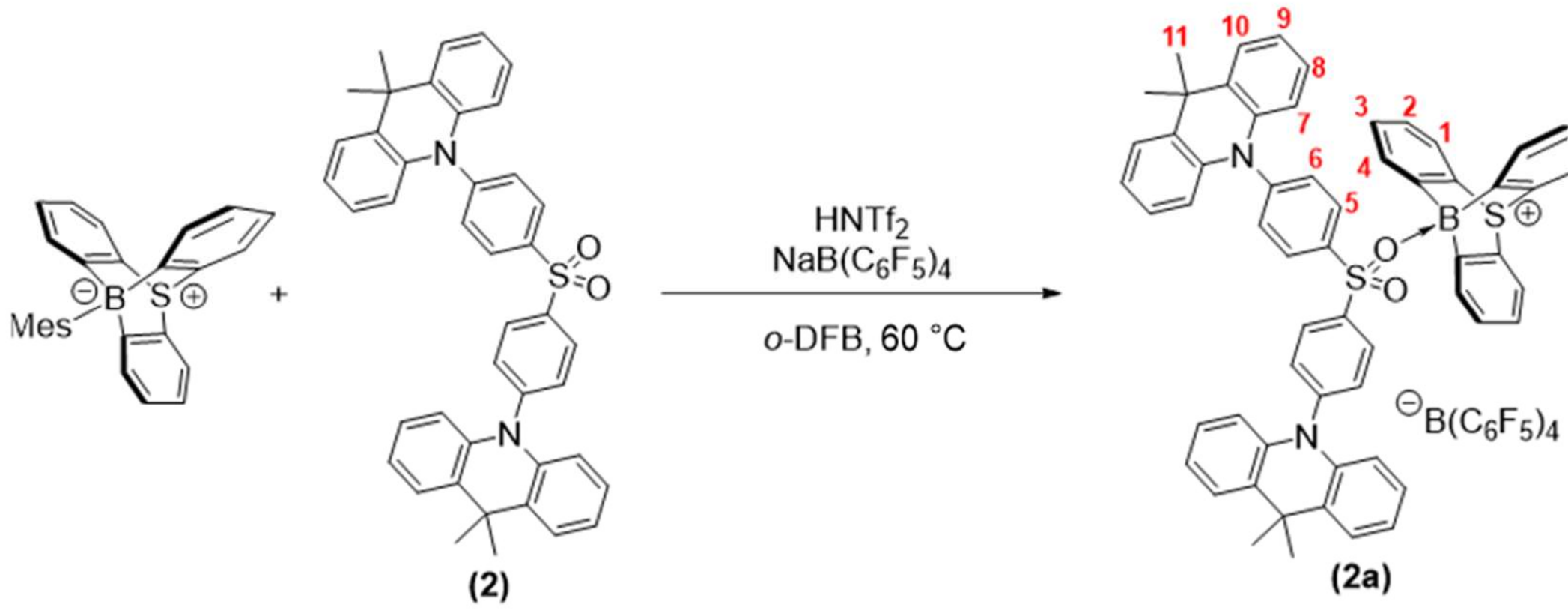

*Notice: Compound (**2a**) is prone to decomposition upon exposure to sources of UV light in solution.*

Under an atmosphere of nitrogen, 0.450 g (0.71 mmol) of DMAC-DPS (**2**), 0.250 g (0.65 mmol) of Mes-BtripS, 0.184 g (0.65 mmol) of $HNTf_2$ and 0.450 g (0.65 mmol) of $NaB(C_6F_5)_4$ were added to an ampoule and then suspended in 3 cm^3 of *ortho*-difluorobenzene with stirring. This reaction mixture was heated to 60 °C for 16 hours, during which the colour changed from yellow to green, before cooling to room temperature. After cooling, the solvent was removed *in-vacuo* and the solids were purified by column chromatography on silica with an eluent of EtOAc/pet ether, where the solvent ratio was varied from 30% to 70% EtOAc. The fractions containing the green product (R_f = ~ 0.1 in 1:1 EtOAc:pet ether) were collected and the solvent removed *in-vacuo*, leaving a yellow-green solid. This solid was then dried under a high-vacuum (10^{-3} mbar) to give **2a** as a yellow powder (0.300 g, 29% yield).

To obtain **2a** free of all trace contaminants (e.g., very low quantities of entrained solvent visible by NMR spectroscopy) the following procedure was used. The previously obtained **2a** was run through a second silica chromatography column, with an eluent of EtOAc/pet ether, where the solvent ratio was varied from 30% to 70% EtOAc. The fractions containing **2a** were collected and the solvent removed in-vacuo. The yellow-green solids obtained were then dissolved in 2 cm^3 of $CHCl_3$ and filtered, where the green filtrate was evaporated to dryness *in-vacuo*. The solid obtained was then dissolved in ~ 1 cm^3 of Et_2O and triturated with pentane. The yellow-green precipitate formed was collected via canula filtration, washed with more pentane and dried *in-vacuo* to remove all residual solvent. 0.135 g of (**2a**) was obtained as a yellow powder (13% isolated yield).

All attempts to crystallise **2a** were unsuccessful.

^1H NMR (500 MHz, THF-d_8, 298 K): δ 8.41 (4H, d, 9.2 Hz, H-5), 7.99 (3H, dd, 7.8 Hz, 0.9 Hz, H-1), 7.79 (4H, d, 9.2 Hz, H-6), 7.73 (3H, dd, 7.4 Hz, 1.1 Hz, H-4), 7.59 (4H, m, H-10), 7.30 (3H, td, 7.4 Hz, 1.1 Hz, H-3), 7.27-7.17 (15H, overlapping m, H-2, 7, 8 & 9), 1.55 (12H, s, H-11).

^{13}C NMR (125.7 MHz, THF-d$_8$, 298 K): δ 153.9, 152.4, 149.3 (dm, $^{1}J_{CF}$ = 244 Hz), 141.7, 141.2, 139.2 (dm, $^{1}J_{CF}$ = 250 Hz), 137.3 (dm, $^{1}J_{CF}$ = 247 Hz), 133.1, 131.9, 131.0, 130.8, 129.8, 129.4, 127.4, 127.0, 126.3, 126.2, 123.2, 120.8, 38.7, 28.5. $[B(C_6F_5)_4]^-$ B–C not observed.

^{19}F NMR (470.4 MHz, THF-d$_8$, 298 K): δ –132.8 (dd, 8F, 16.0 Hz, 7.0 Hz, $[B(C_6F_5)_4]^-$), –165.1 (td, 4F, 21.5 Hz, 5.7 Hz, $[B(C_6F_5)_4]^-$), – 168.6 (t, 8F, 21.5 Hz, $[B(C_6F_5)_4]^-$).

^{11}B NMR (160.4 MHz, THF-d$_8$, 298 K): δ –16.6 (1B, s, $[B(C_6F_5)_4]^-$).

^{10}B NMR (53.7 MHz, THF-d$_8$, 298 K): δ –16.6 (1B, s, $[B(C_6F_5)_4]^-$). No signal was observed for the BtripS.

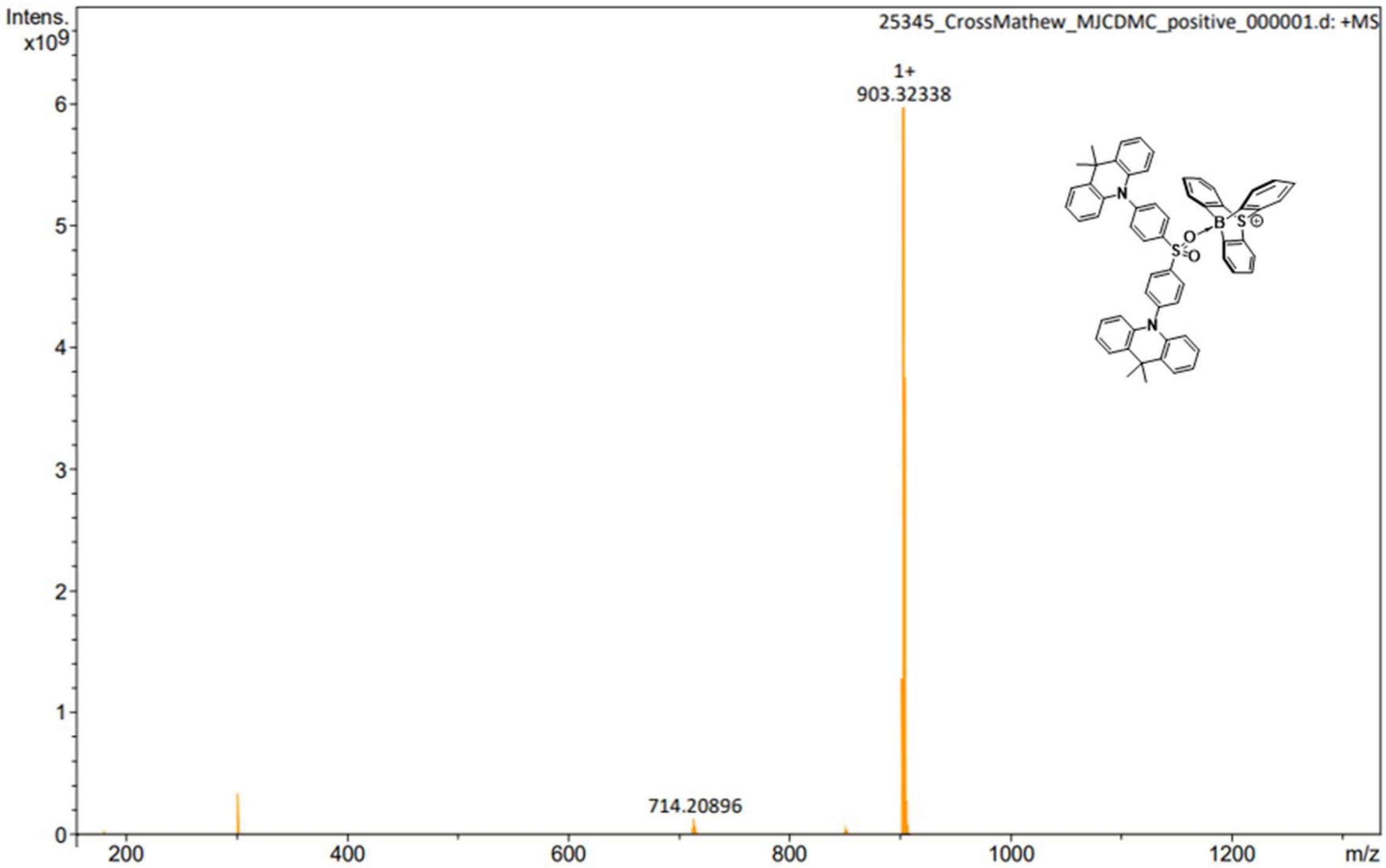

Figure S3. The ESI+ mass spectrum of **2a** showing the molecular ion $[M]^+$ at *m/z* = 903.32 (predicted 903.32) Recorded in MeOH.

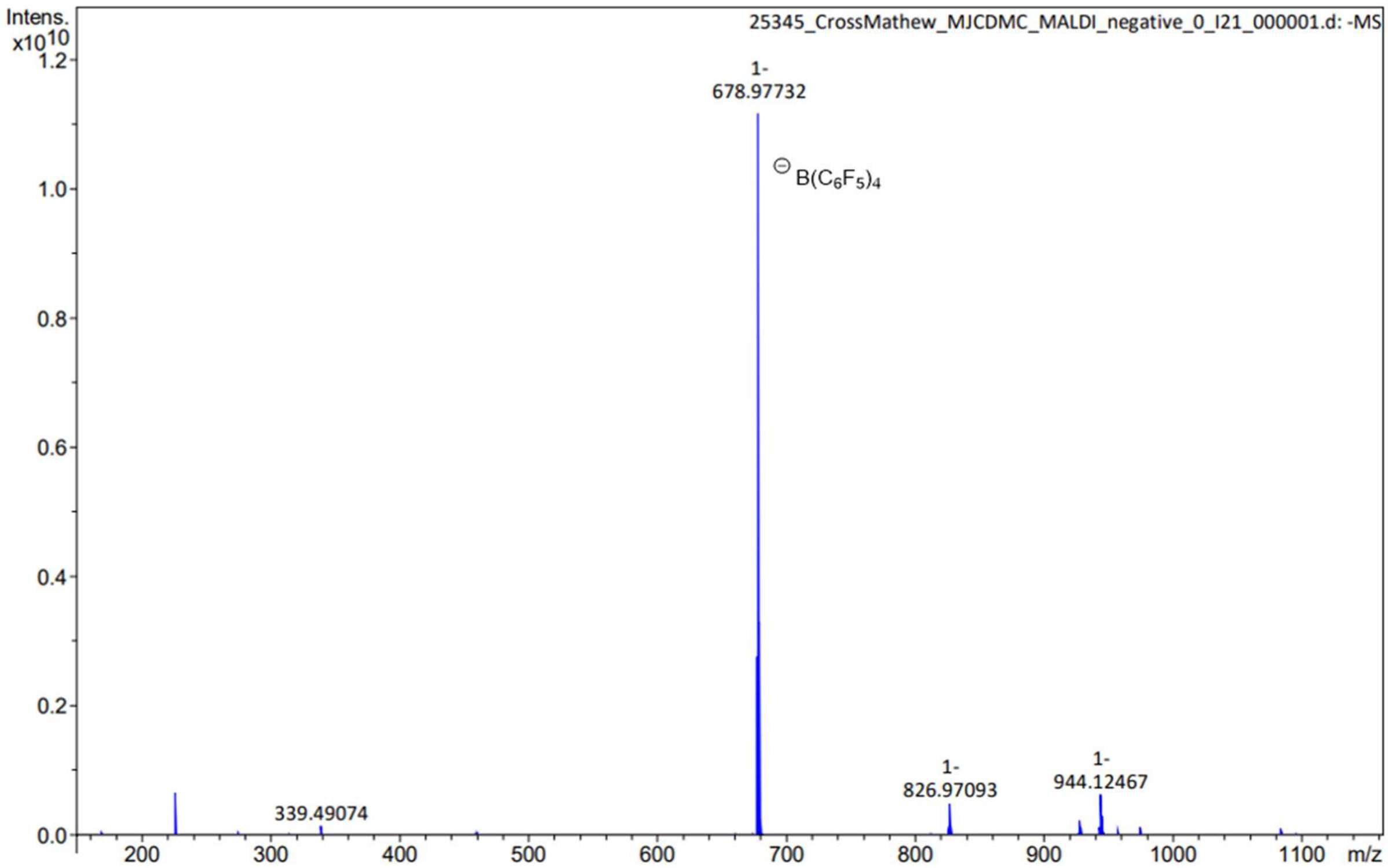

Figure S4. The MALDI- mass spectrum **2a** showing the anion $[B(C_6F_5)_4]^-$ at *m/z* = 678.98 (predicted 678.98).

1.3.3 The synthesis of 3a (DiKTa-BtripS [$B(C_6F_5)_4$])

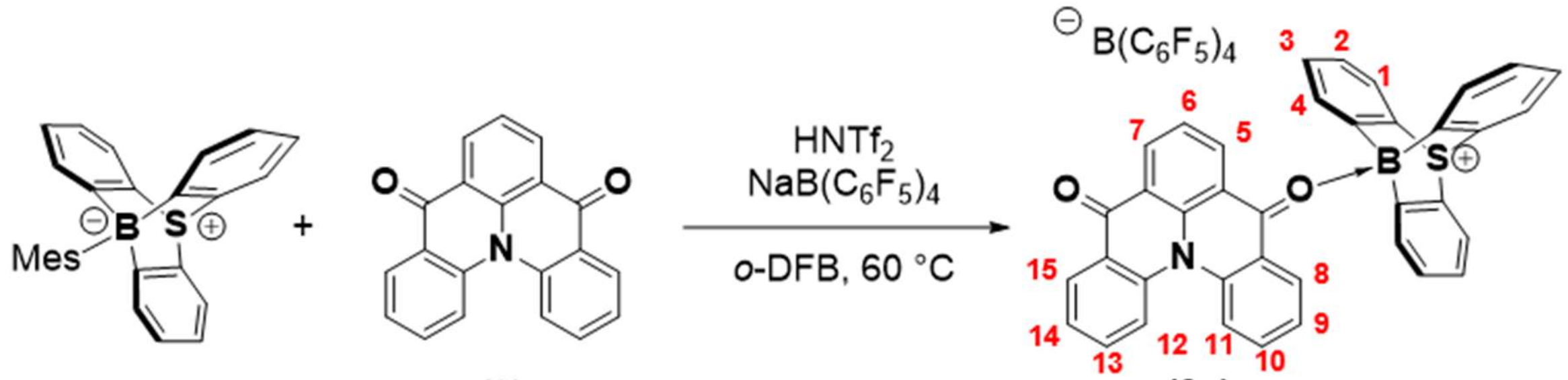

(3) (3a)

*Notice: this compound (**3a**) in solution is prone to decomposition upon exposure to sources of UV light akin to that previously reported for **3**.*[10]

Under an atmosphere of nitrogen, 0.085 g (0.29 mmol) of DiKTa (**3**), 0.094 g (0.24 mmol) of Mes-BtripS, 0.067 g (0.24 mmol) of $HNTf_2$ and 0.168 g (0.24 mmol) of $NaB(C_6F_5)_4$ were added to an ampoule and then suspended in 2 cm^3 of *ortho*-difluorobenzene with stirring. This reaction mixture was heated to 60 °C for 16 hours, during which the colour changed from yellow to red, before cooling to room temperature. After cooling, the solvent was removed *in-vacuo* and the solids were purified by column chromatography on silica with an eluent of EtOAc/pet ether, where the solvent ratio was varied from 30% to 70% EtOAc. The fractions containing the orange product (R_f = ~ 0.1 in 1:1 EtOAc:pet ether) were collected and the solvent removed *in-vacuo*, leaving an orange solid. The orange solid was dissolved in toluene and triturated with pentane, then dissolved in Et_2O and then again triturated with pentane. The resulting solid was washed with pentane, then dried under a high-vacuum (10^{-3} mbar) to give **3a** as an orange powder (0.097 g, 30% yield).

All attempts to crystallise **3a** were unsuccessful.

^{1}H NMR (500 MHz, THF-d_8, 298 K): δ 9.04 (2H, overlapping m, H-5 & H-8), 9.02 (1H, dd, 7.4 Hz, 1.5 Hz, H-7), 8.77 (1H, d, 8.8 Hz, H-11), 8.54 (1H, dd, 7.9 Hz, 1.6 Hz, H-15), 8.39 (1H, d, 8.4 Hz, H-12), 8.18 (1H, ddd, 8.6 Hz, 7.1 Hz, 1.5 Hz, H-10), 8.10 (3H, m, H-1), 7.96 (1H, ddd, 8.6 Hz, 7.3 Hz, 1.7 Hz, H-13), 7.85 (3H, m, H-4), 7.79 (1H, ddd, 8.0 Hz, 7.1 Hz, 0.7 Hz, H-14), 7.72 (1H, ddd, 8.4 Hz, 7.0 Hz, 0.8 Hz, H-9), 7.66 (1H, dd, 8.2 Hz, 7.5 Hz, H-6), 7.29 (6H, m, H-2 & H-3).

^{13}C NMR (125.7 MHz, THF-d_8, 298 K): δ 179.8 (C=O), 178.0 (C=O), 155.0, 149.4 (d, $^1J_{CF}$ = 240 Hz), 142.8, 141.1, 140.3, 139.2 (d, $^1J_{CF}$ = 250 Hz), 138.8, 138.2, 137.3 (d, $^1J_{CF}$ = 252 Hz), 136.4, 134.7, 133.8, 131.8, 131.7, 130.1, 129.7, 129.2, 128.9, 128.7, 127.6, 127.1, 125.7, 124.8, 124.7, 124.0, 122.5, 121.1. $[B(C_6F_5)_4]^-$ B–C not observed.

^{19}F NMR (470.4 MHz, THF-d_8, 298 K): δ –132.7 (dd, 8F, 16.0 Hz, 7.0 Hz, $[B(C_6F_5)_4]^-$), –165.1 (td, 4F, 21.5 Hz, 5.7 Hz, $[B(C_6F_5)_4]^-$), – 168.6 (t, 8F, 21.5 Hz, $[B(C_6F_5)_4]^-$).

^{11}B NMR (160.4 MHz, THF-d_8, 298 K): δ 5.1 (1B, s, BtripS), –16.6 (1B, s, $[B(C_6F_5)_4]^-$).

^{10}B NMR (53.7 MHz, THF-d_8, 298 K): δ 5.5 (1B, s, BtripS), –16.6 (1B, s, $[B(C_6F_5)_4]^-$).

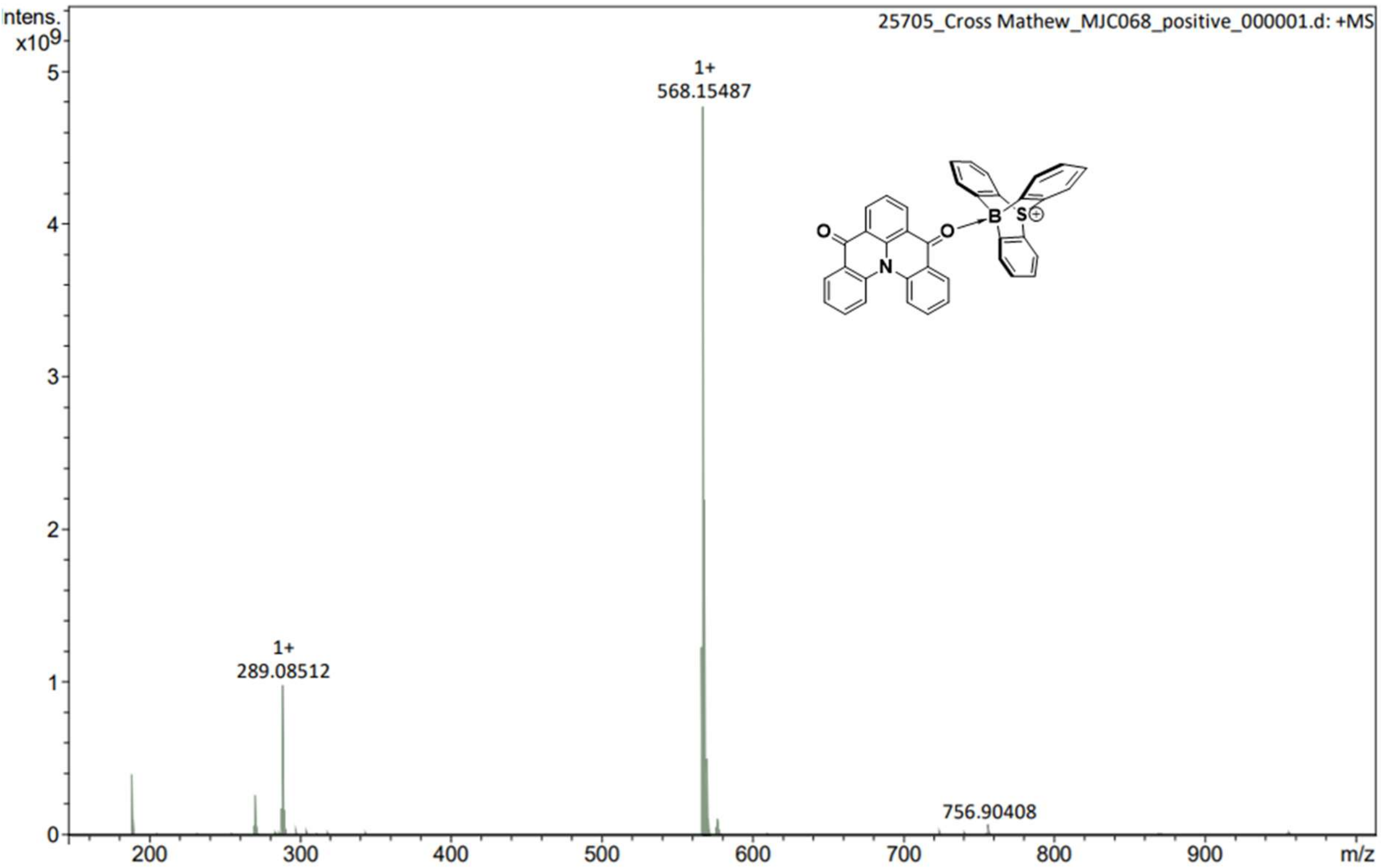

Figure S5. The ESI+ mass spectrum of **3a** showing the molecular ion $[M]^+$ at *m/z* = 568.15 (predicted 568.15). Recorded in MeOH.

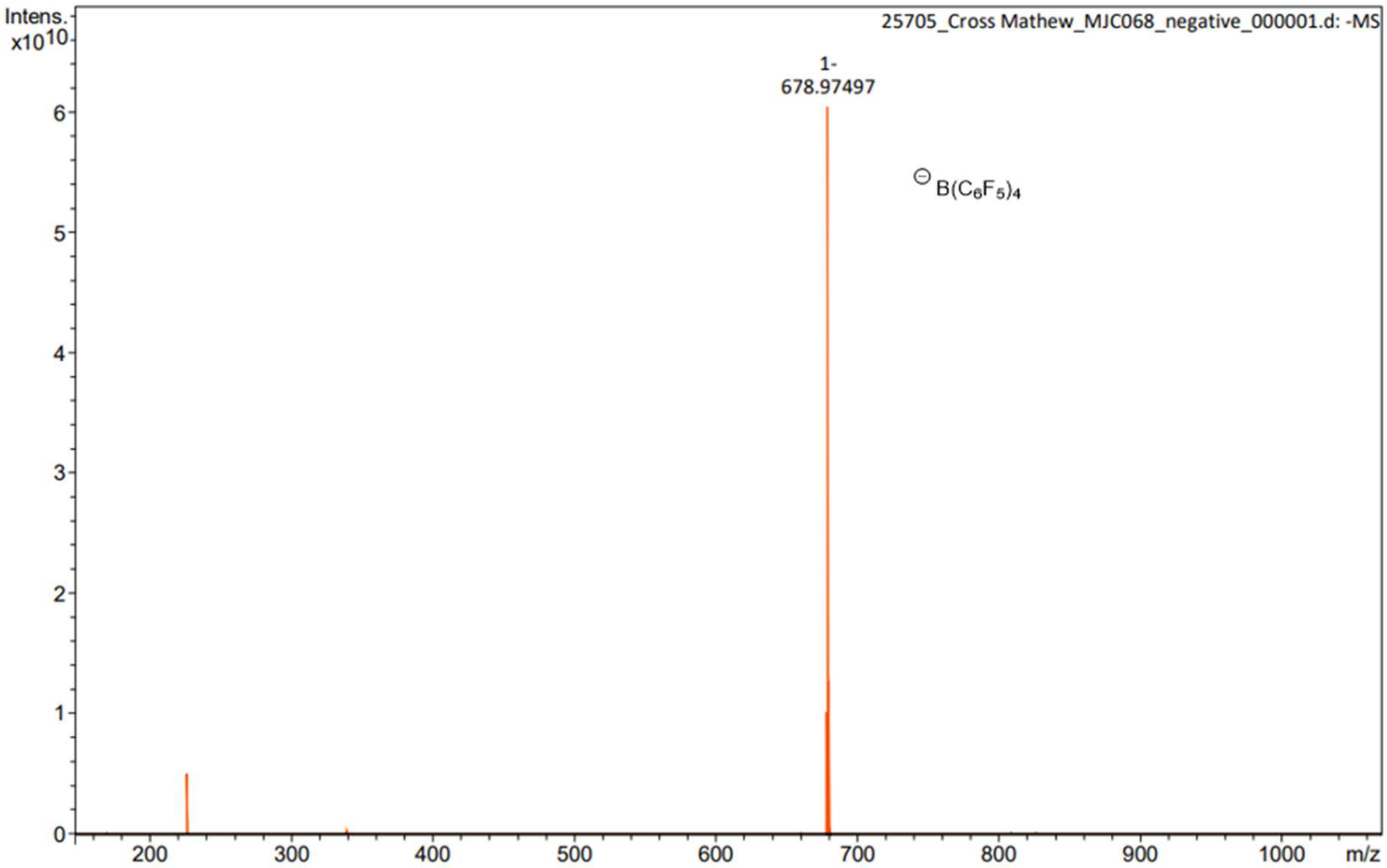

Figure S6. The MALDI- mass spectrum of **3a** showing the anion $[B(C_6F_5)_4]^-$ at *m/z* = 678.97 (predicted 678.98).

1.3.4 The in-situ NMR-scale synthesis of 3-BCF (DiKTa-BCF)

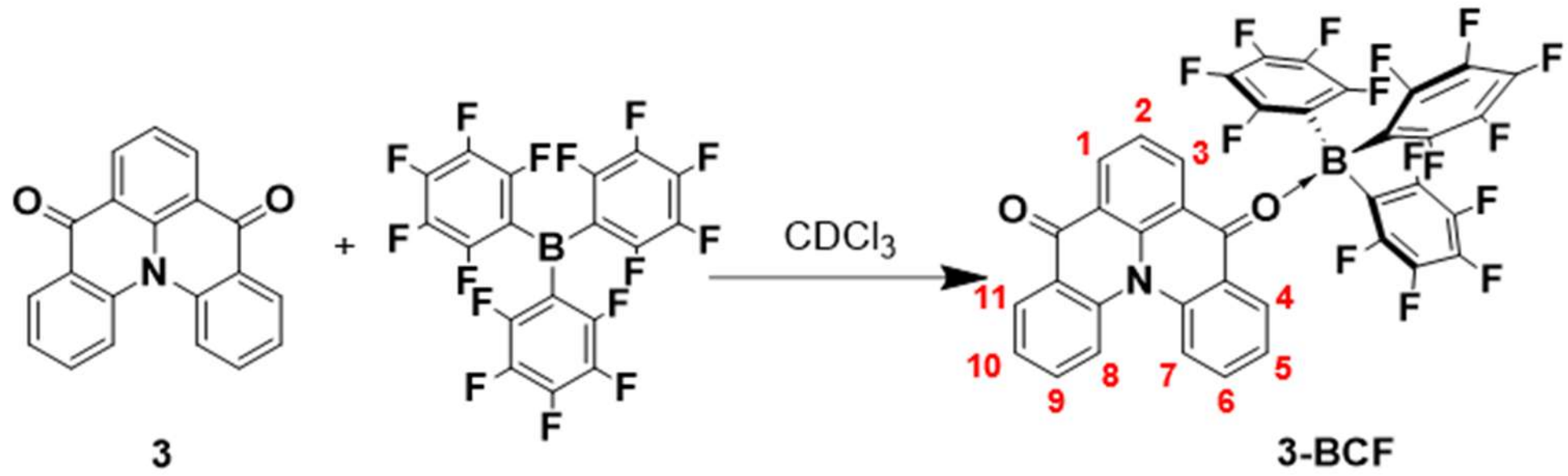

3 3-BCF

*Notice: Compound (**3-BCF**) is prone to rapid decomposition upon exposure to even small quantities of water in solution.*

Under an atmosphere of nitrogen, 0.010 g (0.03 mmol) of DiKTa (**3**) and 0.017 g (0.03 mmol) of BCF were dissolved in 0.5 cm^3 of $CDCl_3$ mixed for 16 hours at room temperature. This solution turned from yellow to orange as **3-BCF** formed. All NMR spectral analyses were performed in-situ.

^{1}H NMR (500 MHz, $CDCl_3$, 298 K): δ 8.87 (2H, d, 7.7 Hz, H-1 & H-3), 8.54 (2H, dd, 8.2 Hz, 1.5 Hz, H-4 & H-11), 8.23 (2H, d, 8.5 Hz, H-7 & H-8), 7.86 (2H, ddd, 8.7 Hz, 7.1 Hz, 1.6 Hz, H-6 & H-9), 7.74 (1H, t, 7.7 Hz, H-2), 7.62 (2H, ddd, 8.1 Hz, 7.3 Hz, 0.9 Hz, H-5 & H-10).

^{13}C NMR (125.7 MHz, $CDCl_3$, 298 K): δ 178.4 (C=O), 148.0 (dm, $^1J_{CF}$ = 242 Hz), 140.3 (dm, $^1J_{CF}$ = 251 Hz), 139.8, 139.0, 137.2 (dm, $^1J_{CF}$ = 248 Hz), 136.3, 135.0, 128.9, 126.9, 125.6, 124.7, 122.0, 121.2, 118.3.

^{1}H NMR (500 MHz, $CDCl_3$, 235 K): δ 8.96 (1H, d, 7.2 Hz, H-1), 8.89 (1H, d, 7.6 Hz, H-3), 8.63 (1H, d, 7.9 Hz, H-4), 8.50 (1H, d, 7.8 Hz, H-11), 8.45 (1H, d, 8.5 Hz, H-7), 8.17 (1H, d, 8.3 Hz H-8), 7.99 (1H, t, 7.5 Hz, H-6), 7.86 (1H, t, 7.4 Hz H-9), 7.79 (1H, t, 7.7 Hz, H-2), 7.69 (1H, t, 7.8 Hz, H-10), 7.65 (1H, t, 7.4 Hz H-5).

^{13}C NMR (125.7 MHz, $CDCl_3$, 235 K): δ 178.2 (C=O), 177.7 (C=O), 147.7 (dm, $^1J_{CF}$ = 242 Hz), 140.5, 139.7 (dm, $^1J_{CF}$ = 258 Hz), 138.7, 138.5, 138.0, 136.9 (dm, $^1J_{CF}$ = 249 Hz), 136.8, 135.7, 133.9, 133.4, 129.3, 128.4, 128.2, 126.9, 126.3, 124.8, 123.1, 122.9, 120.5, 119.7, 117.9.

^{19}F NMR (470.4 MHz, $CDCl_3$, 298 K): δ 134.0 (6F, d, 25.0 Hz, F-ortho), 156.6 (3F, t, 43.9 Hz, F-para), 163.5 (6F, dt, 23.0 Hz, 9.1 Hz, F-meta).

^{11}B NMR (160.4 MHz, $CDCl_3$, 298 K): δ 2.9 (B, br, DiKTa-BCF).

1.3.5 The synthesis of 4a (QPO-BtripS [$B(C_6F_5)_4$])

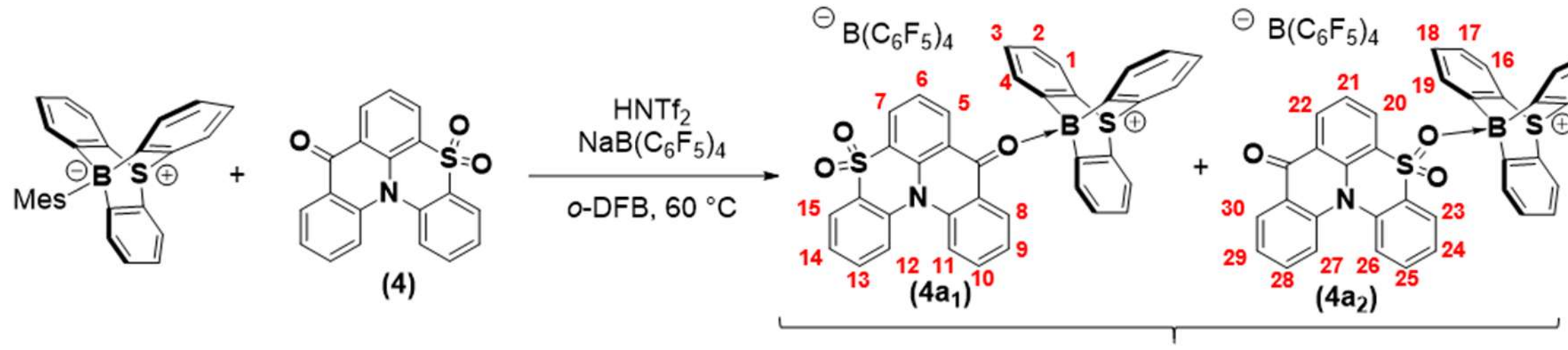

(4a)

*Notice: Compound (**4a**) in solution is prone to decomposition upon exposure to sources of UV light.*[10]

Under an atmosphere of nitrogen, 0.085 g (0.29 mmol) of QPO (**4**), 0.094 g (0.24 mmol) of Mes-BtripS, 0.067 g (0.24 mmol) of $HNTf_2$ and 0.168 g (0.24 mmol) of $NaB(C_6F_5)_4$ were added to an ampoule and then suspended in 2 cm^3 of *ortho*-difluorobenzene with stirring. This reaction mixture was heated to 60 °C for 16 hours, during which the colour changed from yellow to dark yellow, before cooling to room temperature. After cooling, the solvent was removed *in-vacuo* and the solids were purified by column chromatography on silica with an eluent of EtOAc/pet ether, where the solvent ratio was varied from 30% to 70% EtOAc. The fractions containing the yellow product (R_f = ~ 0.1 in 1:1 EtOAc:pet ether) were collected and the solvent removed *in-vacuo*, leaving a yellow solid. The yellow solid was dissolved in Et_2O, triturated with pentane and then washed with pentane. The resulting solid was dried under a high-vacuum (10^{-3} mbar) to give **4a** as a yellow powder (0.082 g, 25% yield). **4a** was determined to be a mixture of isomers (**4a₁** & **4a₂**) by ^{1}H and ^{13}C NMR spectroscopy, which were inseparable by column chromatography or extraction.

The sulfone-bound isomer **4a₂** was crystallised from slow evaporation of a mixture of both isomers in $CDCl_3$. **4a₁** crystals were not recovered from this crystallisation.

^{1}H NMR (500 MHz, THF-d_8, 298 K): δ 9.16 (1H, dd, 7.9 Hz, 1.5 Hz, H-20), 9.03 (1H, dd, 8.6 Hz, 1.2 Hz, H-8), 8.97 (1H, dd, 8.5 Hz, 1.4 Hz, H-5), 8.95 (1H, dd, 7.8 Hz, 1.5 Hz, H-22), 8.93 (1H, dd, 8.1 Hz, 1.5 Hz, H-23), 8.85 (1H, dd, 7.5 Hz, 1.4 Hz, H-7), 8.69 (1H, d, 8.7 Hz, H-11), 8.53 (1H, dd, 8.0 Hz, 1.6 Hz, H-30), 8.43 (1H, m, H-15), 8.36 (1H, d, 8.7 Hz, H-26), 8.30 (1H, d, 8.5 Hz, H-27), 8.23 (1H, ddd, 8.7 Hz, 6.9 Hz, 1.4 Hz, H-10), 8.12 – 8.05 (6H, overlapping m, H-12, H-1, H-21 & H-25), 7.98 – 7.93 (2H, overlapping m, H-24 & H-28), 7.90 (3H, dd, 7.4 Hz, 1.2 Hz, H-16), 7.89 – 7.85 (5H, overlapping m, H-13, H-14, H-4), 7.77 – 7.67 (3H, overlapping m, H-9, H-29, H-6), 7.61 (3H, dd, 6.8 Hz, 1.6 Hz, H-19), 7.29 (6H, overlapping m, H-2 & H-3), 7.19 (6H, overlapping m, H-17 & H-18).

^{13}C NMR (125.7 MHz, THF-d_8, 298 K): δ 180.2, 177.2, 153.2, 149.3 (d, $^1J_{CF}$ = 245 Hz), 143.5, 142.4, 140.8, 140.7, 140.3, 139.2 (d, $^1J_{CF}$ = 250 Hz),139.1, 137.8, 137.3 (d, $^1J_{CF}$ = 249 Hz), 136.6, 135.7, 135.3, 134.6, 134.5, 134.2, 133.8, 135.5, 132.8, 131.8, 131.7, 130.3, 130.1, 129.9, 129.8, 129.7, 129.0, 128.7, 128.2, 128.1 128.0, 127.9, 127.1, 127.0, 126.9, 126.5, 126.3, 126.1, 125.6, 125.2, 125.0, 124.9, 124.7, 124.5, 123.2, 122.9, 122.6, 122.4, 122.2, 121.4. $[B(C_6F_5)_4]^-$ C–B not observed.

^{19}F NMR (470.4 MHz, THF-d_8, 298 K): δ – 132.7 (dd, 8F, 16 Hz, 7.0 Hz, $[B(C_6F_5)_4]^-$), –165.1 (td, 4F, 21.5 Hz, 5.7 Hz, $[B(C_6F_5)_4]^-$), – 168.6 (t, 8F, 21.5 Hz, $[B(C_6F_5)_4]^-$).

^{11}B NMR (160.4 MHz, THF-d_8, 298 K): δ 8.7 (1B, s, BtripS), 5.2 (1B, s, BtripS), – 16.6 (1B, s, $[B(C_6F_5)_4]^-$).

^{10}B NMR (53.7 MHz, THF-d_8, 298 K): δ 6.6 (2B, br, BtripS), –16.6 (1B, s, $[B(C_6F_5)_4]^-$).

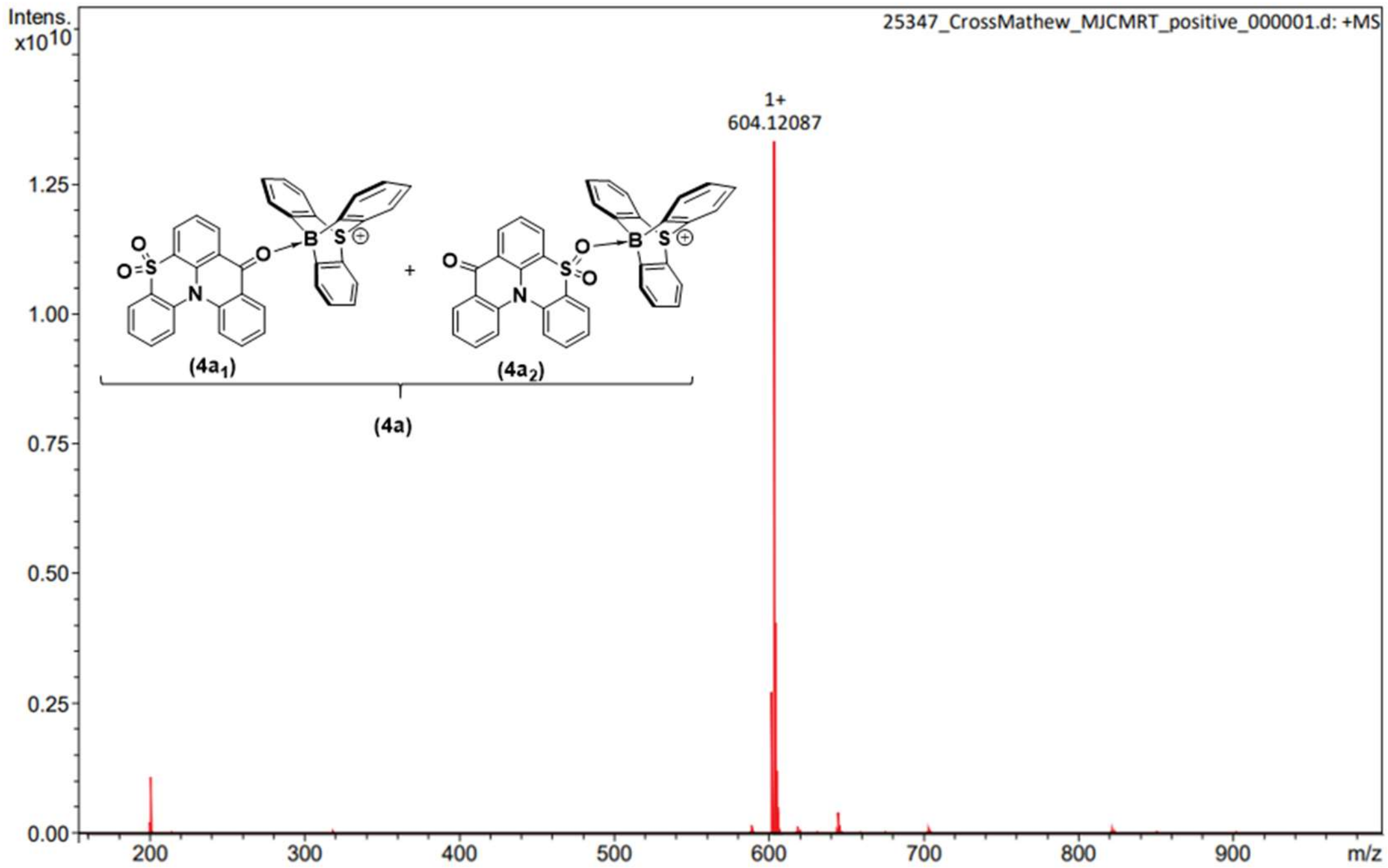

Figure S7. The ESI+ mass spectrum of **4a** showing the molecular ion $[M]^+$ at *m/z* = 604.12 (predicted 604.12). Recorded in MeOH.

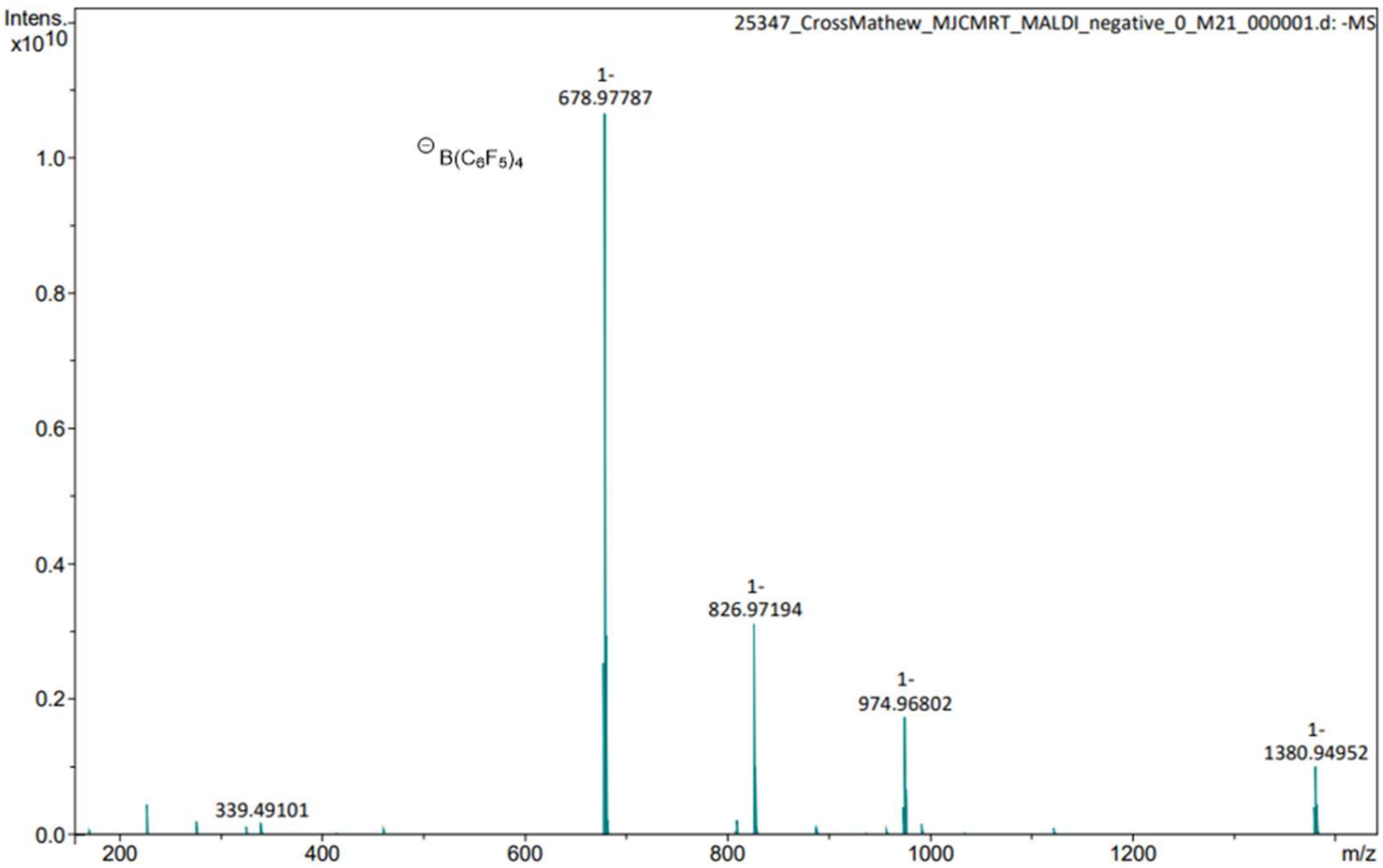

Figure S8. The MALDI- mass spectrum of **4a** showing the anion $[B(C_6F_5)_4]^-$ at *m/z* = 678.98 (predicted 678.98).

1.4 NMR spectra

1.4.1 NMR spectra of 1a (DPA-DPS-BtripS [B(C_6F_5)$_4$])

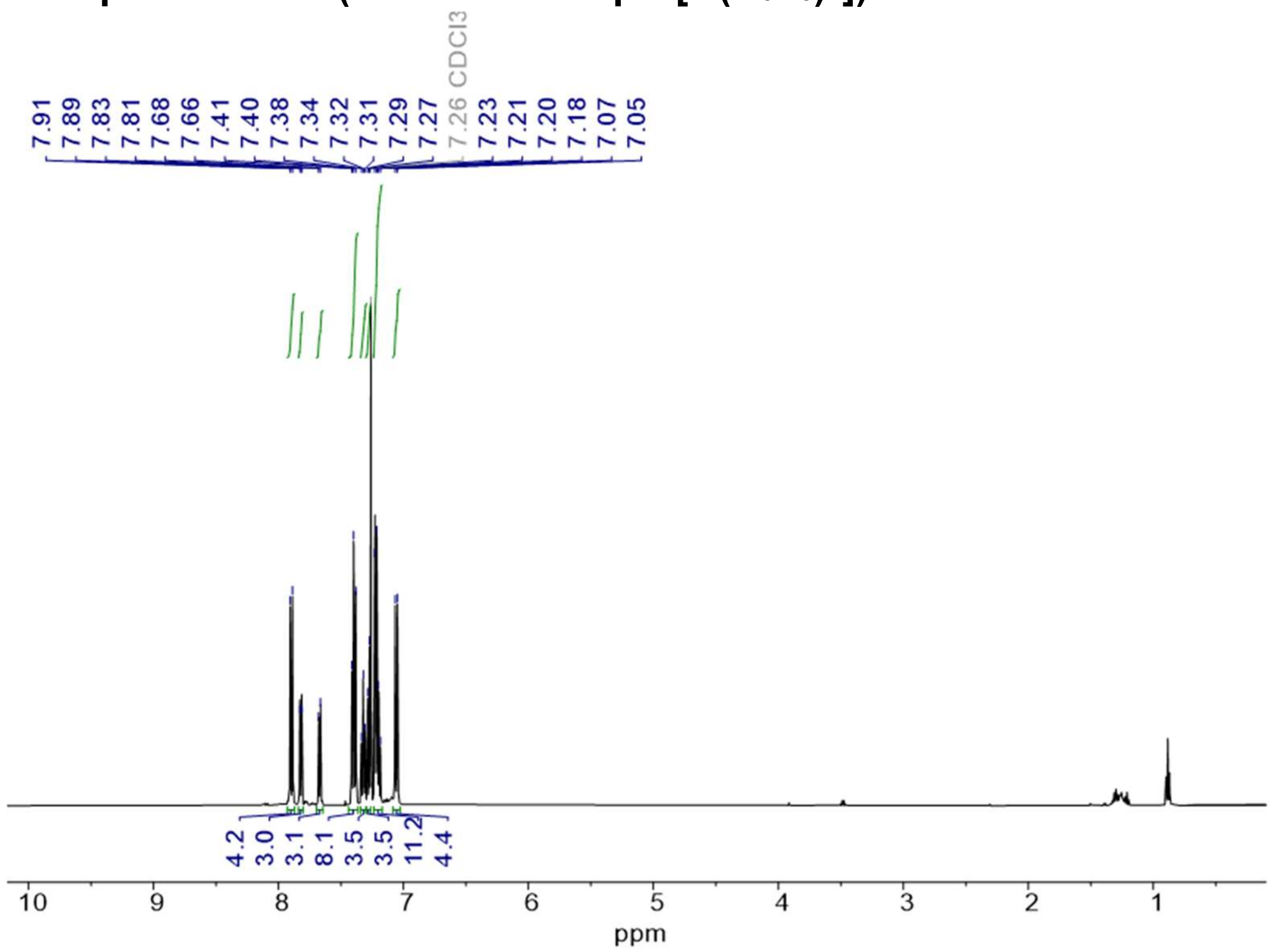

Figure S9. The ^{1}H NMR spectrum of **1a** (DPA-DPS-BtripS [B(C_6F_5)$_4$]) showing traces of entrained alkanes (~1 ppm). Recorded in $CDCl_3$ at 298 K.

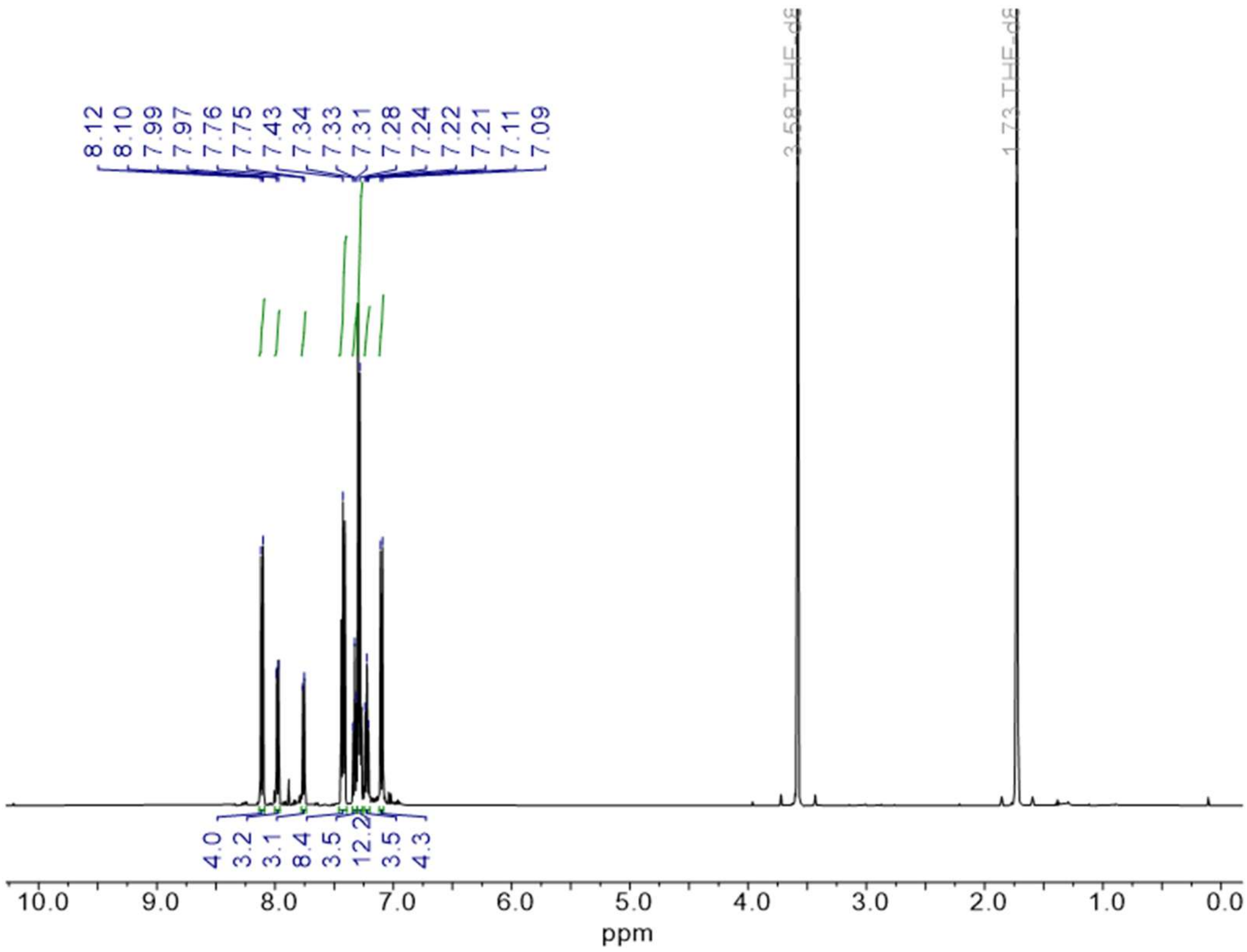

Figure S10. The ^{1}H NMR spectrum of residual-solvent free **1a** (DPA-DPS-BtripS [B(C_6F_5)$_4$]) used for photophysical measurements. Recorded in THF-d_8 at 298 K.

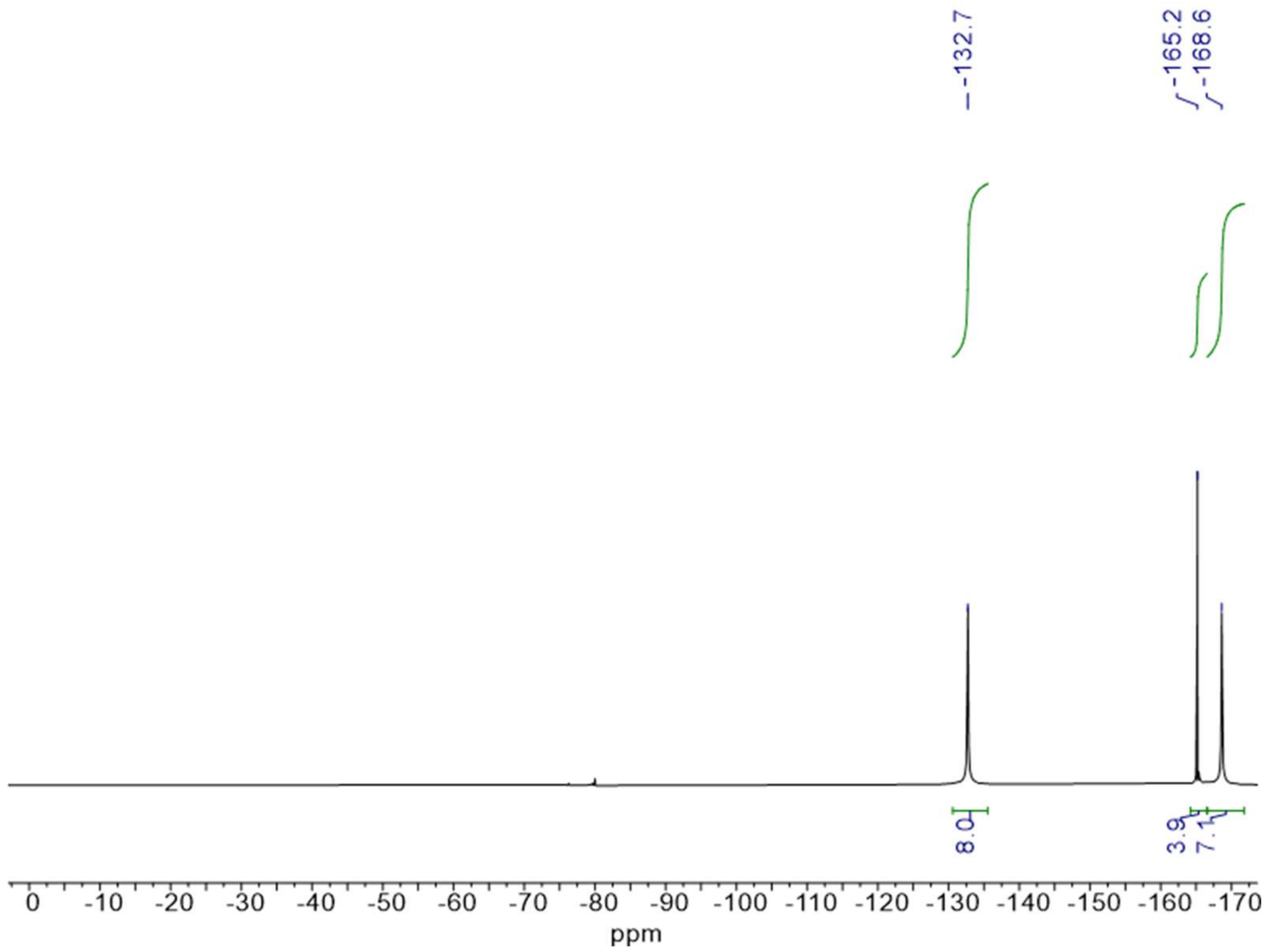

Figure S11. The ^{19}F NMR spectrum of **1a** (DPA-DPS-BtripS $[B(C_6F_5)_4]$) showing signals corresponding to the $[B(C_6F_5)_4]^-$ anion. Recorded in THF-d_8 at 298 K.

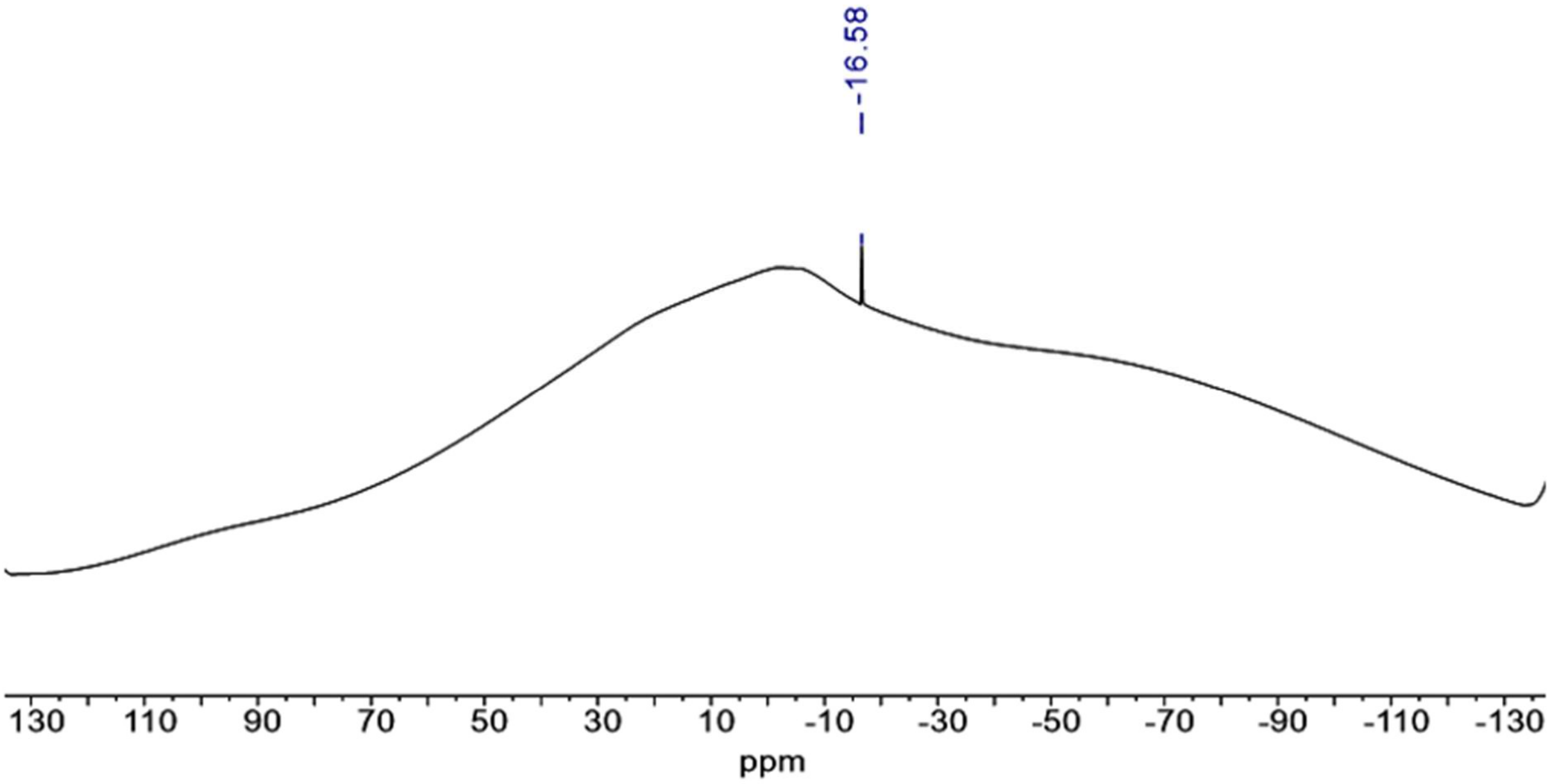

Figure S12. The ^{11}B NMR spectrum of **1a** (DPA-DPS BtripS $[B(C_6F_5)_4]$) showing a signal corresponding to the $[B(C_6F_5)_4]^-$ anion. Recorded in THF-d_8 at 298 K.

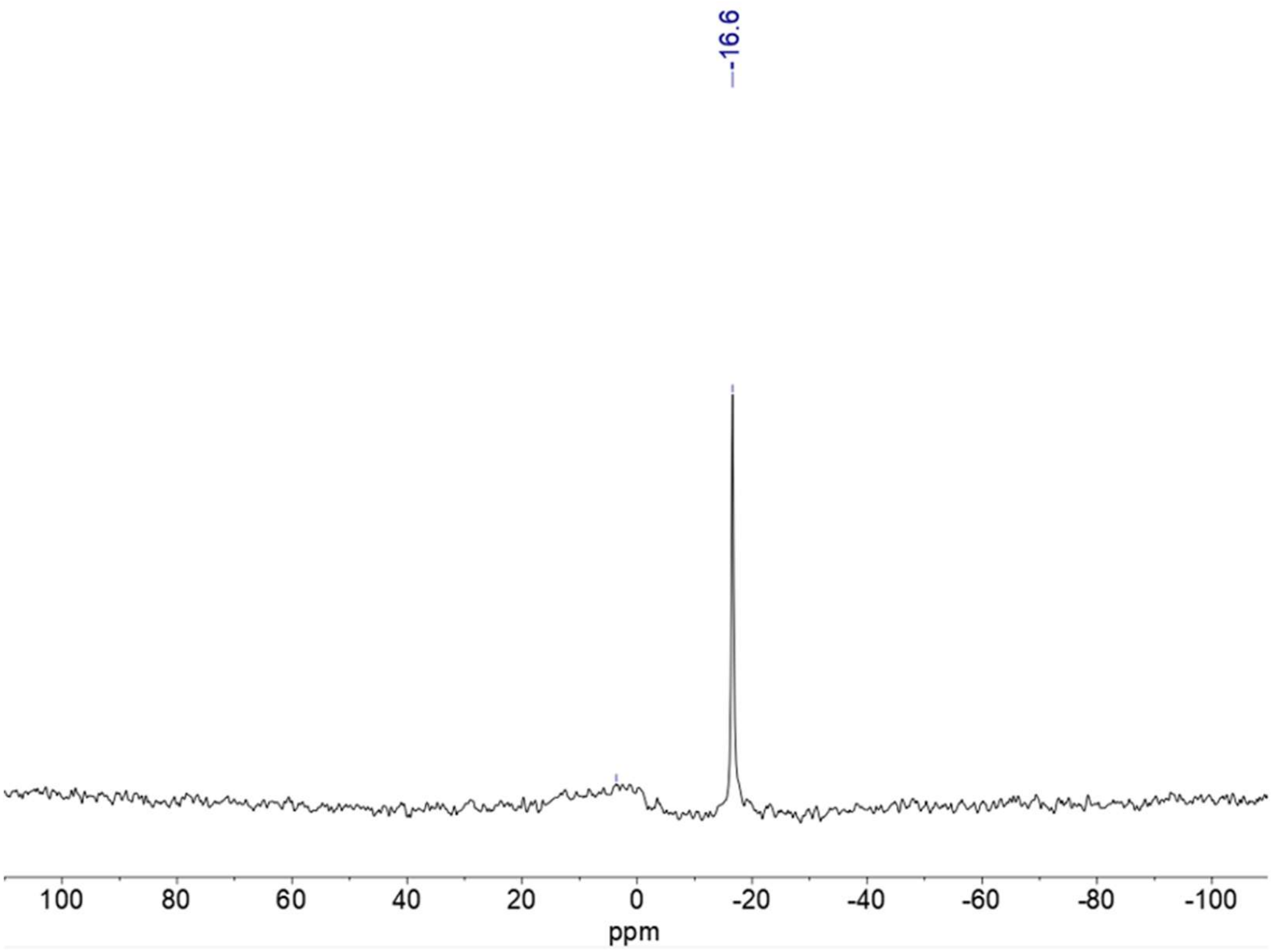

Figure S13. The ^{10}B NMR spectrum of **1a** (DPA-DPS-BtripS $[B(C_6F_5)_4]$) showing a sharp signal corresponding to the $[B(C_6F_5)_4]^-$ anion (– 16.6 ppm). Recorded in THF-d_8 at 298 K.

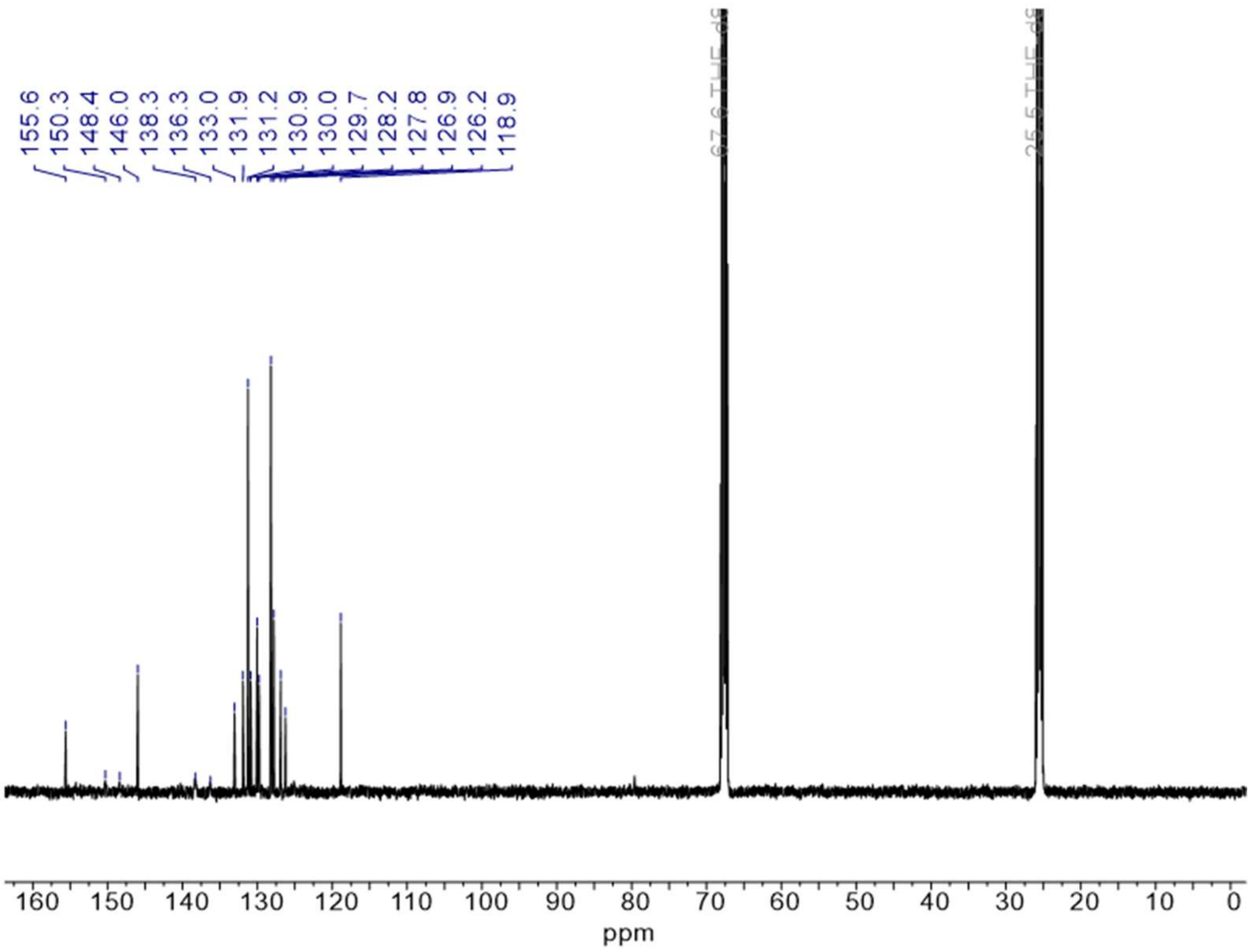

Figure S14. The ^{13}C NMR spectrum of **1a** (DPA-DPS-BtripS $[B(C_6F_5)_4]$) recorded in THF-d_8 at 298 K.

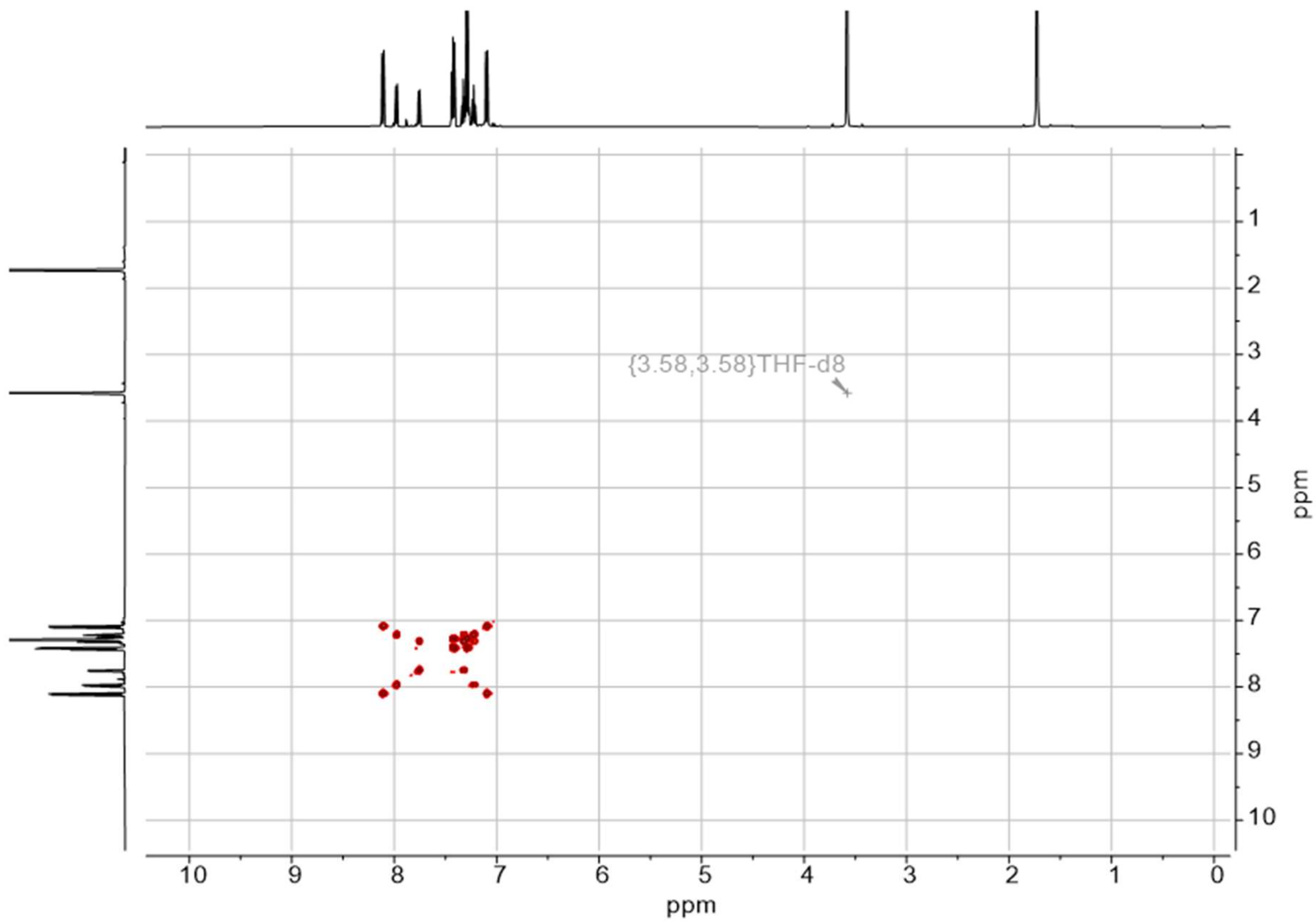

Figure S15. $^{1}H^{1}H$ COSY NMR spectrum of **1a** (DPA-DPS-BtripS $[B(C_6F_5)_4]$) recorded in THF-d_8 at 298 K.

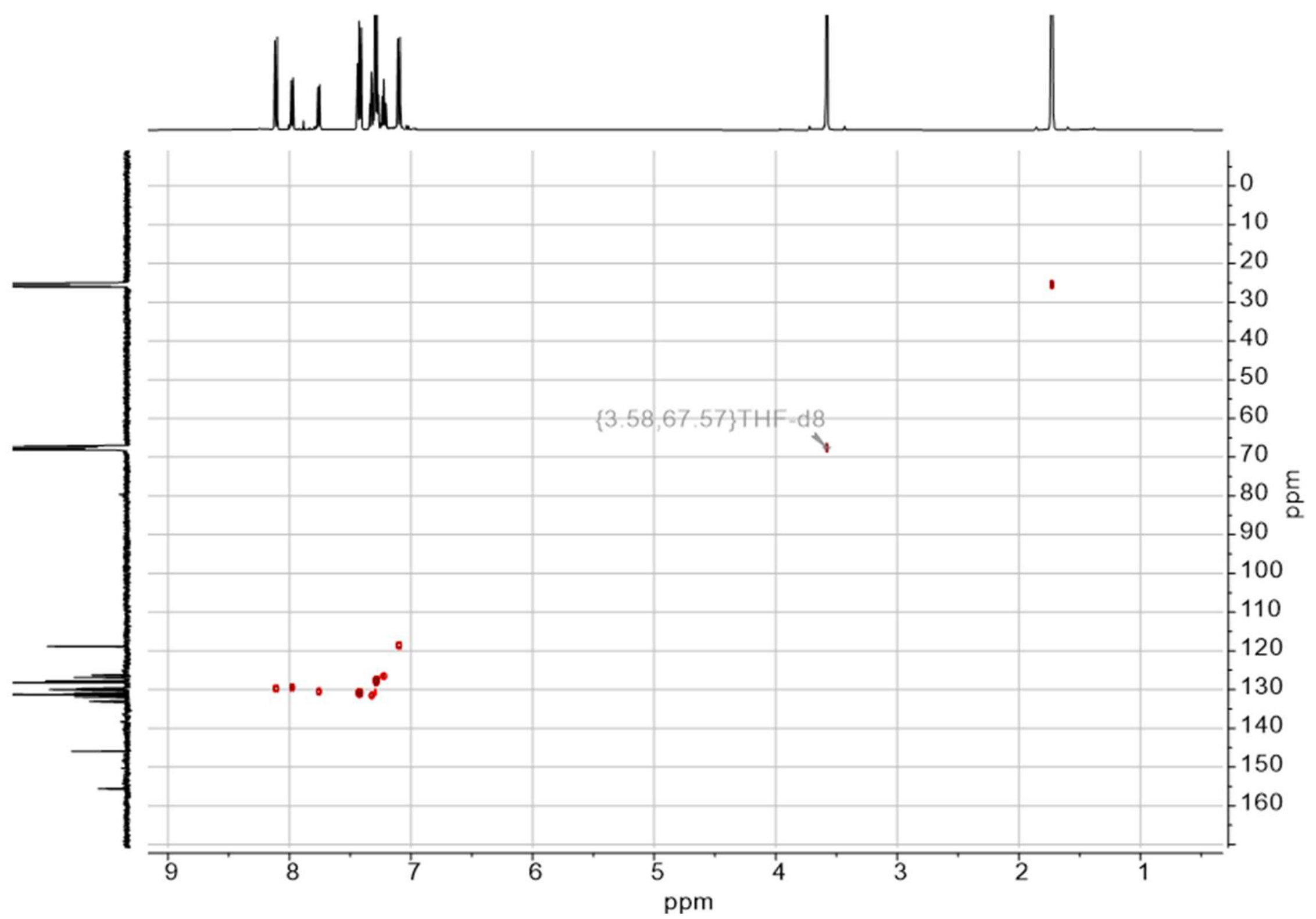

Figure S16. $^{1}H^{13}C$ HSQC NMR spectrum of **1a** (DPA-DPS-BtripS $[B(C_6F_5)_4]$) recorded in THF-d_8 at 298 K.

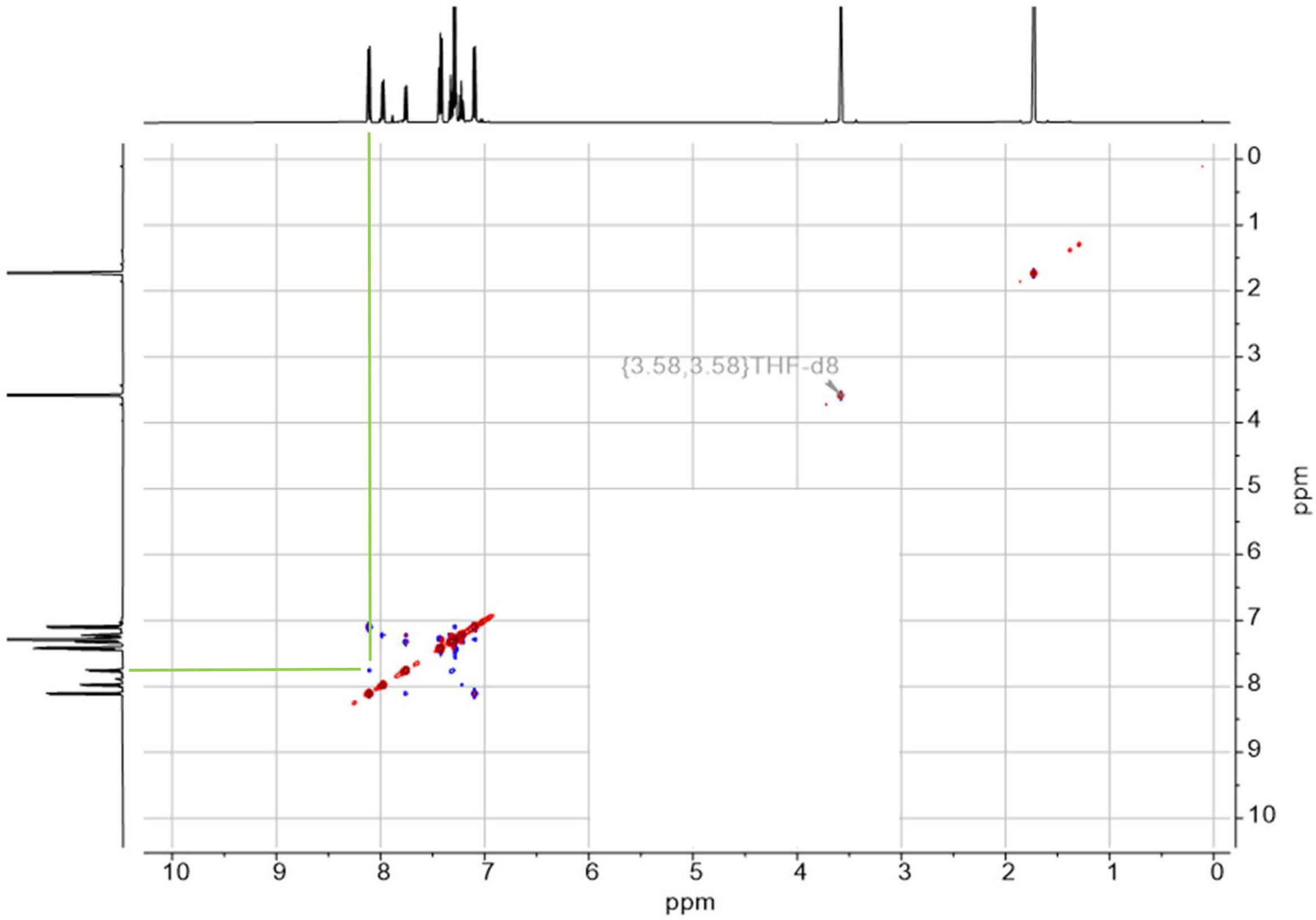

Figure S17. ^{1}H[1]H NOESY NMR spectrum of **1a** (DPA-DPS BtripS [$B(C_6F_5)_4$]) recorded in THF-d_8 at 298 K. A labelled NOE interaction (green lines) between all 3 proximal BtripS protons (highlighted in blue) and the 4 adjacent DPS phenyl protons.

1.4.2 NMR spectra of 2a (DMAC-DPS-BtripS $[B(C_6F_5)_4]$)

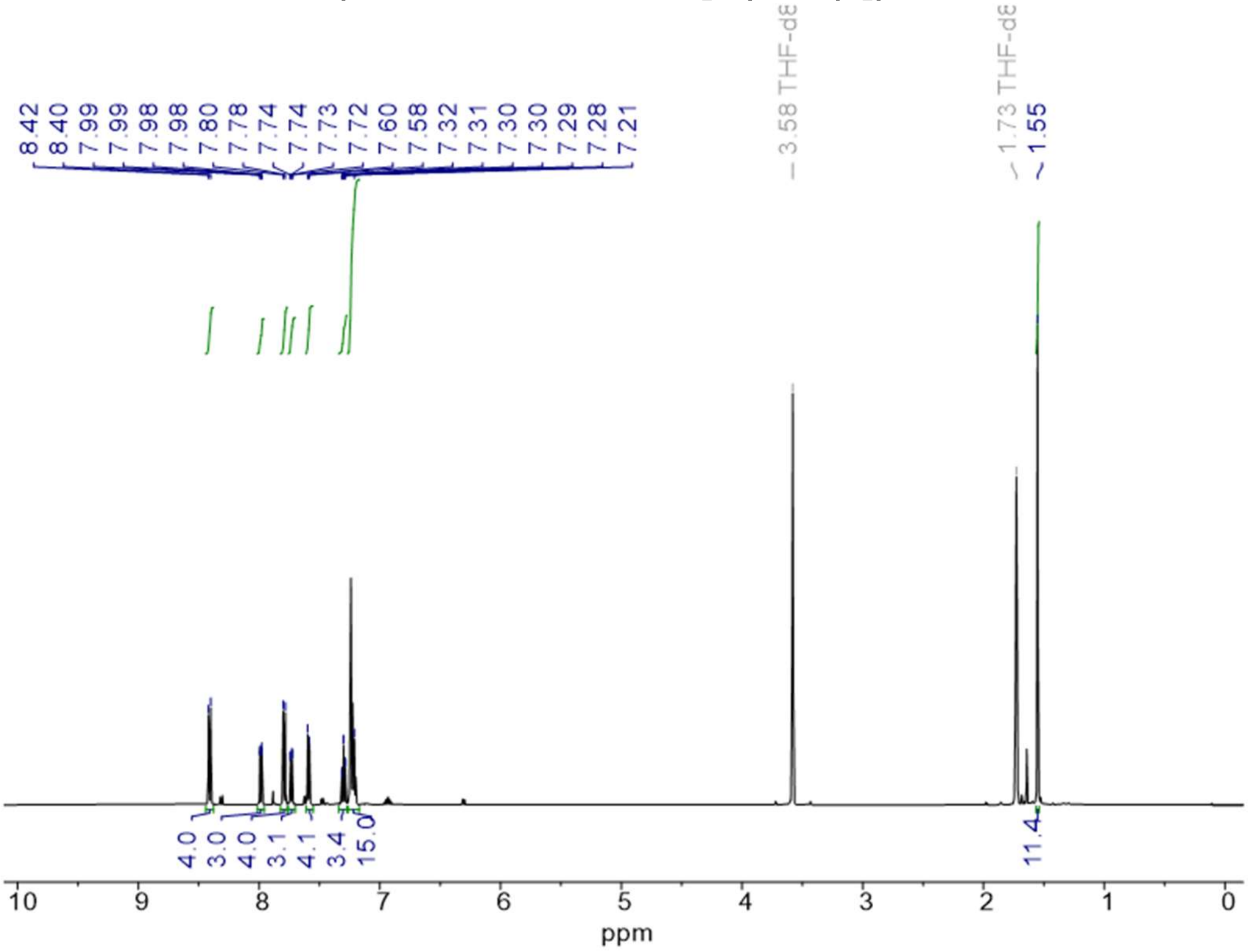

Figure S18. The ^{1}H NMR spectrum of **2a** (DMAC-DPS BtripS $[B(C_6F_5)_4]$) showing a trace impurity of **2** (DMAC-DPS) in the aromatic region. Recorded in THF-d_8 at 298 K.

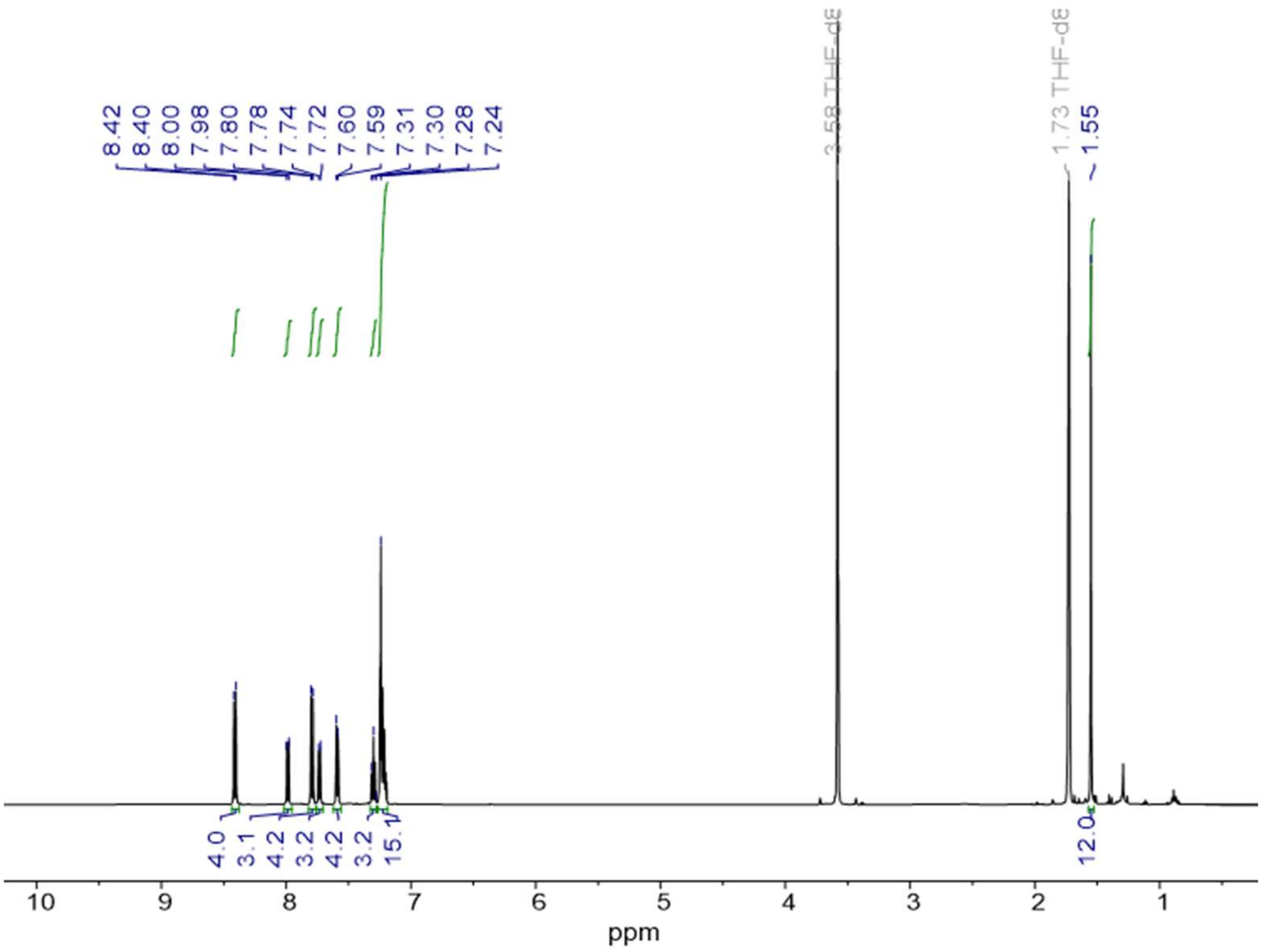

Figure S19. The ^{1}H NMR spectrum of residual-solvent free **2a** (DMAC-DPS-BtripS $[B(C_6F_5)_4]$) used for photophysical measurements. Recorded in THF-d_8 at 298 K.

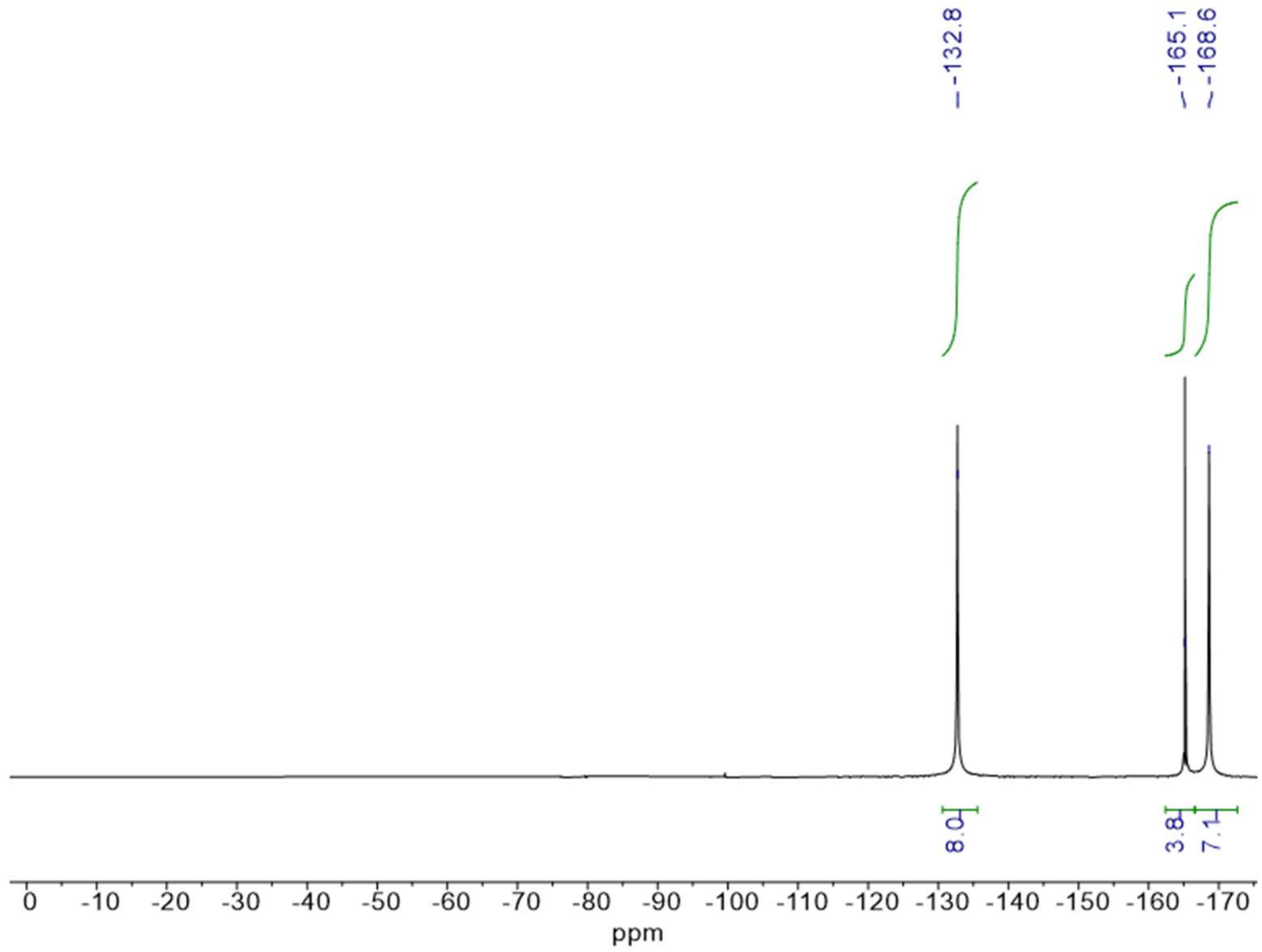

Figure S20. The ^{19}F NMR spectrum of **2a** (DMAC-DPS-BtripS $[B(C_6F_5)_4]$) showing signals corresponding to the $[B(C_6F_5)_4]^-$ anion. Recorded in THF-d_8 at 298 K.

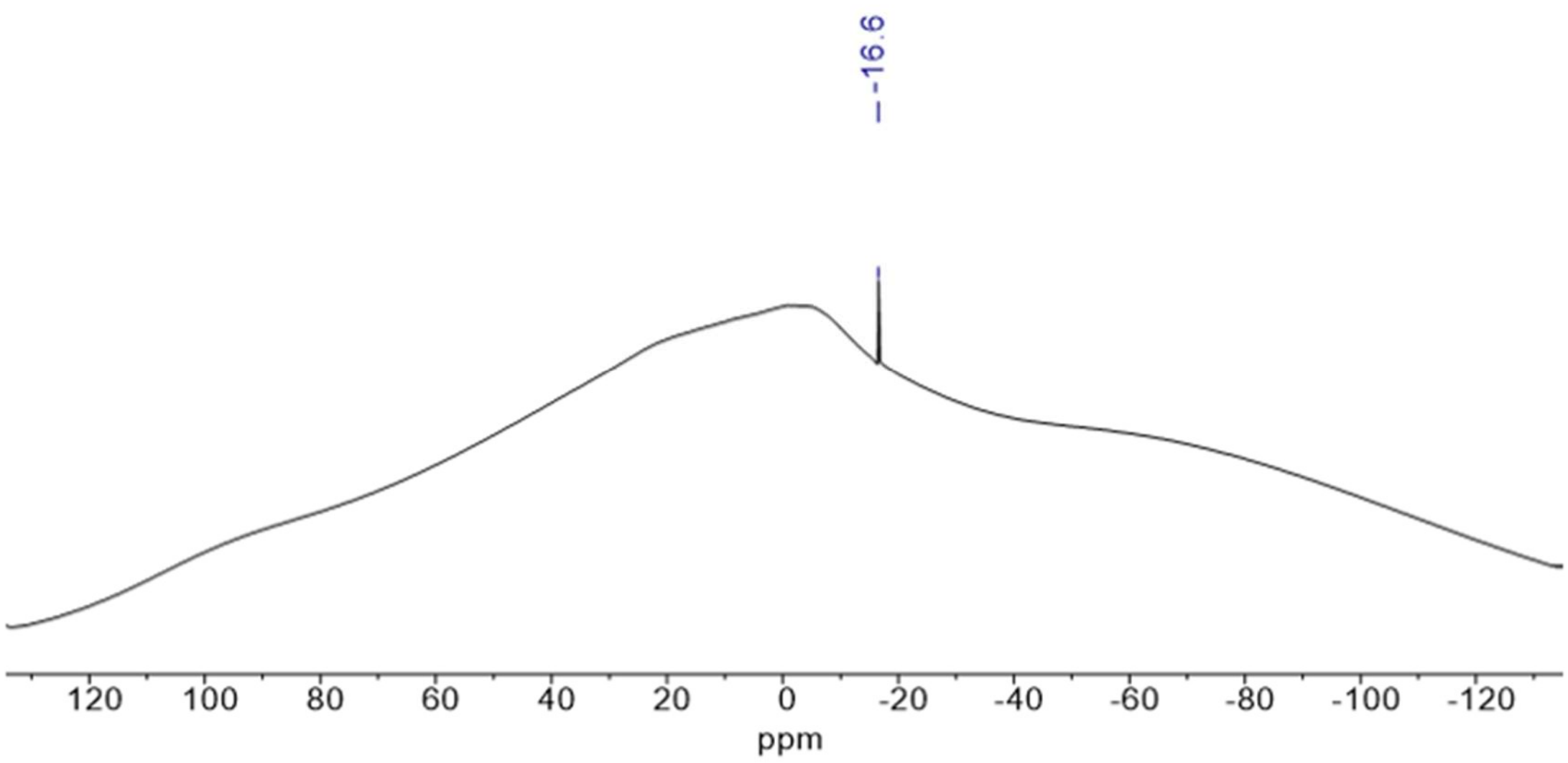

Figure S21. The ^{11}B NMR spectrum of **2a** (DMAC-DPS-BtripS $[B(C_6F_5)_4]$) showing a signal corresponding to the $B(C_6F_5)_4^-$ anion.

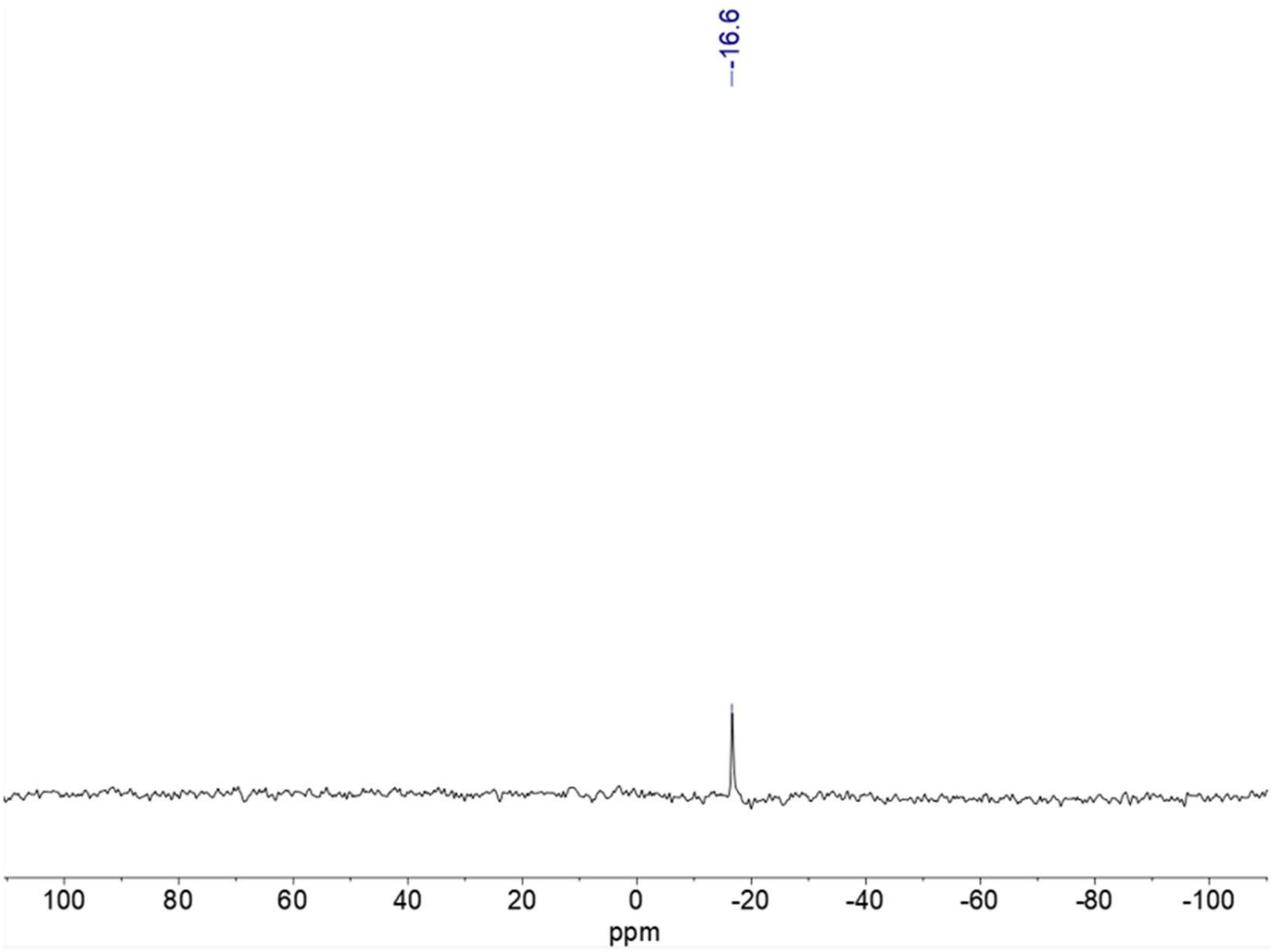

Figure S22. The [10]B NMR spectrum of **2a** (DMAC-DPS-BtripS $[B(C_6F_5)_4]$) showing a signal corresponding to the $[B(C_6F_5)_4]^-$ anion.

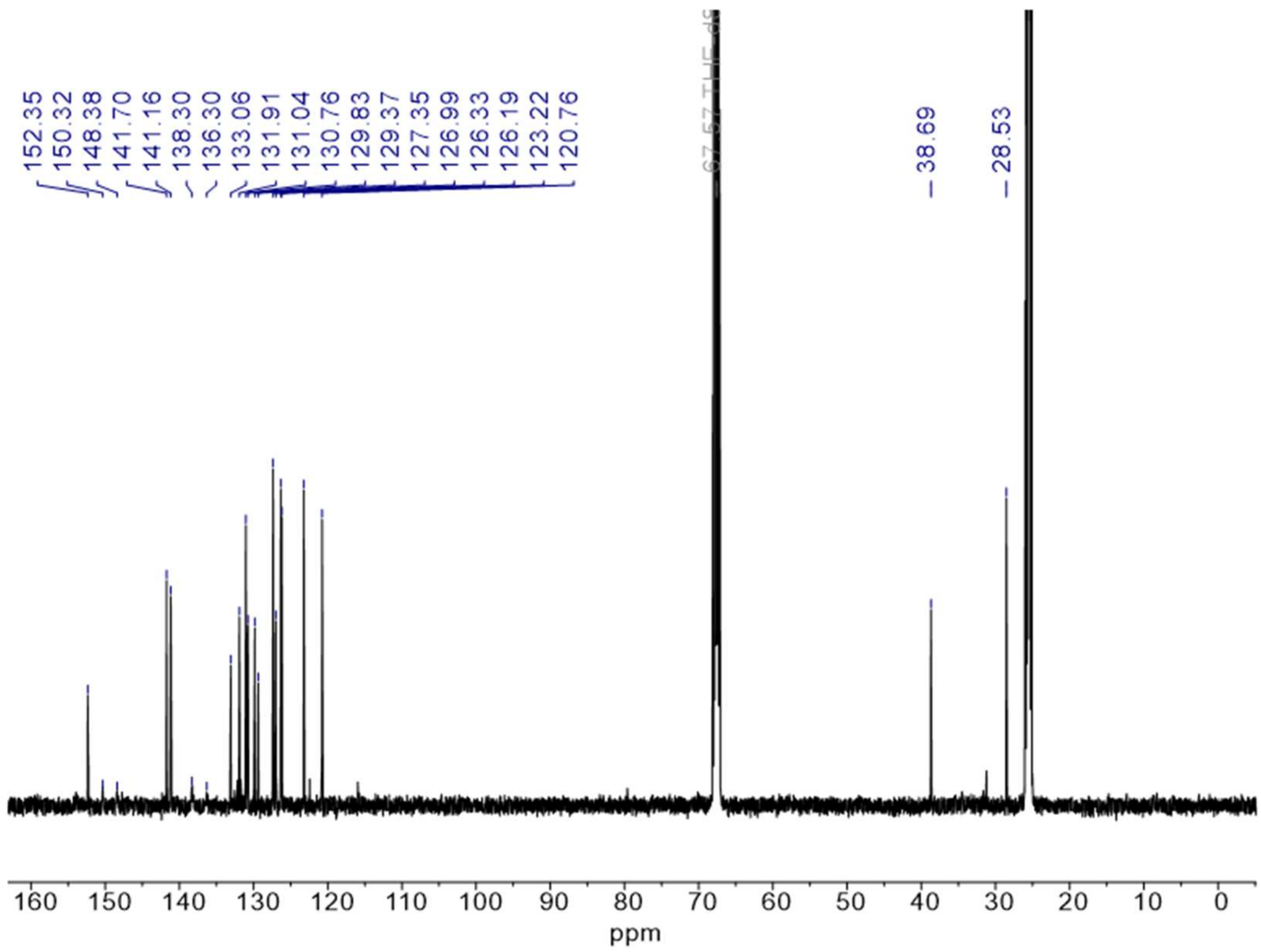

Figure S23. The ^{13}C NMR spectrum of **2a** (DMAC-DPS-BtripS $[B(C_6F_5)_4]$) recorded in THF-d_8 at 298 K.

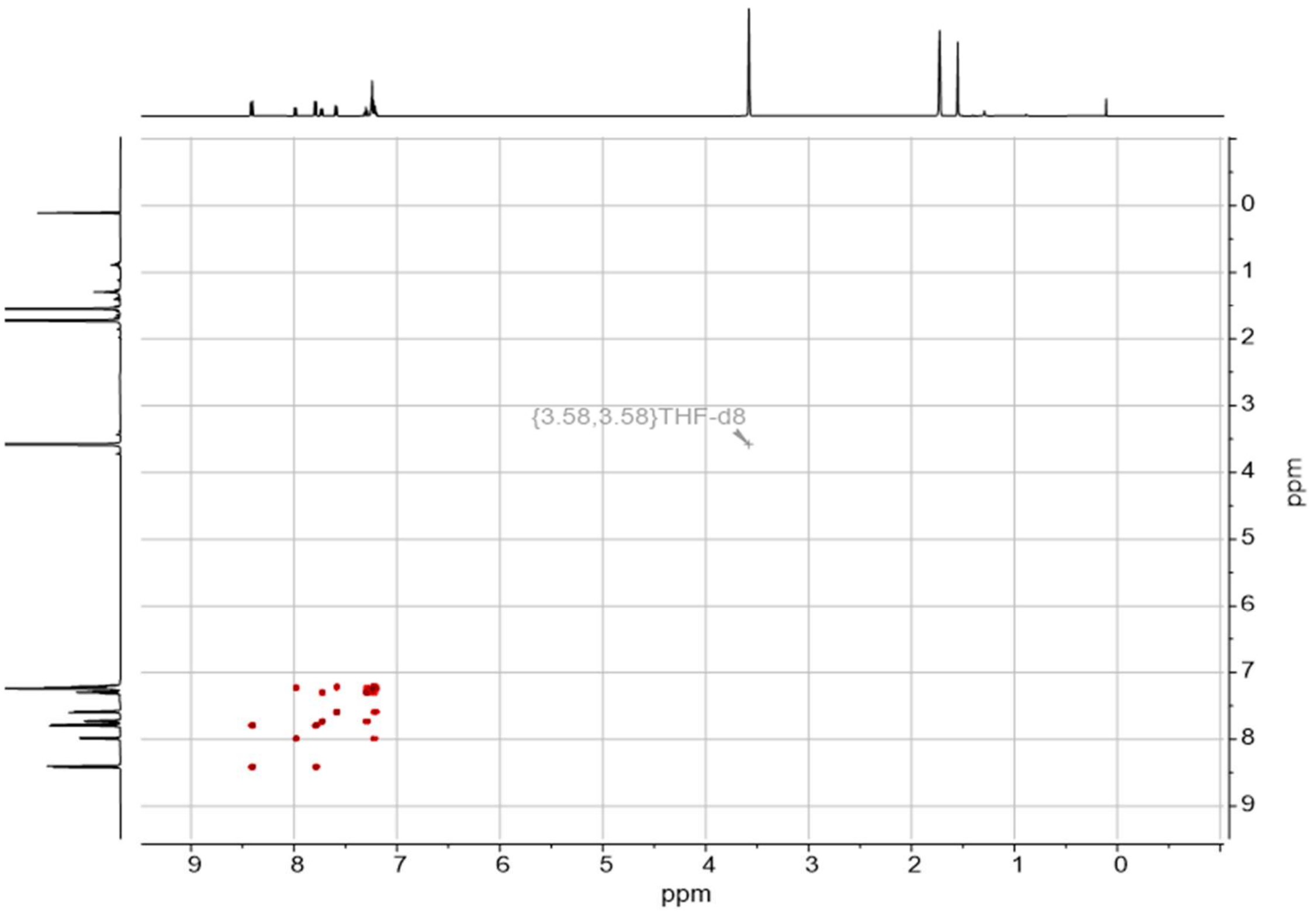

Figure S24. $^{1}H^{1}H$ COSY NMR spectrum of **2a** (DMAC-DPS-BtripS $[B(C_6F_5)_4]$) recorded in THF-d_8 at 298 K.

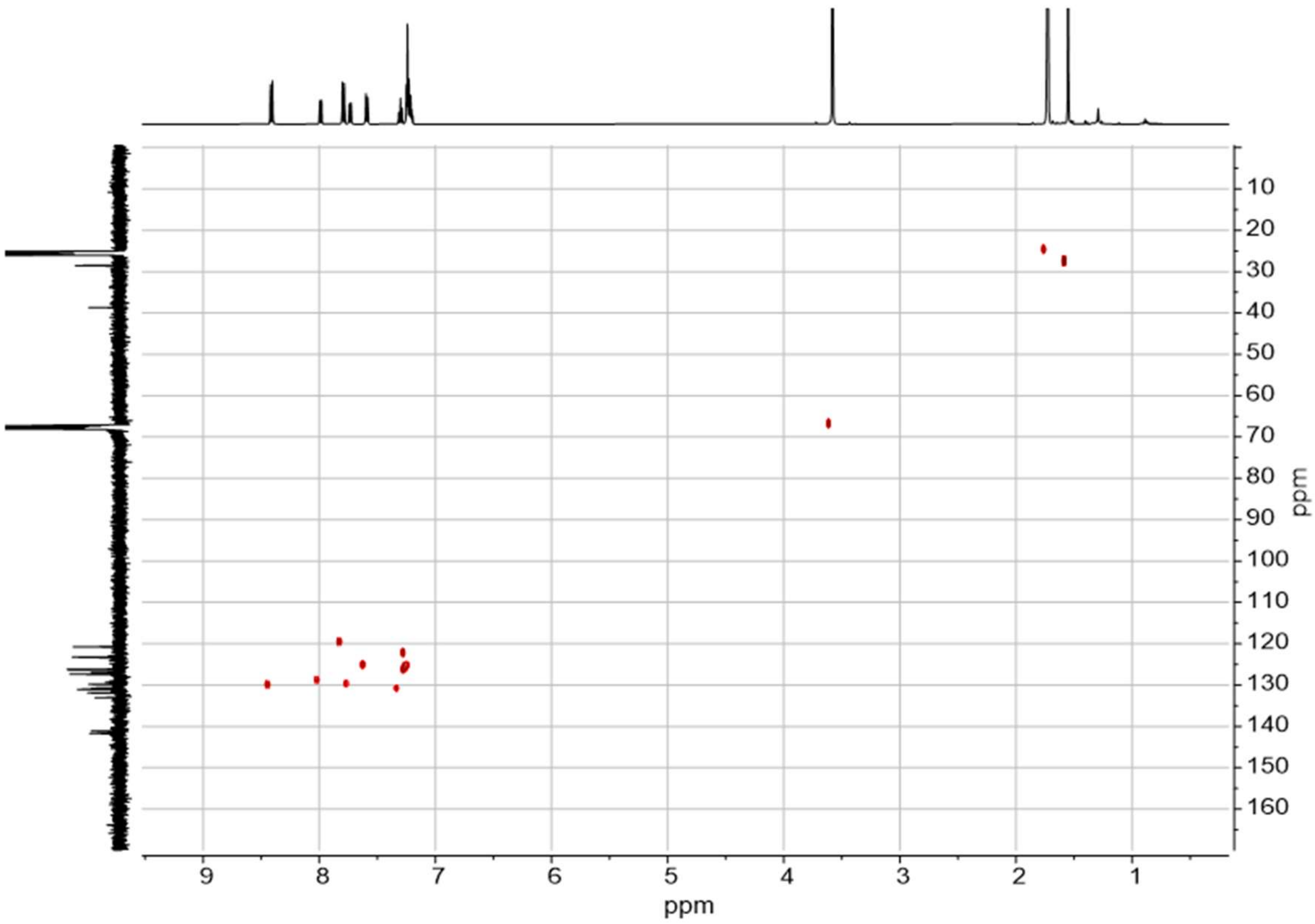

Figure S25. $^{1}H^{13}C$ HSQC NMR spectrum of **2a** (DMAC-DPS-BtripS $[B(C_6F_5)_4]$) recorded in THF-d_8 at 298 K.

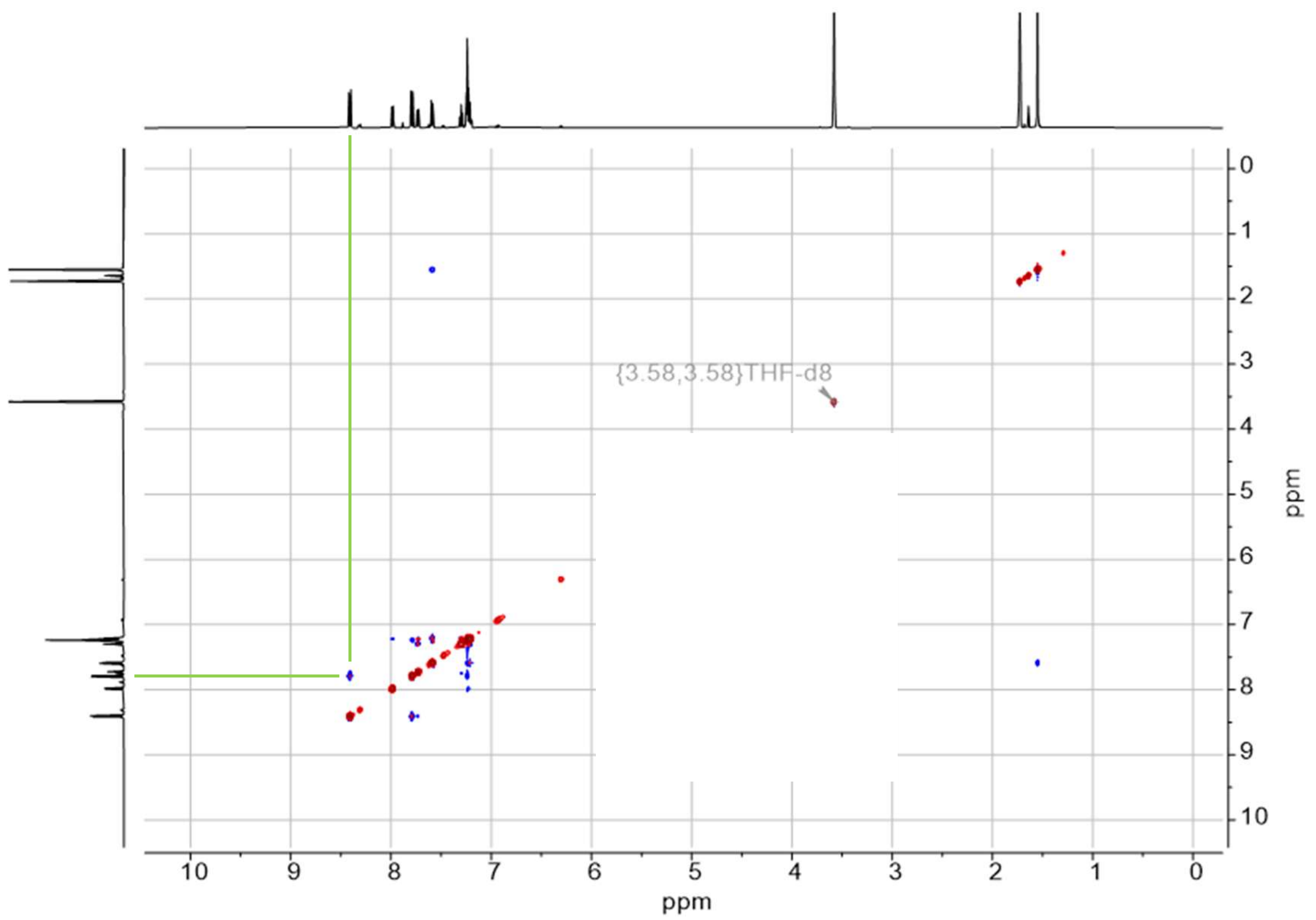

Figure S26. $^1H^1H$ NOESY NMR spectrum of **2a** (DMAC-DPS-BtripS $[B(C_6F_5)_4]$) recorded in THF-d_8 at 298 K. A labelled NOE interaction (green lines) between all 3 proximal BtripS protons (highlighted in blue) and the 4 adjacent DPS phenyl protons.

1.4.3 NMR spectra of 3a (DiKTa-BtripS [B(C_6F_5)$_4$])

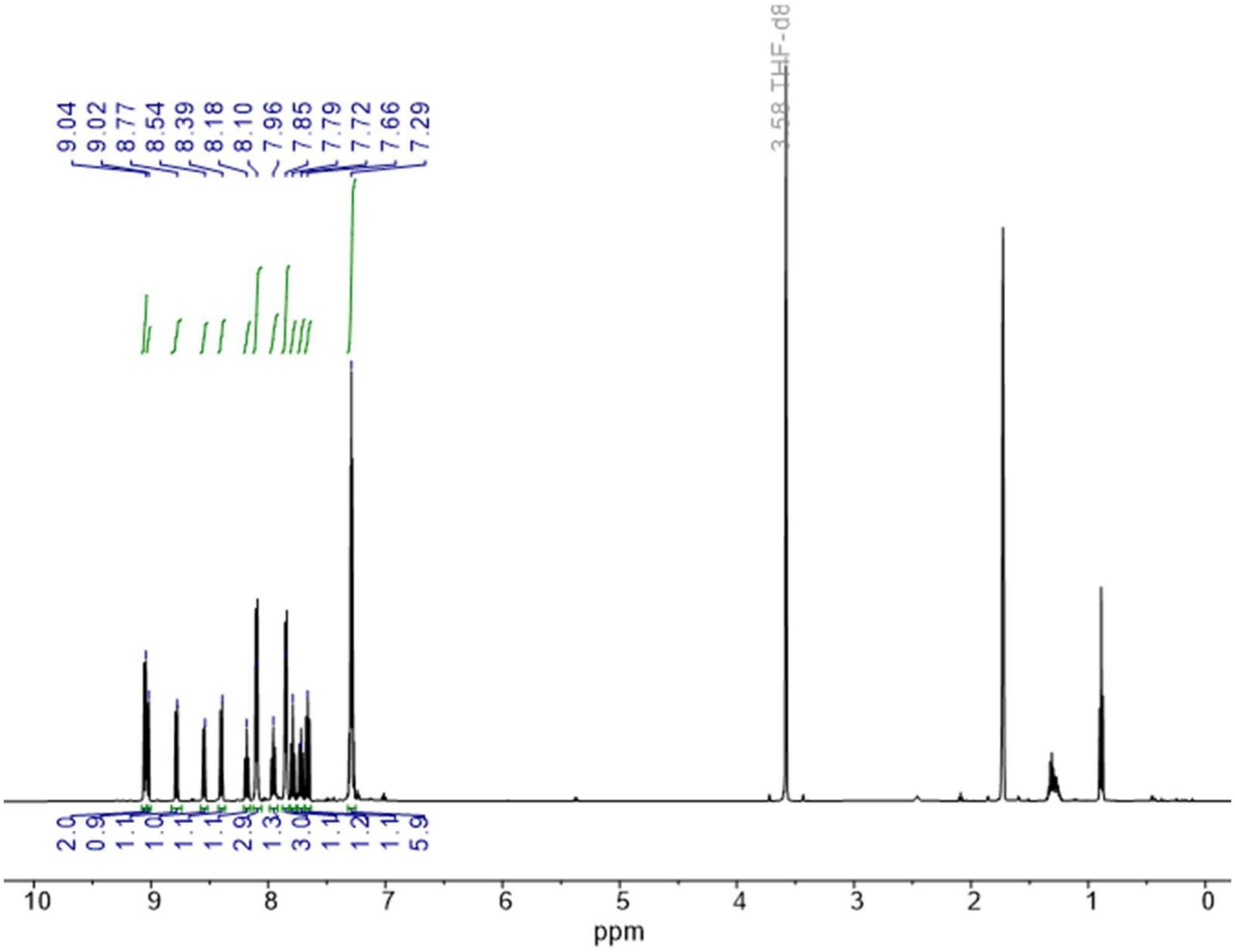

Figure S27. ^{1}H NMR spectrum of **3a** (DiKTa-BtripS [B(C_6F_5)$_4$]) containing residual pentane from workup (0.5 - 1.5 ppm). Recorded in THF-d_8 at 298 K.

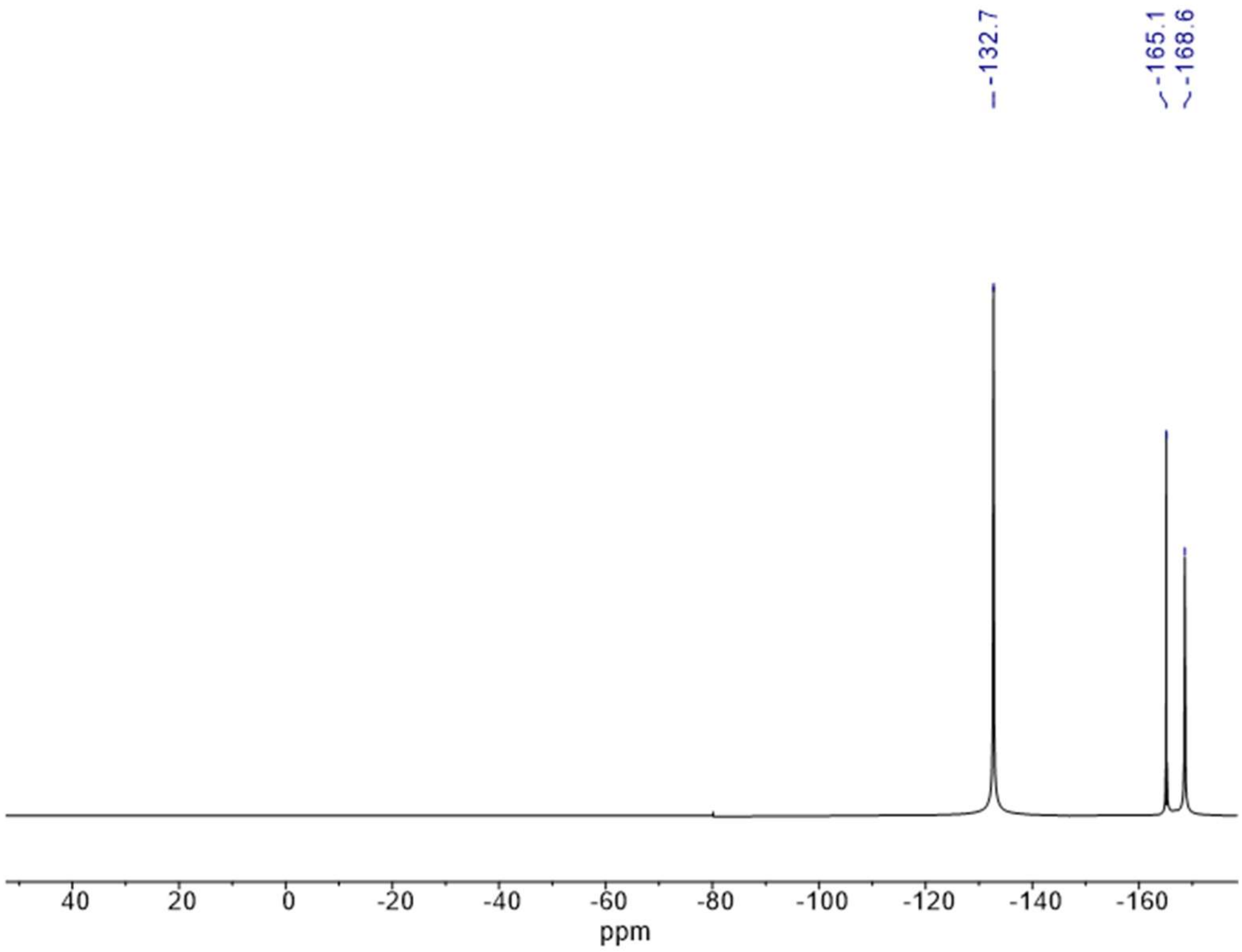

Figure S28. The ^{19}F NMR spectrum of **3a** (DiKTa-BtripS [B(C_6F_5)$_4$]) showing signals corresponding to the [B(C_6F_5)$_4$]$^-$ anion. Recorded in THF-d_8 at 298 K.

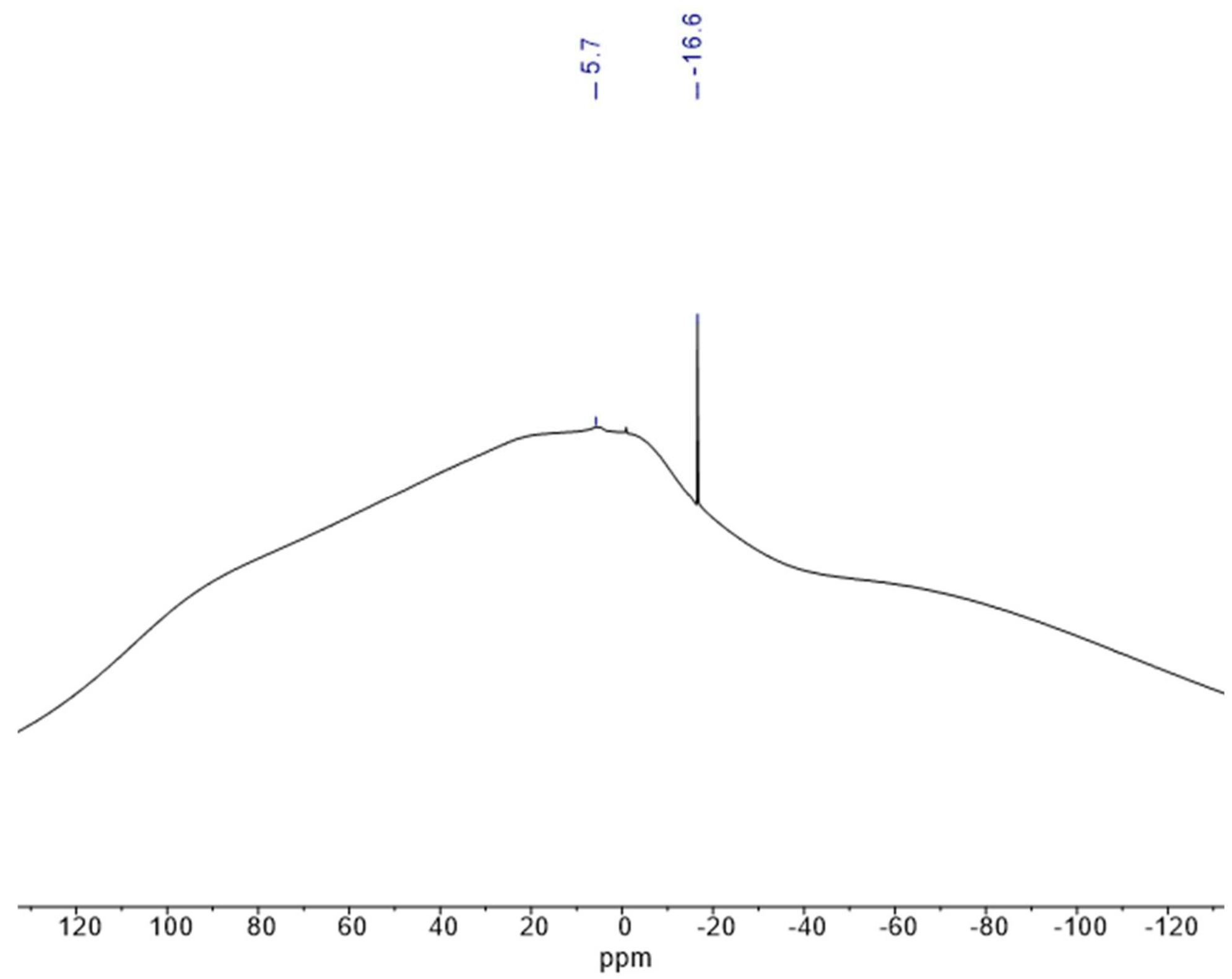

Figure S29. The ^{11}B NMR spectrum of **3a** (DiKTa-BtripS $[B(C_6F_5)_4]$) showing signals corresponding to bound BtripS (broad, 5.7 ppm) and the $[B(C_6F_5)_4]^-$ anion (sharp, –16.6 ppm). Recorded in THF-d_8 at 298 K.

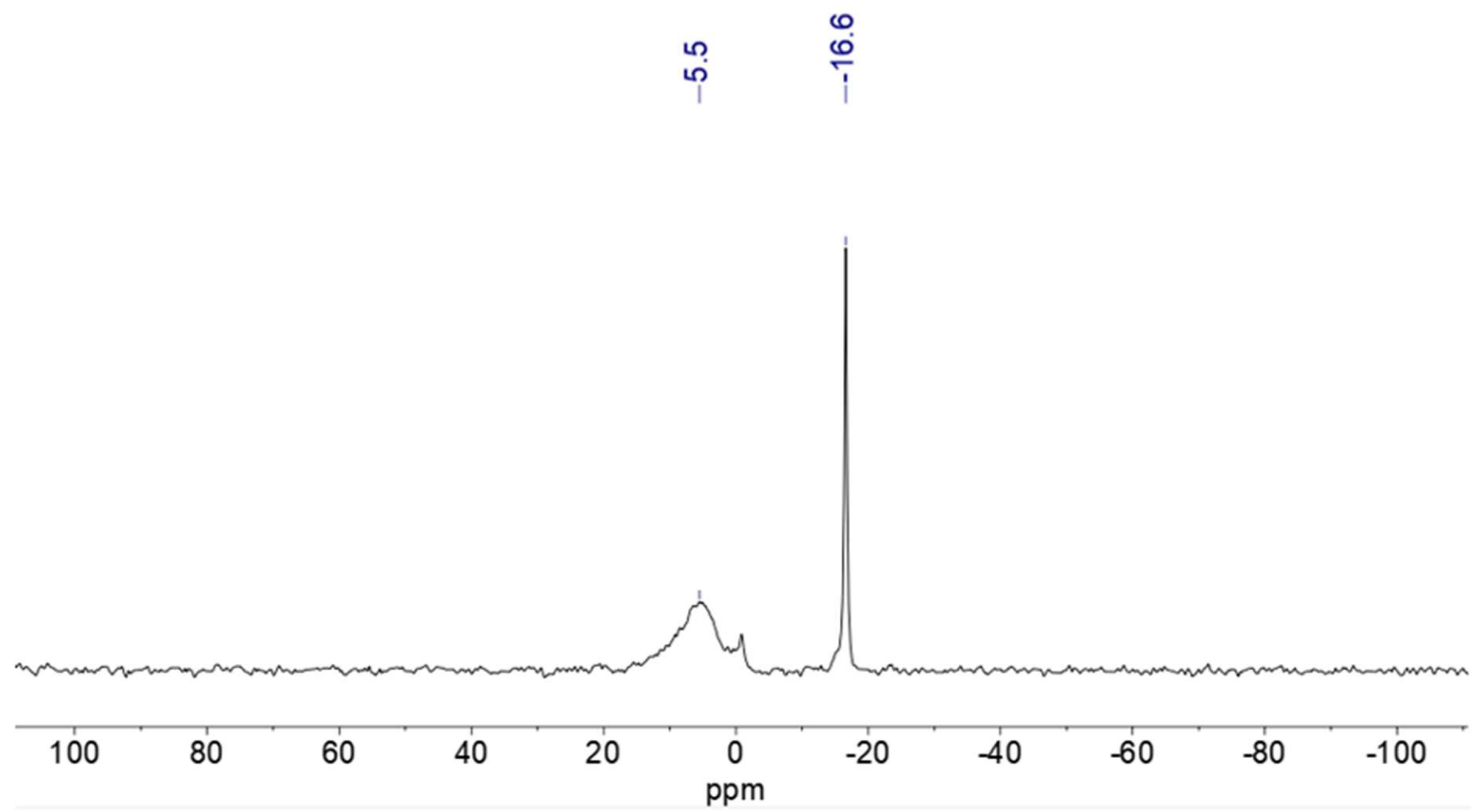

Figure 30. The [10]B NMR spectrum of **3a** (DiKTa-BtripS $[B(C_6F_5)_4]$) showing signals corresponding to the $[B(C_6F_5)_4]^-$ anion (sharp, – 16.6 ppm) and BtripS (broad, 5.5 ppm). Recorded in THF-d_8 at 298 K.

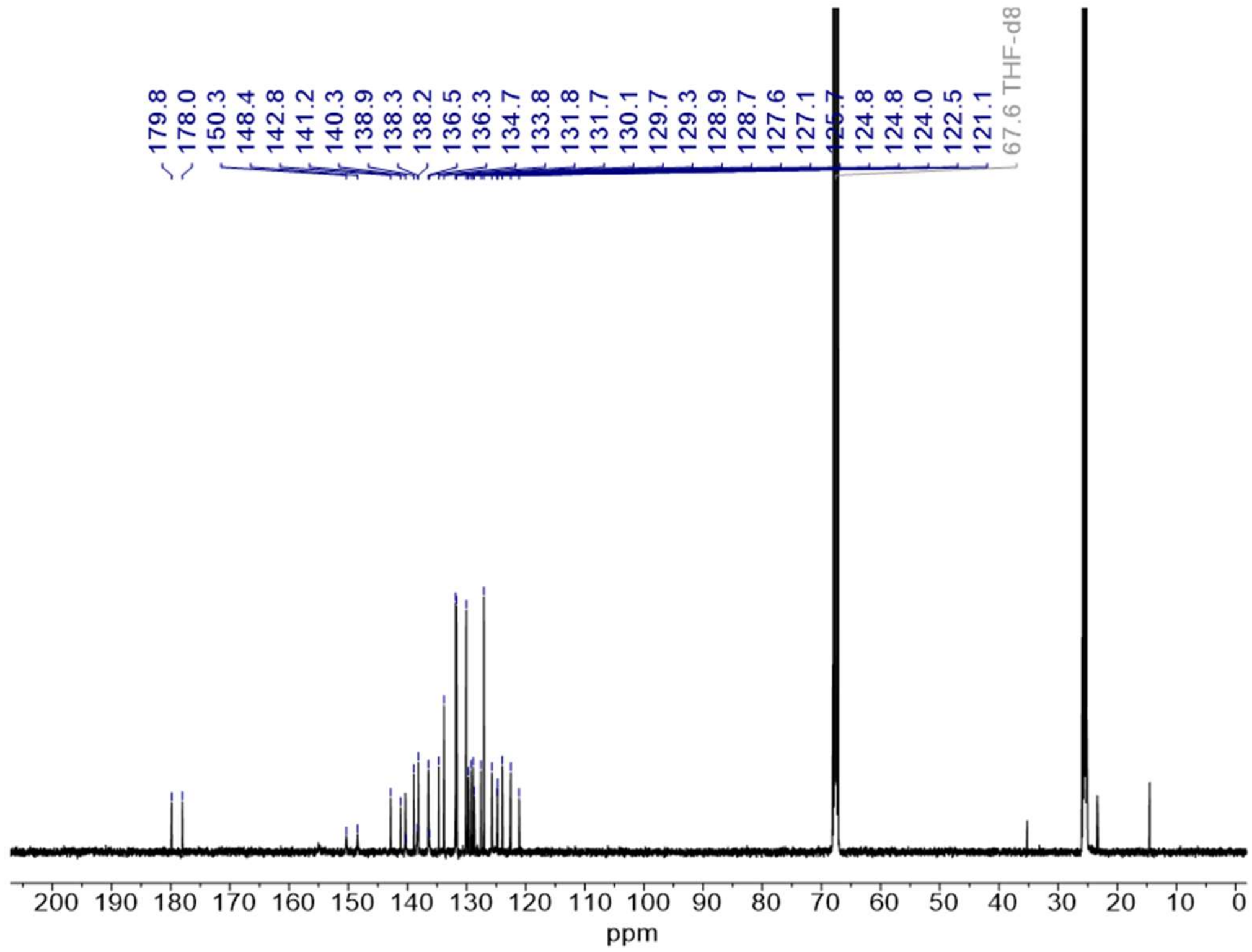

Figure S31. The ^{13}C NMR spectrum of **3a** (DiKTa-BtripS $[B(C_6F_5)_4]$) recorded in THF-d_8 at 298 K.

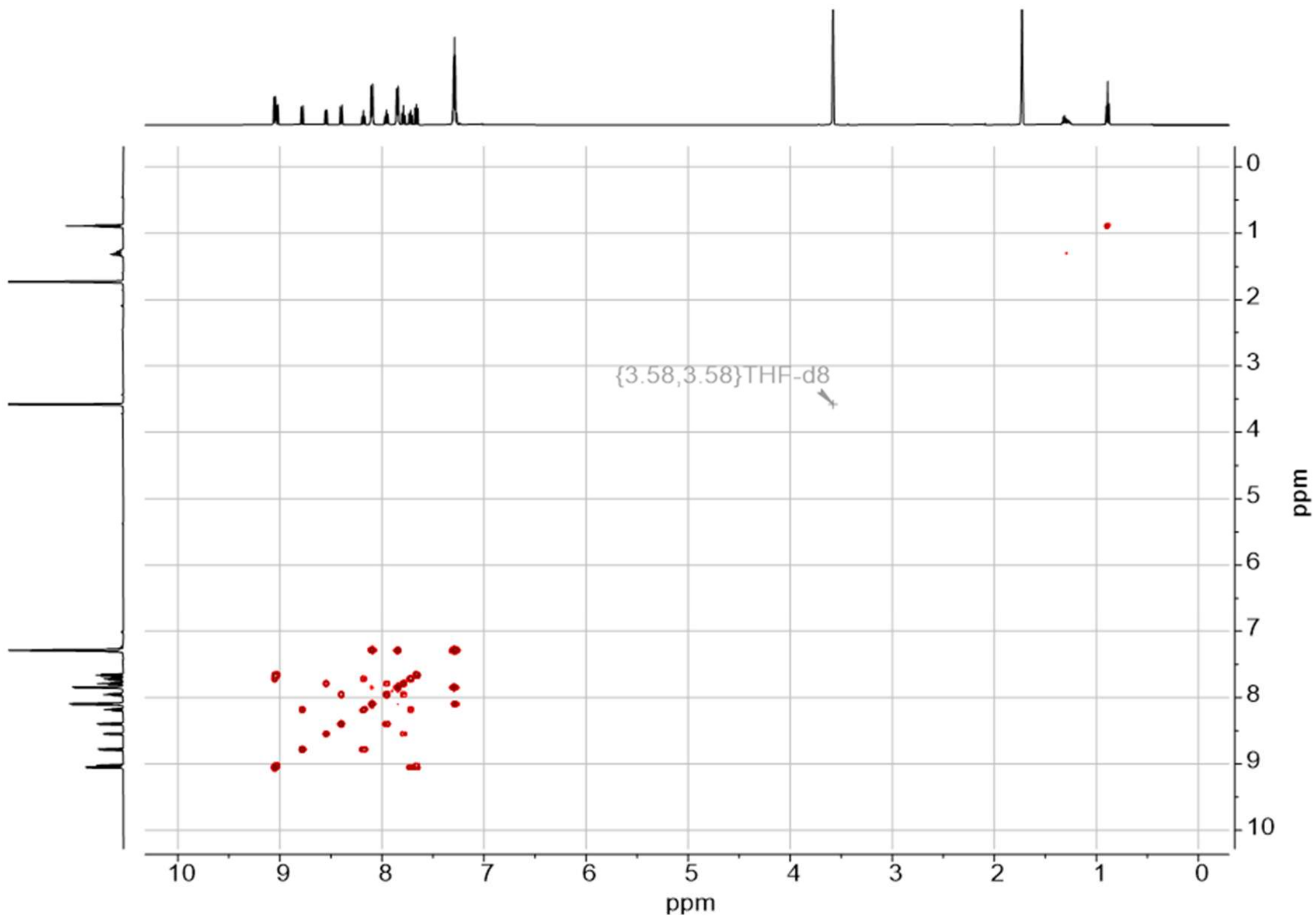

Figure S32. $^{1}H^{1}H$ COSY NMR spectrum of **3a** (DiKTa-BtripS $[B(C_6F_5)_4]$) recorded in THF-d_8 at 298 K.

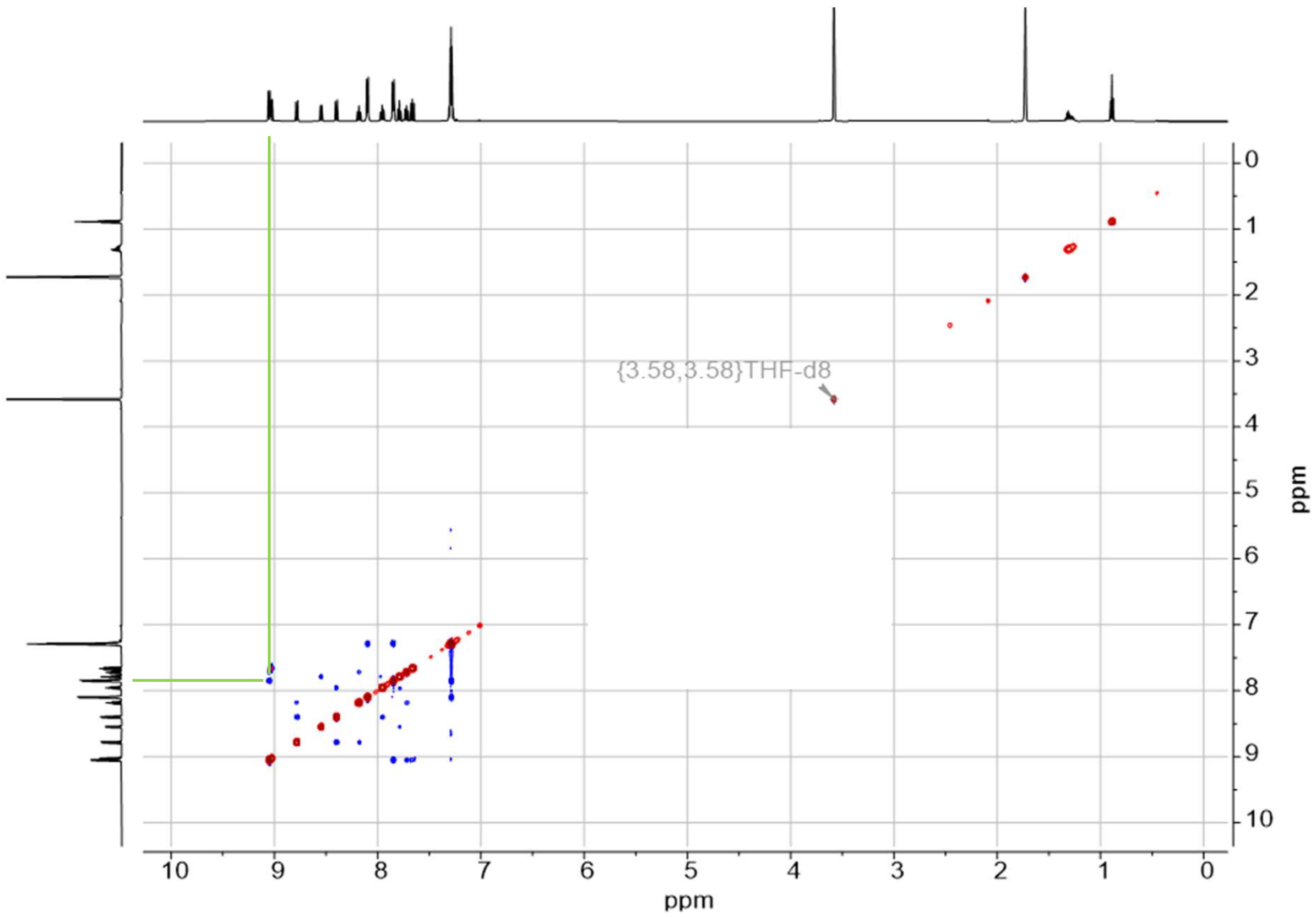

Figure S33. $^1H^1H$ NOESY NMR spectrum of **3a** (DiKTa-BtripS [$B(C_6F_5)_4$]) recorded in THF-d_8 at 298 K. A labelled NOE interaction (green lines) between all 3 proximal BtripS protons (highlighted in blue) and the 2 adjacent DiKTa phenyl protons.

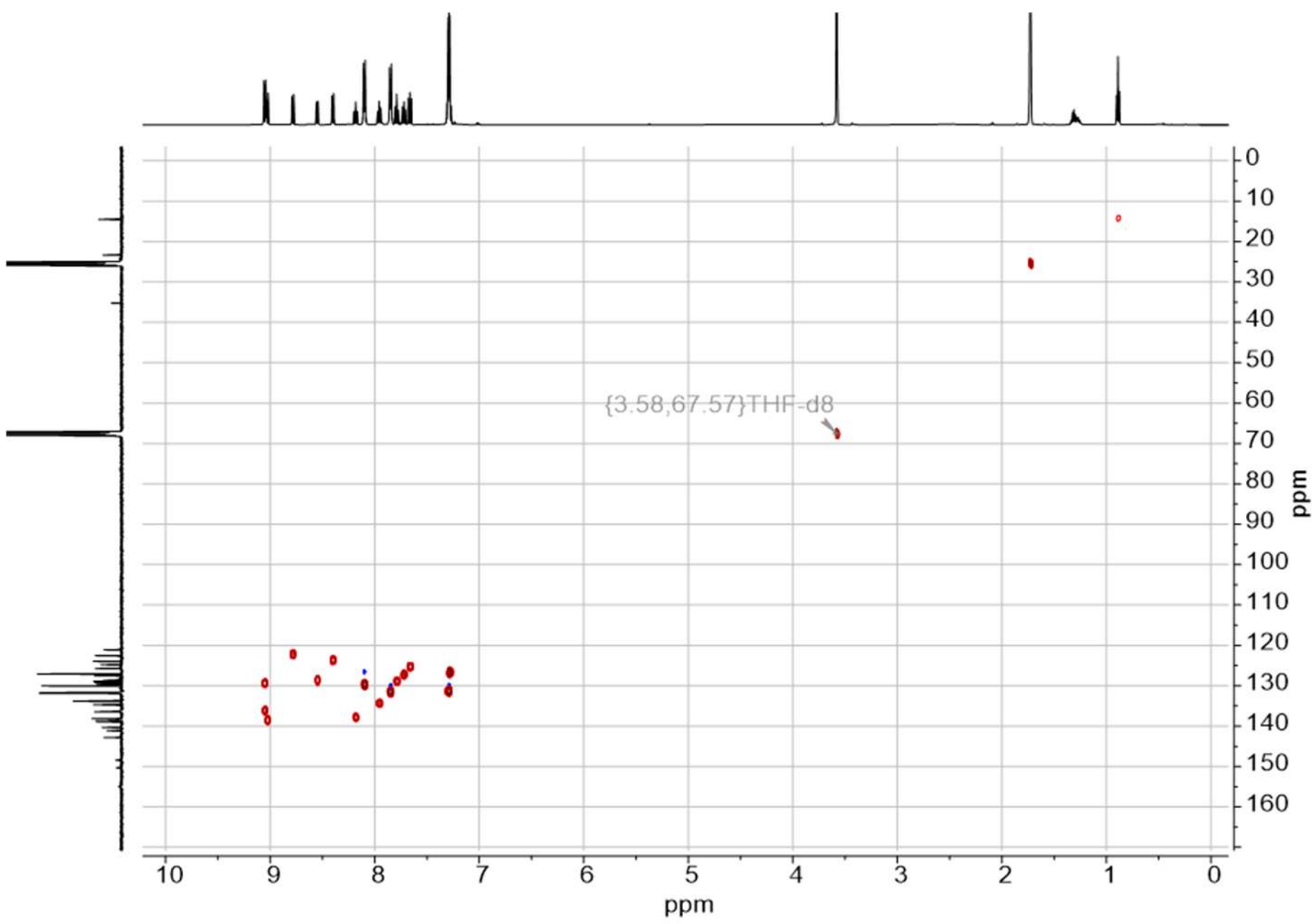

Figure S34. $^1H^{13}C$ HSQC NMR spectrum of **3a** (DiKTa-BtripS [$B(C_6F_5)_4$]) recorded in THF-d_8 at 298 K.

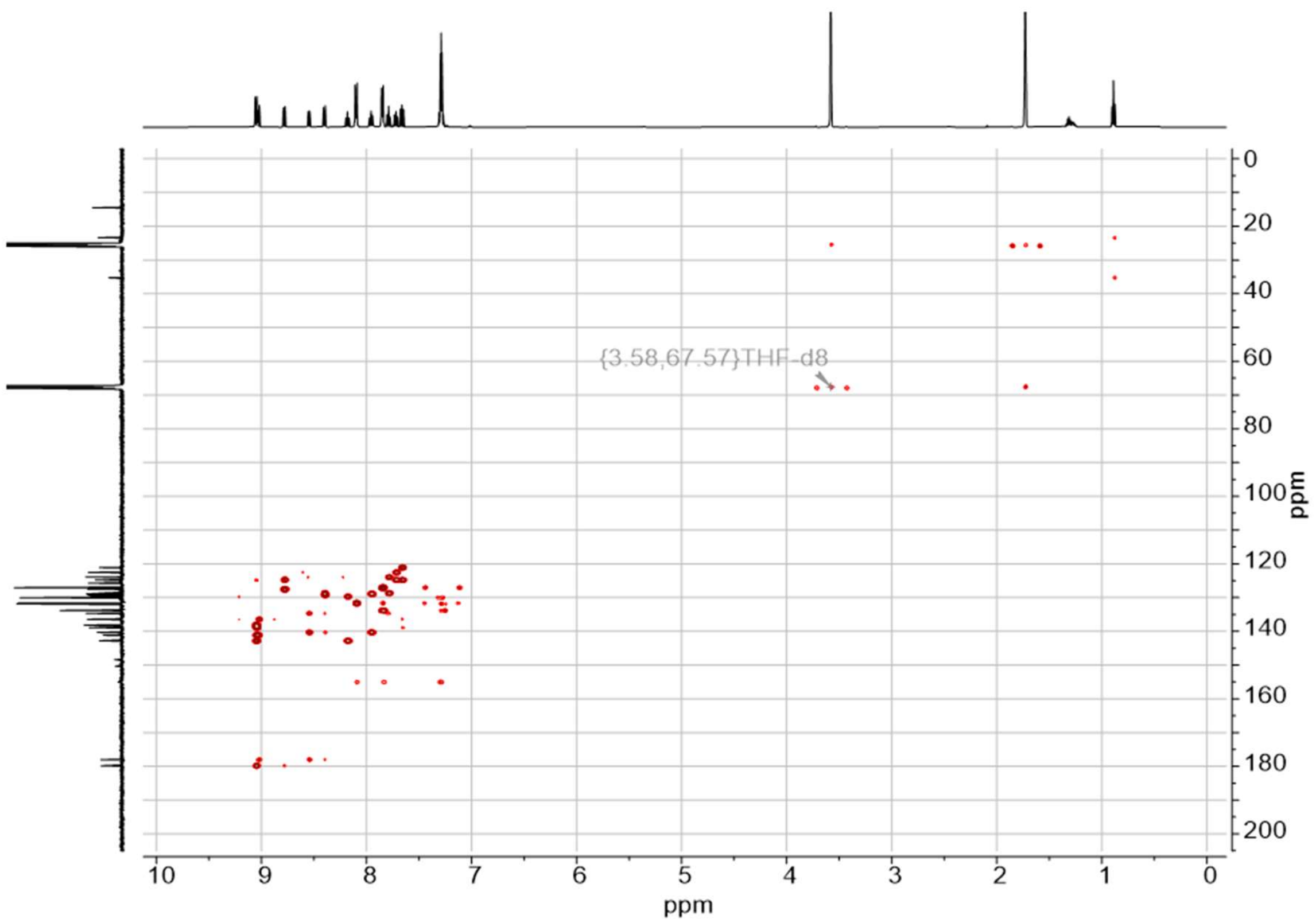

Figure S35. $^1H^{13}C$ HMBC NMR spectrum of **3a** (DiKTa-BtripS [$B(C_6F_5)_4$]) recorded in THF-d_8 at 298 K.

1.4.4 NMR spectra of 3-BCF (DiKTa-BCF)

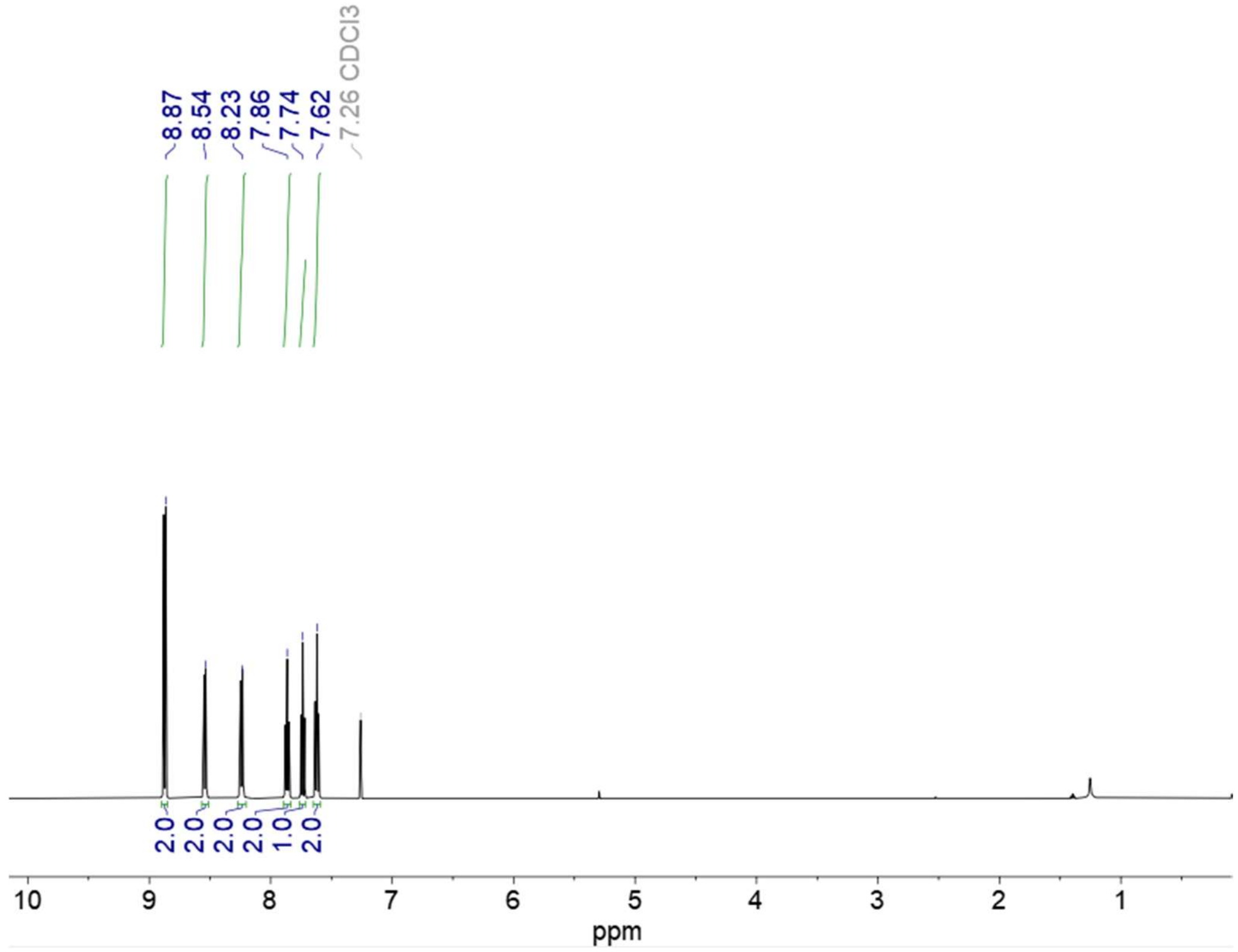

Figure S36. ^{1}H NMR spectrum of **3-BCF** (DiKTa-BCF). Recorded in-situ from synthesis in $CDCl_3$ at 298 K.

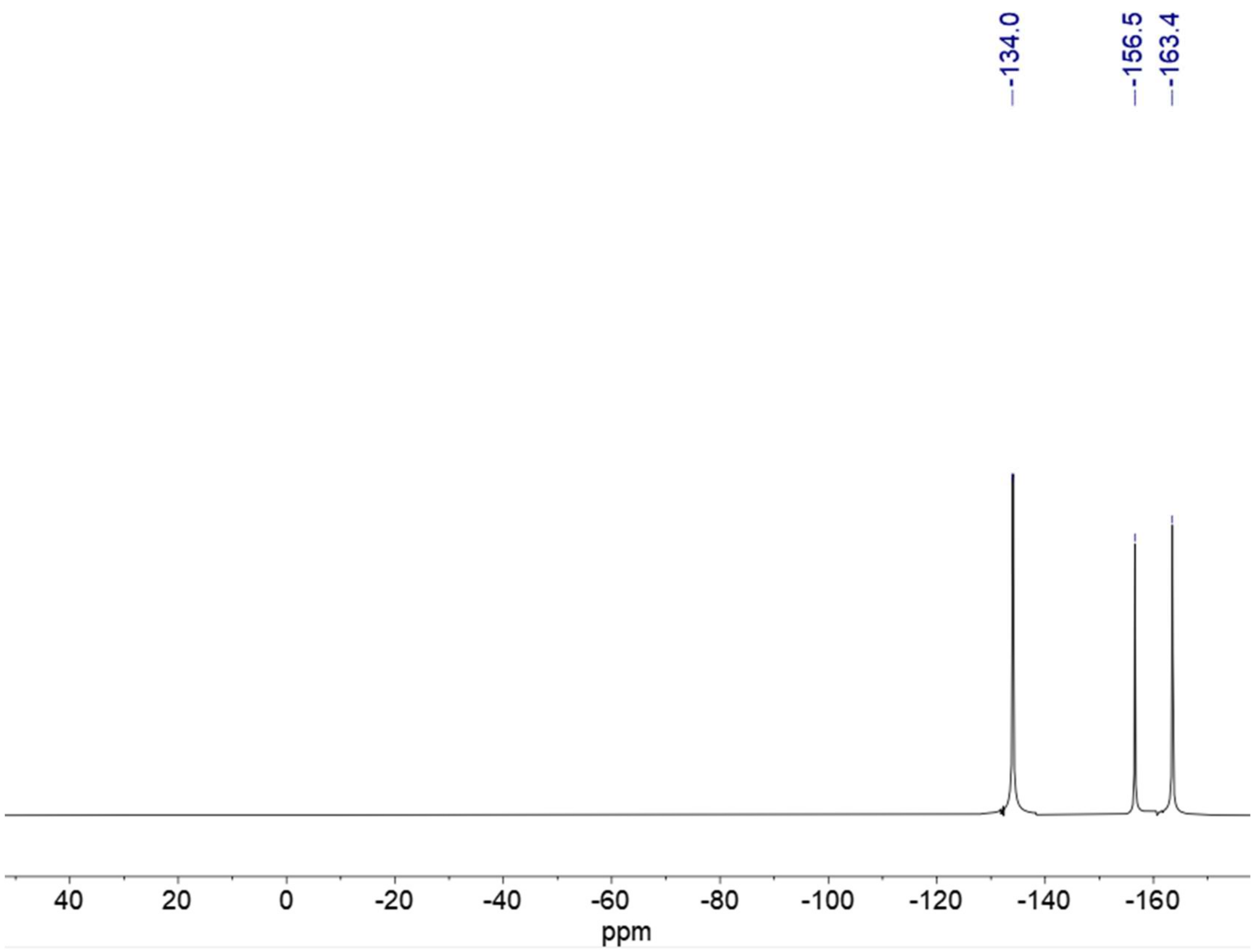

Figure S37. The ^{19}F NMR spectrum of **3-BCF** (DiKTa-BCF) showing signals corresponding to bound BCF. Recorded in $CDCl_3$ at 298 K.

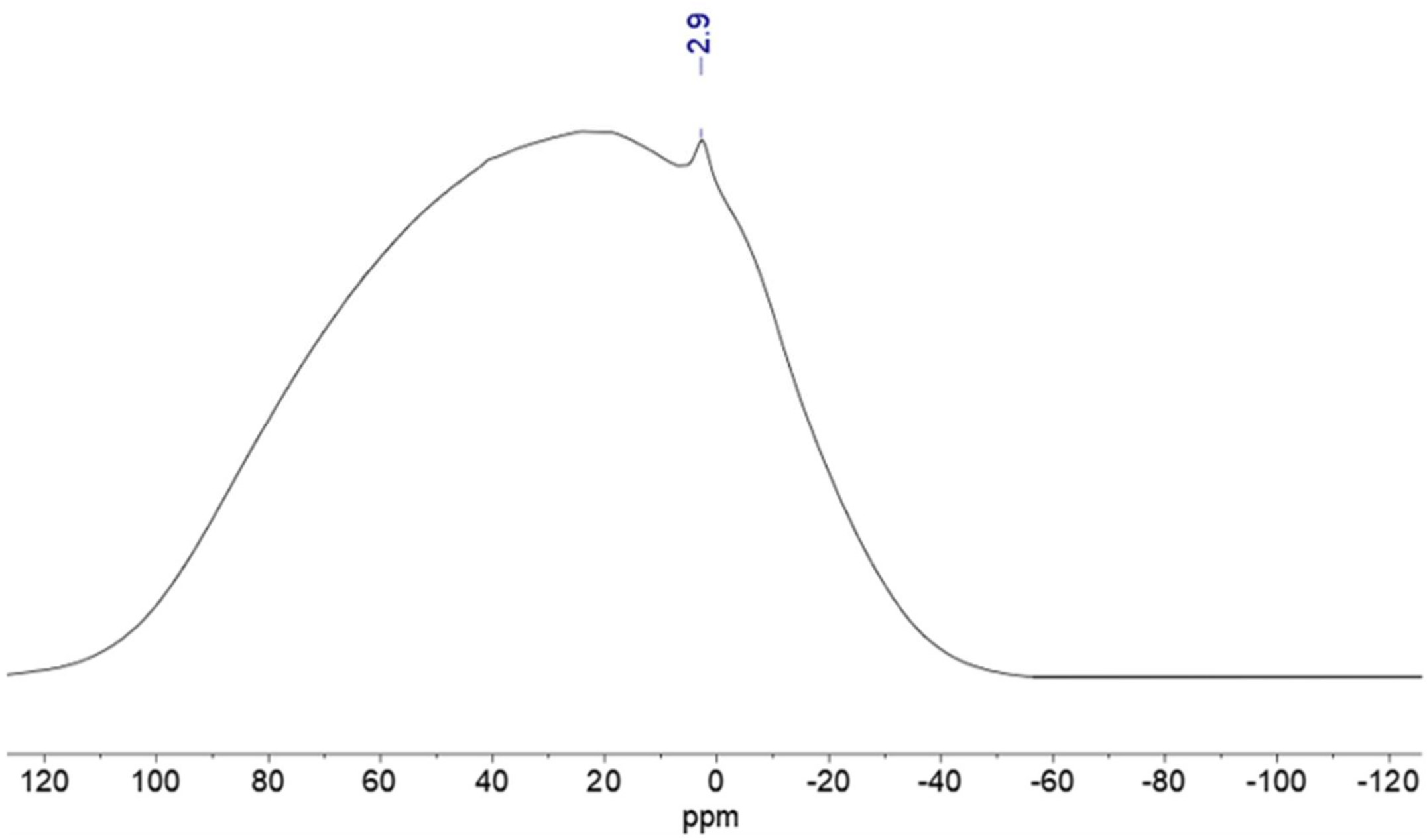

Figure S38. The ^{11}B NMR spectrum of **3-BCF** (DiKTa-BCF) showing a signal corresponding to bound BCF. Recorded in $CDCl_3$ at 298 K.

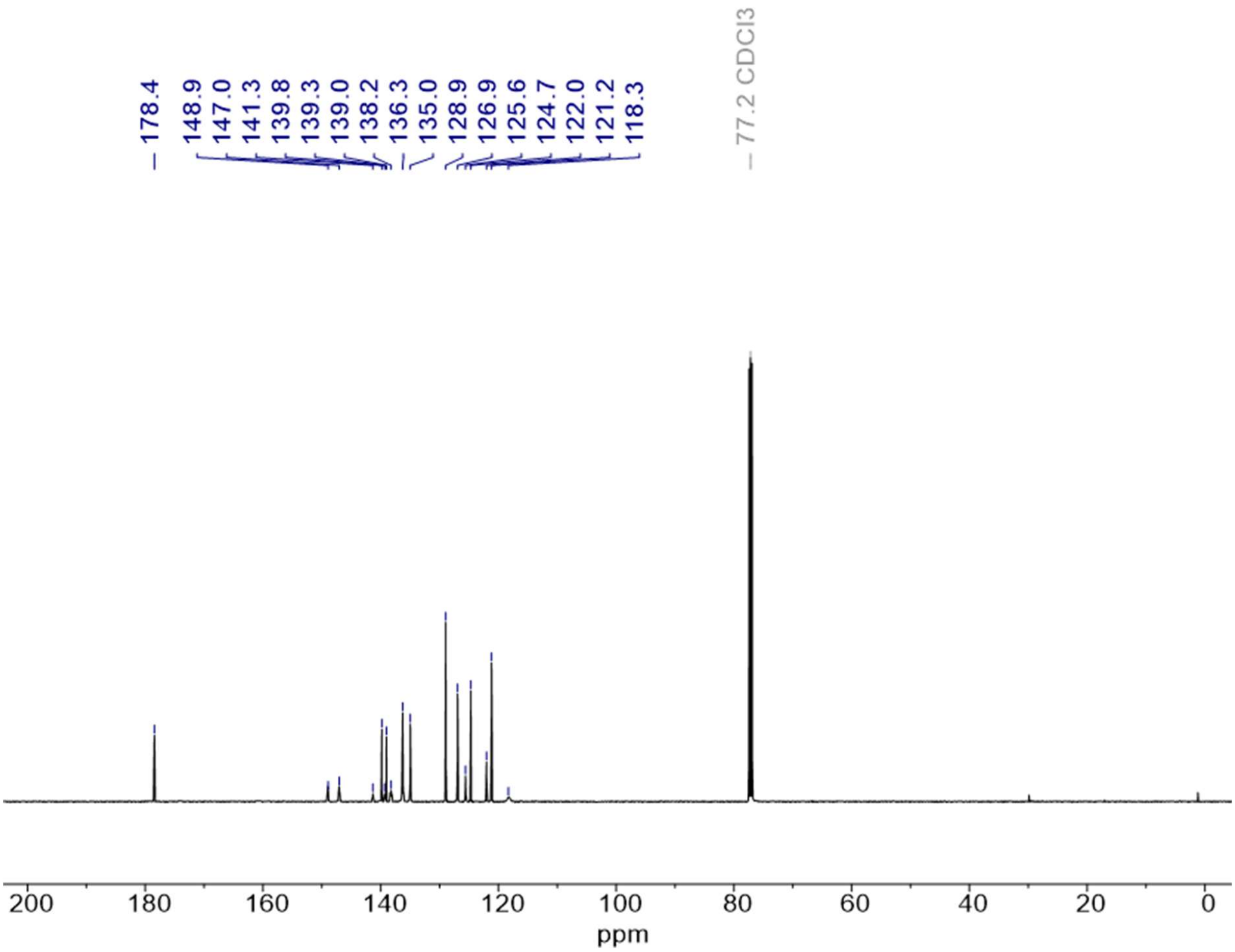

Figure S39. The ^{13}C NMR spectrum of **3-BCF** (DiKTa-BCF) recorded in $CDCl_3$ at 298 K showing 1 C=O signal at 178.4 ppm.

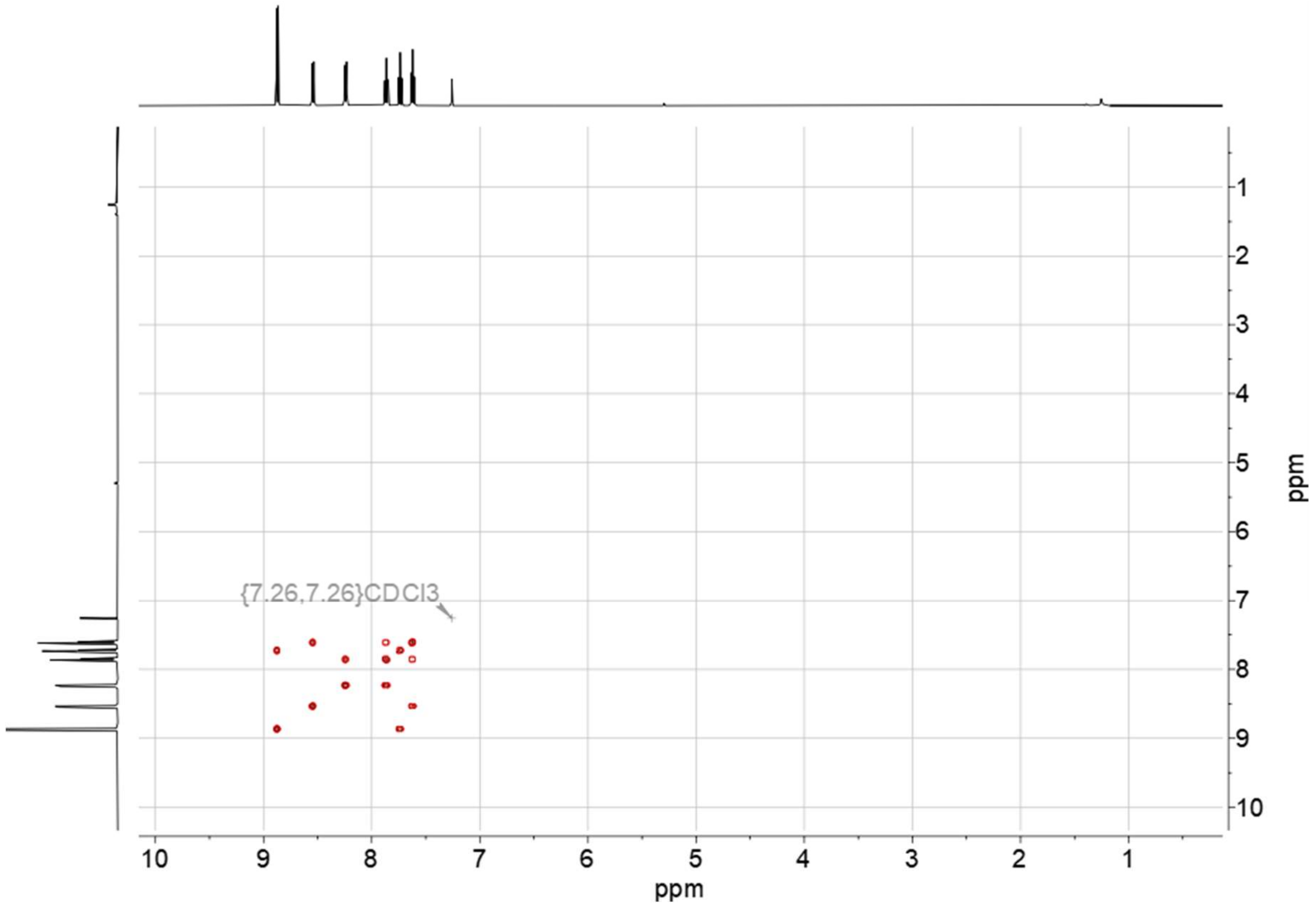

Figure S40. ^{1}H[1]H COSY NMR spectrum of **3-BCF** (DiKTa-BCF) recorded in $CDCl_3$ at 298 K.

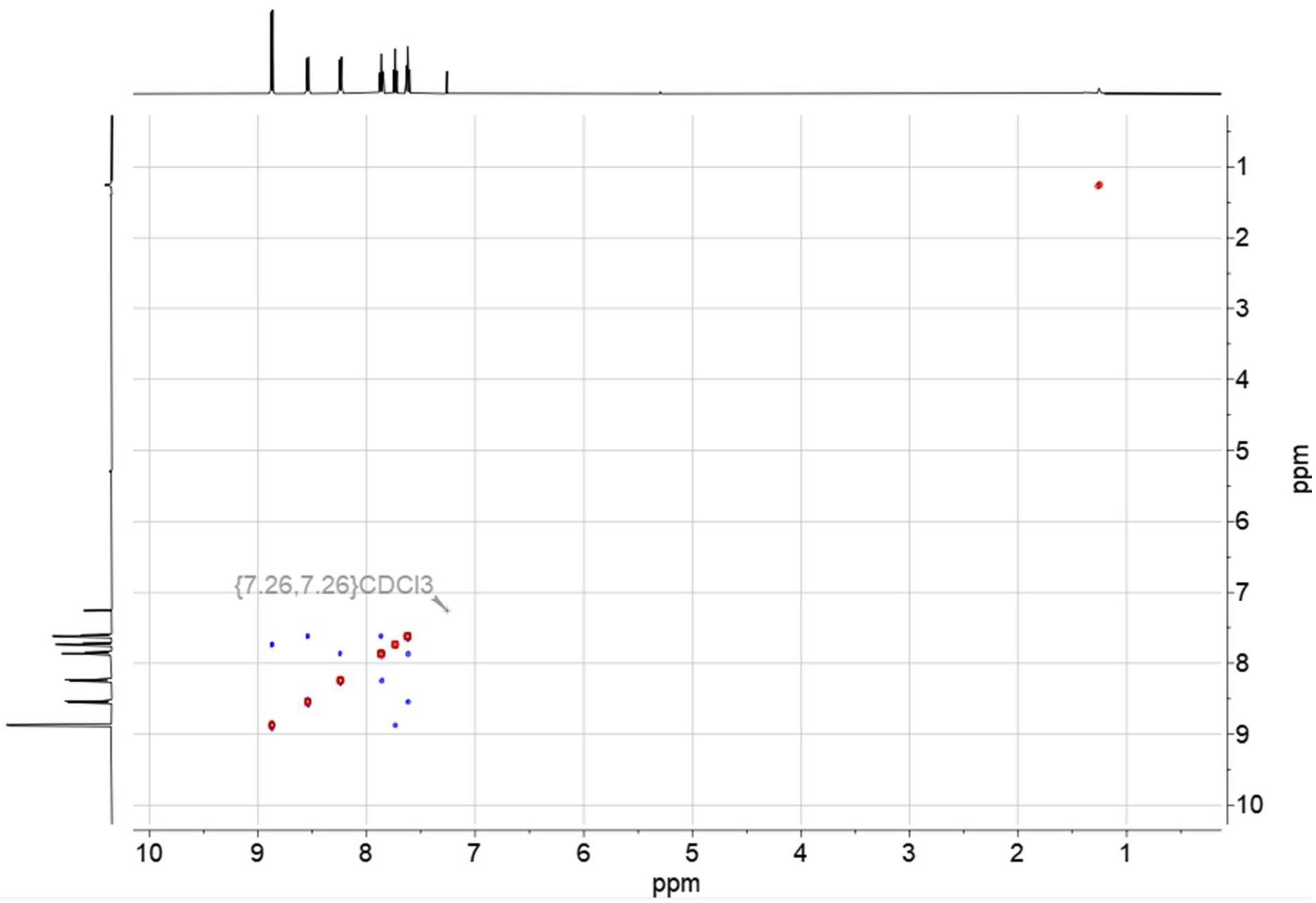

Figure S41. ^{1}H[1]H NOESY NMR spectrum of **3-BCF** (DiKTa-BCF) recorded in $CDCl_3$ at 298 K.

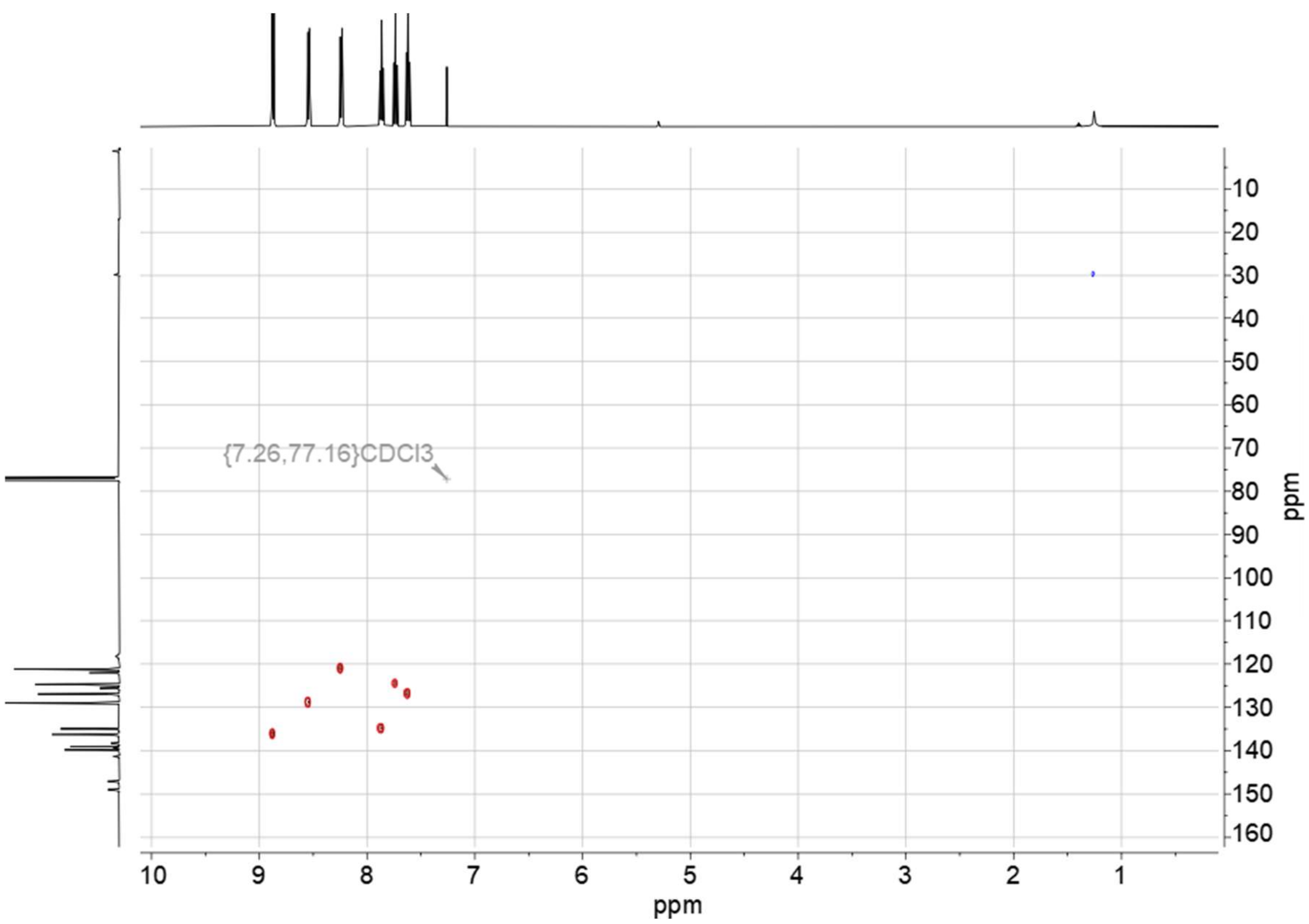

Figure S42. $^{1}H^{13}C$ HSQC NMR spectrum of **3-BCF** (DiKTa-BCF) recorded in $CDCl_3$ at 298 K.

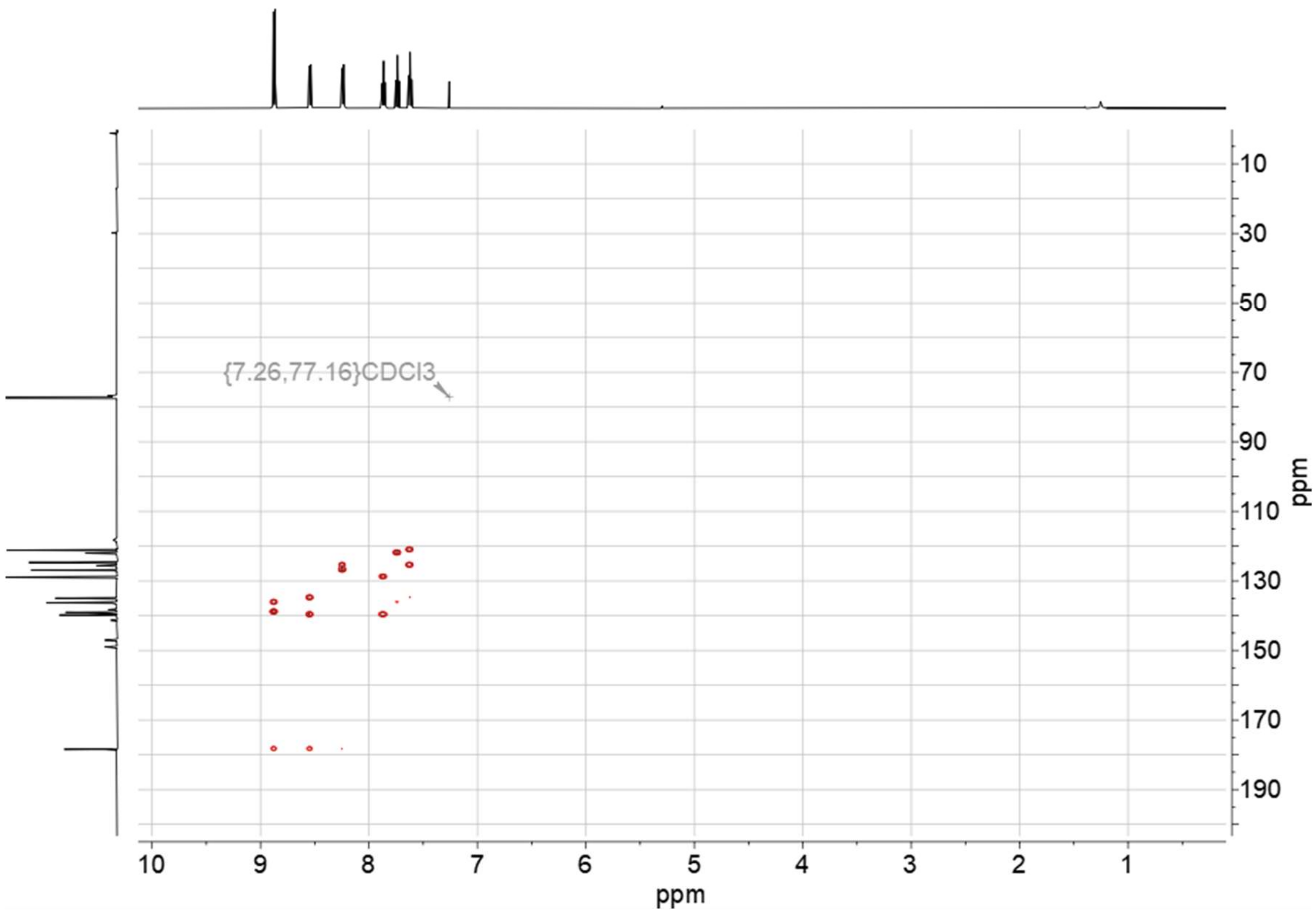

Figure S43. $^{1}H^{13}C$ HMBC NMR spectrum of **3-BCF** (DiKTa-BCF) recorded in $CDCl_3$ at 298 K.

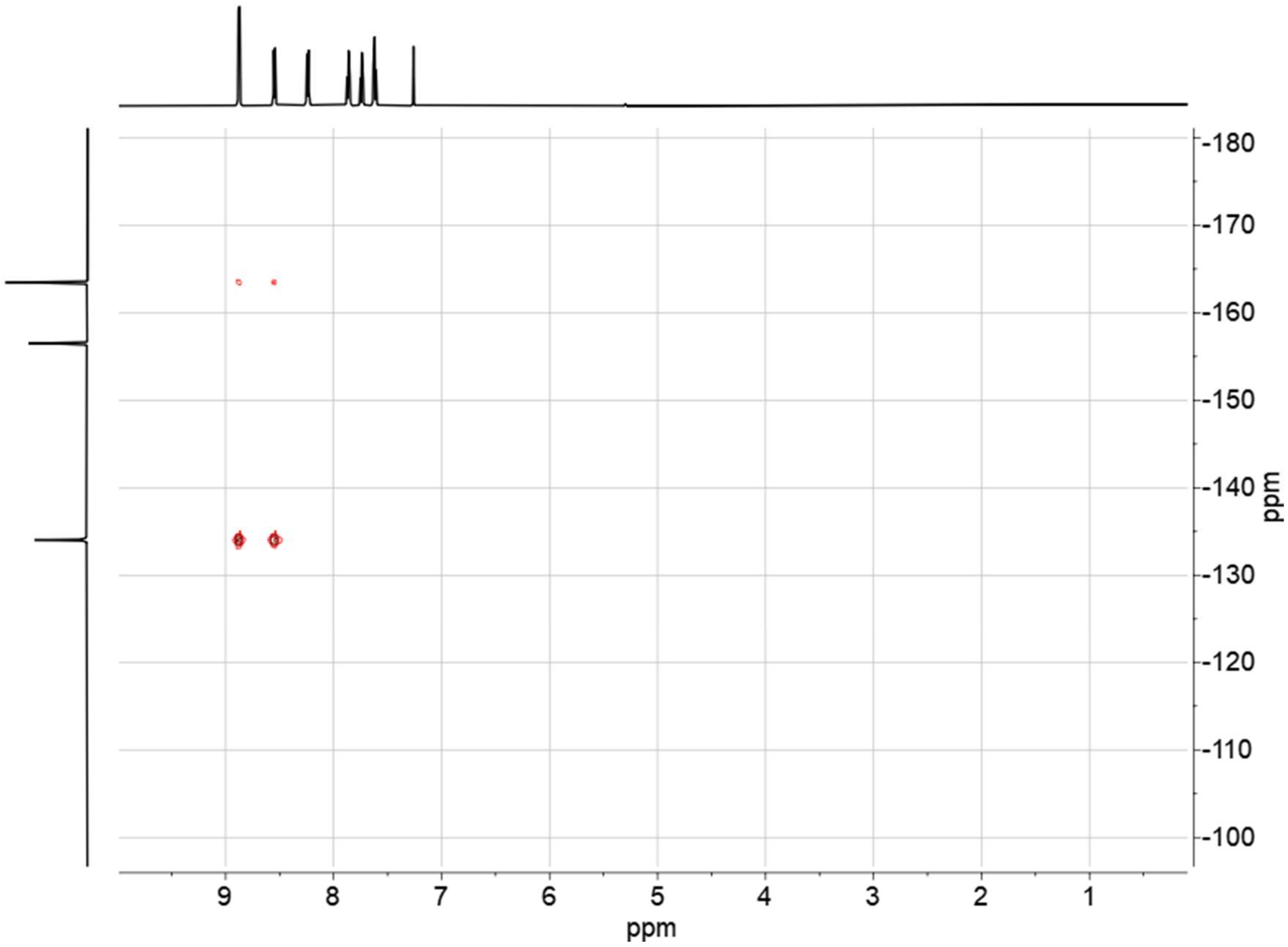

Figure S44. $^1H^{19}F$ HOESY NMR spectrum of **3-BCF** (DiKTa-BCF) recorded in $CDCl_3$ at 298 K.

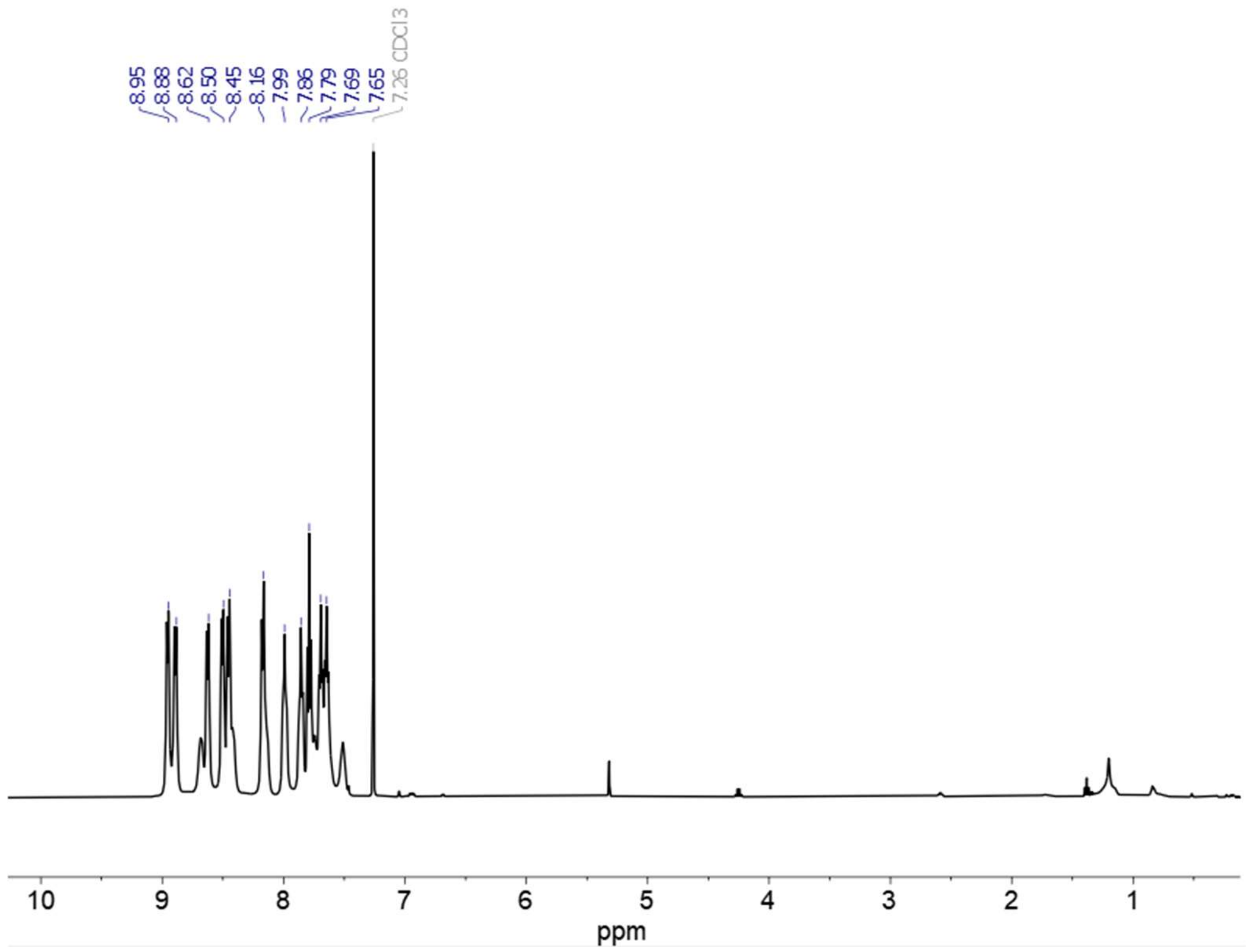

Figure S45. ^1H NMR spectrum of **3-BCF** (DiKTa-BCF). Recorded in-situ from synthesis in $CDCl_3$ at 235 K.

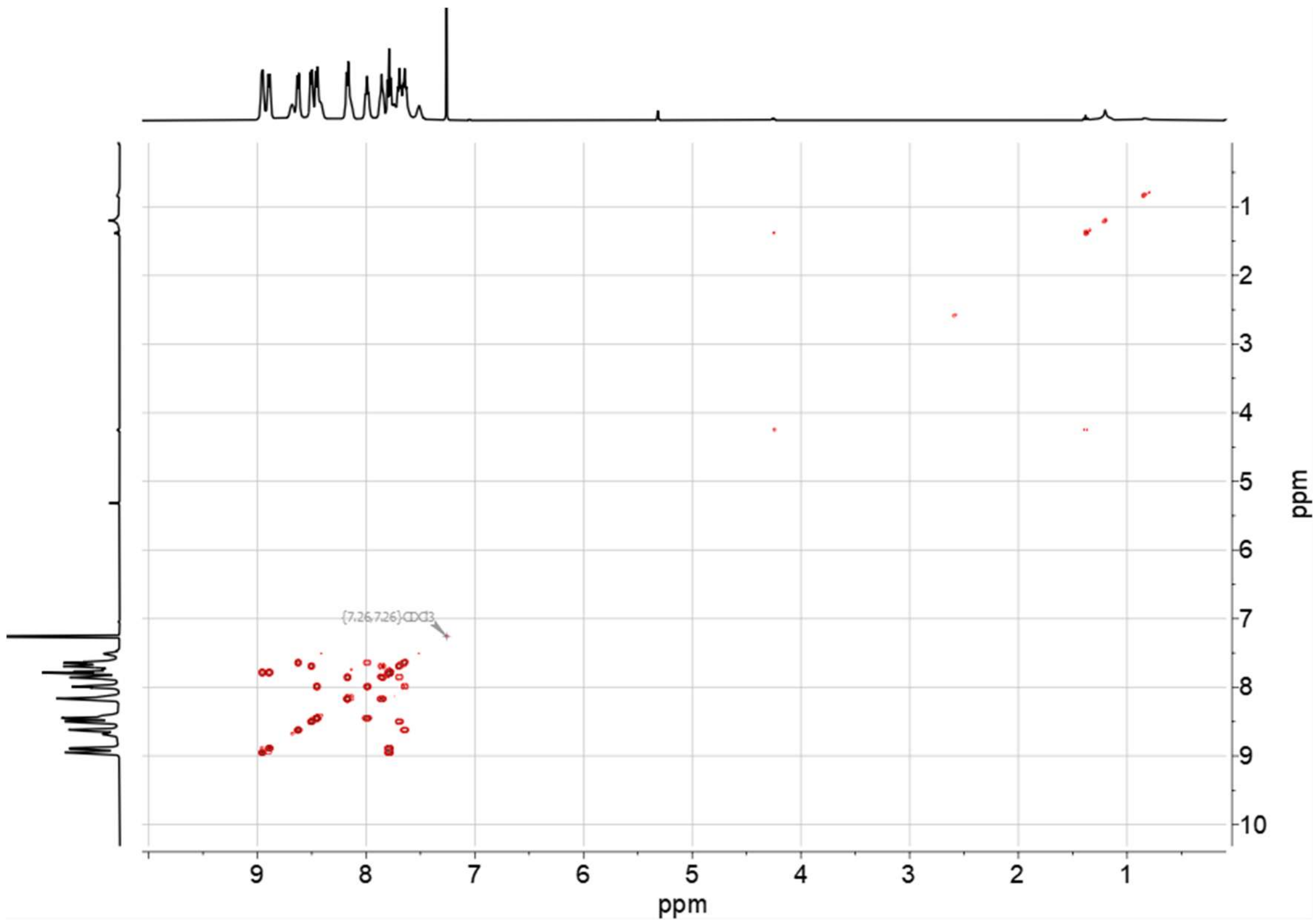

Figure S46. $^{1}H^{1}H$ COSY NMR spectrum of **3-BCF** (DiKTa-BCF) recorded in $CDCl_3$ at 235 K.

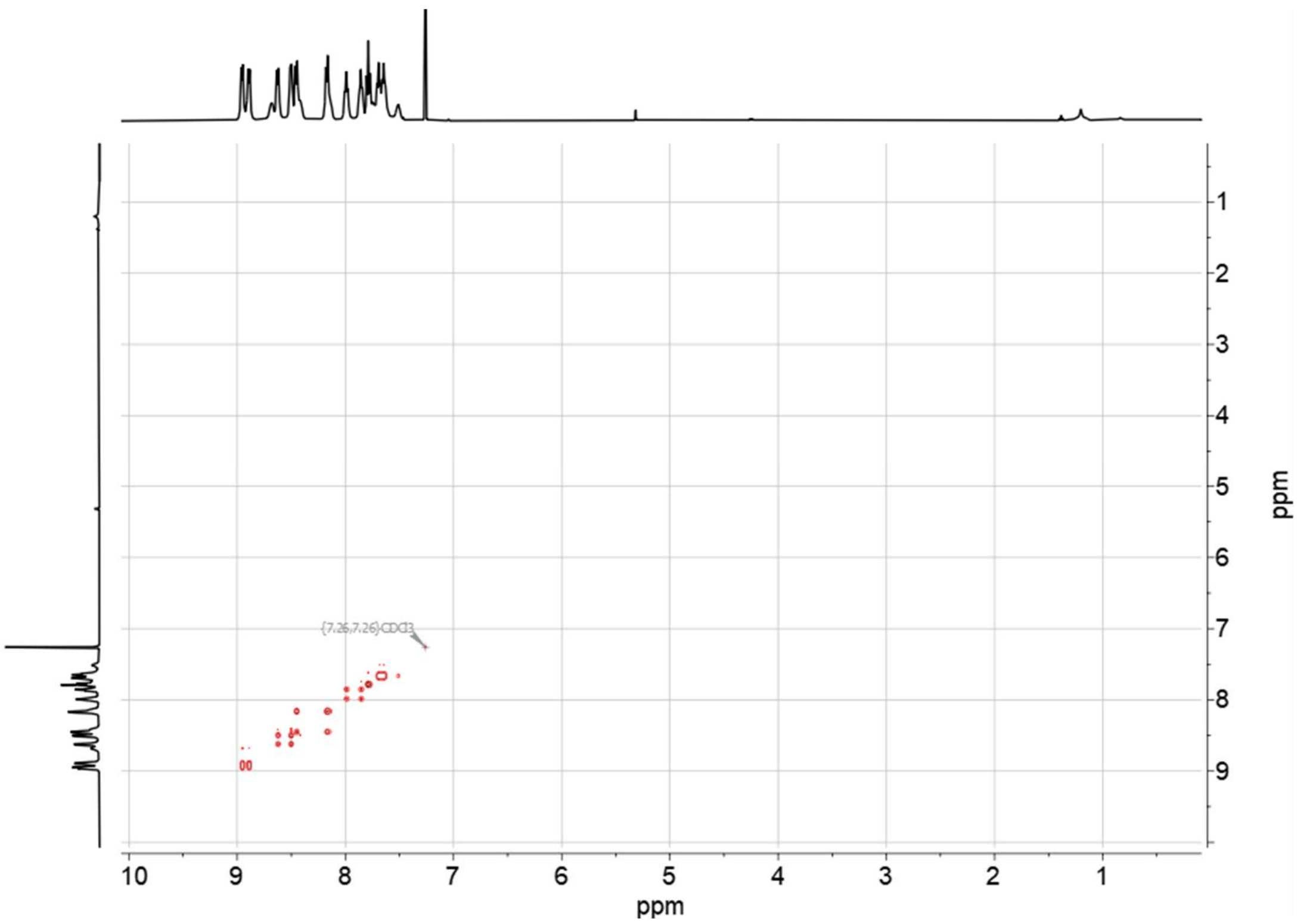

Figure S47. $^{1}H^{1}H$ NOESY NMR spectrum of **3-BCF** (DiKTa-BCF) recorded in $CDCl_3$ at 235 K.

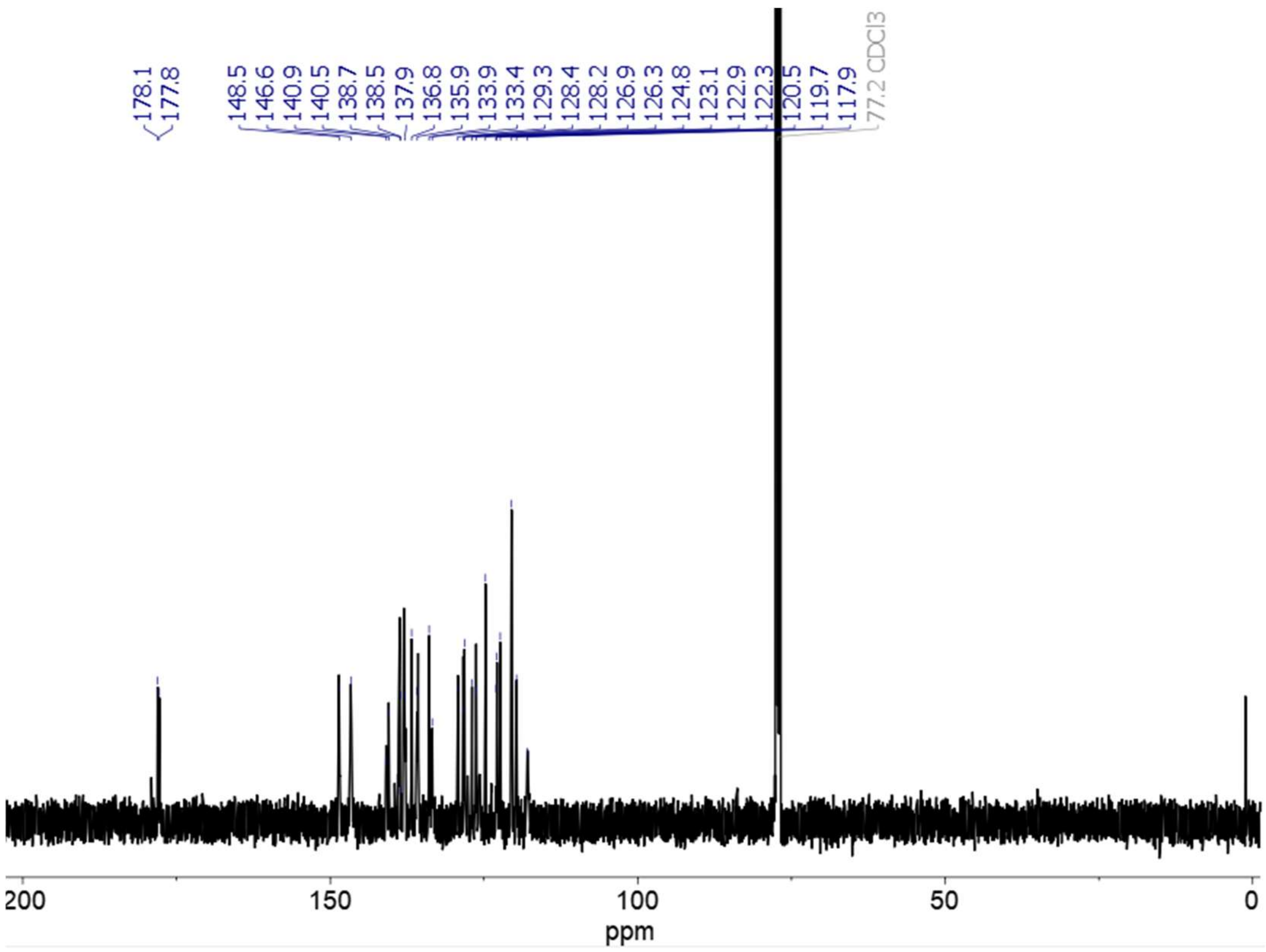

Figure S48. The ^{13}C NMR spectrum of **3-BCF** (DiKTa-BCF) recorded in $CDCl_3$ at 298 K, showing two C=O signals at 177.8 and 178.1 ppm.

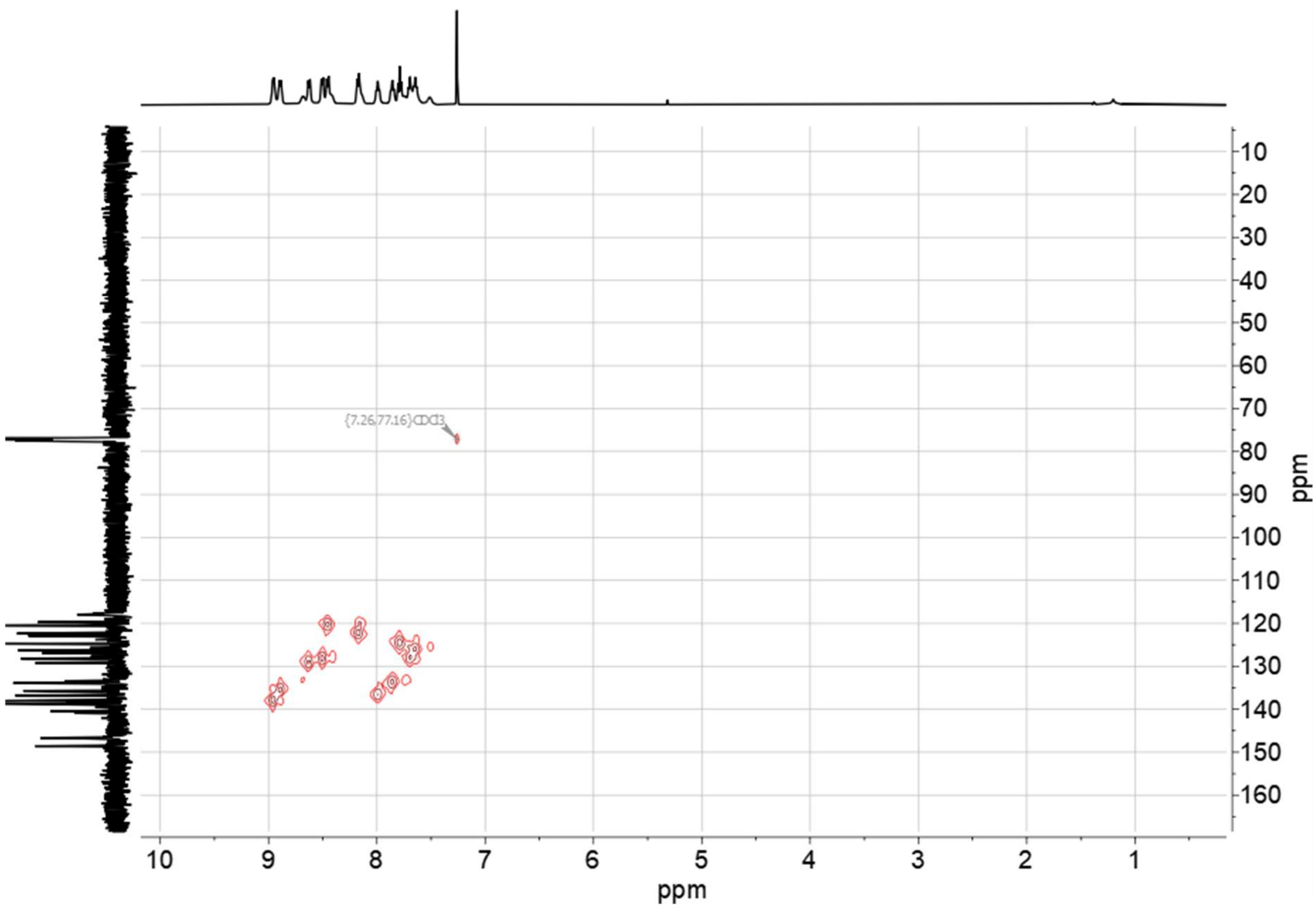

Figure S49. $^{1}H^{13}C$ HSQC NMR spectrum of **3-BCF** (DiKTa-BCF) recorded in $CDCl_3$ at 235 K.

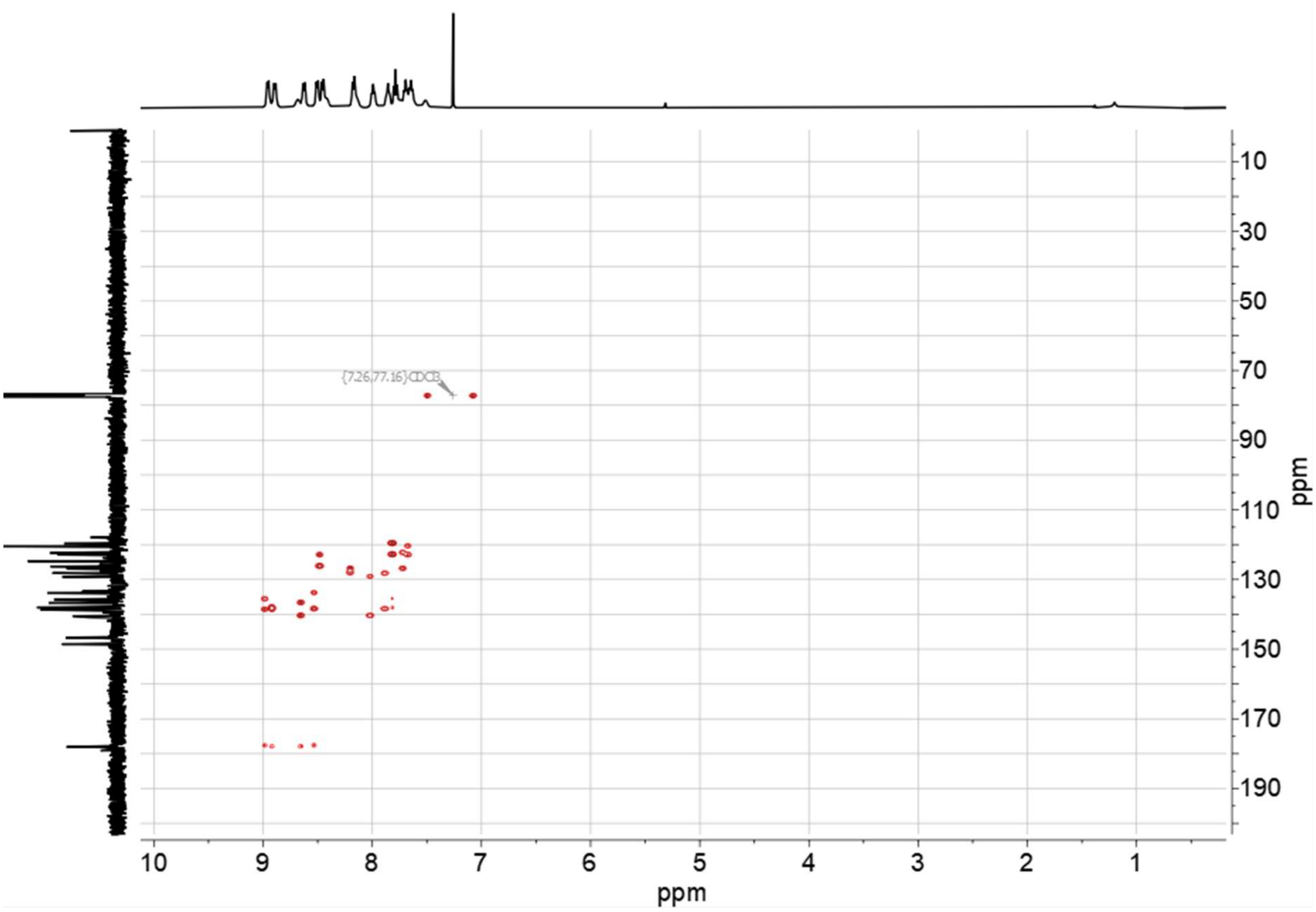

Figure S50. $^{1}H^{13}C$ HMBC NMR spectrum of **3-BCF** (DiKTa-BCF) recorded in $CDCl_3$ at 298 K.

1.4.5 NMR spectra of 4a (QPO-BtripS [$B(C_6F_5)_4$])

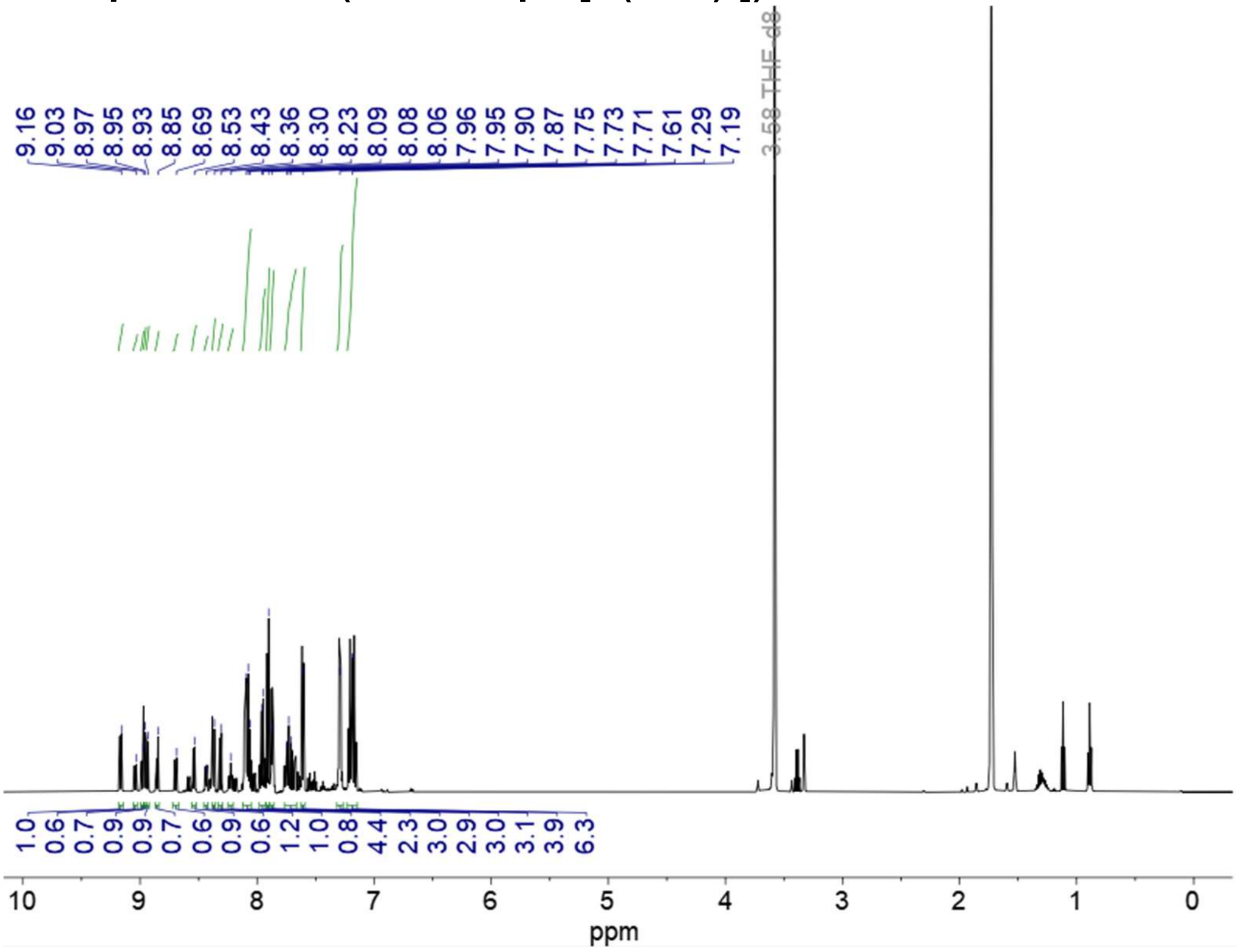

Figure S51. ^{1}H NMR spectrum of **4a** (QPO-BtripS [$B(C_6F_5)_4$]) showing two isomers present in a 3:2 ratio (**4a₂** & **4a₁**) and residual solvent from workup. Recorded in THF-d_8 at 298 K.

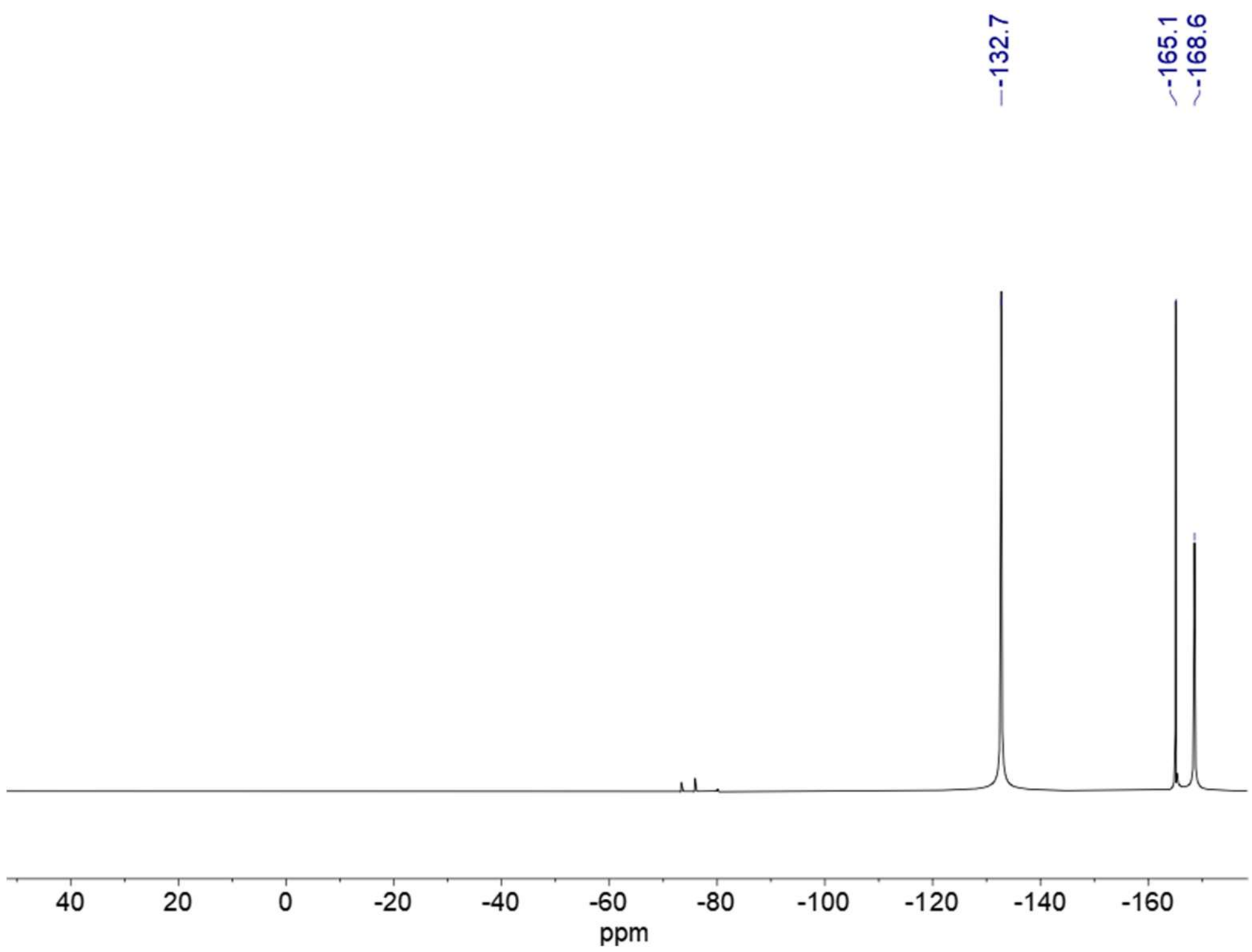

Figure S52 The ^{19}F NMR spectrum of **4a** (QPO-BtripS [$B(C_6F_5)_4$]) showing signals corresponding to the $[B(C_6F_5)_4]^-$ anion. Recorded in THF-d_8 at 298 K.

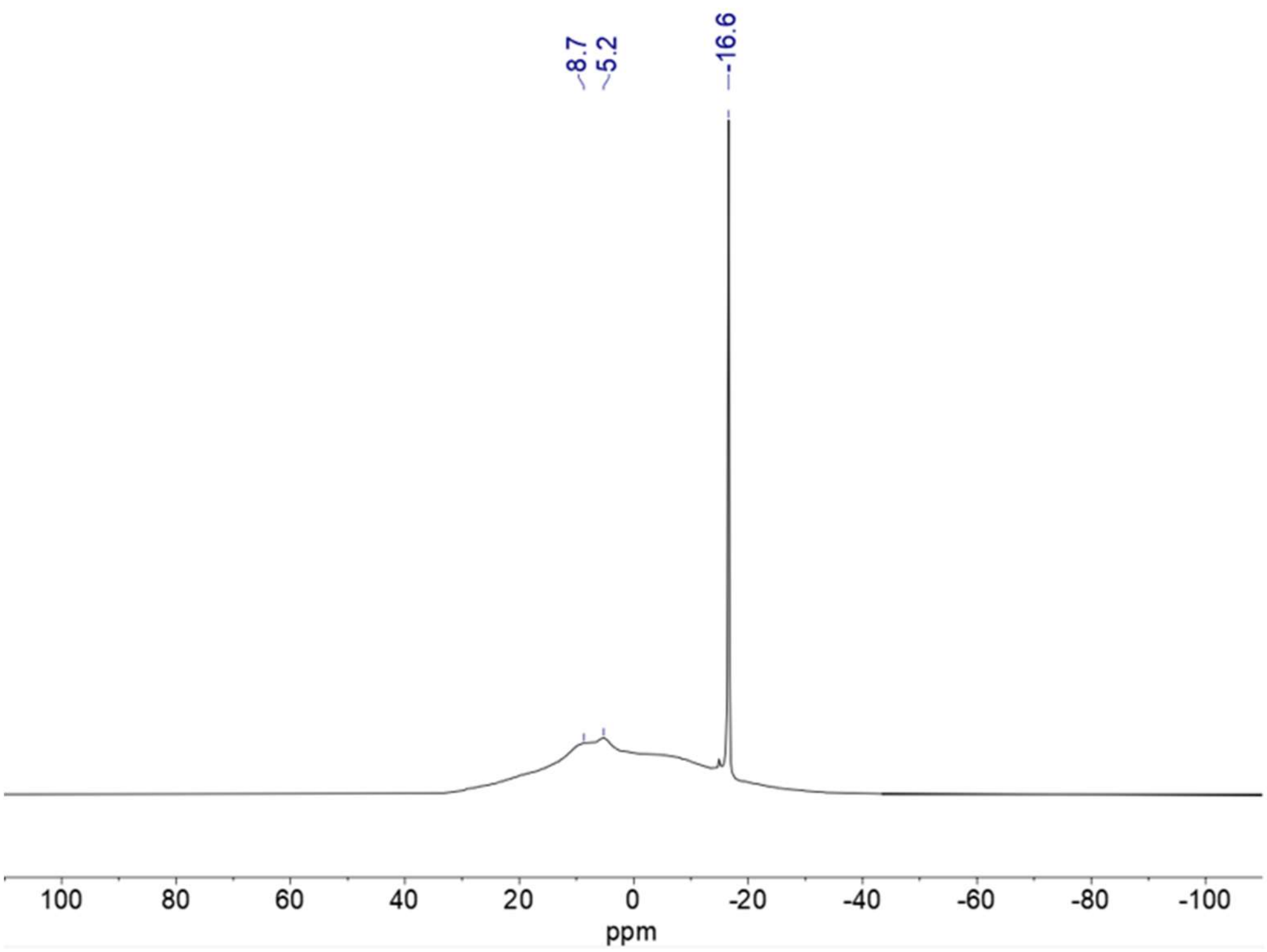

Figure S53. The ^{11}B NMR spectrum of **4a** (QPO-BtripS $[B(C_6F_5)_4]$) showing signals corresponding to the $[B(C_6F_5)_4]^-$ anion (sharp, – 16.6 ppm) and BtripS bound to the sulfone and to the carbonyl unit (broad, 8.7 & 5.2 ppm). Recorded in THF-d_8 at 298 K.

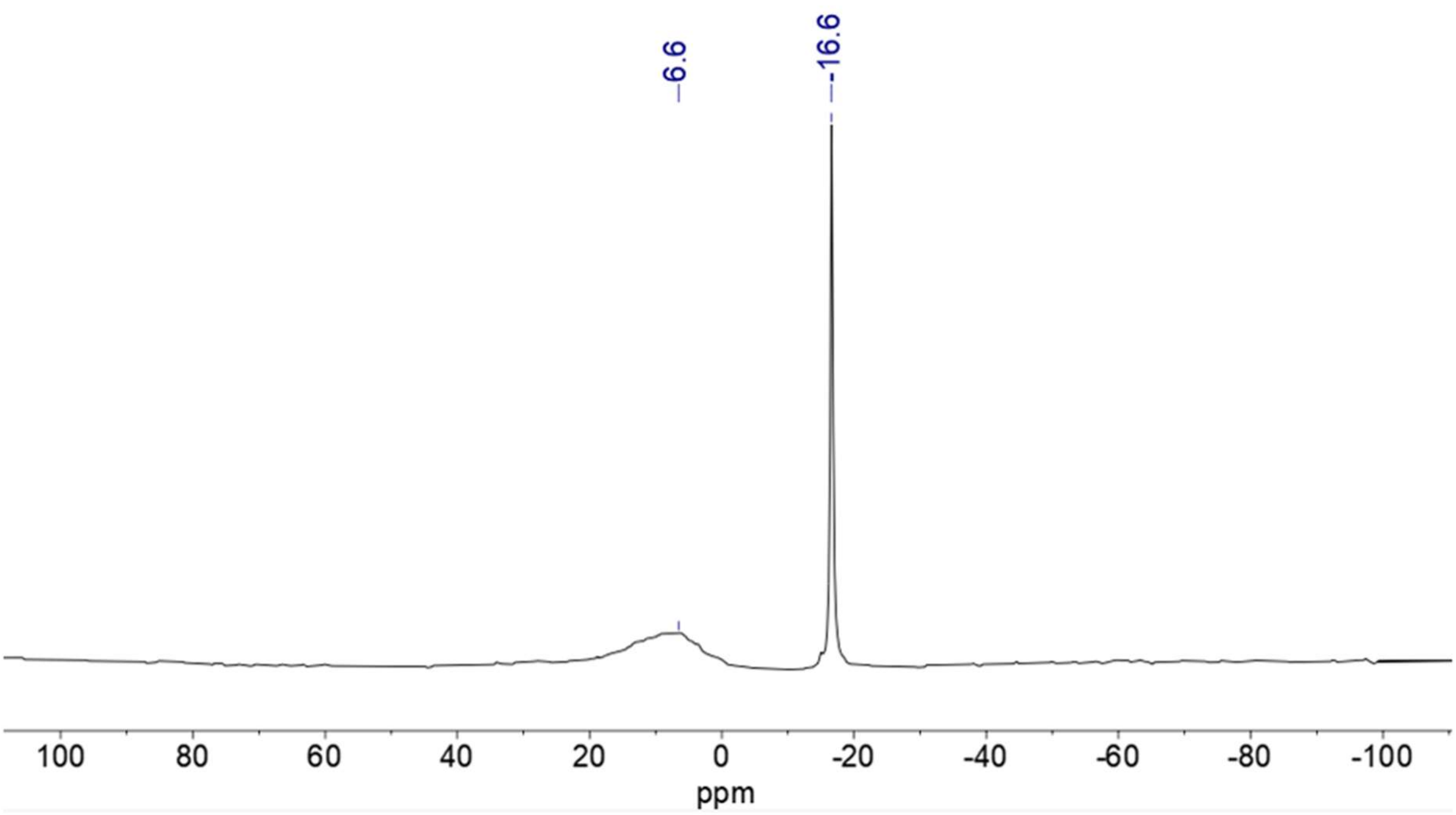

Figure S54. The ^{10}B NMR spectrum of **4a** (QPO-BtripS $[B(C_6F_5)_4]$) showing signals corresponding to the $[B(C_6F_5)_4]^-$ anion (sharp, – 16.6 ppm) and BtripS (broad, 6.6 ppm). Recorded in THF-d_8 at 298 K.

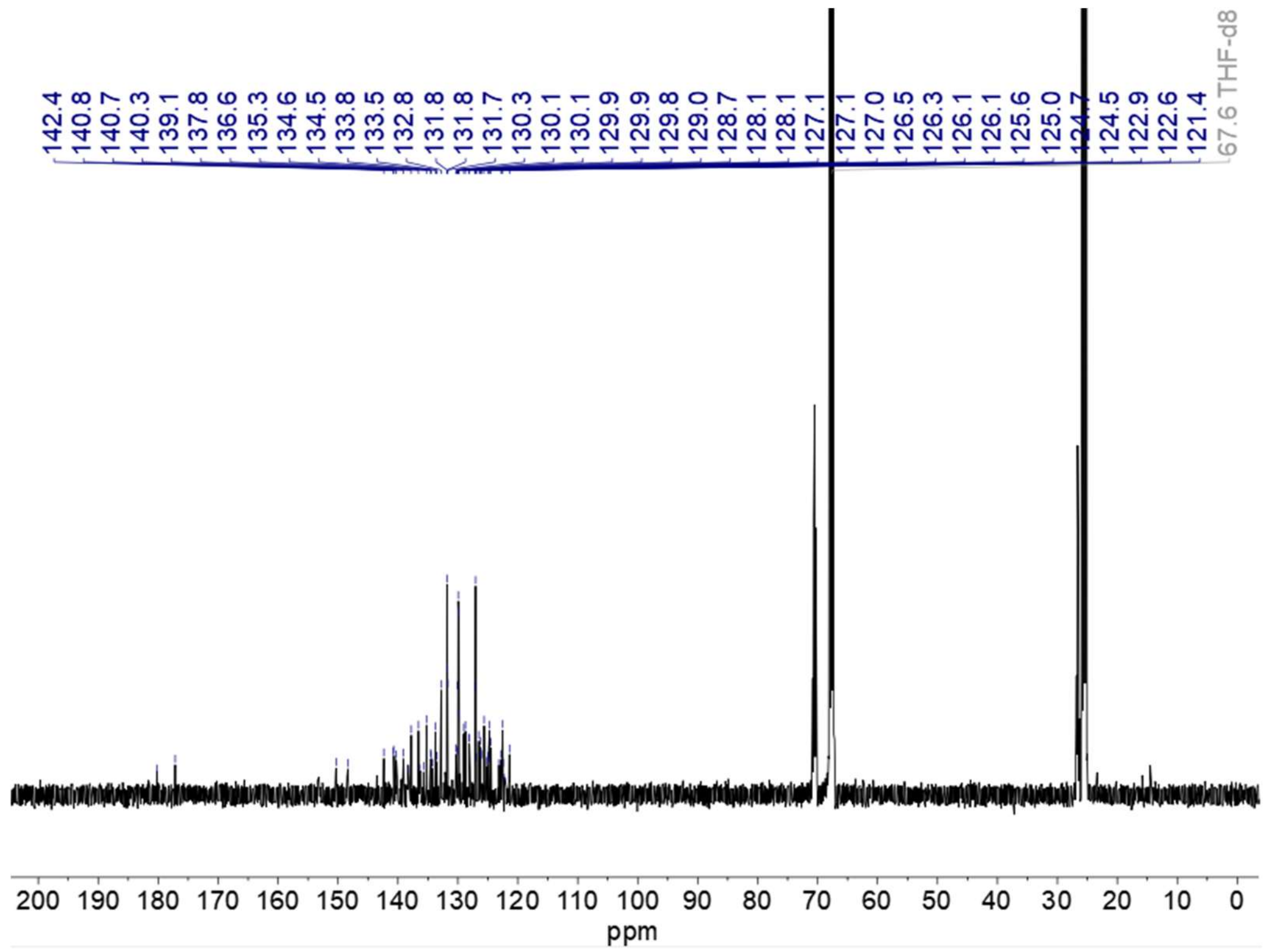

Figure S55. The ^{13}C NMR spectrum of **4a** (QPO-BtripS $[B(C_6F_5)_4]$) recorded in THF-d_8 at 298 K.

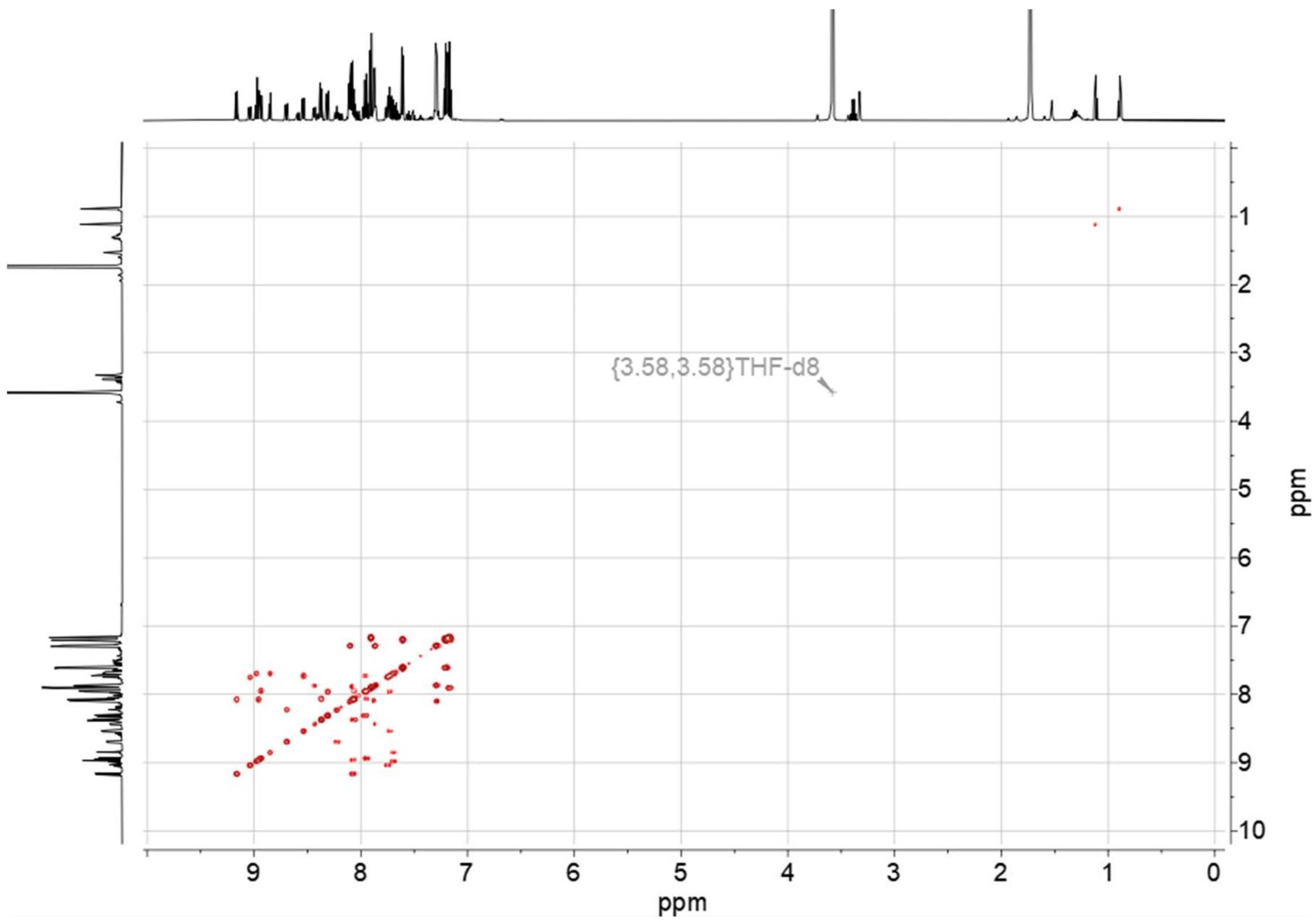

Figure S56. $^{1}H^{1}H$ COSY NMR spectrum of **4a** (QPO-BtripS $[B(C_6F_5)_4]$) recorded in THF-d_8 at 298 K.

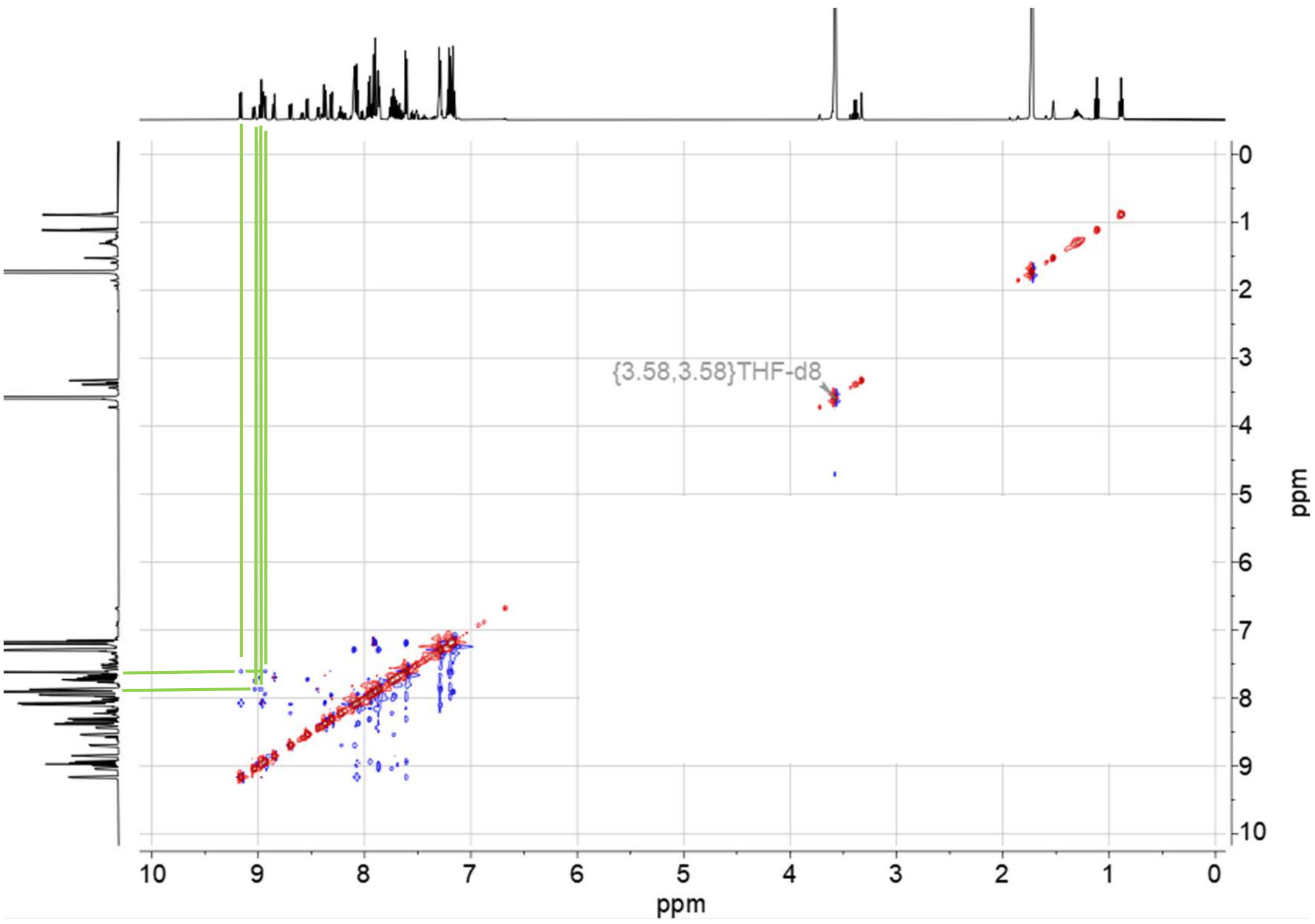

Figure S57. $^1H^1H$ NOESY NMR spectrum of **4a** (QPO-BtripS $[B(C_6F_5)_4]$) recorded in THF-d_8 at 298 K. Labelled NOE interactions (**$4a_1$ & $4a_2$**, green lines) between all 3 proximal BtripS protons (highlighted in blue) and the 2 adjacent QPO phenyl protons for both isomers.

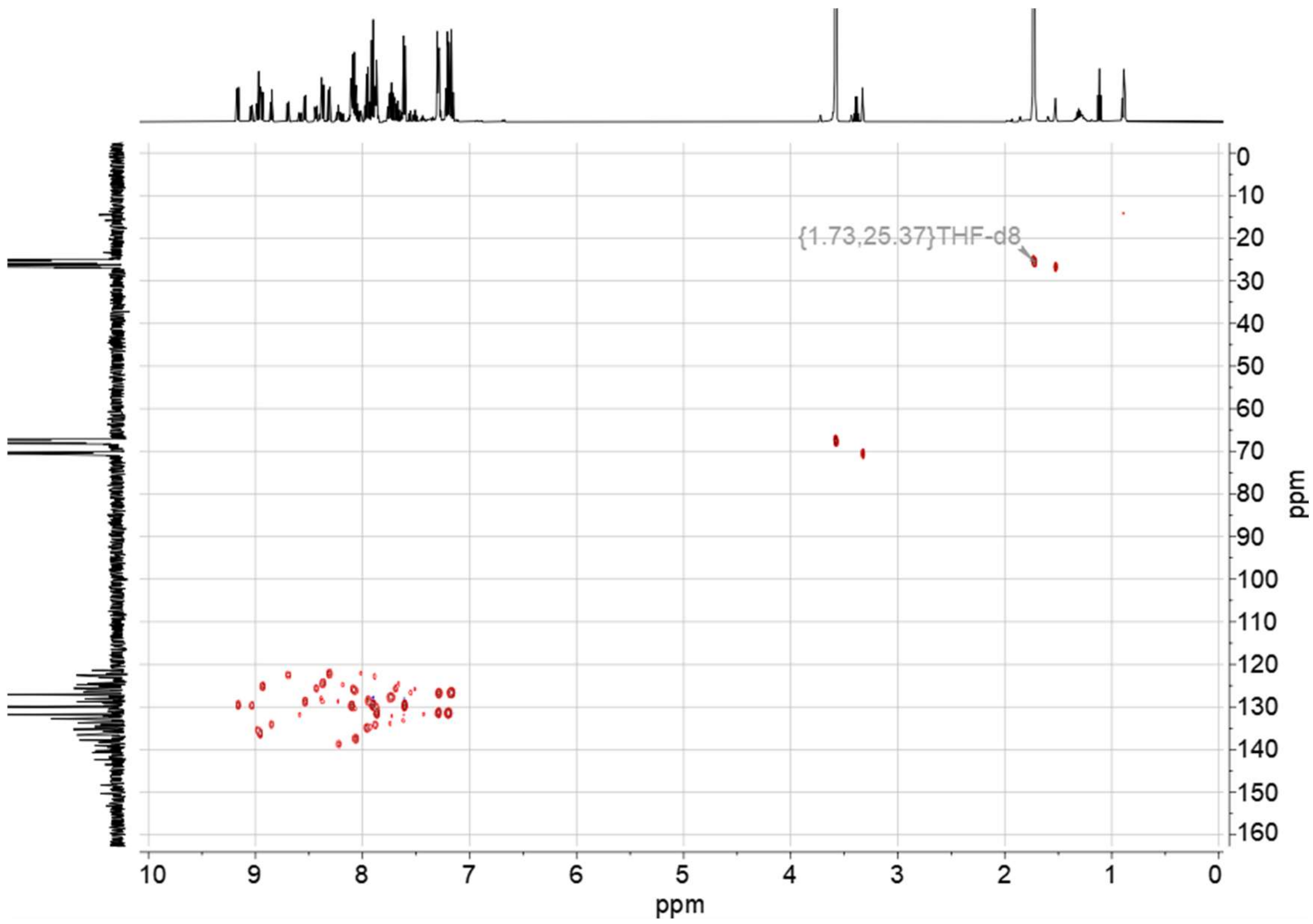

Figure S58. $^1H^{13}C$ HSQC NMR spectrum of **4a** (QPO-BtripS $[B(C_6F_5)_4]$) recorded in THF-d_8 at 298 K.

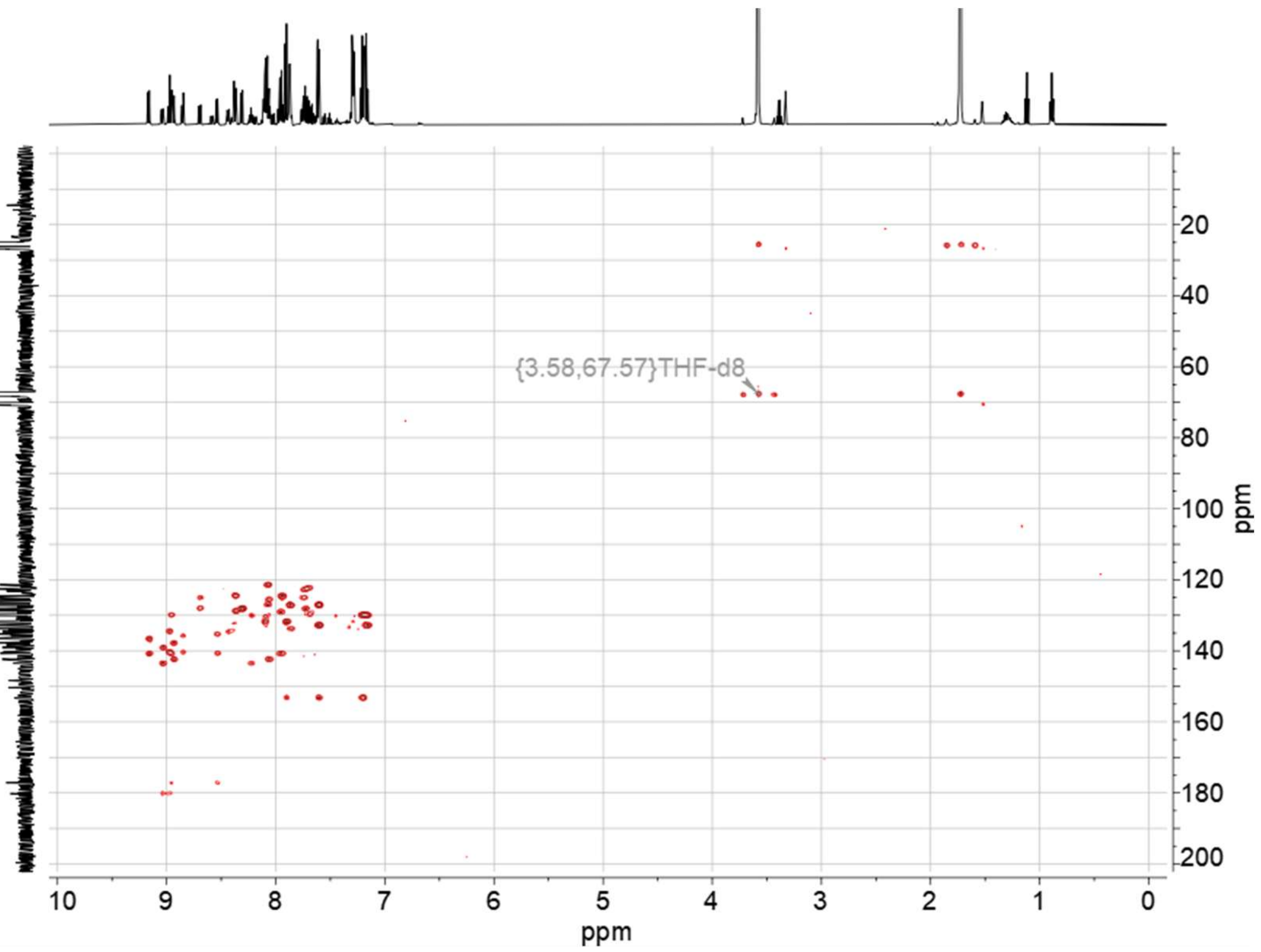

Figure S59. $^{1}H^{13}C$ HMBC NMR spectrum of **4a** (QPO-BtripS [$B(C_6F_5)_4$]) recorded in THF-d_8 at 298 K.

1.5 NMR experiments

1.5.1 Attempted adduct formation with 2 (DMAC-DPS) and BCF

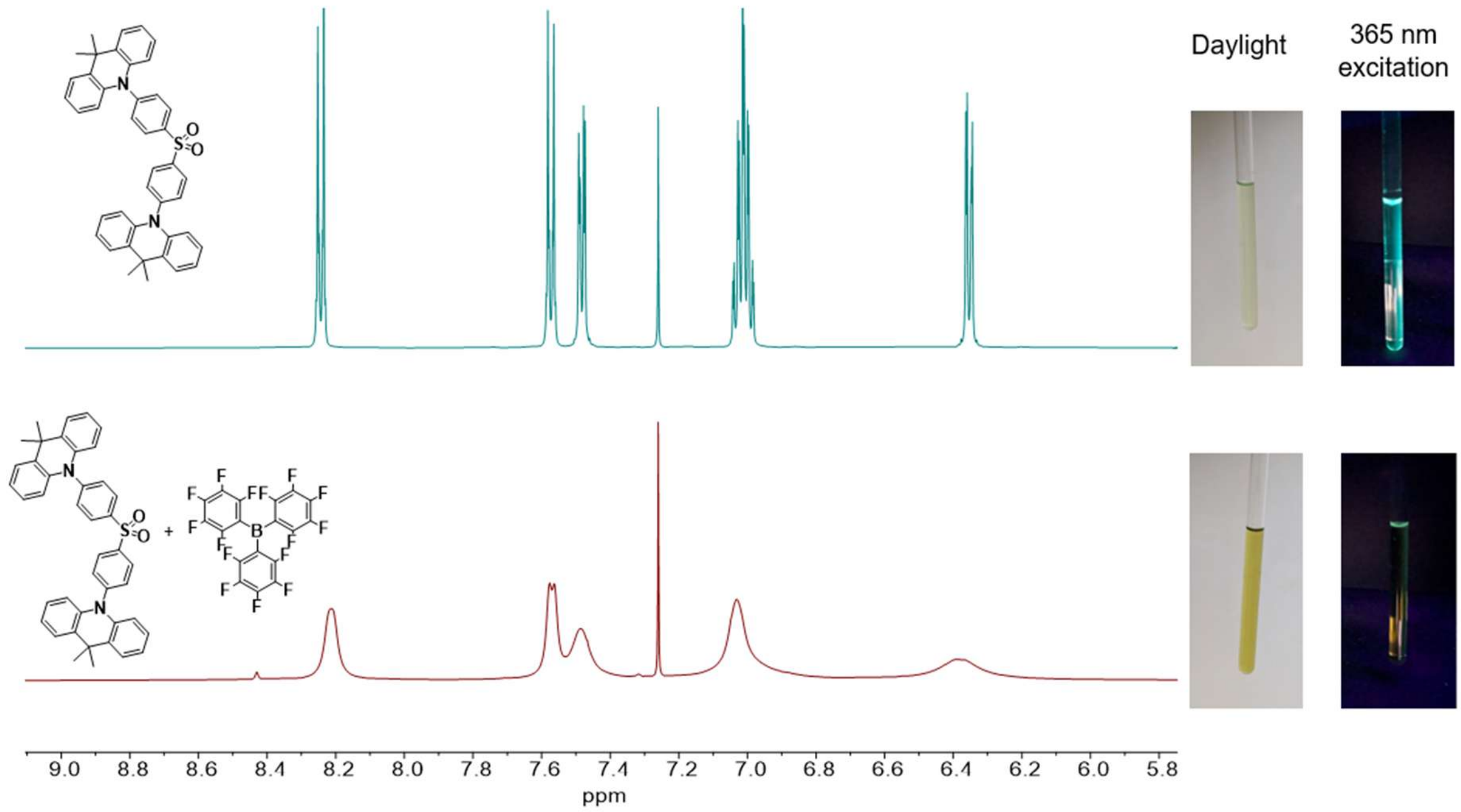

Figure S60. Stacked ^{1}H NMR spectra of **2** (DMAC-DPS, top) and **2** + 1 molar equivalent of BCF. Recorded in $CDCl_3$ at 298 K.

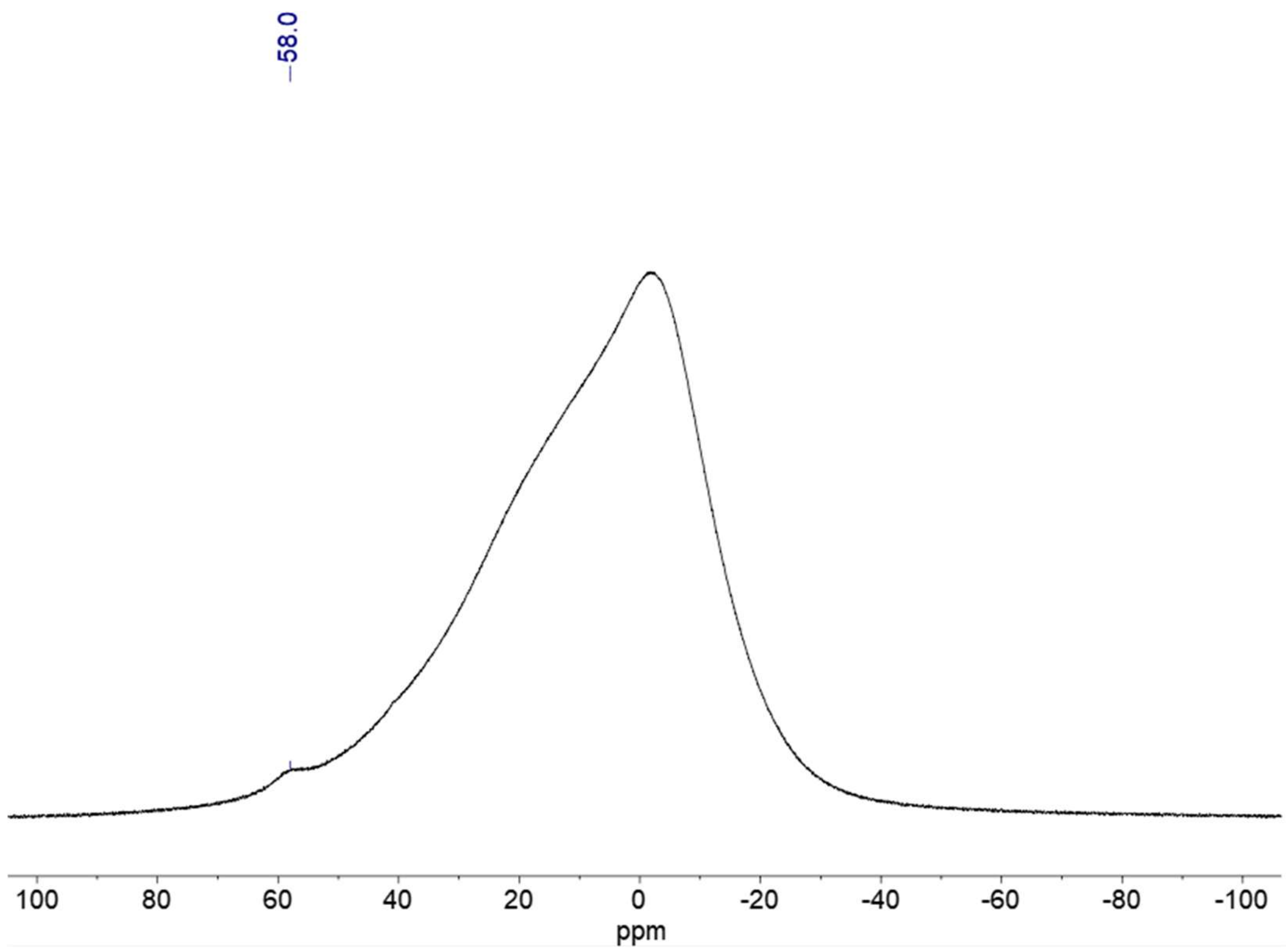

Figure S61. The in-situ ^{11}B NMR spectrum from the reaction between **2** and 1 molar equivalent of BCF, showing a signal close to that reported for free BCF at 58 ppm.[11] Recorded in $CDCl_3$ at 298 K.

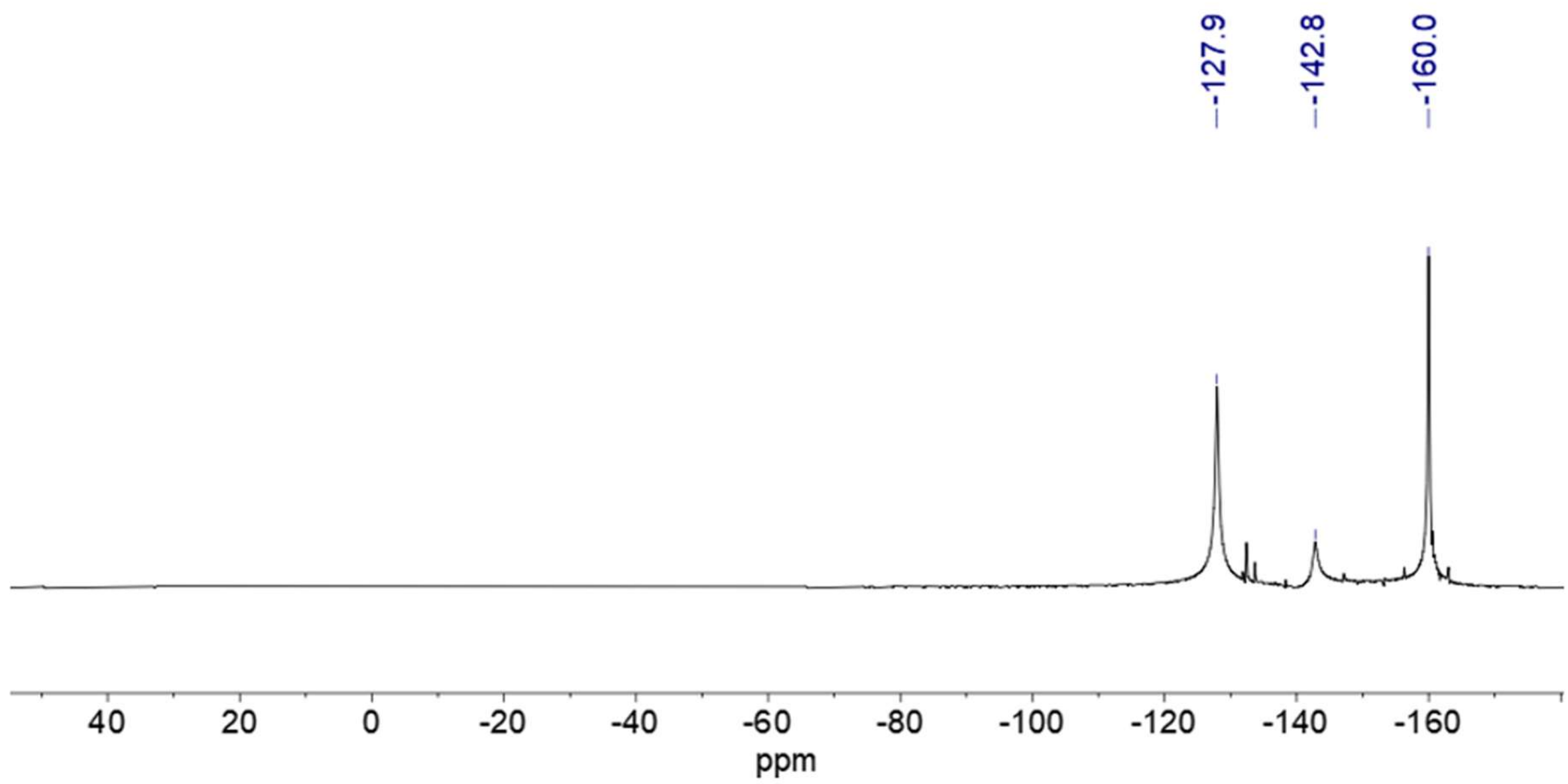

Figure S62. The in-situ ^{19}F NMR spectrum of the attempted reaction between **2** and 1 molar equivalent of BCF, showing signals close to that reported for free BCF.[11] Recorded in $CDCl_3$ at 298 K.

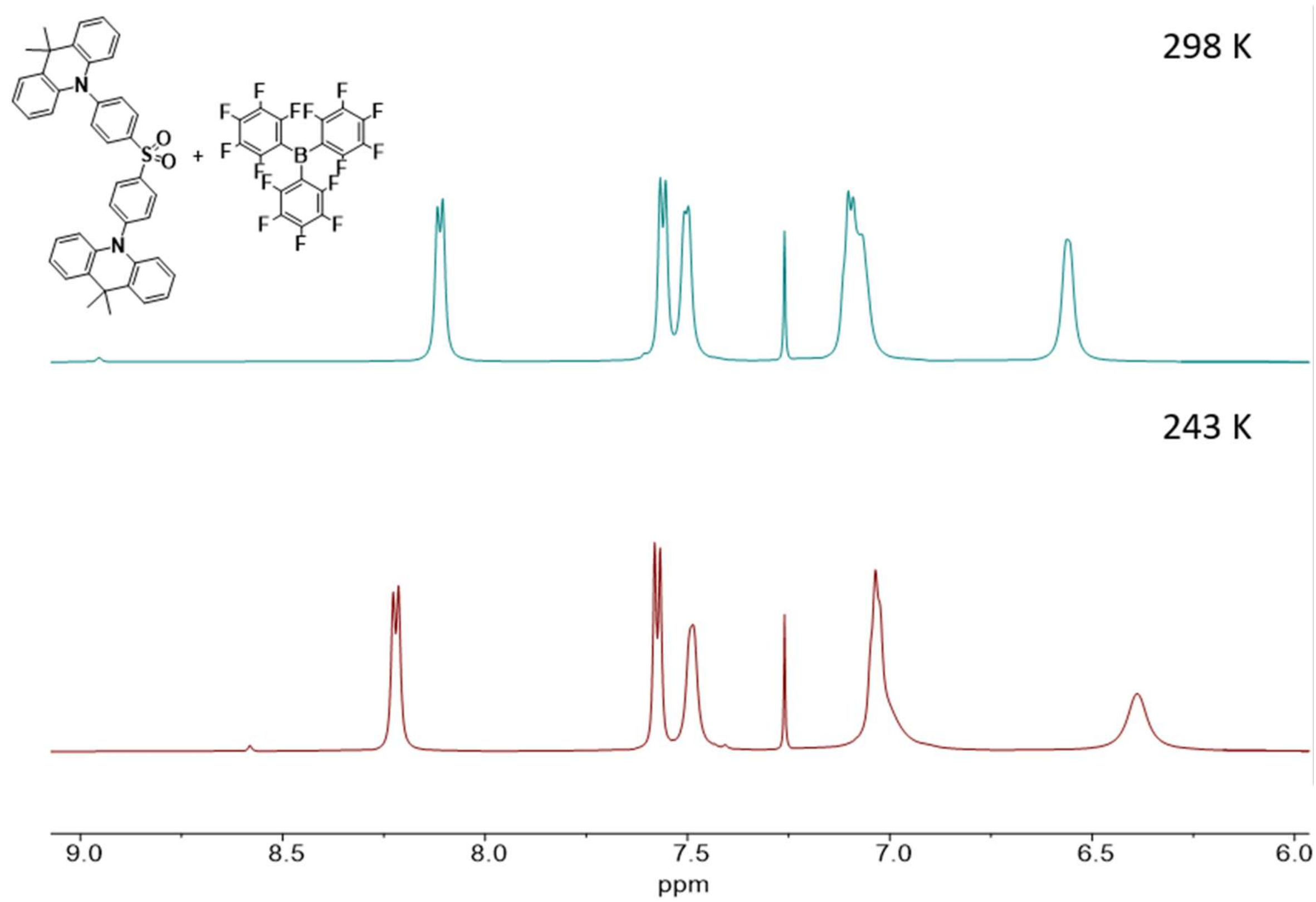

Figure S63. Stacked ^{1}H NMR spectra of **2** + 1 molar equivalent of BCF at 298 K (top) and at 243 K (bottom). Recorded in $CDCl_3$.

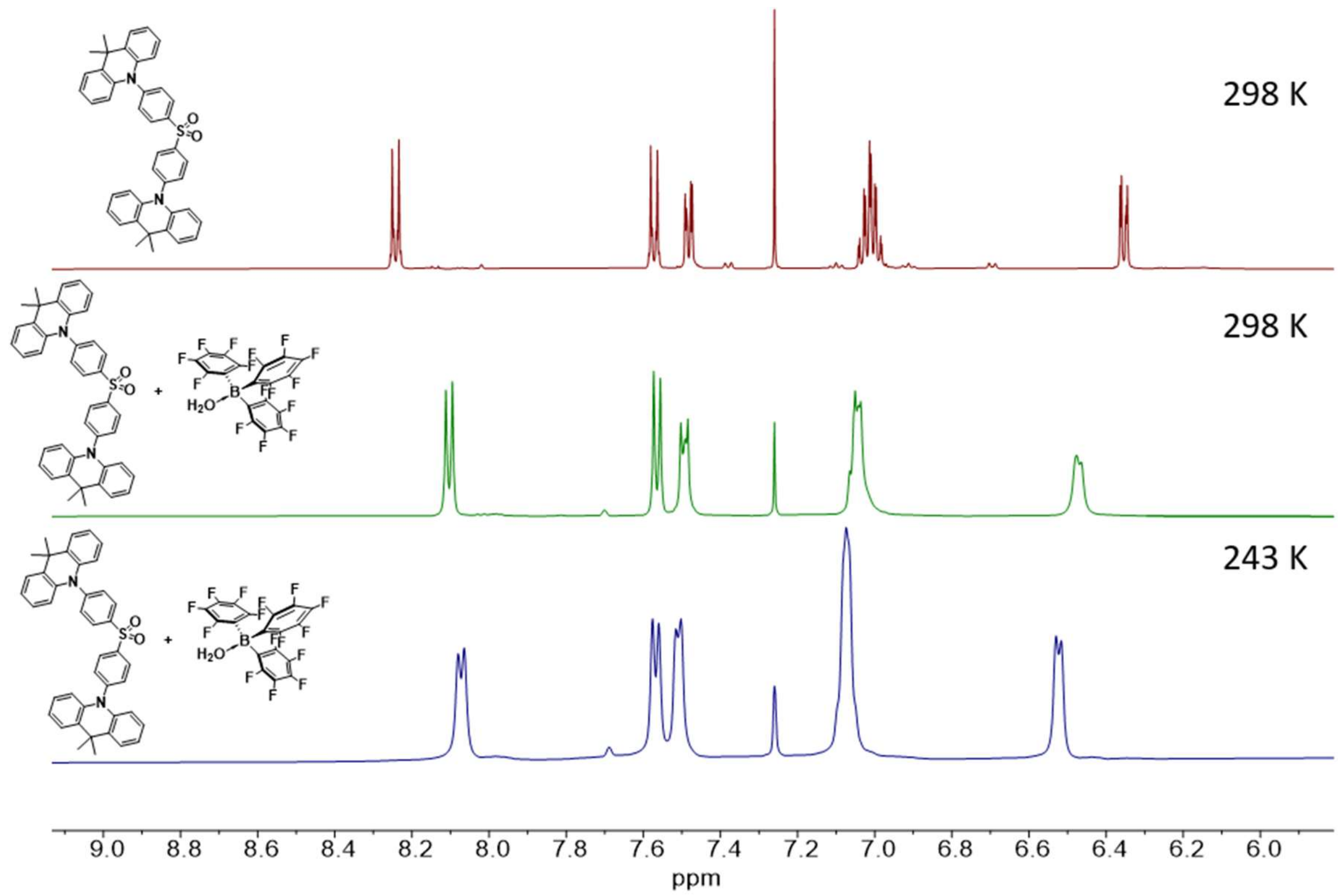

Figure S64. The ^{1}H NMR spectra of: **2** at 298 K (top), **2** + 1 molar equivalent of BCF·OH_2 at 298 K (middle), **2** + 1 molar equivalent of BCF·OH_2 at 243 K (bottom). All recorded in $CDCl_3$.

1.5.2 Low-temperature 1H NMR spectrum of 3-BCF (DiKTa-BCF)

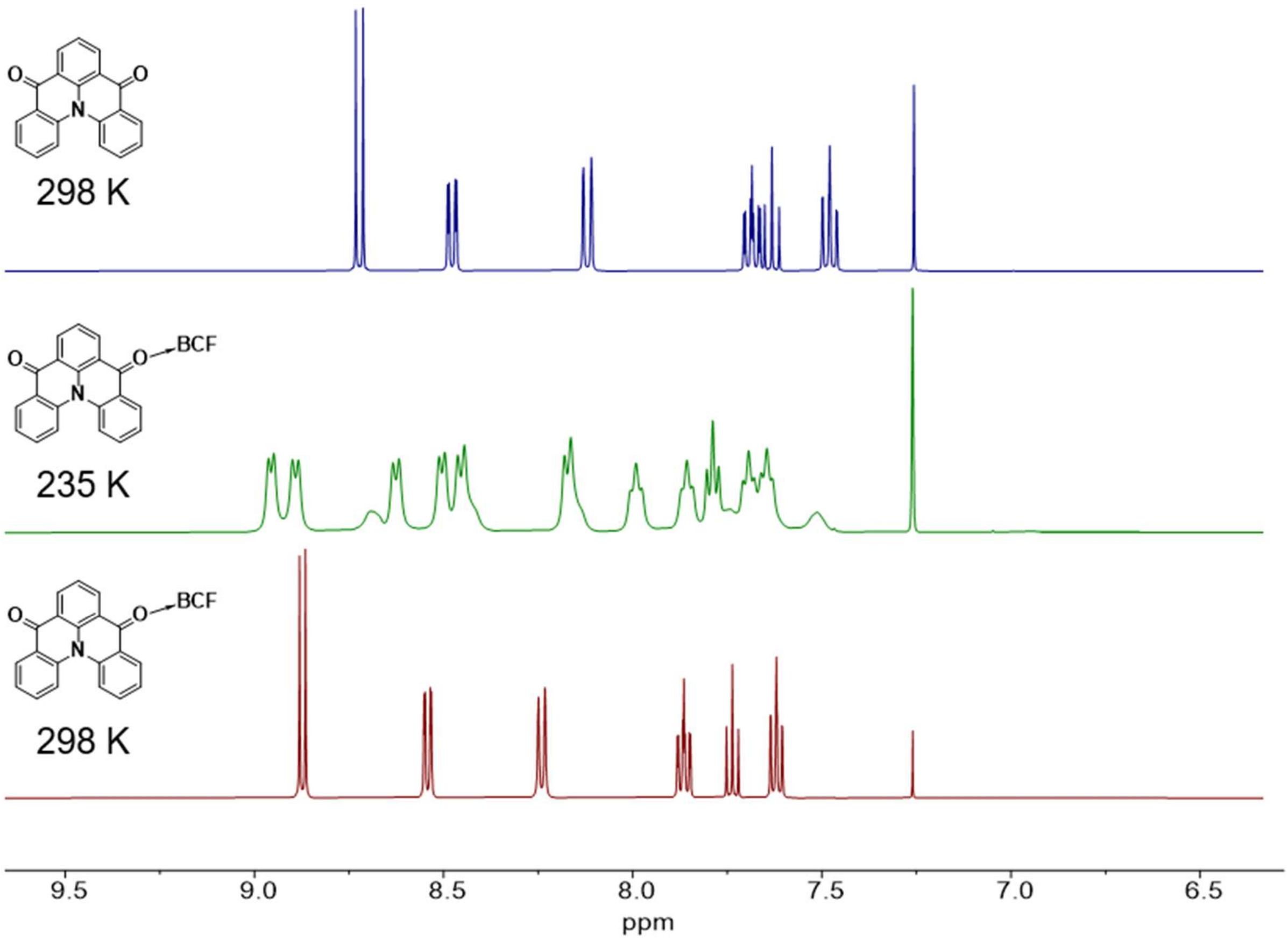

Figure S65. ^{1}H NMR spectra of **3** at 298 K (top), and **3-BCF** at 235 K (middle) and **3-BCF** at 298 K (bottom). All recorded in $CDCl_3$. Upon cooling to 235K, minor broad signals corresponding to free **3** are observed alongside **3-BCF**.

1.5.3 1a (DPA-DPS BtripS [B(C_6F_5)$_4$]) stability

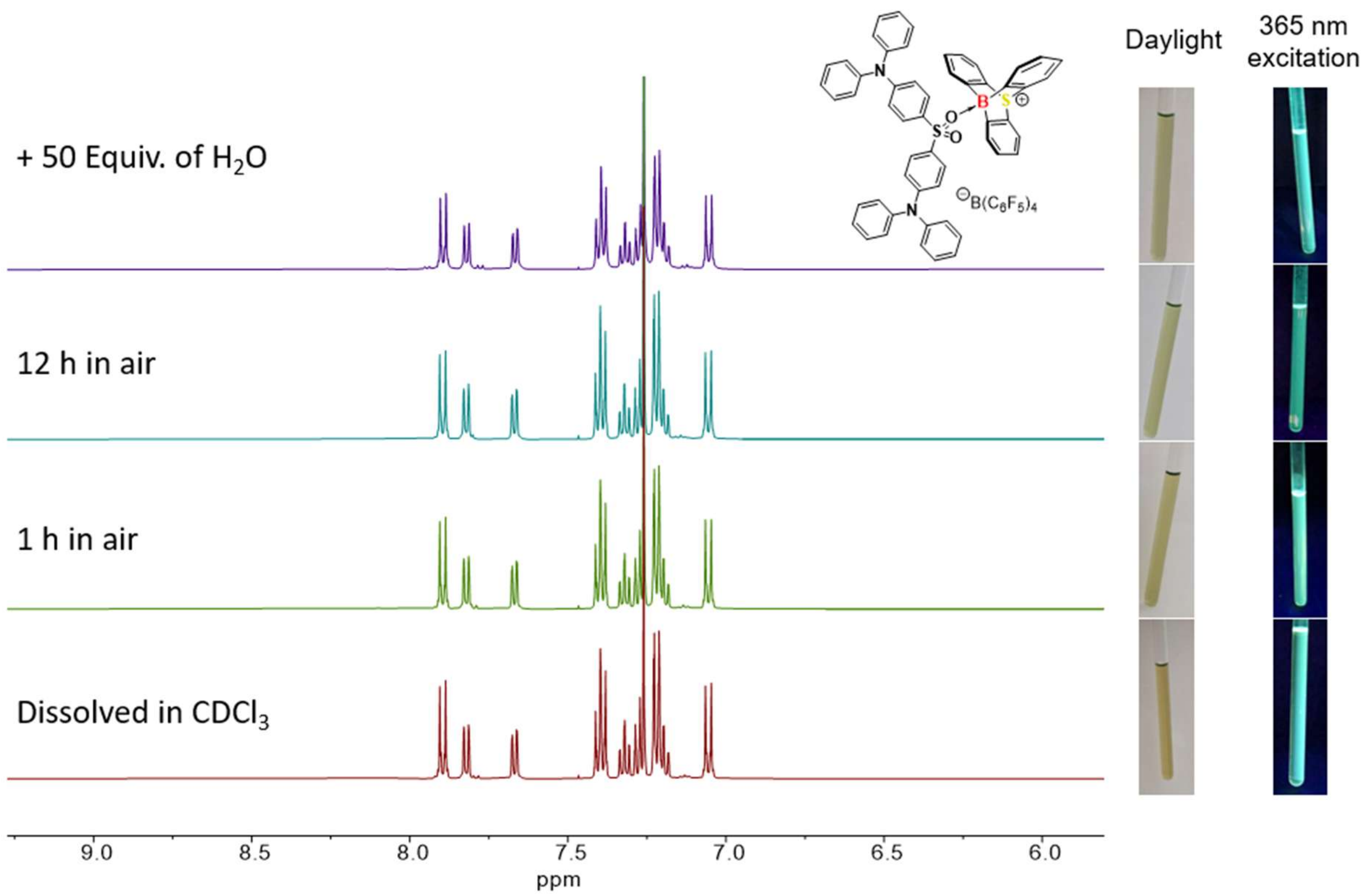

Figure S66. Stacked ^{1}H NMR spectra showing no degradation of **1a** (DPA-DPS-BtripS [B(C_6F_5)$_4$]) when exposed to ambient air and water. Recorded in $CDCl_3$ at 298 K. For the middle two spectra, the NMR sample was sparged with 2 cm^3 of air and left to stand in-air for 1 and 12 h respectively. For the top spectrum, after exposure to air for 12 hours, 50 molar equivalents of H_2O were added to the sample, and it was inverted in-air for 30 minutes before the spectrum was recorded. No visual change to the sample or decrease in fluorescence emission intensity were observed.

1.5.4 2a (DMAC-DPS BtripS [$B(C_6F_5)_4$]) stability

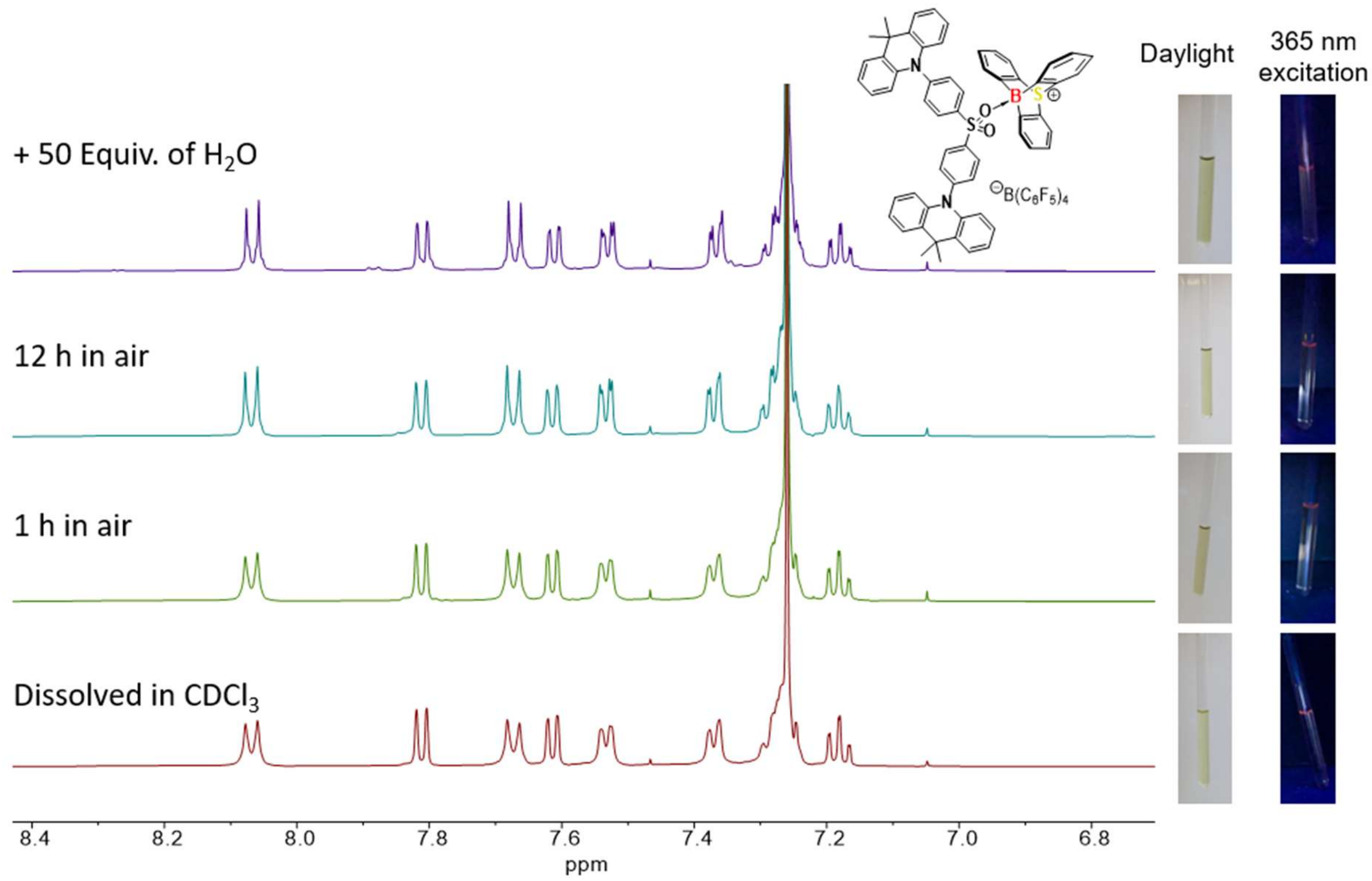

Figure S67. Stacked ^{1}H NMR spectra showing no degradation of **2a** (DMAC-DPS-BtripS [$B(C_6F_5)_4$]) when exposed to ambient air and water. Recorded in $CDCl_3$ at 298 K. For the middle two spectra, the NMR sample was sparged with 2 cm^3 of air and left to stand in-air for 1 and 12 h respectively. For the top spectrum, after exposure to air for 12 hours, 50 molar equivalents of H_2O were added to the sample, and it was inverted in-air for 30 minutes before the spectrum was recorded. No visual change to the sample or decrease in fluorescence emission intensity were observed.

1.5.5 3a (DiKTa BtripS [$B(C_6F_5)_4$]) stability

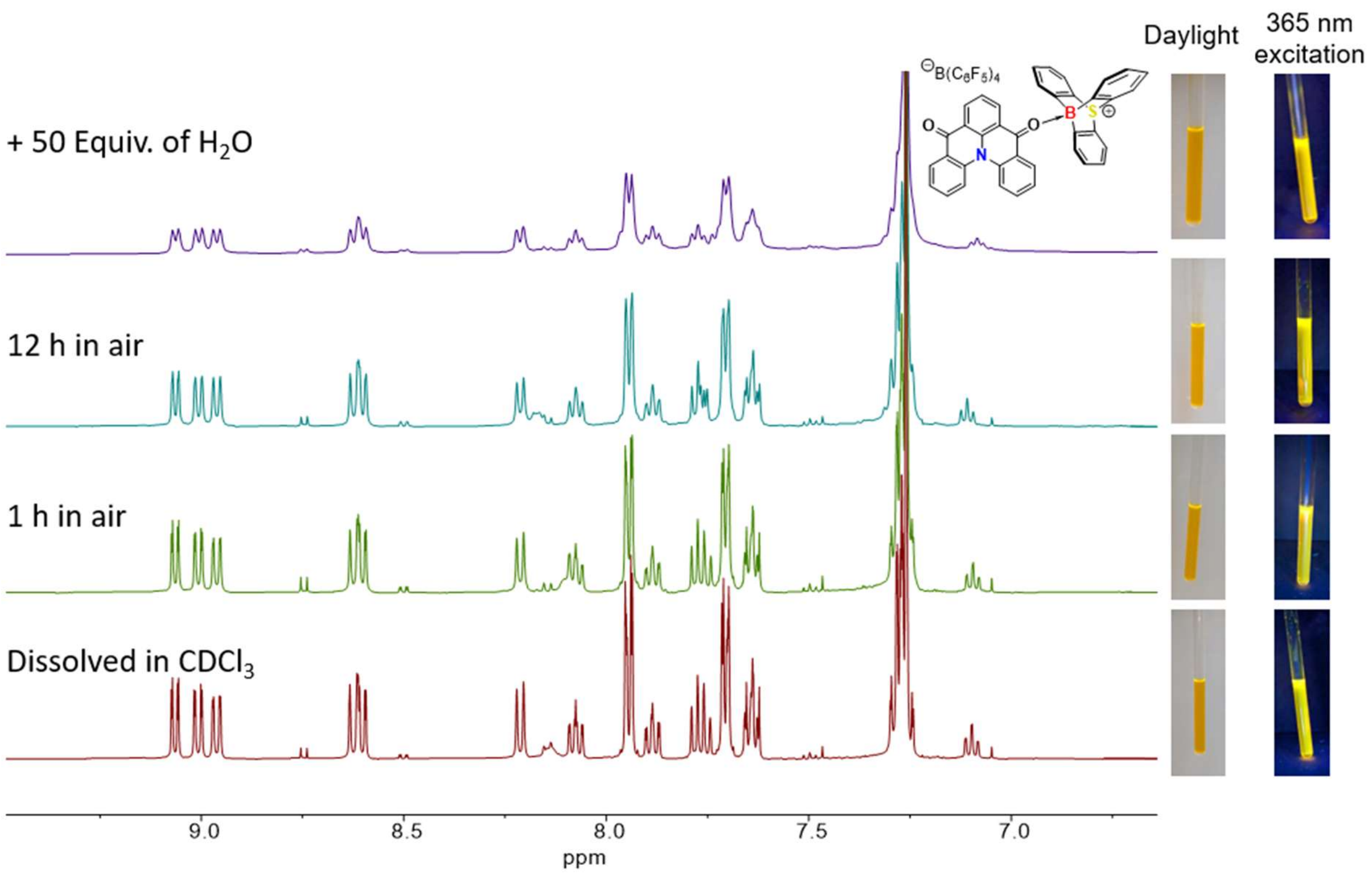

Figure S68. Stacked ^1H NMR spectra showing no degradation of **3a** (DiKTa BtripS [$B(C_6F_5)_4$]) when exposed to ambient air and water. Recorded in $CDCl_3$ at 298 K. For the middle two spectra, the NMR sample was sparged with 2 cm^3 of air and left to stand in-air for 1 and 12 h respectively. For the top spectrum, after exposure to air for 12 hours, 50 molar equivalents of H_2O were added to the sample, and it was inverted in-air for 30 minutes before the spectrum was recorded. No visual change to the sample or decrease in fluorescence emission intensity were observed.

1.5.6 3-BCF (DiKTa-BCF) stability

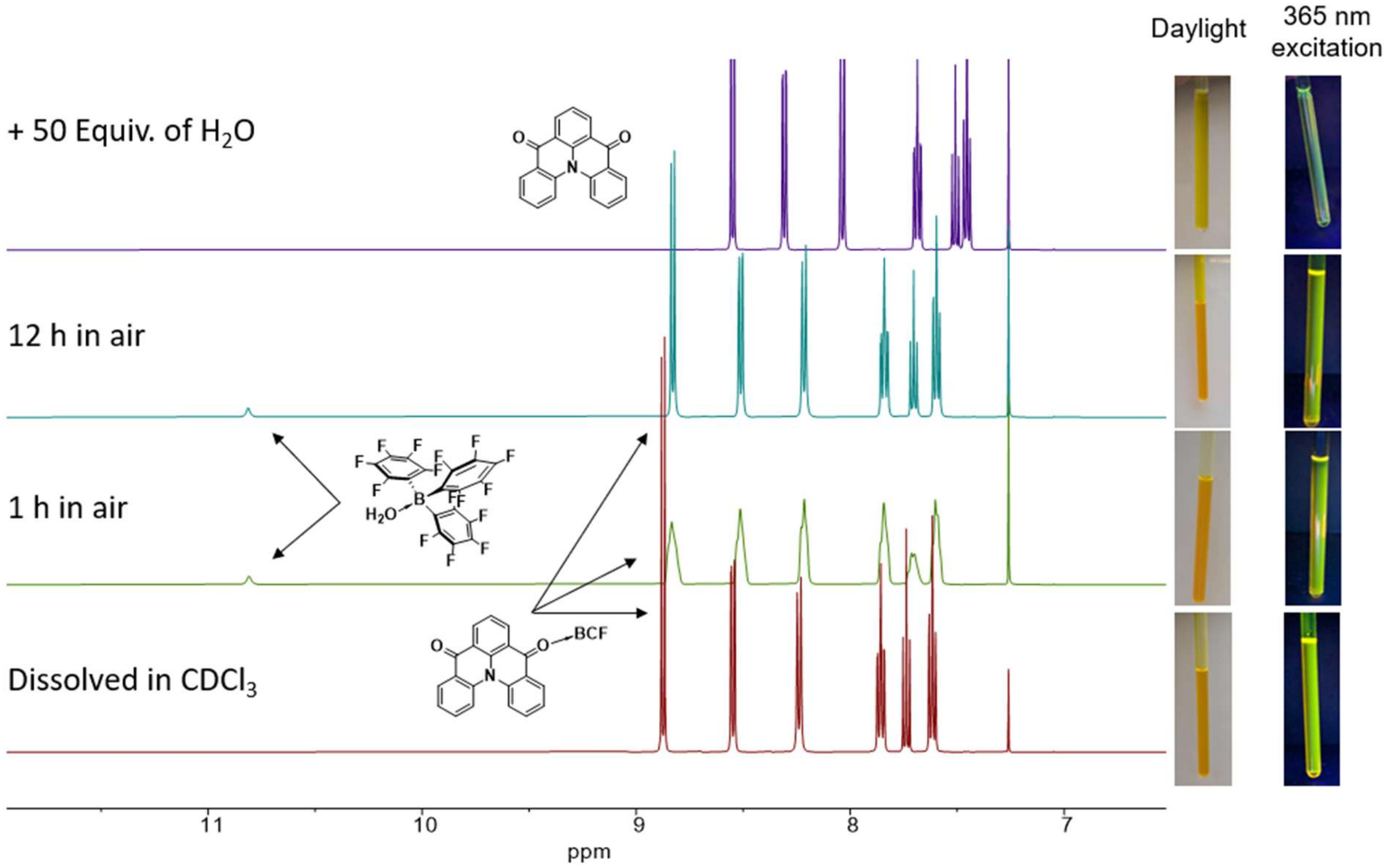

Figure S69. Stacked ^{1}H NMR spectra showing the degradation of **3-BCF** (DiKTa-BCF) when exposed to ambient air and water. Recorded in $CDCl_3$ at 298 K. For the middle two spectra, the NMR sample was sparged with 2 cm^3 of air and left to stand in-air for 1 and 12 h respectively. For the top spectrum, after exposure to air for 12 hours, 50 molar equivalents of H_2O were added to the sample, and it was inverted in-air for 30 minutes before the spectrum was recorded. Decrease in fluorescence emission intensity can be observed concurrently with BCF dissociation to form free DiKTa and the H_2O adduct of BCF (10.8 ppm).[12]

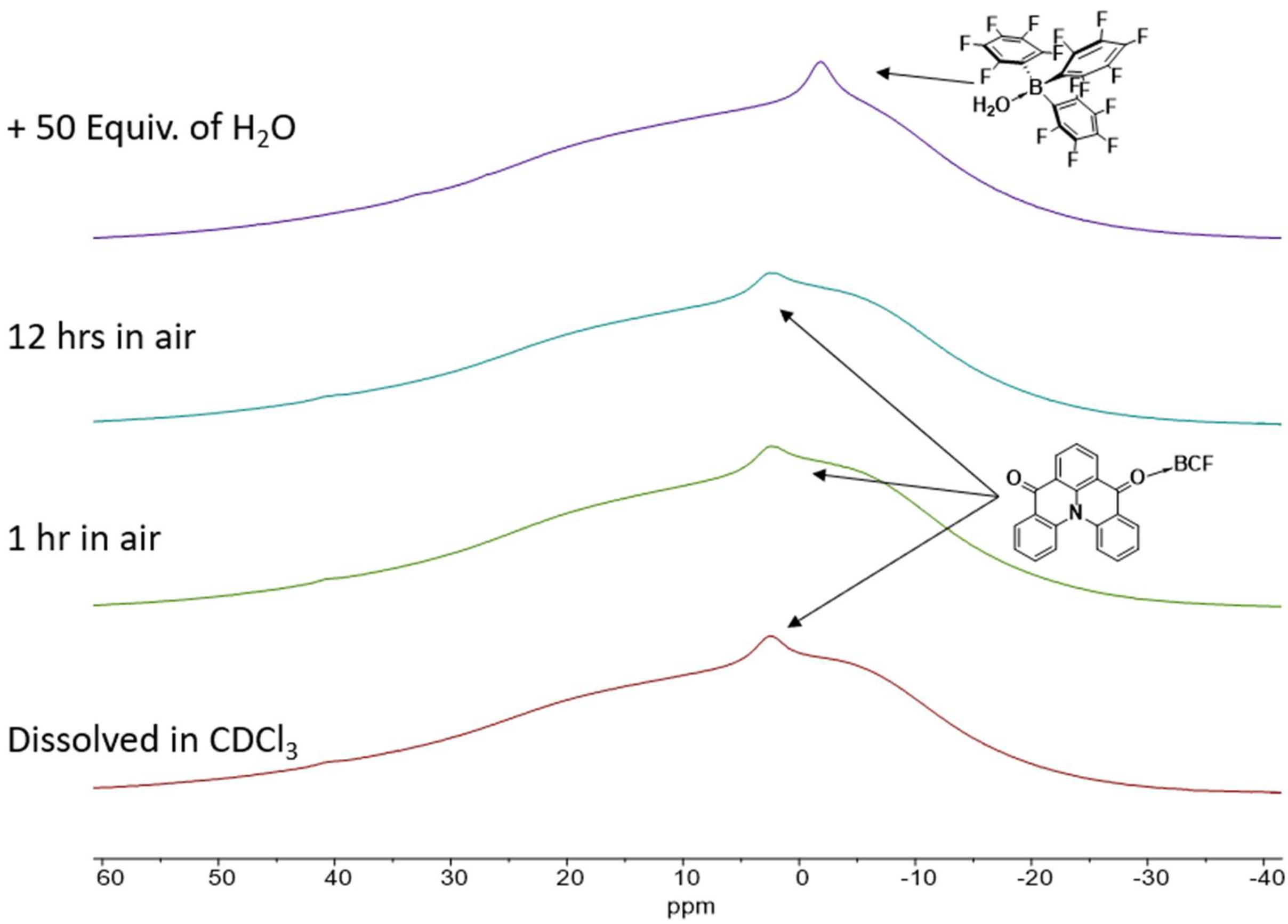

Figure S70. Stacked ^{11}B NMR spectra showing the degradation of **3-BCF** (DiKTa-BCF) when exposed to ambient air and water. Recorded in $CDCl_3$ at 298 K. For the middle two spectra, the NMR sample was sparged with 2 cm^3 of air and left to stand in-air for 1 and 12 h respectively. For the top spectrum, after exposure to air for 12 hours, 50 molar equivalents of H_2O were added to the sample, and it was inverted in-air for 30 minutes before the spectrum was recorded. The dissociation of **3-BCF** (+2 ppm) to form the H_2O adduct of BCF (– 2 ppm).[13]

1.5.7 4a (QPO BtripS [$B(C_6F_5)_4$]) stability

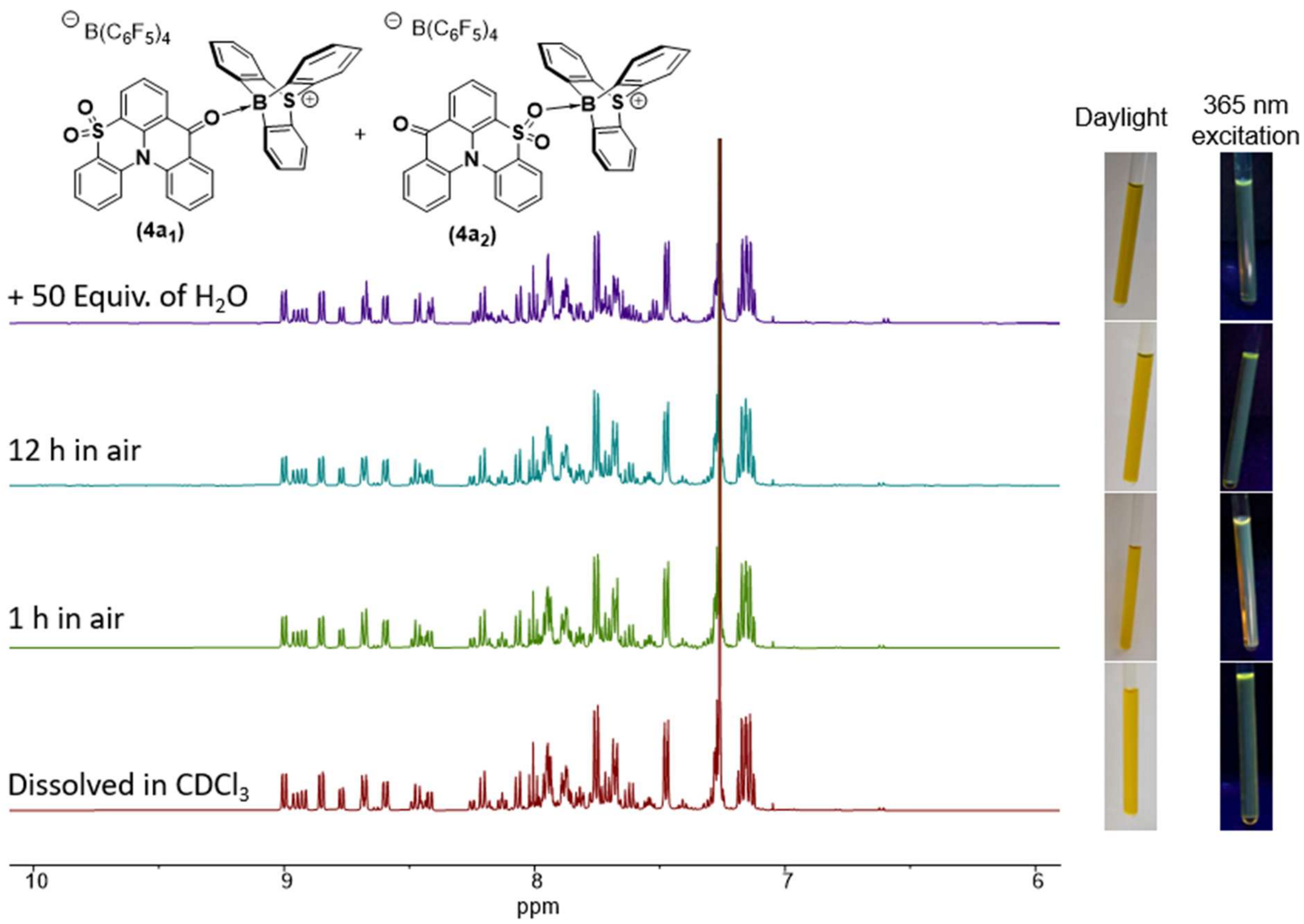

Figure S71. Stacked ^{1}H NMR spectra showing no degradation of **4a** (QPO-BtripS [$B(C_6F_5)_4$]) when exposed to ambient air and water. Recorded in $CDCl_3$ at 298 K. For the middle two spectra, the NMR sample was sparged with 2 cm^3 of air and left to stand in-air for 1 and 12 h respectively. For the top spectrum, after exposure to air for 12 hours, 50 molar equivalents of H_2O were added to the sample, and it was inverted in-air for 30 minutes before the spectrum was recorded. No visual change to the sample or decrease in fluorescence emission intensity were observed.

1.5.8 A comparison of Lewis vs Brønsted acid effects on DiKTa (3-BCF)

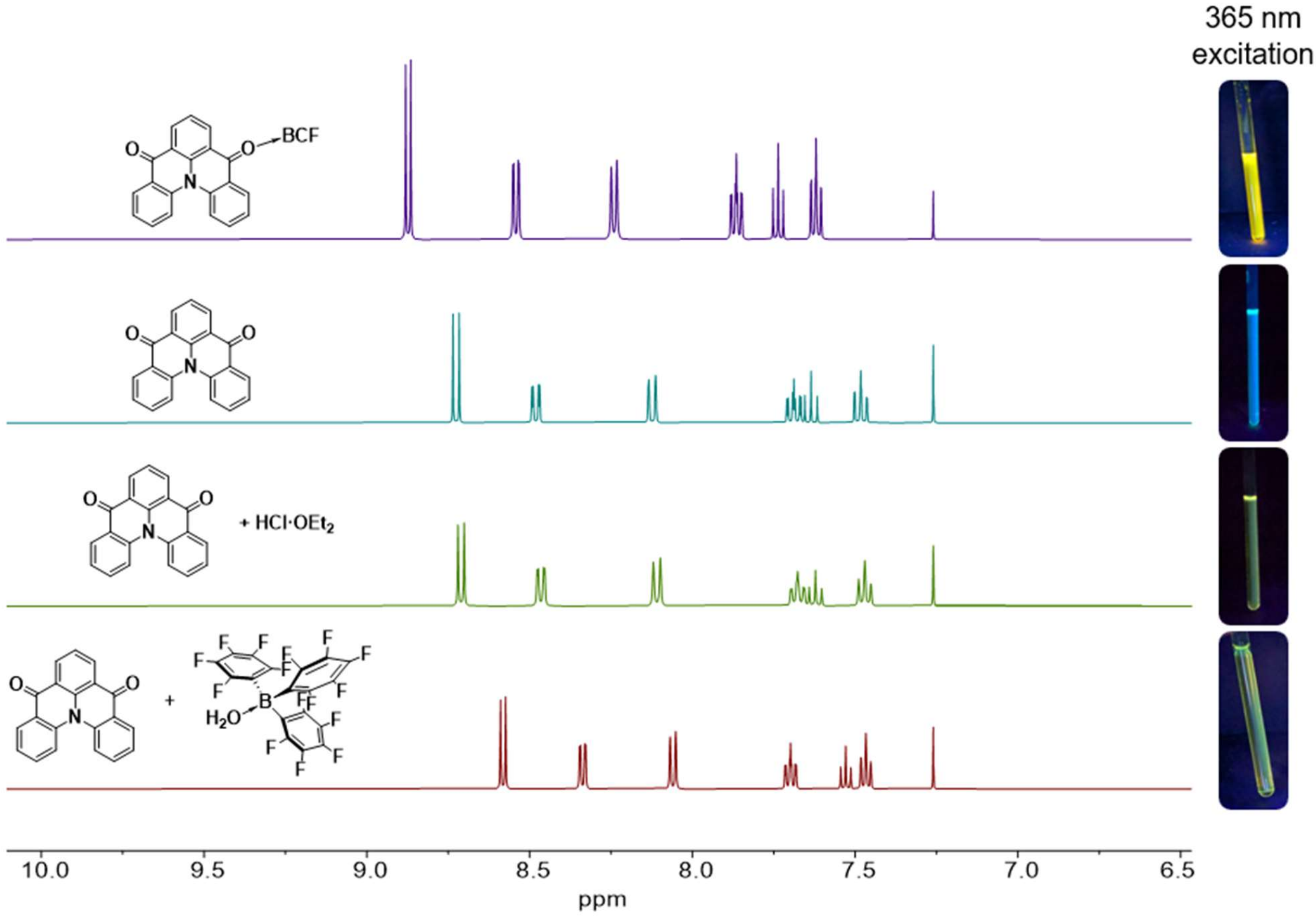

Figure S72. ^{1}H NMR spectra of (from top to bottom) DiKTa-BCF (**3-BCF**), DiKTa (**3**), DiKTa + 1 molar equivalent of HCl·OEt$_2$ and DiKTa + 1 molar equivalent of H_2O·BCF. All recorded in CDCl$_3$ at 298 K. Lewis acidic BCF induces down-field shifts of resonances whereas Brønsted acids HCl and H_2O·BCF induce up-field shifts.

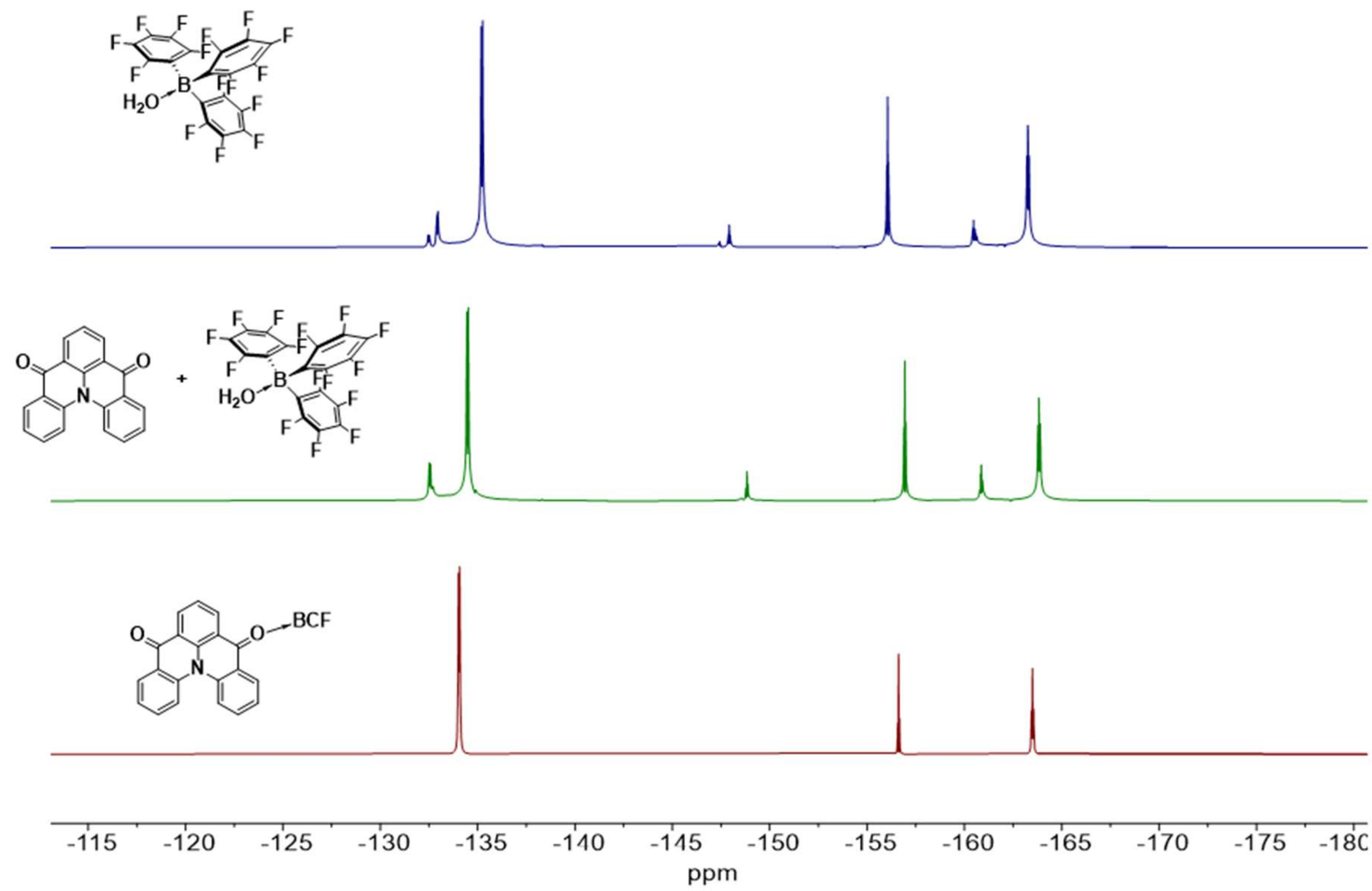

Figure S73. ^{19}F NMR spectra of H_2O·BCF (top), DiKTa + 1 molar equivalent of H_2O·BCF (middle) and DiKTa-BCF (**3-BCF**) (bottom).[14] The trace impurity in the top two spectra corresponds to $F_5C_6B(OH)_2$ present in commercial BCF (~25% by integral).[15] All recorded in $CDCl_3$ at 298 K.

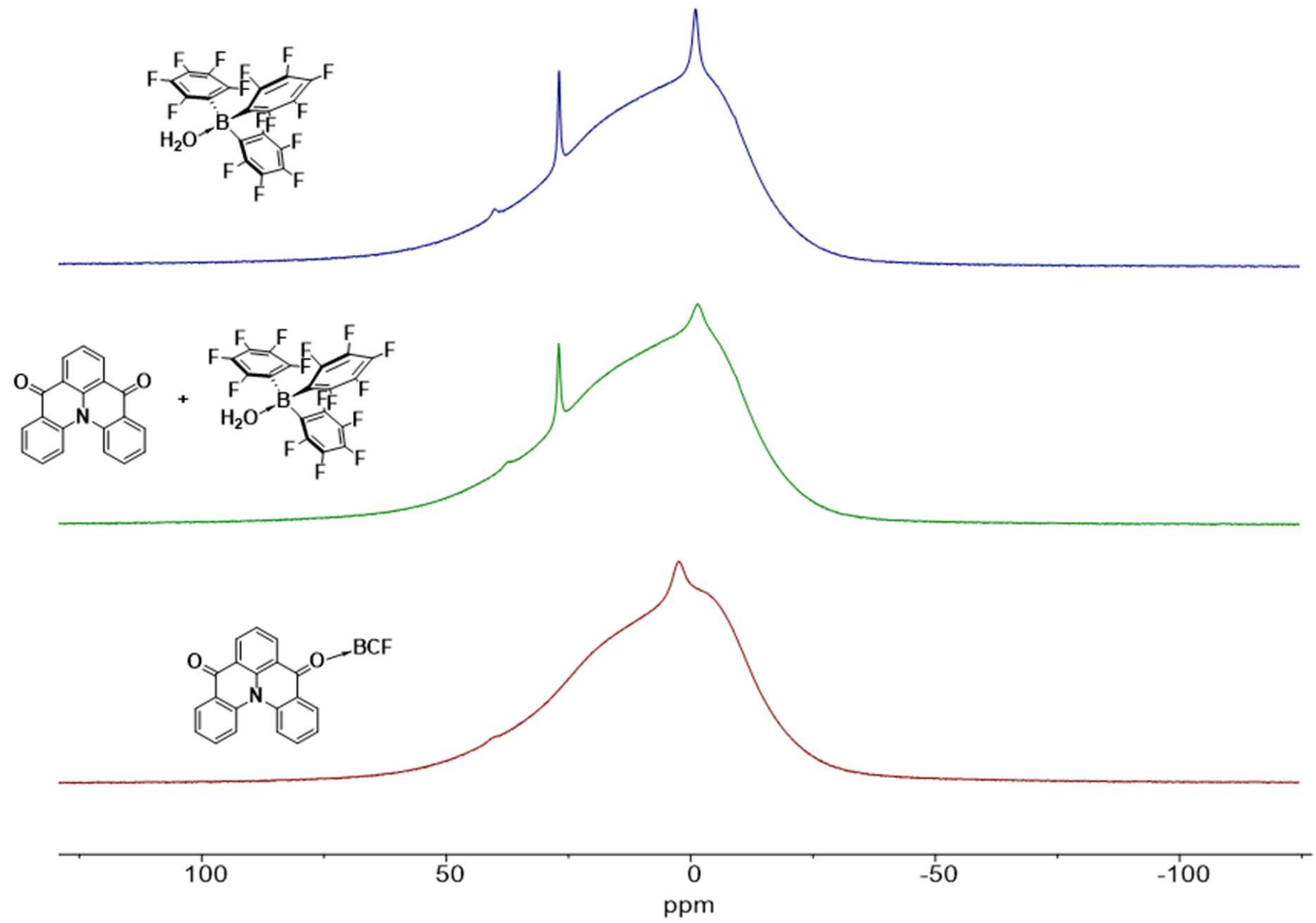

Figure S74. ^{11}B NMR spectra of $H_2O{\cdot}BCF$ (top), DiKTa + 1 molar equivalent of $H_2O{\cdot}BCF$ (middle) and DiKTa-BCF (**3-BCF**) (bottom). The major impurity in the top two spectra at 27 ppm corresponds to $F_5C_6B(OH)_2$ present in commercial BCF.[15] All recorded in $CDCl_3$ at 298 K.

1.6. Crystallography

All crystallographic data was collected on a XtaLAB Synergy-S diffractometer with a HyPix-Arc 100 detector at the University of Edinburgh. Data collections were recorded at *T* = 100.0(10) K using an Oxford Cryosystems Cryostream 1000 utilising liquid nitrogen boiloff.[16] Diffraction images from raw frame data were reduced using CryAlisPro. Structures were solved using SHELXT and refined to convergence using SHELXL through the OLEX2 GUI using full matrix least squares minimisation on F^2.[17-20] Non-hydrogen atoms were refined anisotropically, and hydrogen atoms were placed geometrically, allowed to ride upon their parent atoms. The crystallographic data is summarised in **Table S1** and full details are given in the supplementary deposited CIF file (CCDC 2531047). This can be obtained free of charge from the Cambridge Crystallographic Data Centre.

1.6.1 Crystallographic details

4a2 (QPO-BtripS [$B(C_6F_5)_4$]) crystallised with 1.5 equiv. of $CDCl_3$ (from slow evaporation crystallisation) in the asymmetric unit. This $CDCl_3$ resides within a large void in the crystal structure and exhibits significant disorder. The $CDCl_3$ containing C62 was modelled as disordered across the unit cell boundary and constrained to 0.5 occupancy. A large (2.03 e^-) residual electron density peak adjacent to this solvent molecule likely originates from further, un-modellable positional disorder of this $CDCl_3$ corresponding to ~ 10% occupancy of a Cl atom. The $CDCl_3$ containing C63 (A & B) was modelled as disordered between two positions, corresponding to freely refined occupancies of 0.9 and 0.1. SADI and DELU restraints were utilised to model both components of this disorder, and EADP was required to restrain atoms Cl2B and C63B (relating to Cl1B to and C63A respectively) to non-negative displacement parameters when modelled anisotropically.

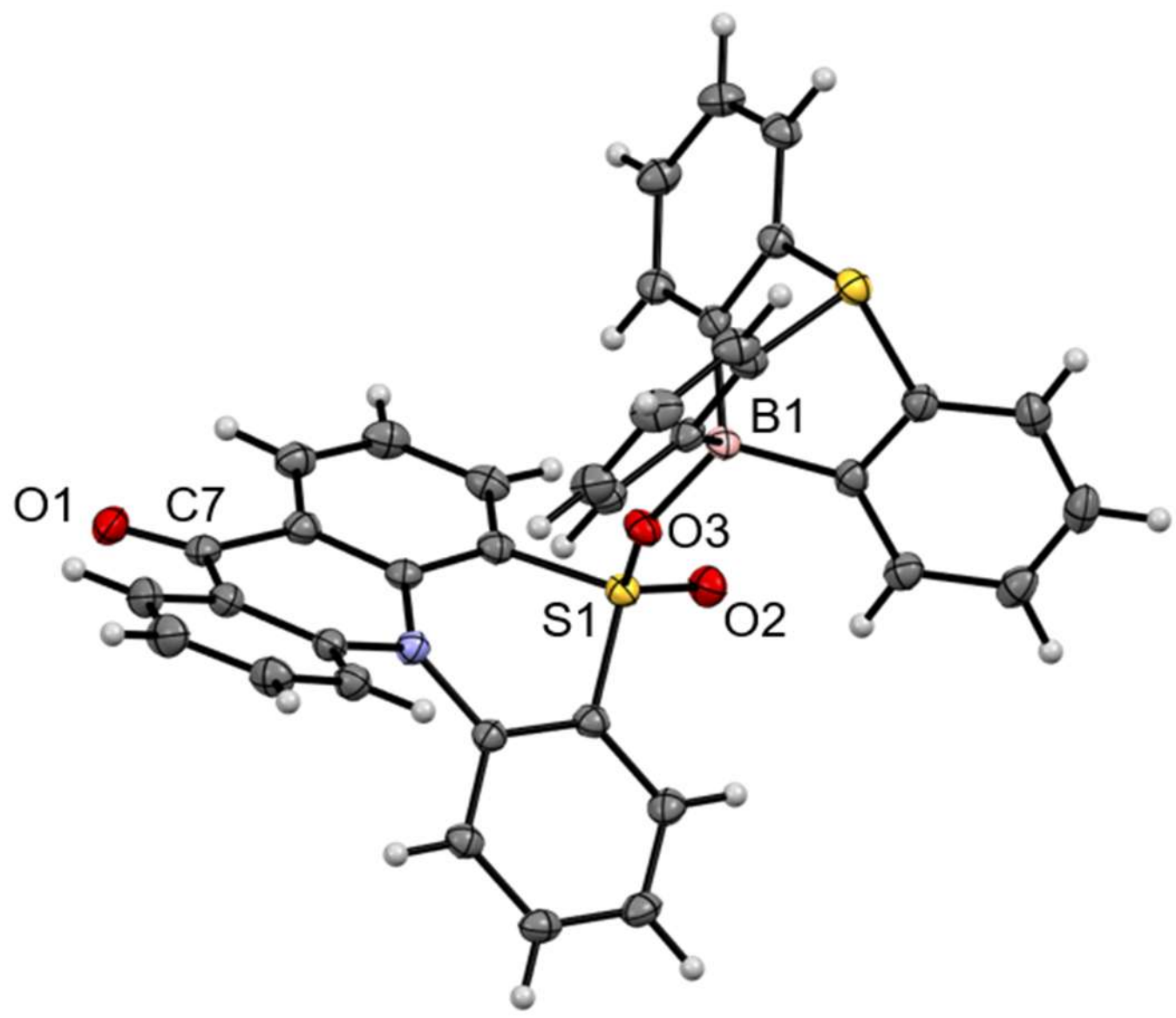

Figure S75. Molecular structure of **4a2** (QPO-BtripS [$B(C_6F_5)_4$]) determined by single crystal x-ray diffraction, showing binding of the BtripS Lewis acid to the sulfone moiety. The solvent of crystallisation ($CDCl_3$) and the [$B(C_6F_5)_4$]$^-$ anion are omitted for clarity. Selected bond lengths [Å] and angles [°]: S(1)-O(2) 1.4291(12), S(1)-O(3) 1.4910(12), B(1)-O(3) 1.562(2), C(7)-O(1) 1.227(2), O(2)-S(1)-O(3) 116.83(7), S(1)-O(3)-B(1) 142.12(11). Ellipsoids at 50% probability.

1.6.2 Crystallographic data tables

Table S1

Identification code	***4a₂***
CCDC number	2531047
Empirical formula	$C_{62.5}H_{23}B_2Cl_{4.5}D_{1.5}F_{20}NO_3S_2$
Formula weight	1464.10
Temperature/K	100.00(10)
Crystal system	triclinic
Space group	P-1
a/Å	13.1269(2)
b/Å	13.4379(2)
c/Å	17.3543(3)
α/°	90.5740(10)
β/°	102.7790(10)
γ/°	107.5120(10)
Volume/Å³	2837.41(8)
Z	2
ρ_{calc}*g/cm³*	1.714
μ/mm⁻¹	0.424
F(000)	1458.0
Crystal size/mm³	0.64 × 0.24 × 0.2
Radiation	Mo Kα (λ = 0.71073)
2Θ range for data collection/°	3.638 to 61.494
Index ranges	-18 ≤ h ≤ 18, -18 ≤ k ≤ 19, -23 ≤ l ≤ 24
Reflections collected	65307
Independent reflections	15317 [R_{int} = 0.0290, R_{sigma} = 0.0249]
Data/restraints/parameters	15317/8/899
Goodness-of-fit on F^2	1.036
Final R indexes [I>=2σ (I)]	R_1 = 0.0404, wR_2 = 0.1036
Final R indexes [all data]	R_1 = 0.0522, wR_2 = 0.1114
Largest diff. peak/hole / e $Å^{-3}$	2.03/-0.73

1.7 Photophysical characterisation

1.7.6 3-BCF (DiKTa-BCF)

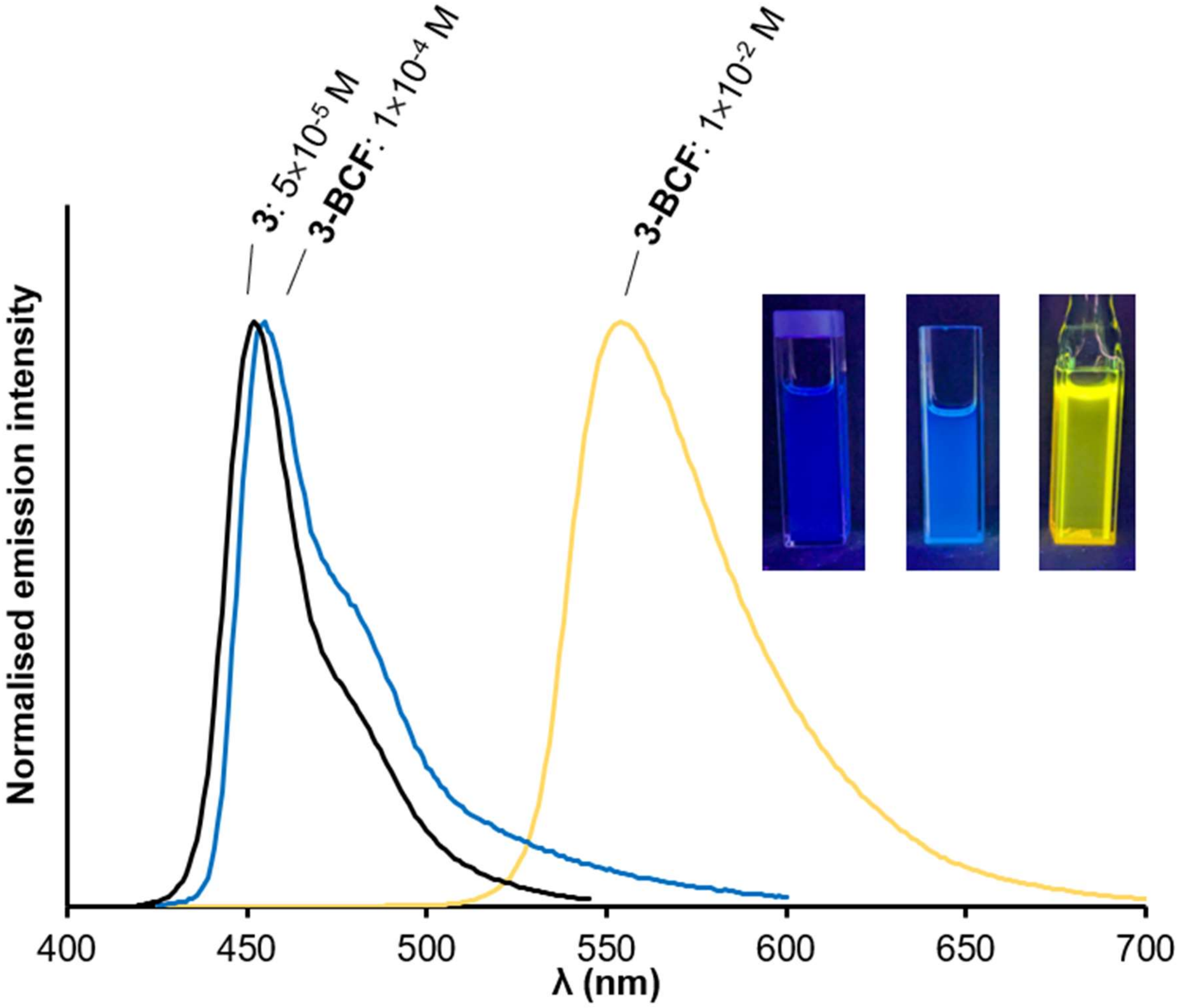

Figure S76. The intensity normalised emission spectra of **3** (DiKTa, at 5×10^{-5} M) and **3-BCF** (DiKTa-BCF, at 1×10^{-4} and 1×10^{-2} M, λ_{exc} = 290 nm). All recorded in toluene. At low concentrations, trace water present in the toluene obtained from an SPS system (~ 10 ppm, 5×10^{-4} M) induces hydrolysis of **3-BCF** to **3** and H_2O-BCF.

1.7.7 2 (DMAC-DPS) + H_2O-BCF

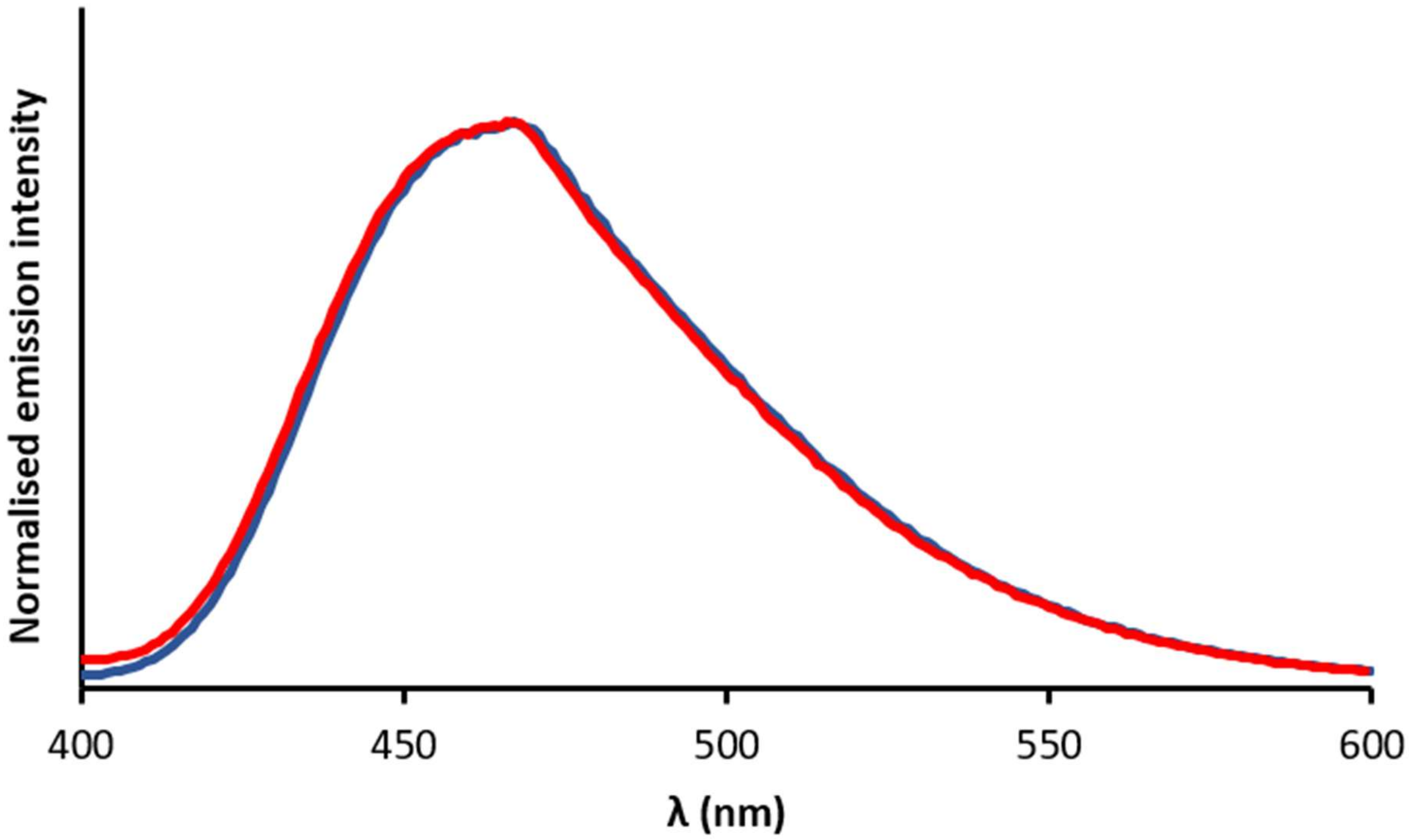

Figure S77. The emission spectra of **2** (DMAC-DPS) in the presence of 1 molar equivalent of the BCF water adduct (red) overlaid with the emission spectrum of **2** alone (blue). Both recorded in toluene at 1×10^{-5} M (λ_{exc} = 290 nm) excitation, where no shift in emission is observed.

1.7.8 BtripS adducts 1 – 4 and 1a – 4a

Table S2. Summary of recorded and reported photophysical properties.

	Solution State									1 wt% film in PMMA			
	λ_{abs} / nm [a]	ε / x 10^3 M^{-1} cm^{-1} [a]	λ_{PL} / nm [a]	FWHM / nm [a]	ΔE_{ST} / eV [b]	Φ_{PL} / % [c]	HOMO / eV [d]	LUMO / eV [d]	ΔE / eV [d]	λ_{PL} / nm	ΔE_{ST} / eV	Φ_{PL} / % [e]	τ_p (ns) / τ_d (μs)
**1**	350	22.2	400	50	0.42	54 [x]	-5.34	-	-	401	0.50	30	1.8 / -
**1a**	367	29.5	523	121	0.31	46 [x]	-5.30	-2.68	2.62	452	0.40	32	3.2 / 5546
**2**	370	2.0	466	80	-	33 [x]	-5.16	-	-	458	0.06	39	12.4 / 32.3
**2a**	425	0.5	653	173	0.01	2 [x]	-5.25	-3.05	2.20	538	-	33	12.0 / 0.998
	434	21.5	451	28	0.19	77 [y]	-	-3.05	-	468	0.20	13	3.1 / 353.7
**3a**	500	5.5	562	91	0.18	62 [z]	-	-3.90	-	553	-	20	10.1 / 13904.9
**4**	398	11.5	419	40	0.25	2 [x]	-	-2.93	-	433	0.15	6	1.9 / 824.9
**4a**	465	2.4	551	97	-	44 [z]	-	-3.92, -3.37	-	530	0.11	30	10.0 / 317.3

[a] In dilute toluene solution at 293 K. [b] Determined from the onsets of the SS PL and delayed emission spectra in 2-MeTHF at 77 K. [c] In degassed dilute toluene solution at 293 K. λ_{exc} = 340 (**1**), 360 (**1a**, **2a**), 366 (**2**), 410 (**3**), 488 (**3a**), 335 (**4**), 488 (**4a**). [d] In 0.1 M [^{n}Bu$_4$N]PF$_6$, determined using $E_{HOMO/LUMO} = -(E_{ox/red} + 4.8)$ eV, where E_{ox} and E_{red} are the onset of anodic and cathodic peak potentials, respectively, calculated from DPV vs Fc/Fc$^+$.[21] [e] Measured using an integrating sphere. [x] Quinine sulfate reference (54.6% in 0.05 M H_2SO_4, aerated). [y] $[Ru(bpy)_3]^{2+}$ reference (Φ_r = 1.8% in MeCN, aerated). [z] Rhodamine 6G reference (Φ_r = 94% in EtOH, aerated).[9]

Regarding the minor spectral broadening, this is attributed to reduced rigidity in the Lewis adduct. We attribute this is to the BTripS unit and is tentatively assigned to motion around the O---BTripS bond. This is supported by NOESY NMR experiments where there is evidence for motion involving the O---BTripS unit in the DA- and MR-TADF compounds in solution (see Fig. S17, S26, S33, and S57). In solution we have more (or lower barriers to) rotation of the large BTripS unit, thus in solution the BtripS unit can act as a rotor which is an non-radiative pathway. In the film, this motion is prevented (or has a higher barrier), resulting in improved PLQY.

The TRPL measurements reveal that **1a** and **2a** each have a monoexponential delayed PL lifetime (τ_d = 5546 and 0.998 μs, respectively). For **2a**, this represents a 40-fold decrease in delayed lifetime compared to that of parent **2** (from 38.0 μs to 0.998 μs for **2** and **2a**, respectively). The ISC and RISC rate constants (k_{ISC} and k_{RISC}, respectively) were extracted from a complete fit of the TR PL decays (Table **S3**). In both cases, k_{ISC} and k_{RISC} increase on adduct formation. Compound **2a** (1.95×10^6 s^{-1}) displays a fast k_{RISC} compared to **2** (3.35×10^4 s^{-1}), which is reflected in the decreased lifetime. These observations are consistent with the observed reduction in ΔE_{ST} for both **1a** and **2a** relative to **1** and **2**.

Table **S3**. Summary of the photophysical properties for 1 wt% **1**, **1a**, **2**, and **2a** in PMMA.

	λ_{PL} / nm [a]	E_{S1} / eV [b]	E_{T1} / eV [b]	ΔE_{ST} / eV [b]	Φ_{PL} / % [c]	τ_p (ns) [d]/ τ_d (μs) [e]	k_{ISC} / s^{-1} [f]	k_{RISC} / s^{-1} [f]	k_r^S / s^{-1} [f]	k_{nr}^S / s^{-1} [f]
**1**	401	3.40	2.90	0.50	30	1.8 / -	-	-	1.67×10^8	3.89×10^8
**1a**	452	3.18	2.78	0.40	32	3.2 / 5546	5.17×10^7	2.16×10^2	8.35×10^7	1.77×10^8
**2**	458	3.12	3.06	0.06	39	12.4 / 32.3	4.34×10^6	3.35×10^4	2.90×10^7	4.54×10^7
**2a**	538	2.82	-	-	33	12.0/ 0.998	3.95×10^7	1.95×10^6	1.42×10^7	2.88×10^7

[a] λ_{exc} = 320 nm for **2**, 335 nm for **1**, 340 nm for **1a**, and 360 nm for **2a**. [b] Determined from the onsets of the SS PL and delayed emission spectra at 77 K (λ_{exc} = 320 nm for **2**, 335 nm for **1**, 340 nm for **1a**, and 360 nm for **2a**. [c] Measured using an integrating sphere (λ_{exc} = 320 nm for **1**, **1a**, and **2**, 350 nm for **2a**). [d] Measured by TCSPC (λ_{exc} = 375 nm). [e] Measured by MCS (λ_{exc} = 320 nm for **2**, 335 nm for **1**, and 340 nm for **1a**) or TCSPC (λ_{exc} = 375 nm (**2a**)). [f] Rate constants were calculated using the steady-state approximation method as described in the literature. [22]

Comparing **3** vs. **3a** there is a significant increase in delayed lifetime τ_d (from τ_d = 353.7 for **3** to τ_d = 13,905 μs for **3a**). This is the opposite of what was observed for the DA-TADF emitters. For **3** there is a T_2 state intermediate in energy between the S_1 and T_1 states. In contrast, for **3a** there is no intermediate triplet state between the S_1 and T_1 states. The spin-orbit coupling matrix element (SOCME) values between S_1 and T_1 were determined for **3** and **3a** based on the optimised T_1 geometry, as well as between

S_1 and T_2 for **3** (see **1.8.5** TDDFT analysis for computational details). The S_1-T_1 SOCME values for **3** and **3a** are 26.99 and 0.53 cm^{-1}, respectively, implying faster ISC/RISC cycling for **3** than **3a**. Additionally, a S_1-T_2 SOCME value of 3.76 cm^{-1} was found for **3**. This indicates that RISC can also proceed *via* the intermediate T_2 state for this molecule. The k_{ISC}/k_{RISC} values for **3** and **3a** were determined to be 9.93 × 10^7/4.08 × 10^3 s^{-1} and 3.47 ×10^7/1.11 × 10^2 s^{-1}, respectively (Table **S4**). In this case, adduct formation resulted in slower ISC and RISC, which is consistent with the calculations and is attributed to the combination of a reduced SOC between S_1 and T_1 in **3a** and the absence of an intermediate triplet state in **3a**.

Table **S4**. Summary of photophysical properties of 1 wt% **3** and **3a** in PMMA.

	λ_{PL} / nm [a]	E_{S1} / eV [b]	E_{T1} / eV [b]	ΔE_{ST} / eV [b]	Φ_{PL} / % [c]	τ_p (ns) [d] /t_d (μs) [e]	k_{ISC} / s^{-1} [f]	k_{RISC} / s^{-1} [f]	k_r^S / s^{-1}	k_{nr}^S / s^{-1}
**3**	468	2.72	2.52	0.20	13	3.1 / 353.7	9.93 × 10^7	4.08 × 10^3	2.90 × 10^7	1.94 × 10^8
**3a**	553	2.42	-	-	20	10.1 / 13904.9	3.47 × 10^7	1.11 × 10^2	1.29 × 10^7	5.15 × 10^7

[a] λ_{exc} = 330 nm for **3**, 340 nm for **3a**. [b] Determined from the onsets of the SS PL and delayed emission spectra at 77 K (λ_{exc} = 330 nm for **3**, 340 nm for **3a**. [c] Measured using an integrating sphere (λ_{exc} = 330 nm for **3**, 480 nm for **3a**. [d] Measured by TCSPC (λ_{exc} = 375 nm). [e] Measured by MCS (λ_{exc} = 330 nm for **3**, 340 nm for **3a**). [f] ISC and RISC rate constants were calculated using the steady-state approximation method as described in the literature.[22]

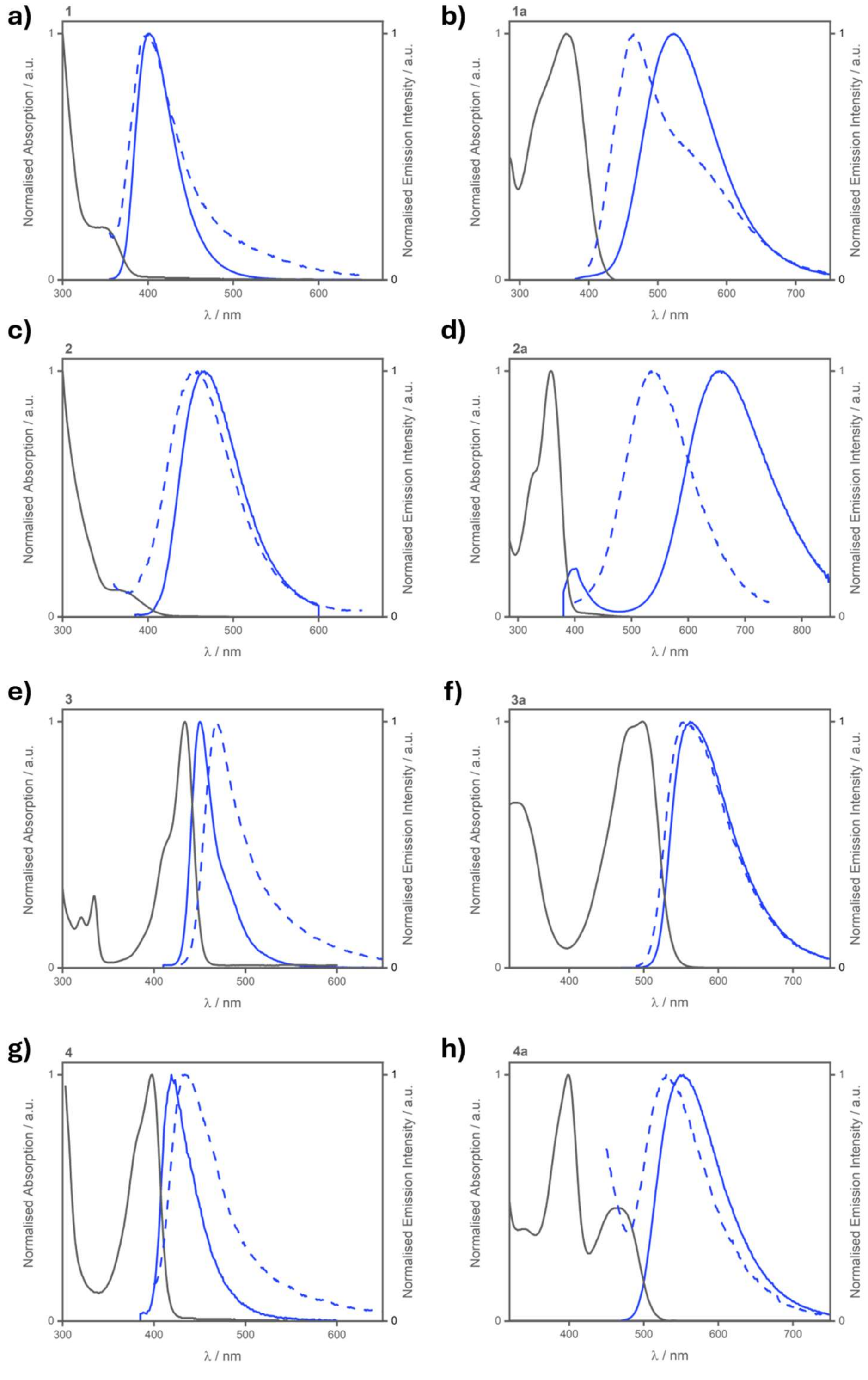

Figure S78. Normalised absorption (grey) and PL in dilute toluene solution (blue, solid) and in 1 wt% PMMA (blue, dashed) of a) **1** (λ_{exc} = 340 nm (solution) or λ_{exc} = 335 nm (film)); b) **1a** (λ_{exc} = 340 nm); c) **2** (λ_{exc} = 366 nm (solution) or λ_{exc} = 320 nm (film)); d) **2a** (λ_{exc} = 340 nm); e) **3** (λ_{exc} = 335 nm (solution) or λ_{exc} = 330 nm (film)); f) **3a** (λ_{exc} = 450 (solution) or 480 nm (film)); g) **4** (λ_{exc} = 366 (solution) or 380 nm (film)); and h) **4a** (λ_{exc} = 450 (solution) or 465 nm (film)).

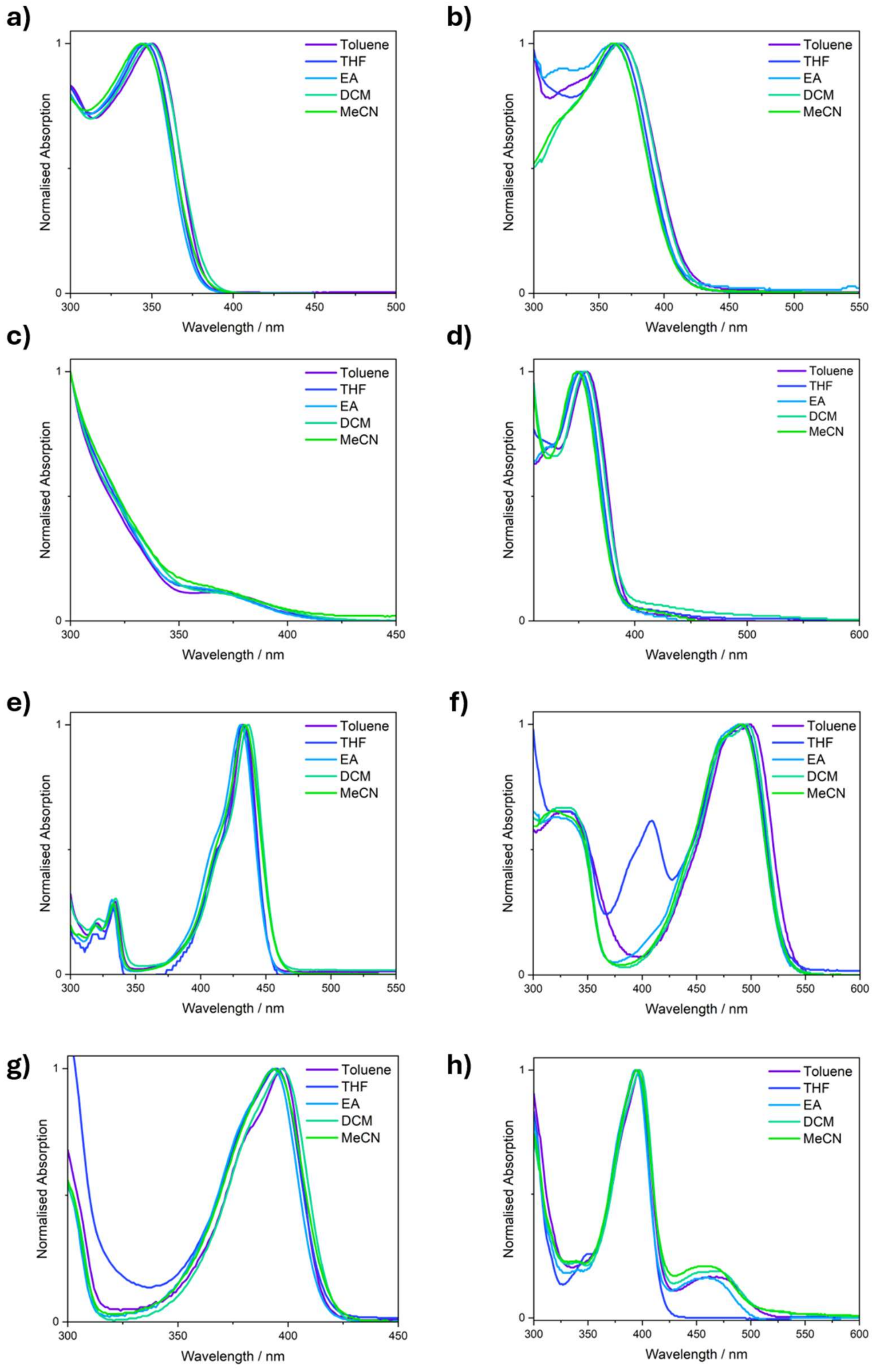

Figure S79. Absorption solvatochromism study of dilute solutions in increasing polarity from toluene to acetonitrile (MeCN) of a) **1** b) **1a**; c) **2**; d) **2a**; e) **3**; f) **3a**; g) **4**; and h) **4a**.

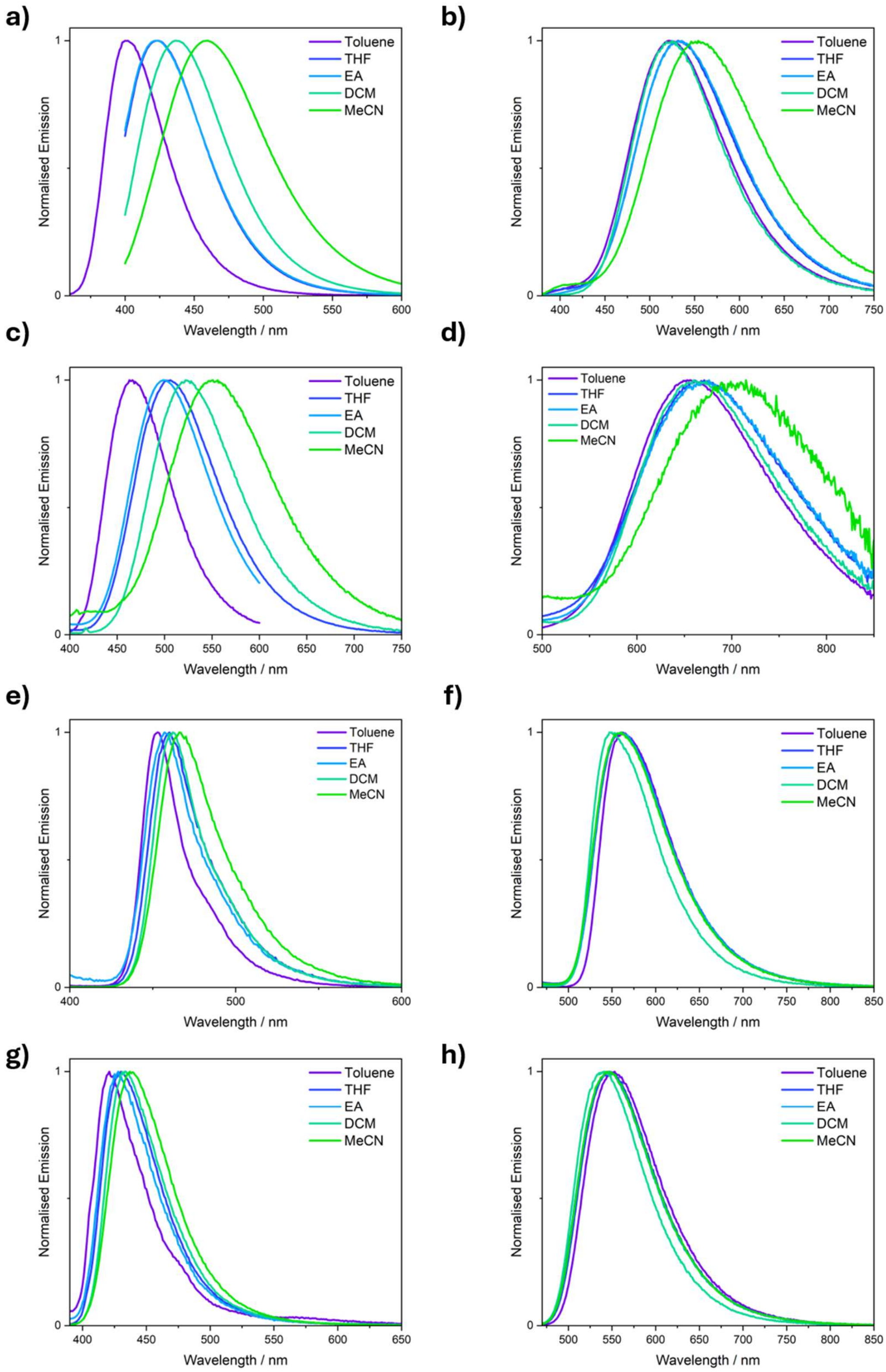

Figure S80. PL solvatochromism study of dilute solutions in increasing polarity from toluene to acetonitrile (MeCN) of a) **1** (λ_{exc} = 340 nm); b) **1a** (λ_{exc} = 360 nm); c) **2** (λ_{exc} = 340 nm); d) **2a** (λ_{exc} = 360 nm); e) **3** (λ_{exc} = 340 nm); f) **3a** (λ_{exc} = 450 nm); g) **4** (λ_{exc} = 360 nm); and h) **4a** (λ_{exc} = 450 nm).

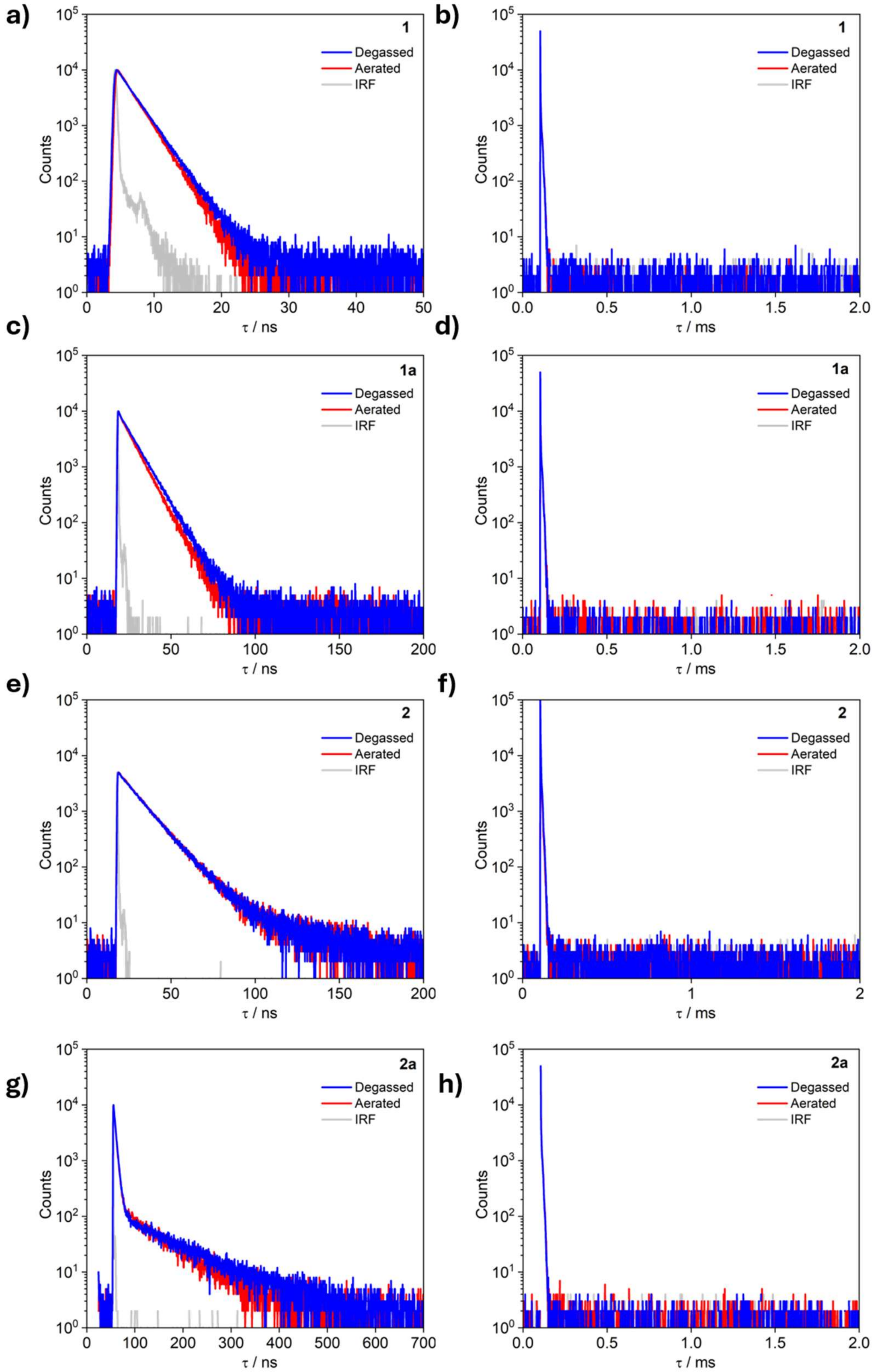

Figure S81. Room temperature PL decays for 1 (a) TCSPC, λ_{exc} = 375, b) MCS, λ_{exc} = 340 nm), 1a (c) TCSPC, λ_{exc} = 375, d) MCS, λ_{exc} = 340 nm), 2 (e) TCSPC, λ_{exc} = 375, f) MCS, λ_{exc} = 366 nm), 2a (g) TCSPC, λ_{exc} = 375, h) MCS, λ_{exc} = 360 nm).

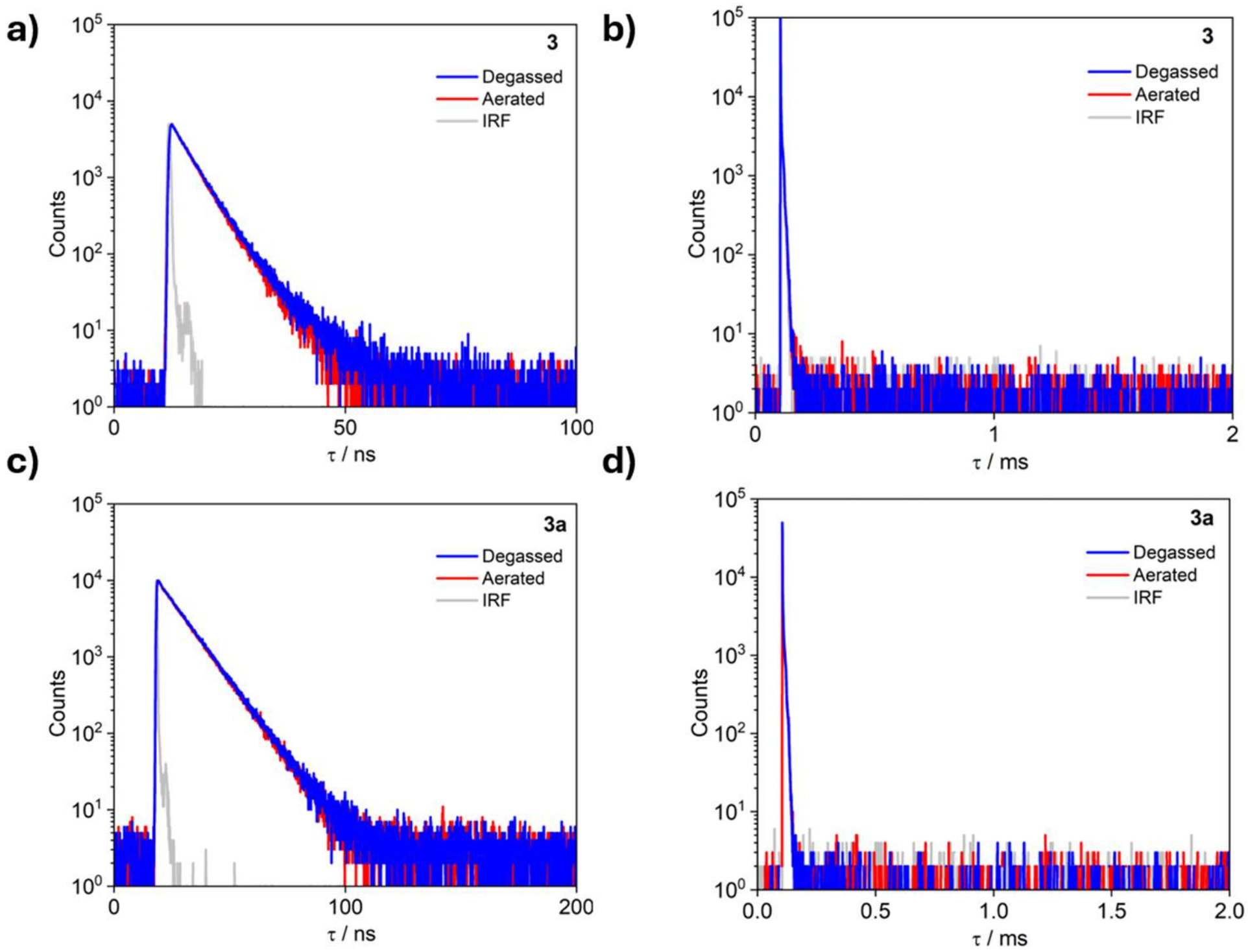

Figure S82. Room temperature PL decays in toluene for **3** (a) TCSPC, λ_{exc} = 375, b) MCS, λ_{exc} = 340 nm) and **3a** (c) TCSPC, λ_{exc} = 375, d) MCS, λ_{exc} = 360 nm).

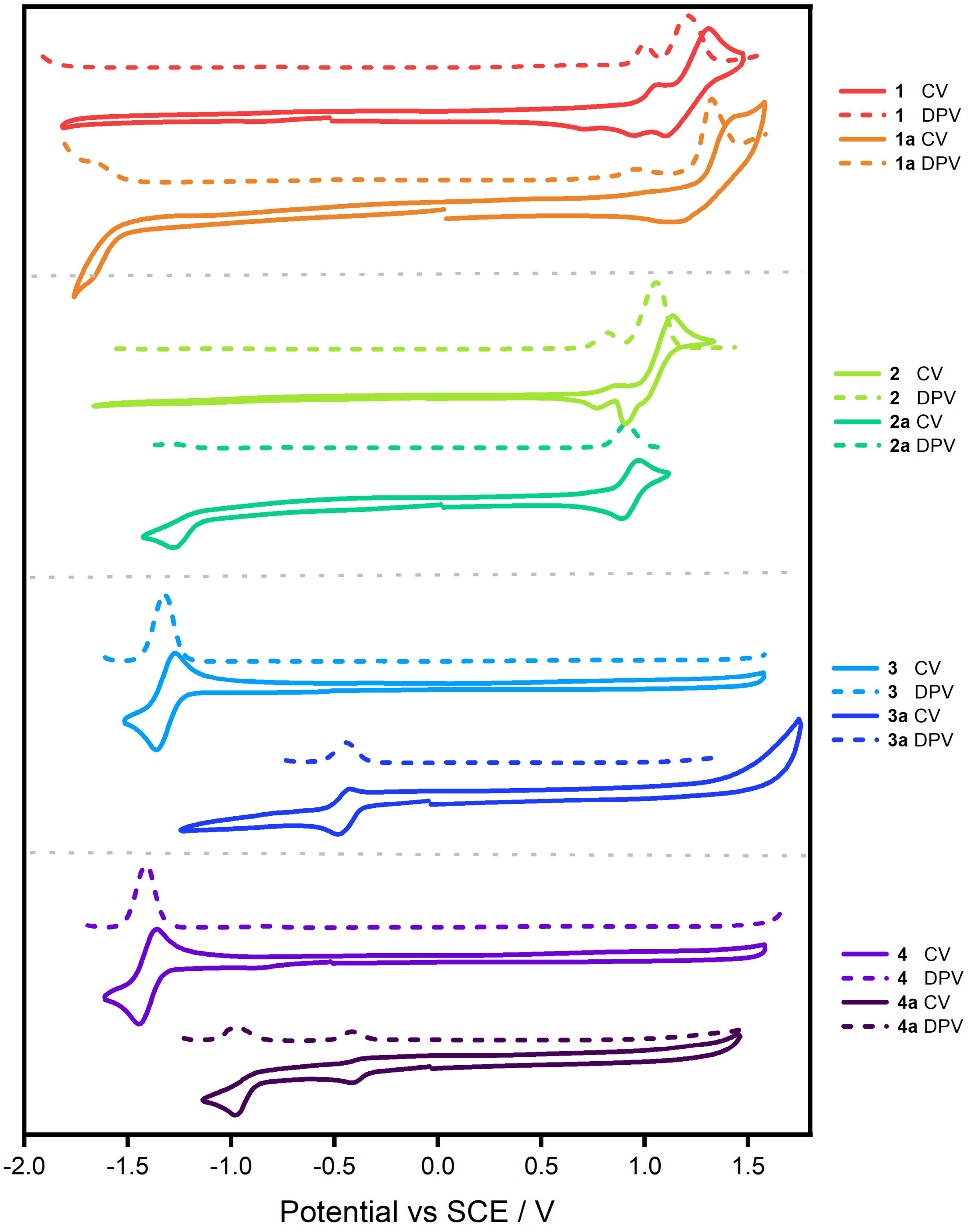

Figure S83. Cyclic voltammetry (CV) and differential pulse voltammetry (DPV) measurements performed in DCM solution (0.1 M [$^{n}Bu_4N$]PF_6) at a scan rate of 0.1 V s^{-1} and referenced to SCE ($E_{ox/red}$ [V *vs.* SCE] = $E_{ox/red}$ [V *vs.* Fc/Fc^+] + 0.46).[8]

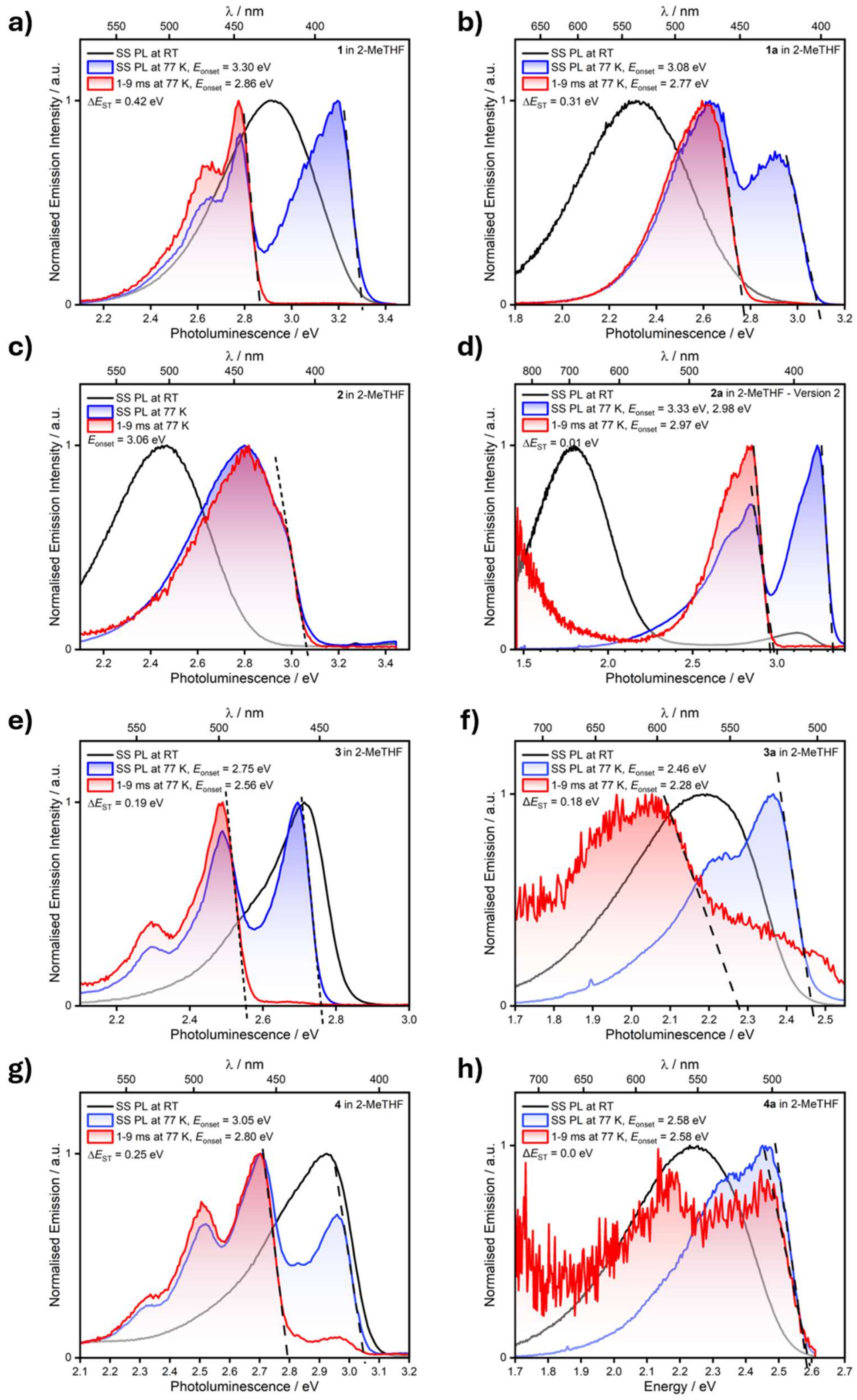

Figure S84. Steady-state PL spectra at room temperature (black) and 77 K (blue) and time-gated PL spectra (1-9 ms; red) in 2-MeTHF glass of a) **1** (λ_{exc} = 340 nm); b) **1a** (λ_{exc} = 360 nm); c) **2** (λ_{exc} = 340 nm); d) **2a** (λ_{exc} = 340 nm); e) **3** (λ_{exc} = 340 nm); f) **3a** (λ_{exc} = 450 nm); g) **4** (λ_{exc} = 340 nm); h) **4a** (λ_{exc} = 460 nm).

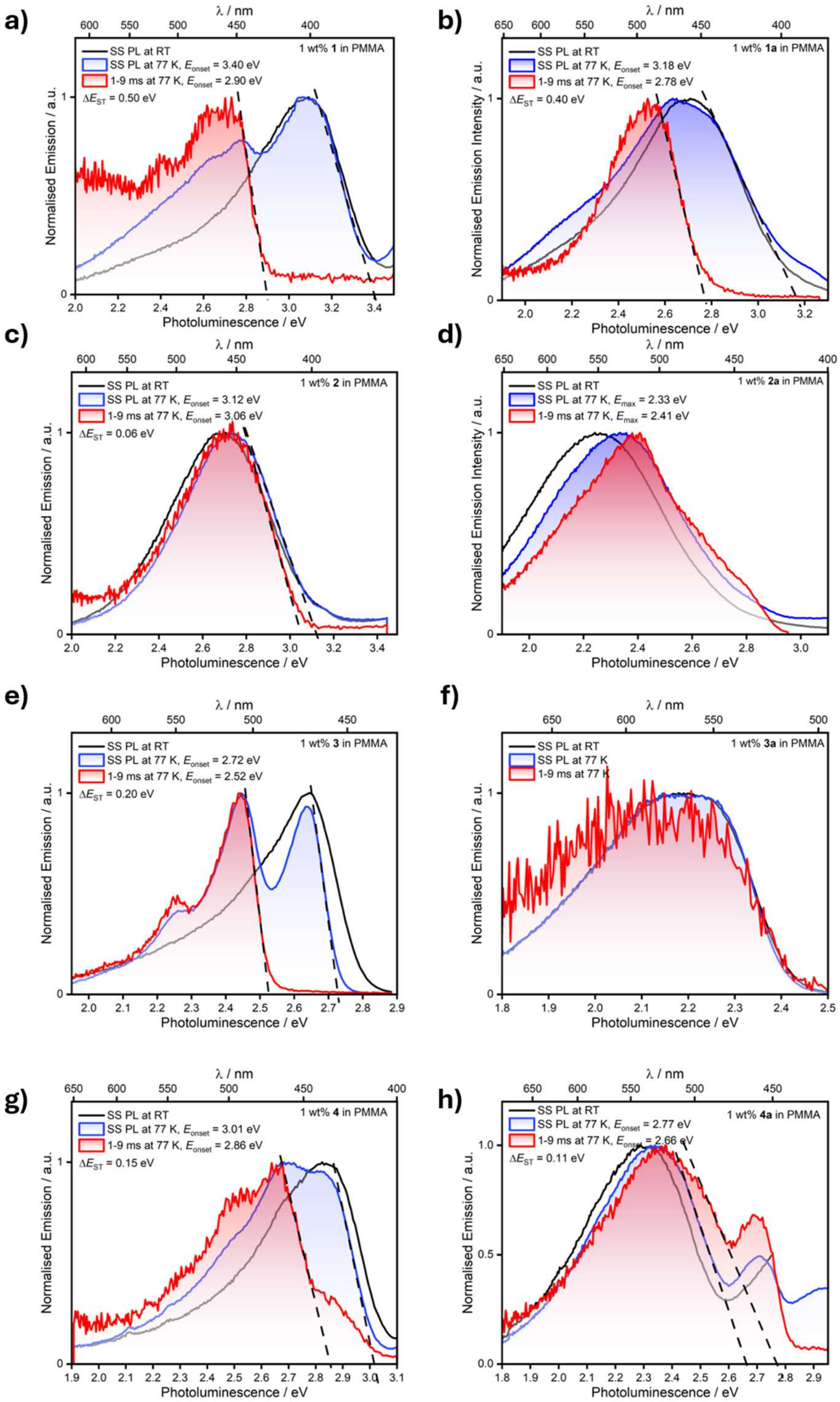

Figure S85. Steady-state PL spectra at room temperature (black) and 77 K (blue) and time-gated PL spectra (1-9 ms; red) in 1 wt% emitter in PMMA film of a) **1** (λ_{exc} = 335 nm); b) **1a** (λ_{exc} = 340 nm); c) **2** (λ_{exc} = 320 nm); d) **2a** (λ_{exc} = 360 nm); e) **3** (λ_{exc} = 330 nm); f) **3a** (λ_{exc} = 340 nm); g) **4** (λ_{exc} = 380 nm); h) **4a** (λ_{exc} = 400 nm).

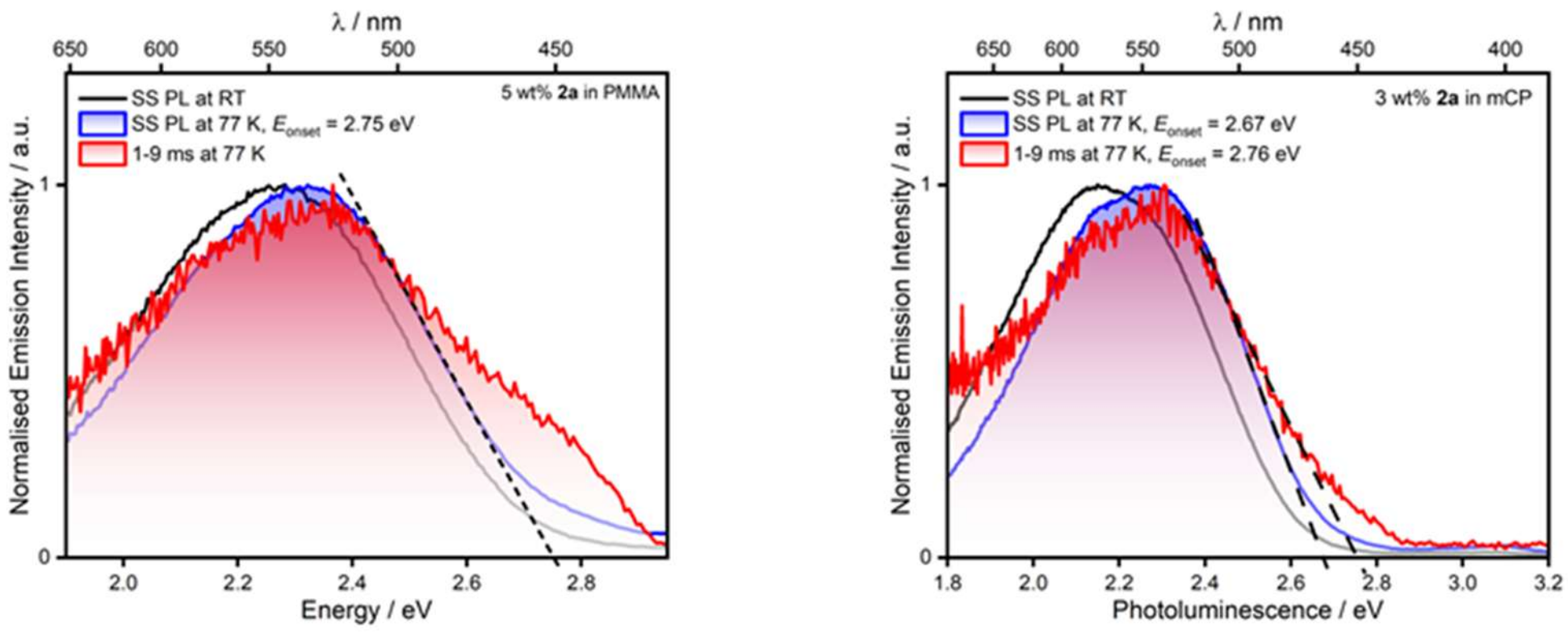

Figure S86. Steady-state PL spectra at room temperature (black) and 77 K (blue) and time-gated PL spectra (1-9 ms; red) of 2a (λ_{exc} = 360 nm) in 5 wt% emitter in PMMA film (left) and 3 wt% in mCP.

These films both showed similar emission spectra to that of the 1 wt% film of **2a.** We tentatively assign the issue with extracting ΔE_{ST} from our (multiple) thin film measurements to the presence of more than one conformer of **2a** in these films (see section 1.9).

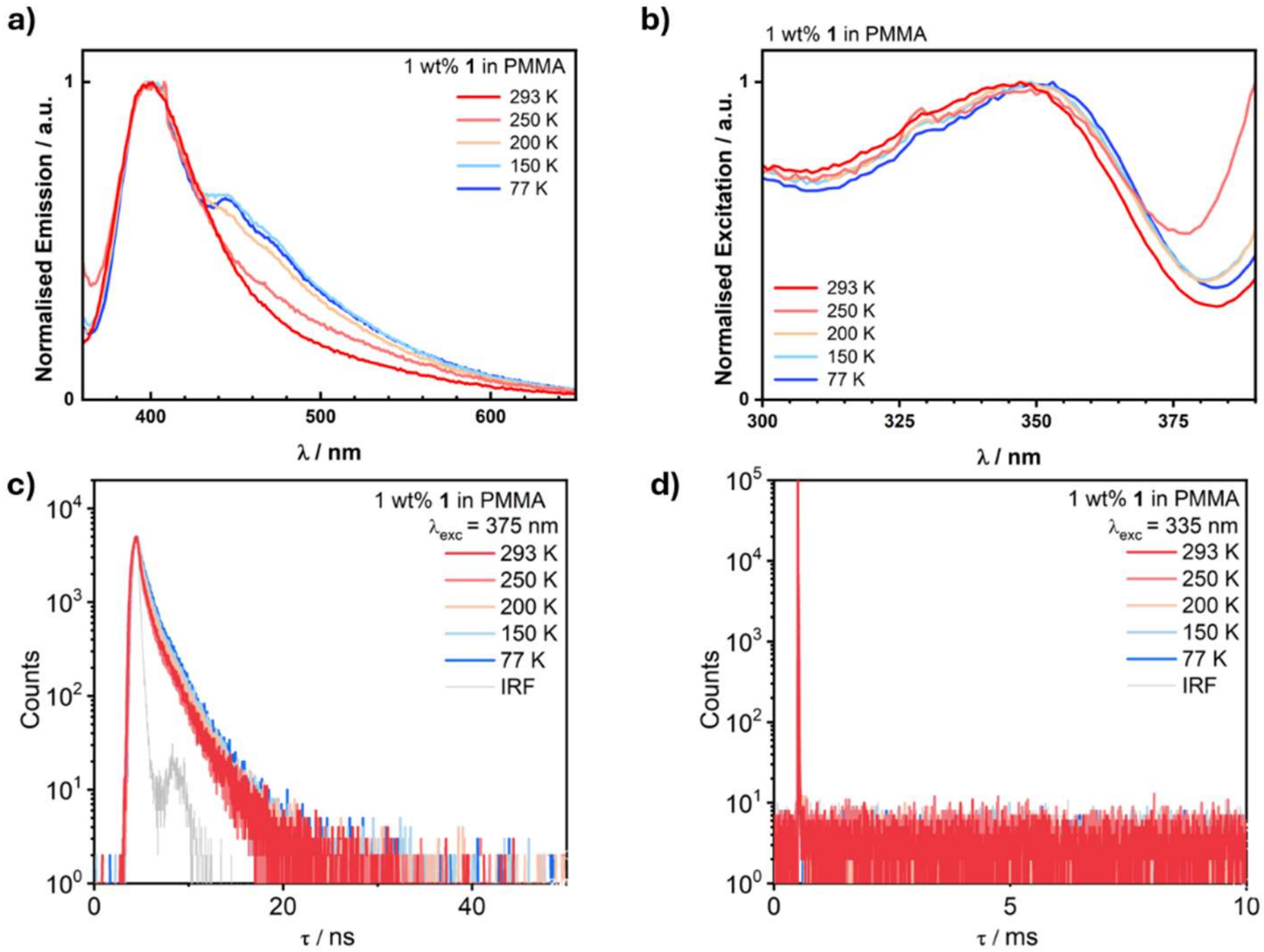

Figure S87. Temperature-dependent measurements of 1 wt% **1** in PMMA under vacuum a) Steady-state PL (λ_{exc} = 335 nm) b) Excitation (λ_{em} = 400 nm) c) prompt PL decay (λ_{exc} = 375 nm, TCSPC) d) delayed emission lifetimes ((λ_{exc} = 335 nm, MCS, 100 Hz).

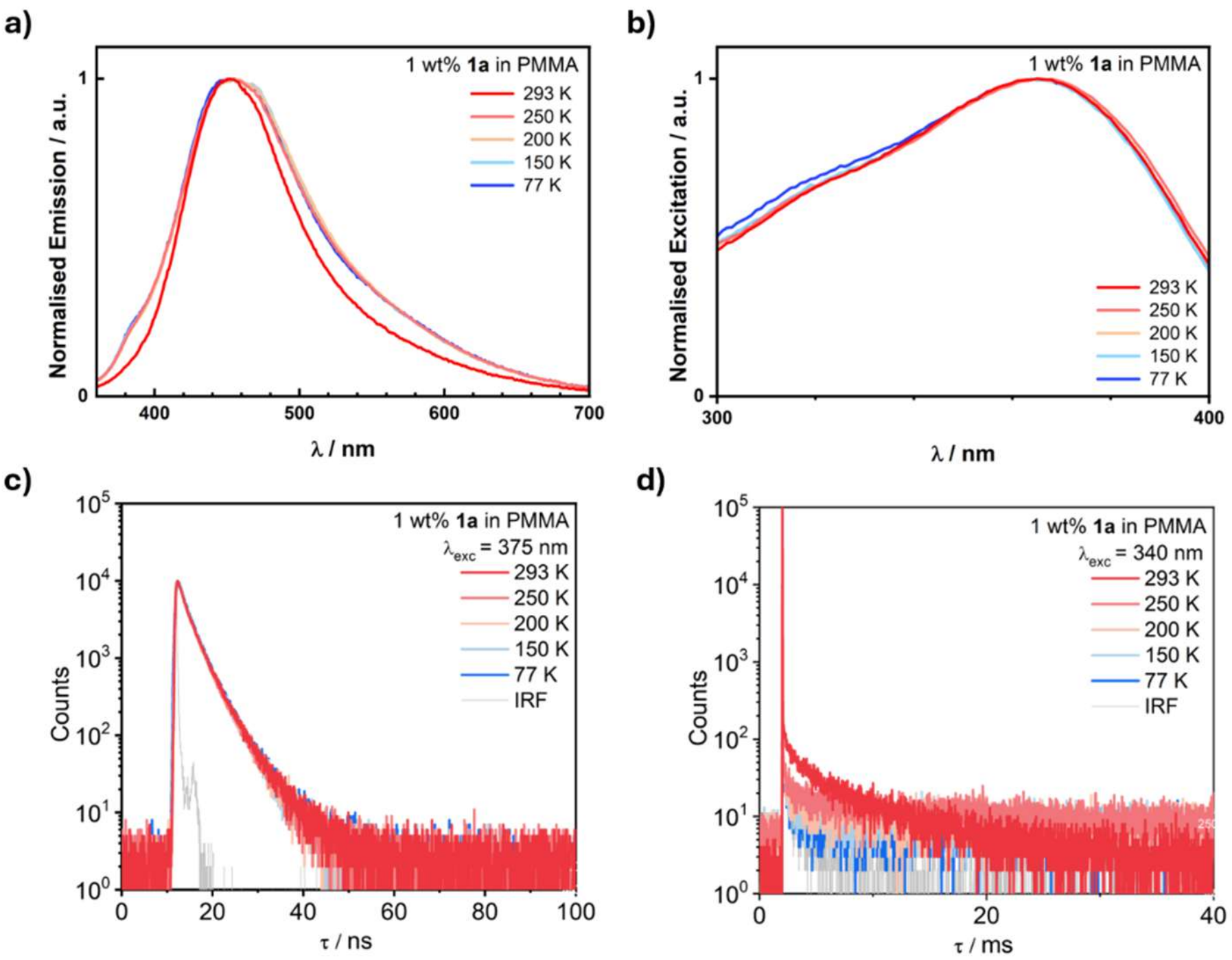

Figure S88. Temperature-dependent measurements of 1 wt% **1a** in PMMA under vacuum a) Steady-state PL (λ_{exc} = 340 nm) b) Excitation (λ_{em} = 450 nm) c) prompt PL decay (λ_{exc} = 375 nm, TCSPC) d) delayed emission lifetimes ((λ_{exc} = 340 nm, MCS, 12.5 Hz).

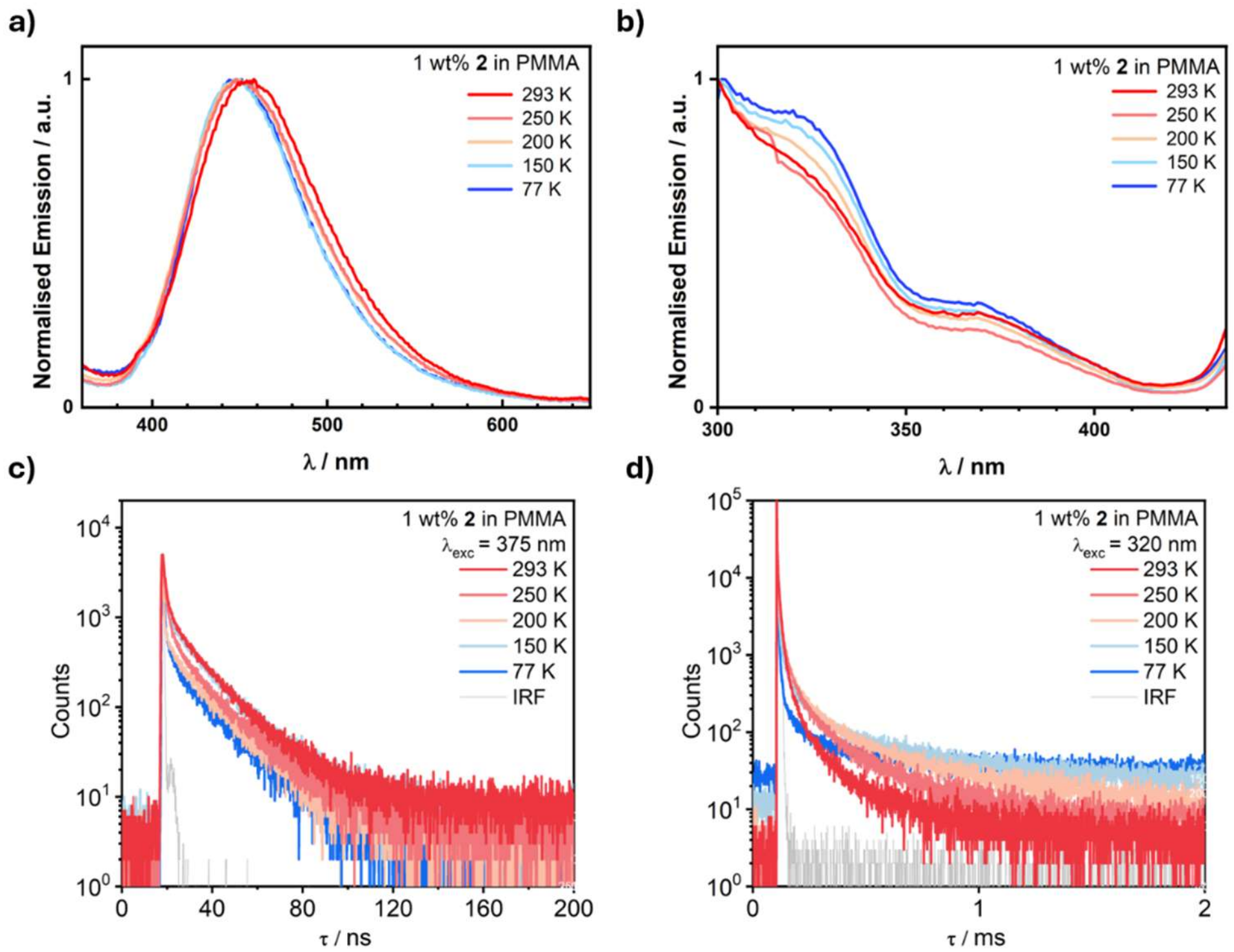

Figure S89. Temperature-dependent measurements of 1 wt% **2** in PMMA under vacuum a) Steady-state PL (λ_{exc} = 320 nm) b) Excitation (λ_{em} = 454 nm) c) prompt PL decay (λ_{exc} = 375 nm, TCSPC) d) delayed emission lifetimes (λ_{exc} = 320 nm, TCSPC).

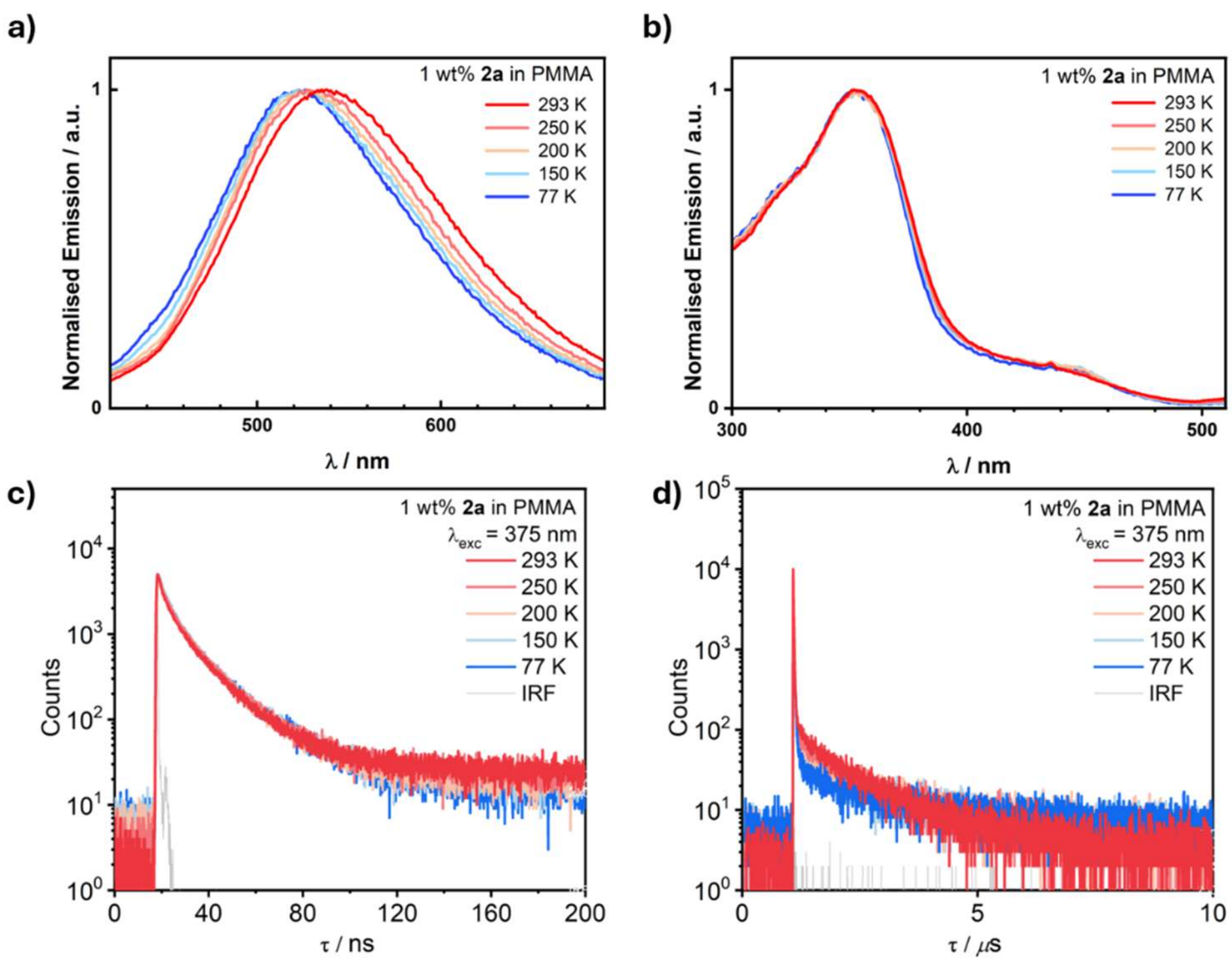

Figure S90. Temperature-dependent measurements of 1 wt% **2a** in PMMA under vacuum a) Steady-state PL (λ_{exc} = 360 nm) b) Excitation (λ_{em} = 535 nm) c) prompt PL decay (λ_{exc} = 375 nm, TCSPC) d) delayed emission lifetimes (λ_{exc} = 375 nm, TCSPC).

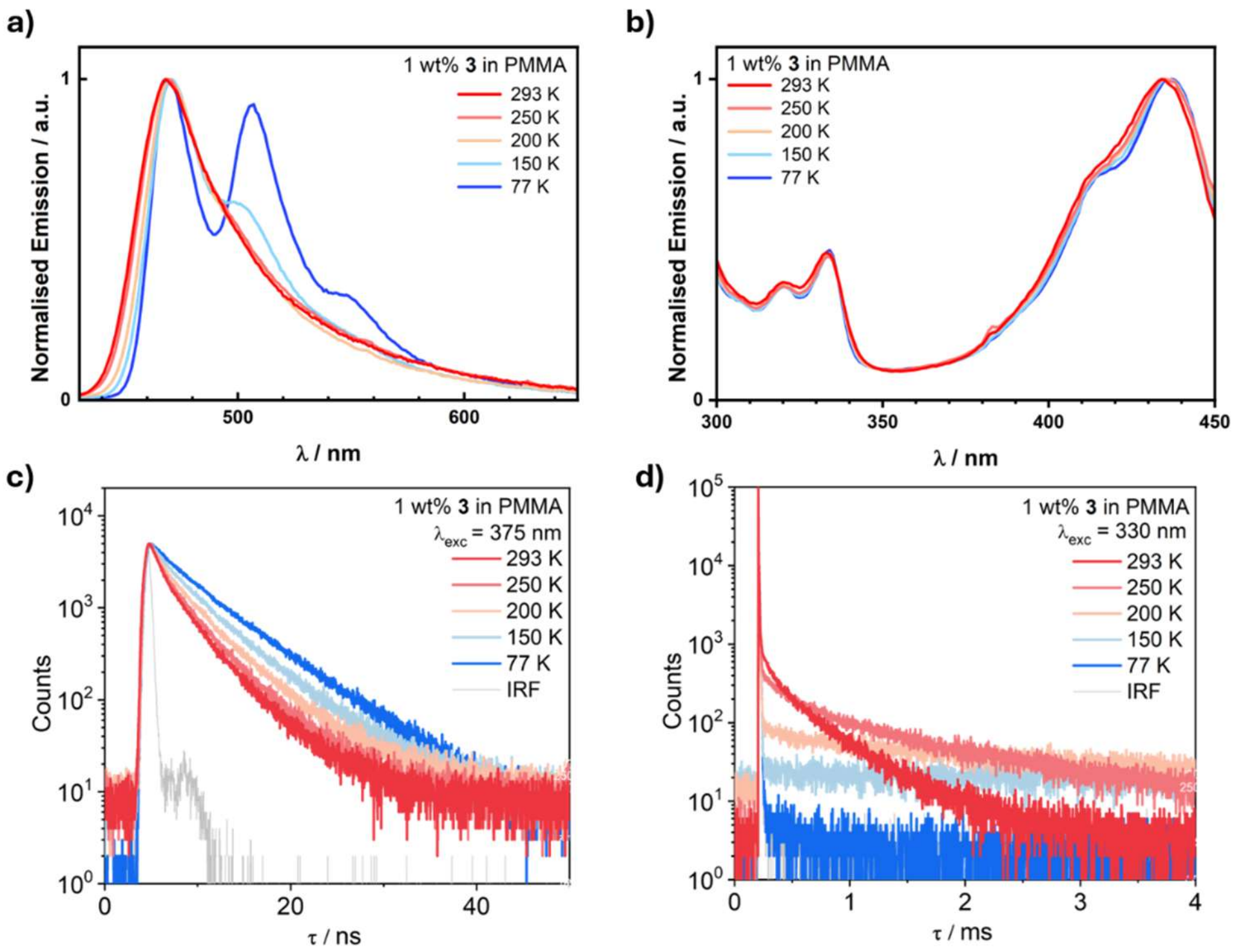

Figure S91. Temperature-dependent measurements of 1 wt% **3** in PMMA under vacuum a) Steady-state PL (λ_{exc} = 465 nm) b) Excitation (λ_{em} = 550 nm) c) prompt PL decay (λ_{exc} = 375 nm, TCSPC) d) delayed emission lifetimes (λ_{exc} = 330 nm, MCS, 100 Hz).

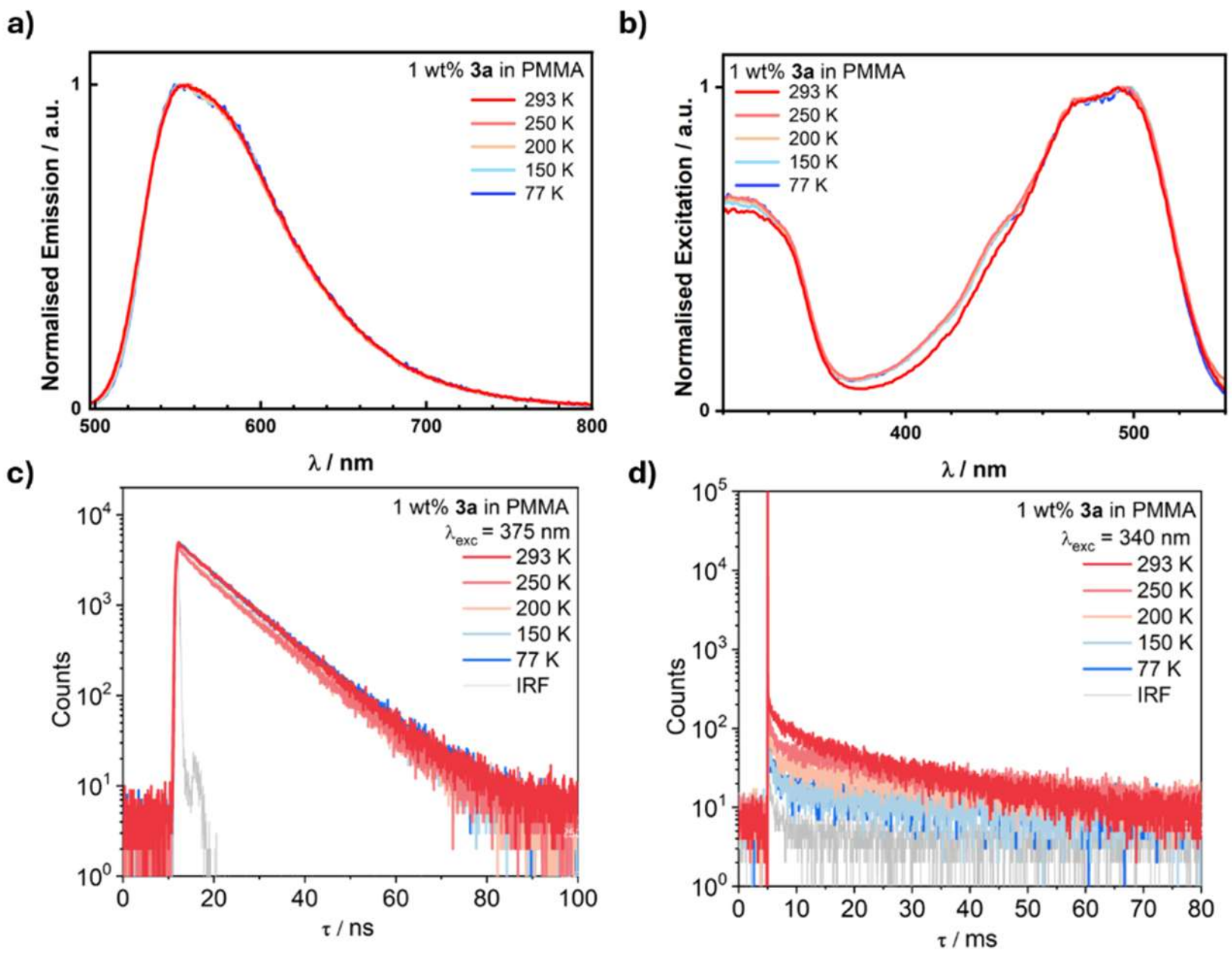

Figure S92. Temperature-dependent measurements of 1 wt% **3a** in PMMA under vacuum a) Steady-state PL (λ_{exc} = 340 nm) b) Excitation (λ_{em} = 555 nm) c) prompt PL decay (λ_{exc} = 375 nm, TCSPC) d) delayed emission lifetimes (λ_{exc} = 340 nm, MCS, 10 Hz).

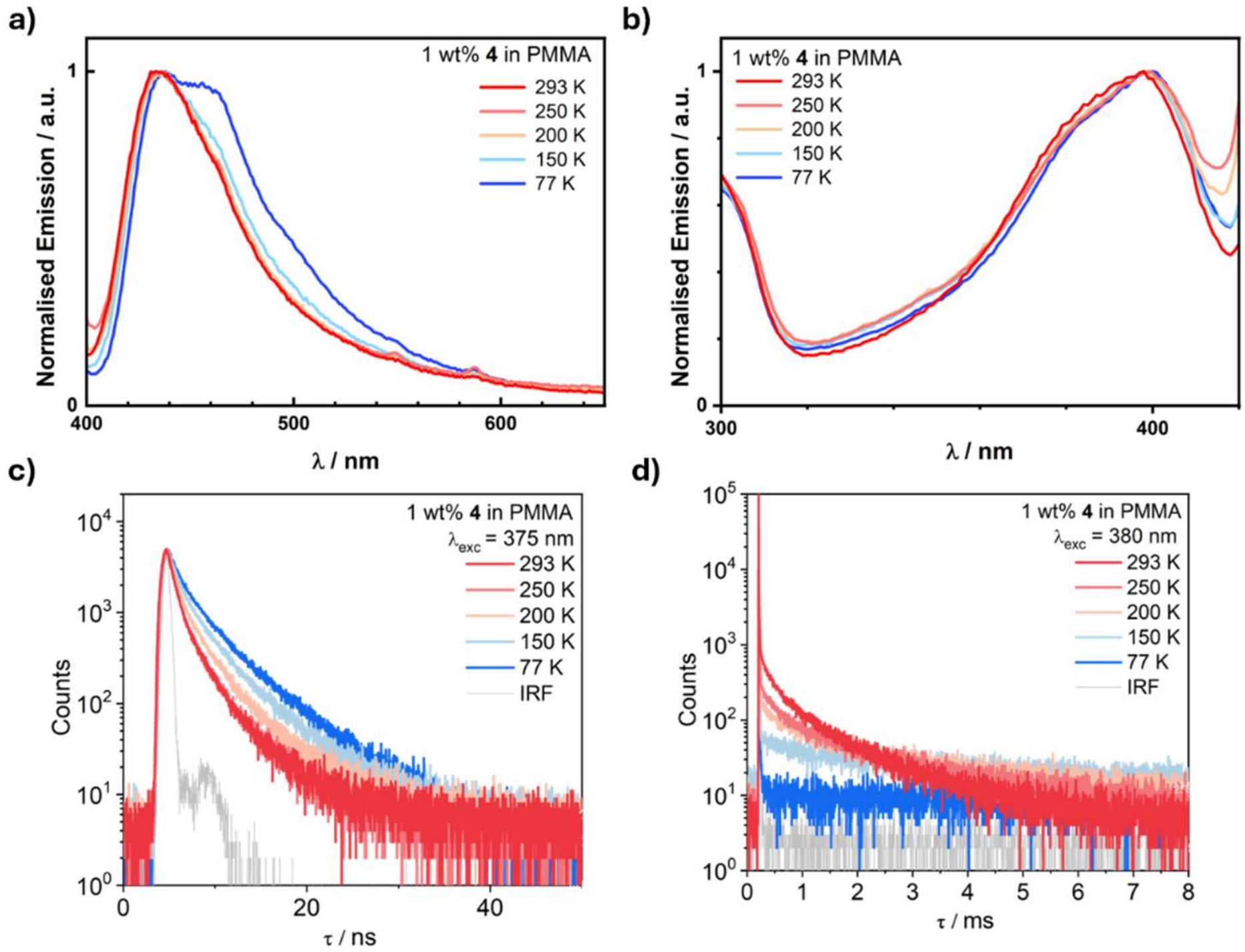

Figure S93. Temperature-dependent measurements of 1 wt% **4** in PMMA under vacuum a) Steady-state PL (λ_{exc} = 380 nm) b) Excitation (λ_{em} = 435 nm) c) prompt PL decay (λ_{exc} = 375 nm, TCSPC) d) delayed emission lifetimes (λ_{exc} = 380 nm, MCS, 100 Hz).

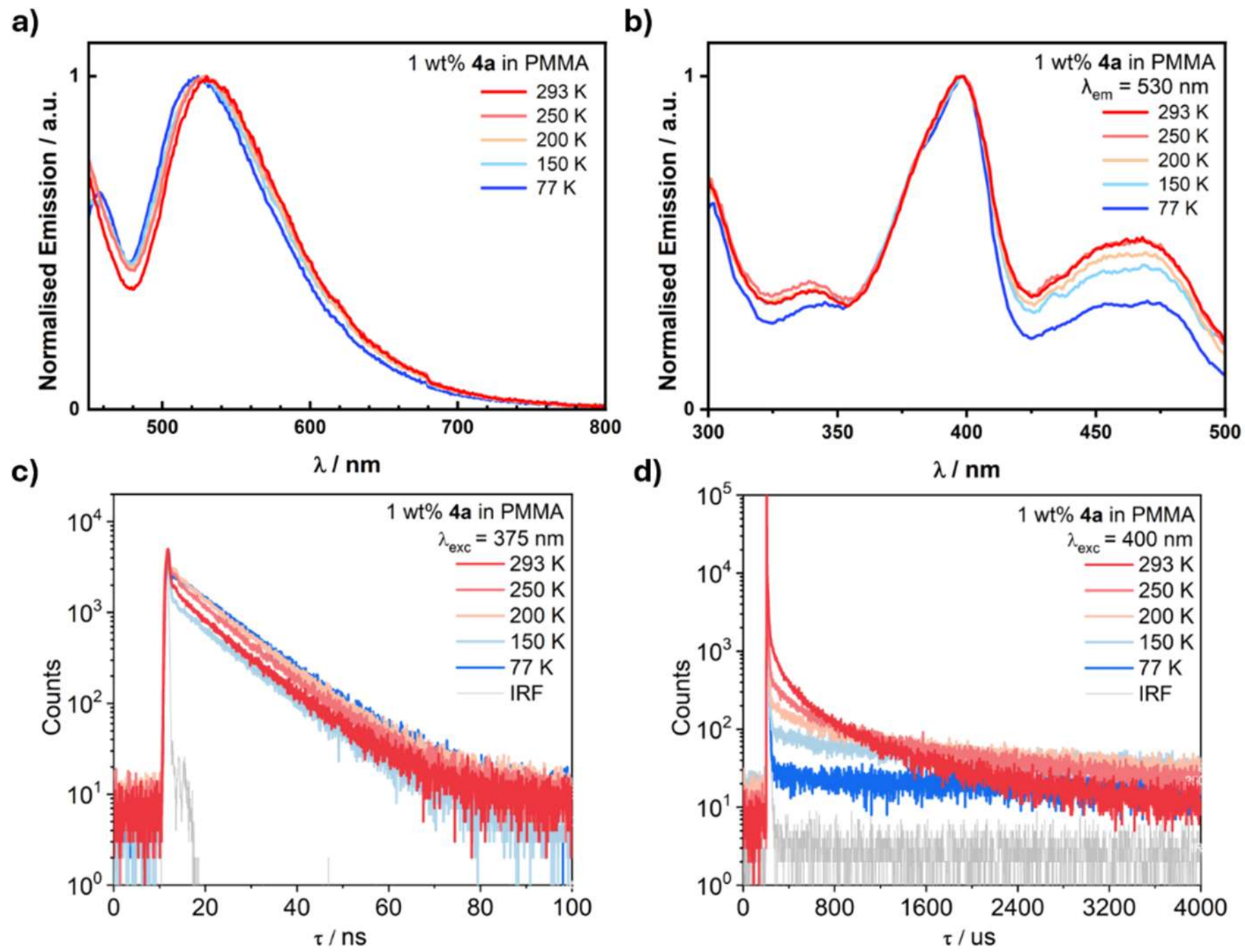

Figure S94. Temperature-dependent measurements of 1 wt% **4a** in PMMA under vacuum a) Steady-state PL (λ_{exc} = 465 nm) b) Excitation (λ_{em} = 550 nm) c) prompt PL decay (λ_{exc} = 375 nm, TCSPC) d) delayed emission lifetimes (λ_{exc} = 390 nm, MCS, 100 Hz).

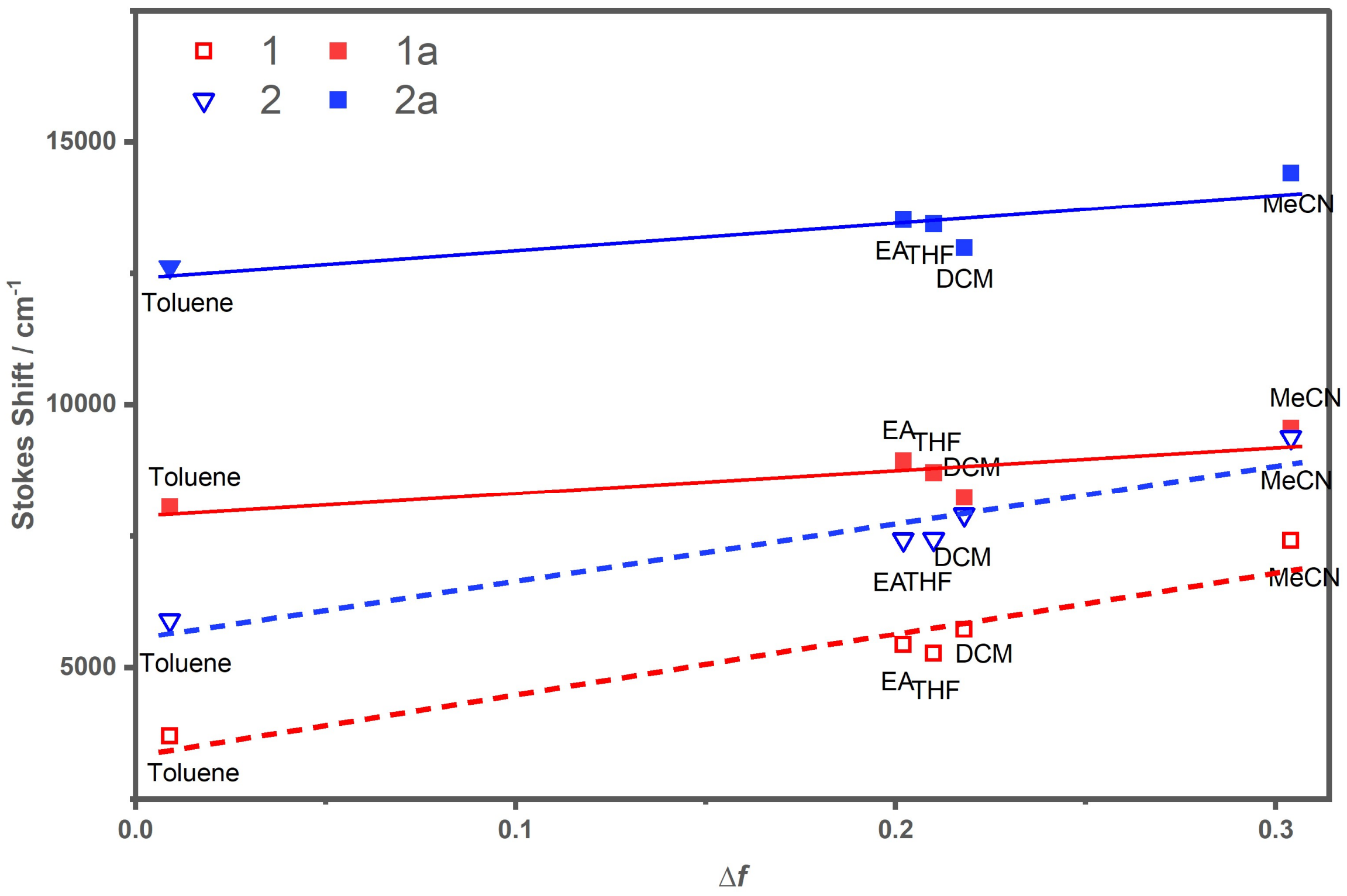

Figure S95. Lippert-Mataga plot of **1**, **1a**, **2** and **2a**

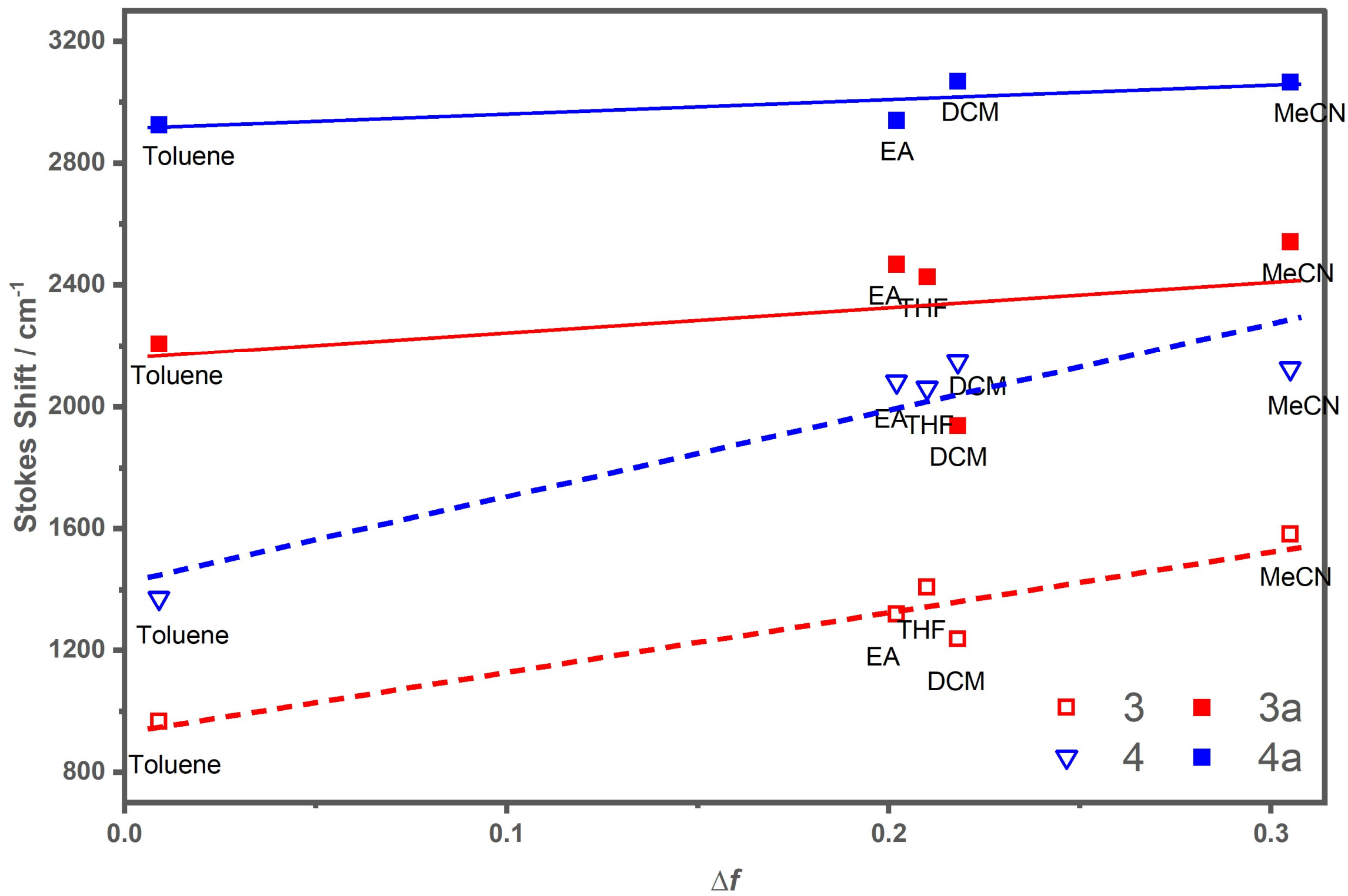

Figure S96. Lippert-Mataga plot of **3**, **3a**, **4** and **4a**

1.8 Computational details

1.8.1 1,3,5-Triphenyltriazine BCF binding calculations

The free energy change upon Lewis adduct formation between 1,3,5-triphenyltriazine and BCF were performed using the Gaussian 16 software suite.[23] Geometry optimisation were performed at the MN15/Def2TZVP (scrf: $CHCl_3$) level of theory, and frequency calculations were performed at the same level to confirm minima (no negative frequencies).[24-28]

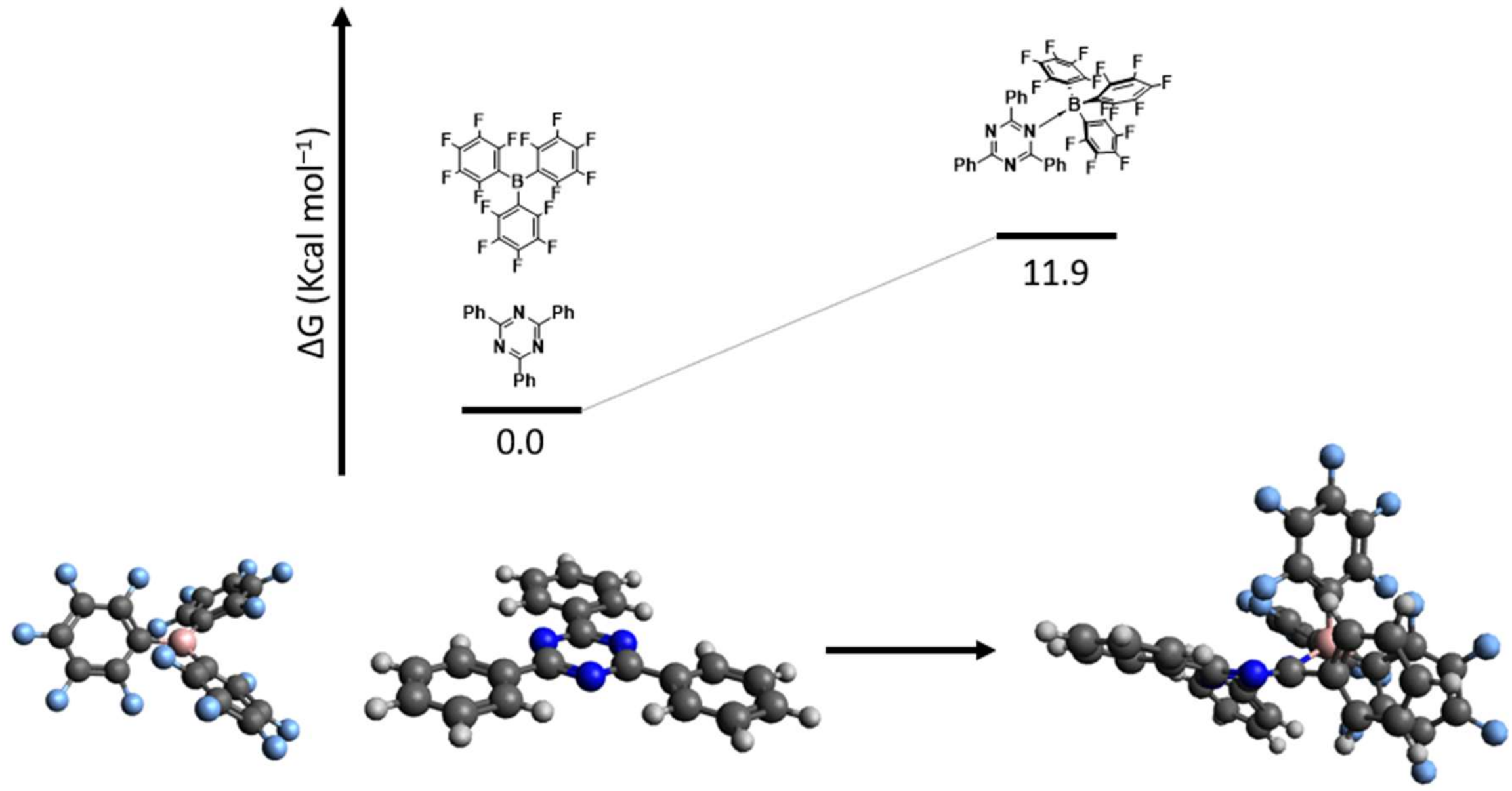

Figure S97. Top: The calculated ΔG change upon binding of BCF to triphenyl triazine, showing a highly endergonic process (+11.9 Kcal mol^{-1}, MN15/Def2TZVP, scrf: $CHCl_3$). Bottom: The calculated large distortion of the triazine ring upon adduct formation with BCF, indicating significant steric derived distortion.

1.8.2 Lewis acid bathochromic shift model orbital calculations

NBO calculations were performed using NBO 3.1 as part of the Gaussian 16 software suite.[23, 29] Geometry optimisation was carried out at the PBE0/6-31G(d,p) (SMD: THF) level of theory, and frequency calculations were performed at the same level to confirm minima (no negative frequencies) and generate molecular orbitals.[24-28] Orbitals were visualised using Avogadro.[30]

1.8.3 DMAC DPS BF_3 MO and NBO calculations

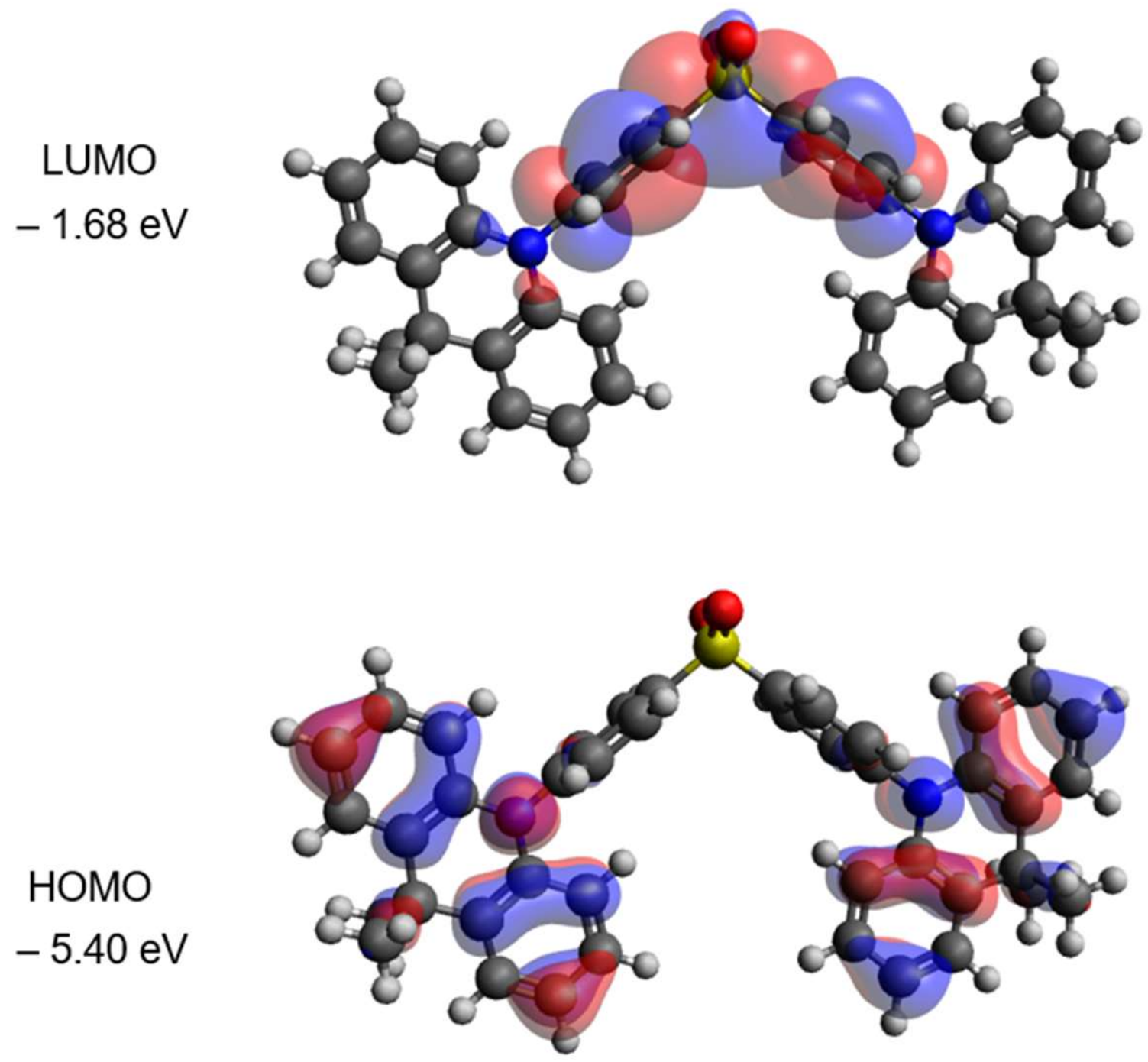

Figure S98. Selected molecular orbitals (HOMO & LUMO) and their respective energies calculated for DMAC-DPS (**2**).

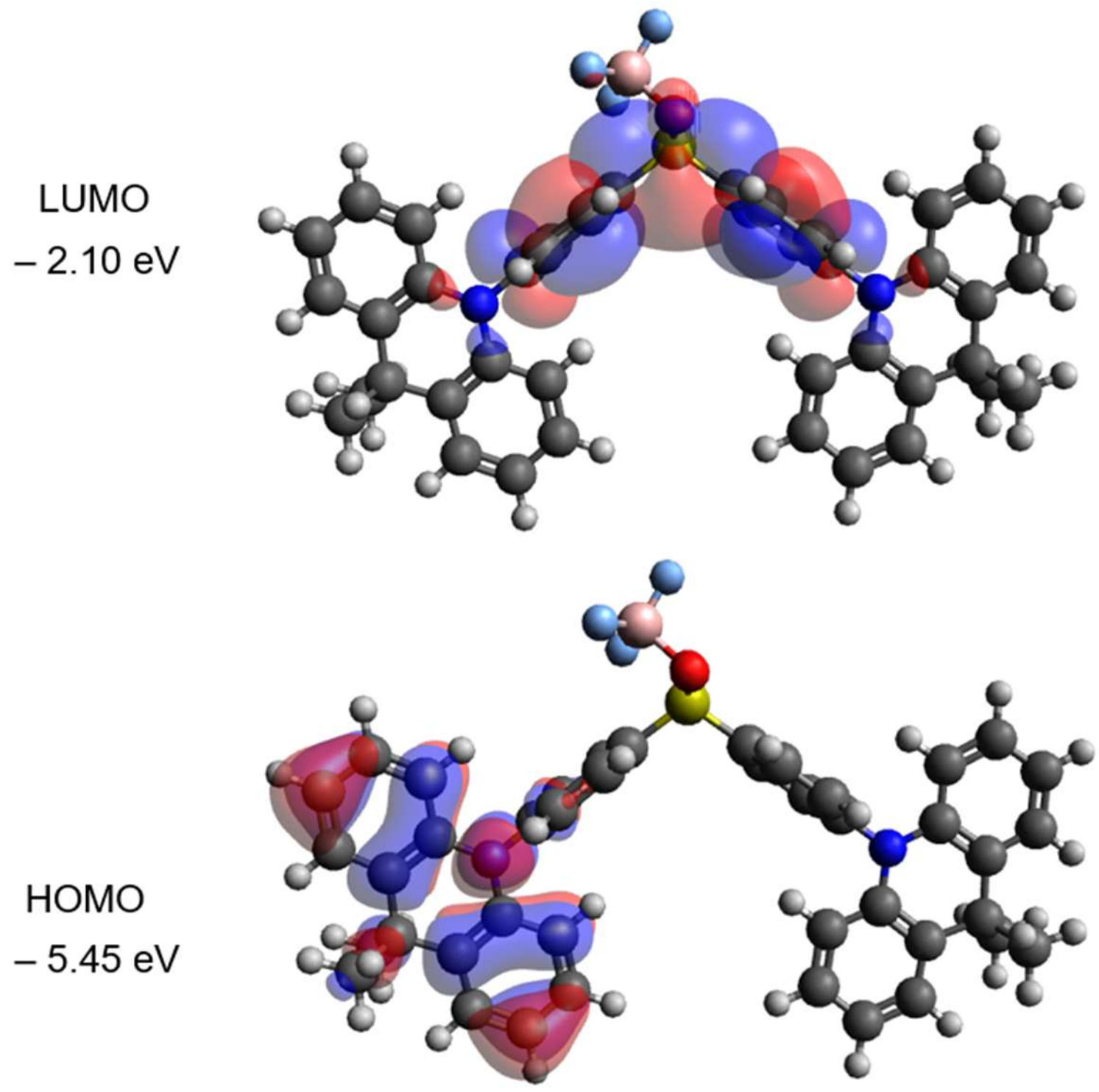

Figure S99. Selected molecular orbitals (HOMO & LUMO) and their respective energies calculated for DMAC-DPS BF_3.

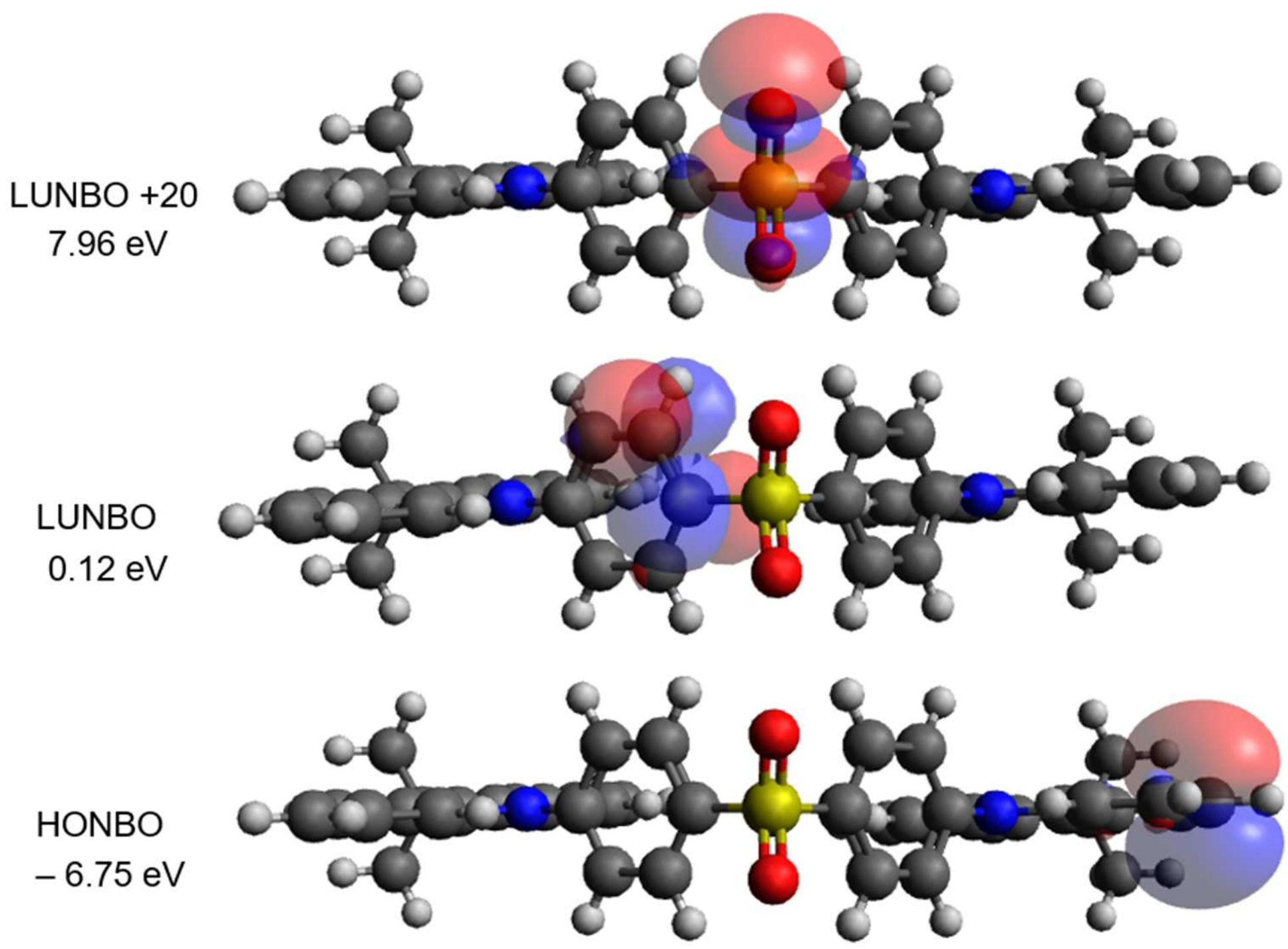

Figure S100. Selected natural bonding orbitals (HONBO, LUNBO & S-O σ^* LUNBO+20) and their respective energies calculated for DMAC-DPS (**2**).

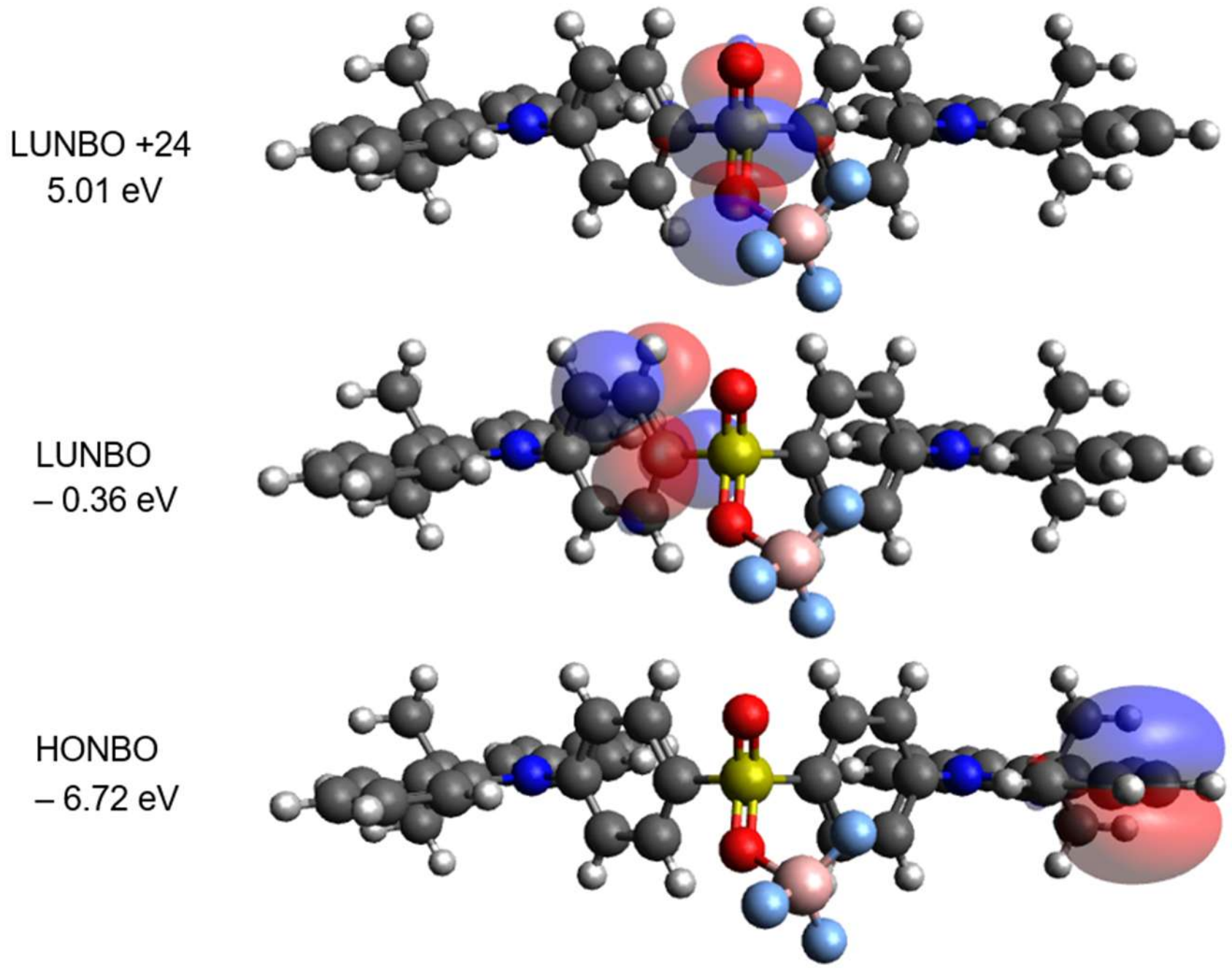

Figure S101. Selected natural bonding orbitals (HONBO, LUNBO & BF_3-bound S-O σ^* LUNBO+24) and their respective energies calculated for DMAC-DPS BF_3.

1.8.4 DiKTa BF_3 MO and NBO calculations

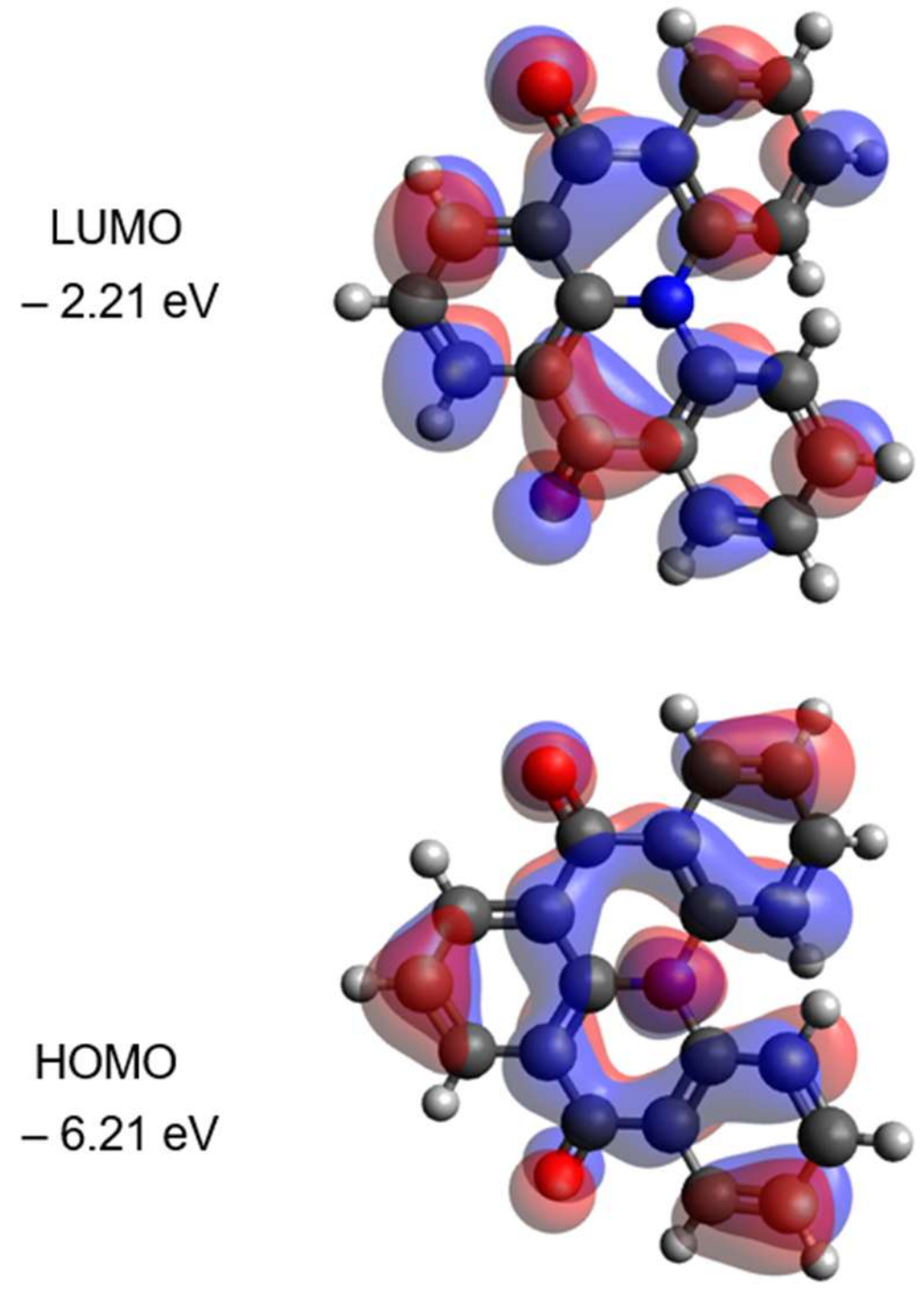

Figure S102. Selected molecular orbitals (HOMO & LUMO) and their respective energies calculated for DiKTa (**3**).

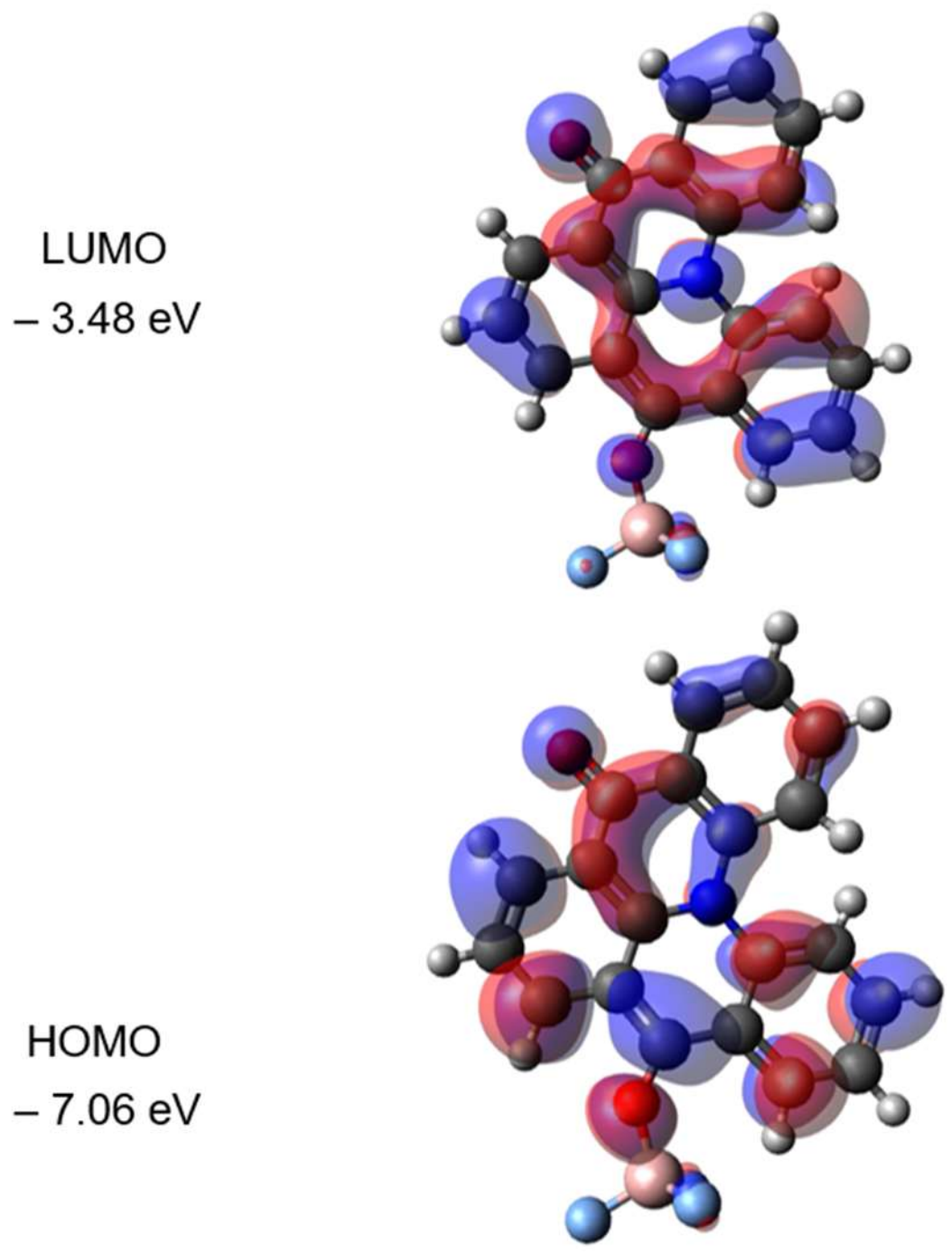

Figure S103. Selected molecular orbitals (HOMO & LUMO) and their respective energies calculated for DiKTa BF_3.

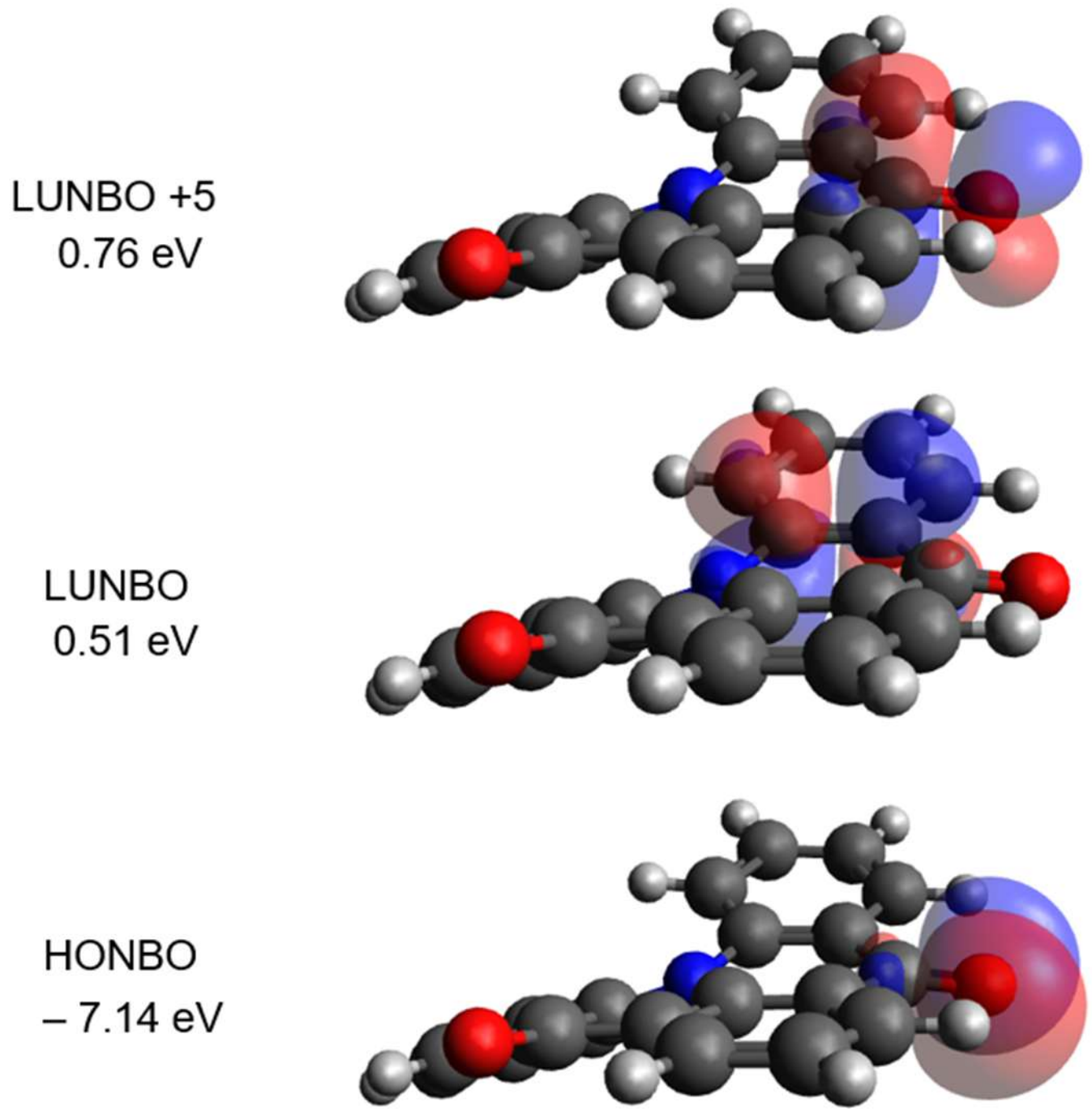

Figure S104. Selected natural bonding orbitals (HONBO, LUNBO & C-O σ* LUNBO+5) and their respective energies calculated for DiKTa (**3**).

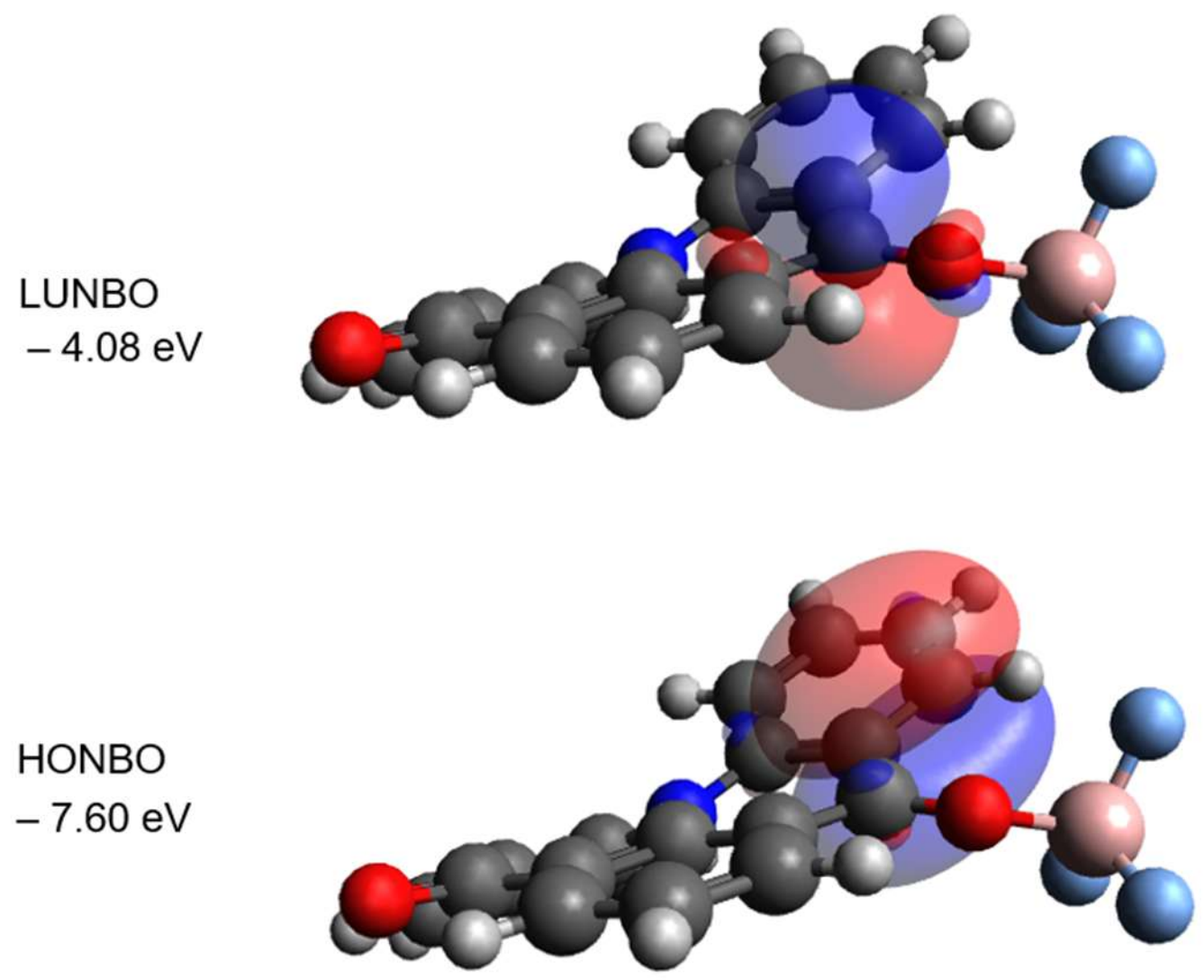

Figure S105. Selected natural bonding orbitals (HONBO, & BF_3-bound C-O σ* LUNBO) and their respective energies calculated for DiKTa BF_3.

1.8.5 TDDFT analysis

TDDFT analysis was submitted to and processed using the Digichem software package version 7, which incorporates a number of publicly available software libraries, including; cclib for the parsing of result files, VMD/Tachyon for 3D rending, Matplotlib for the plotting of graphs, Open Babel/Pybel for file interconversion, and PySOC for the calculations of spin-orbit coupling.[23, 31-39] All ground-state optimizations have been carried out at the Density Functional Theory (DFT) level with Gaussian 16 using the PBE0 functional with the 6-31G(d,p) basis set.[40, 41] For a more accurate prediction of the excited states of the multi-resonant thermally delayed fluorescent (MR-TADF) compounds, modelling using ADC(2) with the cc-pVDZ basis set was performed within Turbomole (7.5.0).[42] Dihedral angles were visualised using Jmol; an open-source Java viewer for chemical structures in 3D.[43] Spin-orbit coupling matrix elements (SOCME) were calculated based on the optimised T_1 excited state geometry using PySOC.[44]

1.8.6 TADF emitters 1a & 2a

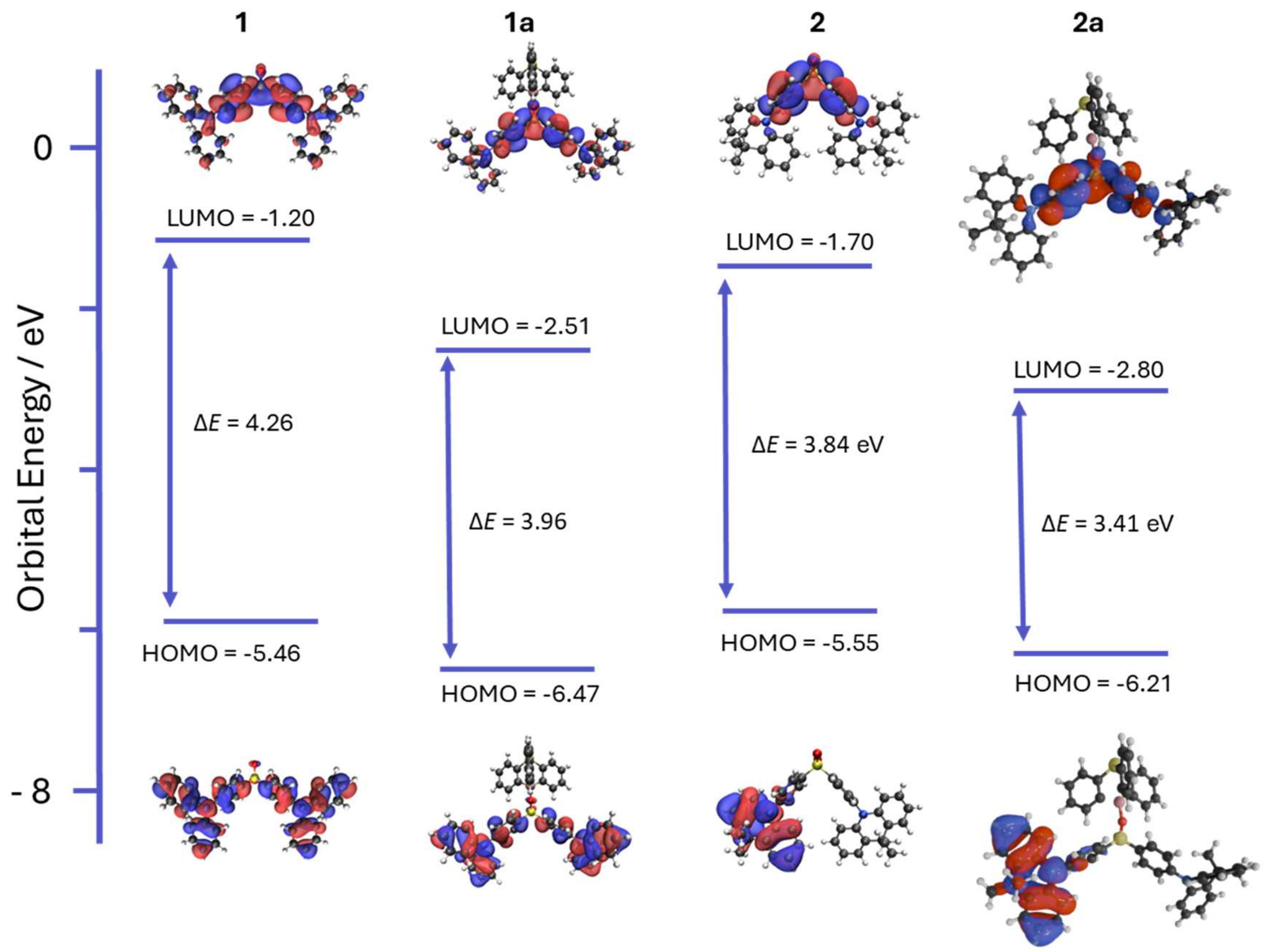

Figure S106. HOMO and LUMO energy levels calculated at the PBE0/6-31G(d,p) (in toluene) level of theory for **1**, **1a**, **2**, and **2a**. Orbital density plots are of the LUMO (top) and HOMO (bottom) (isovalue = 0.02).

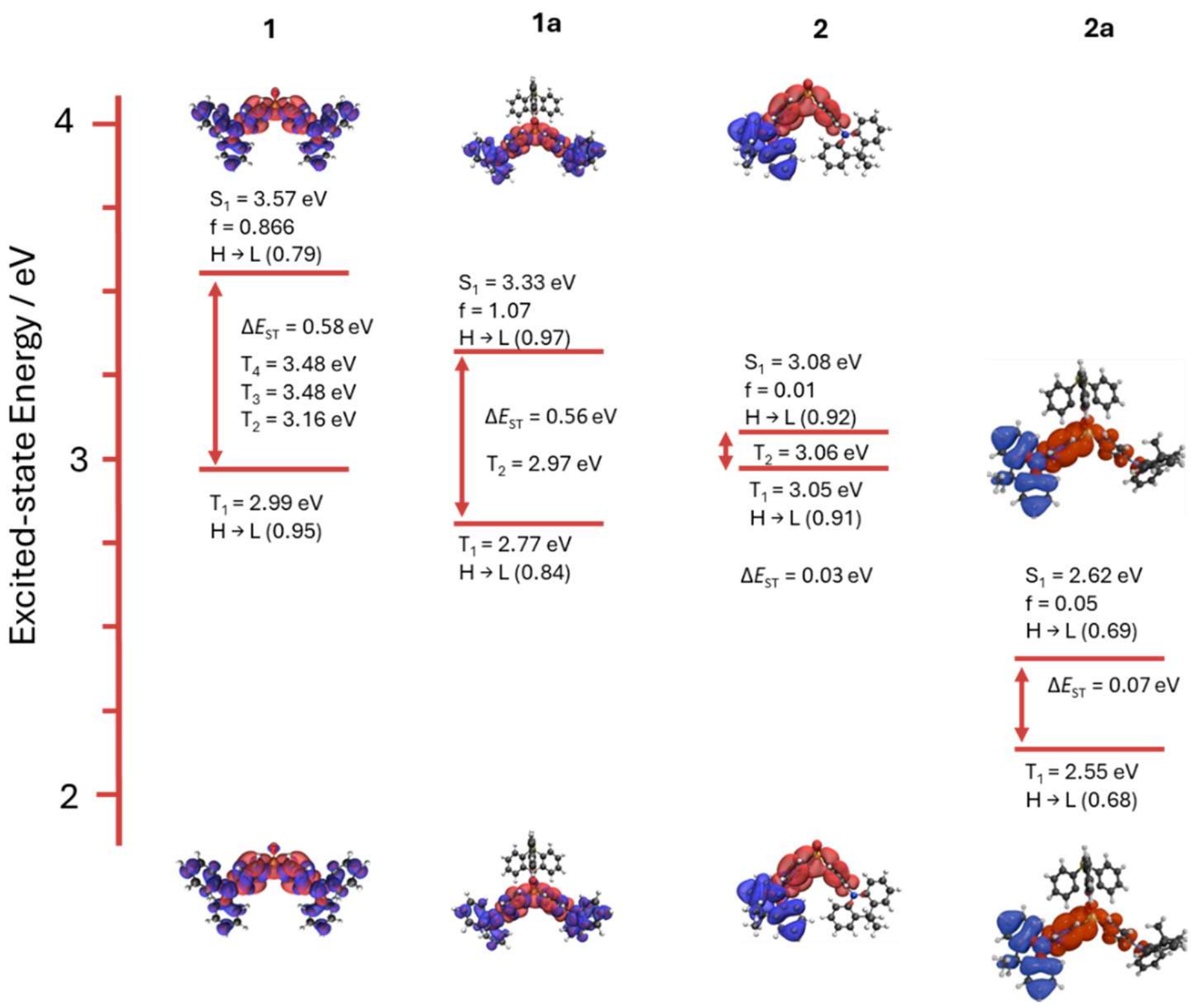

Figure S107. Excited state energy levels calculated at the PBE0/6-31G(d,p) (in toluene) level of theory for **1**, **1a**, **2**, and **2a**. Density plots of the NTO hole (blue) and electron (red) of the S_1 (top) and T_1 (bottom) states (isovalue = 0.02)

1.8.7 MR-TADF emitters 3a, 3-BCF & 4a

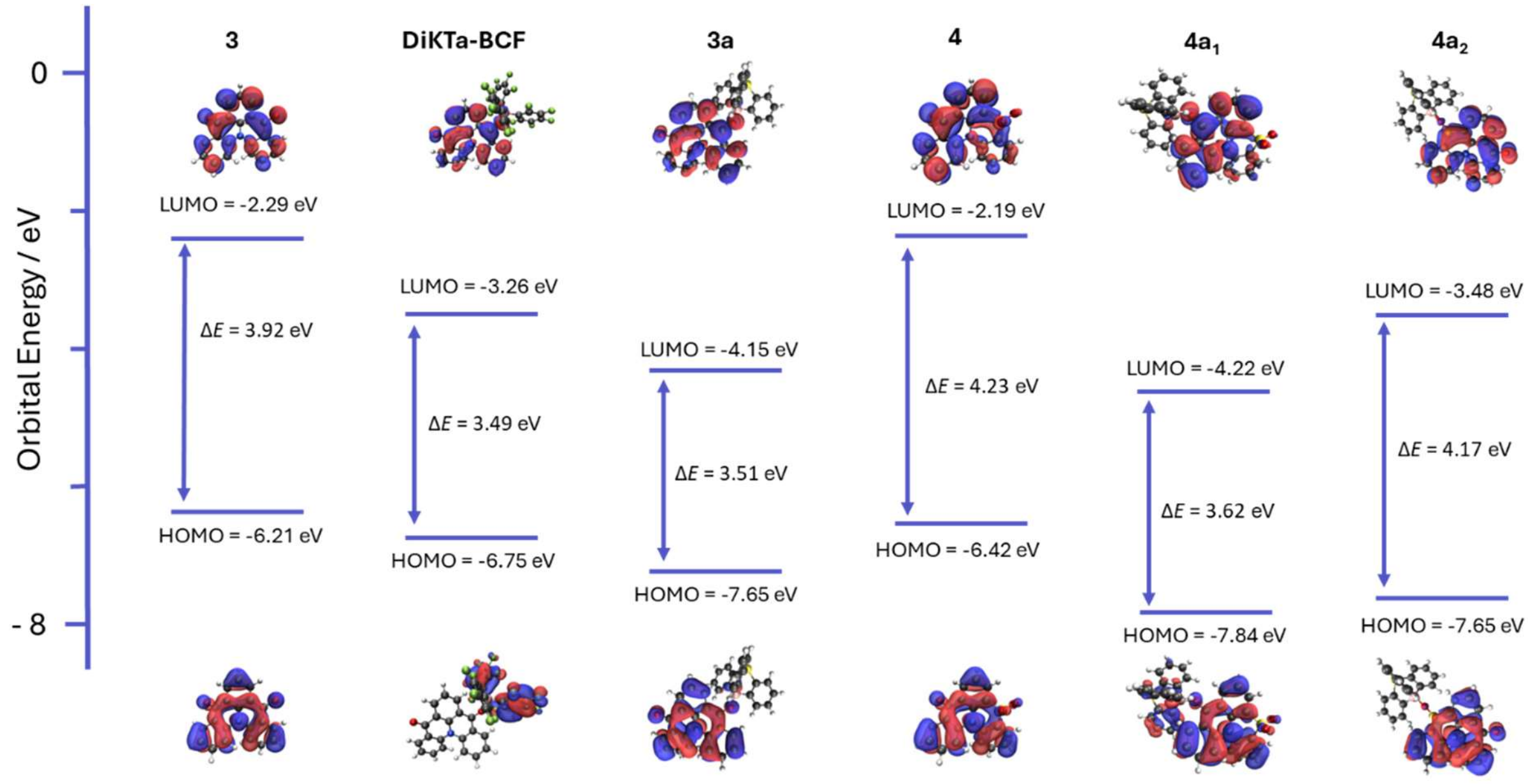

Figure S108. HOMO and LUMO energy levels calculated at the PBE0/6-31G(d,p) (in toluene) level of theory for **3**, **3-BCF**, **3a**, **4**, **4a₁** (bound at carbonyl oxygen) and **4a₂** (bound at sulphone oxygen). Orbital density plots are of the LUMO (top) and HOMO (bottom) (isovalue = 0.02).

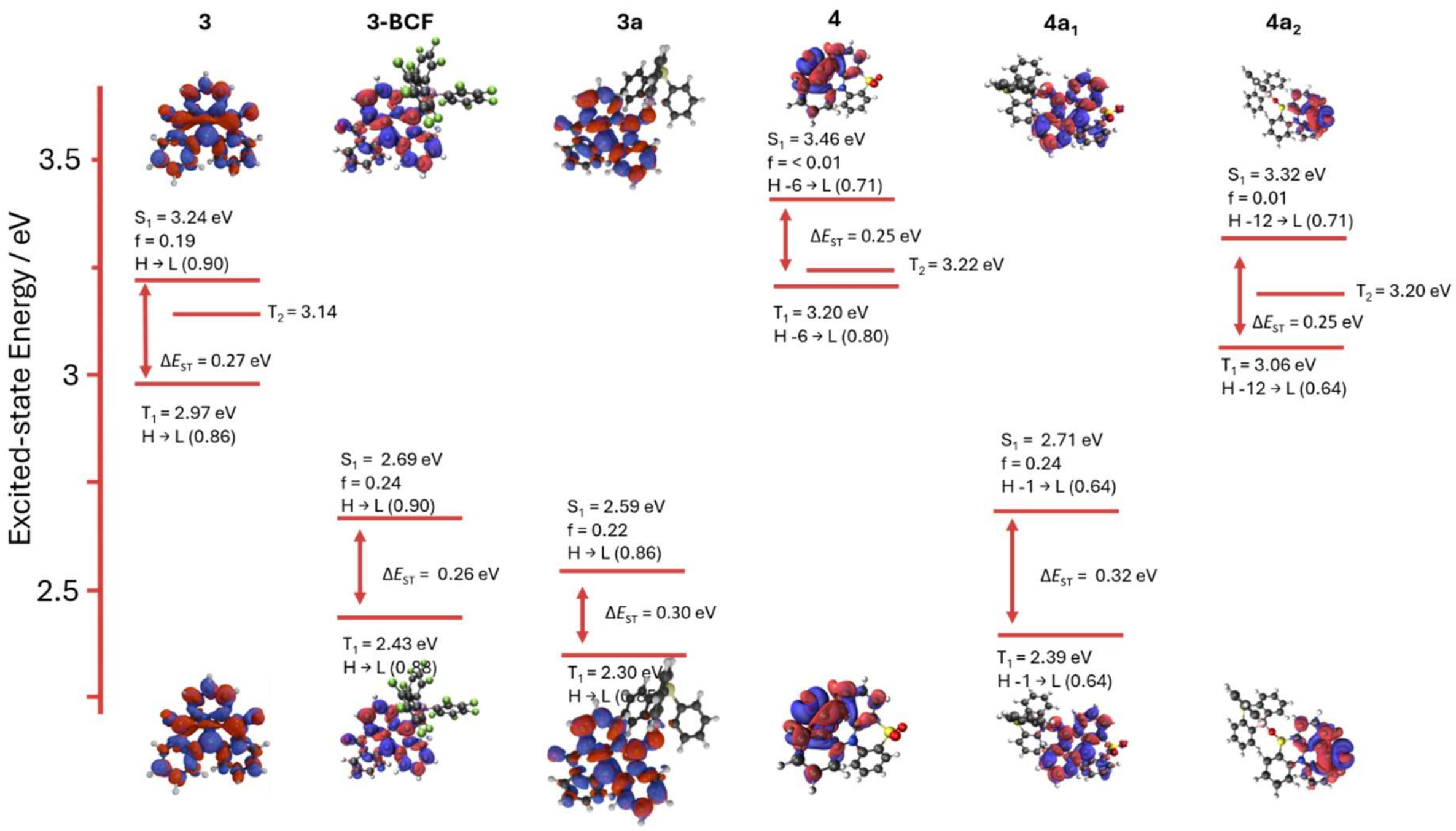

Figure S109. Excited state energy levels calculated at the ADC(2)/cc-pVDZ (gas phase) level of theory for **3**, **3-BCF**, **3a**, **4**, **4a$_1$** (bound at carbonyl oxygen) and **4a$_2$** (bound at sulphone oxygen). Density plots of the NTO hole (blue) and electron (red) of the S_1 (top) and T_1 (bottom) states (isovalue = 0.02)

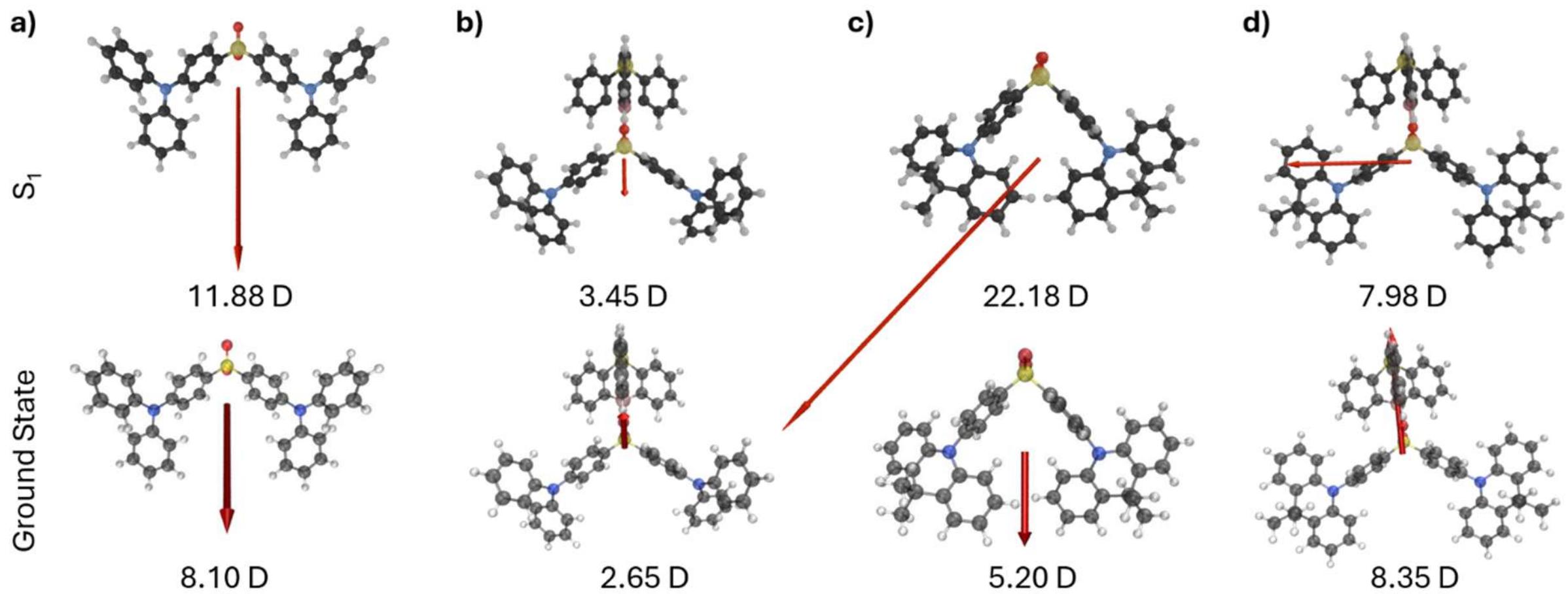

Figure S110. Ground- and excited-state permanent dipole moments calculated at the PBE0/6-31G(d,p) (in toluene) level of theory for a) **1**, b) **1a**, c) **2**, d) **2a**.

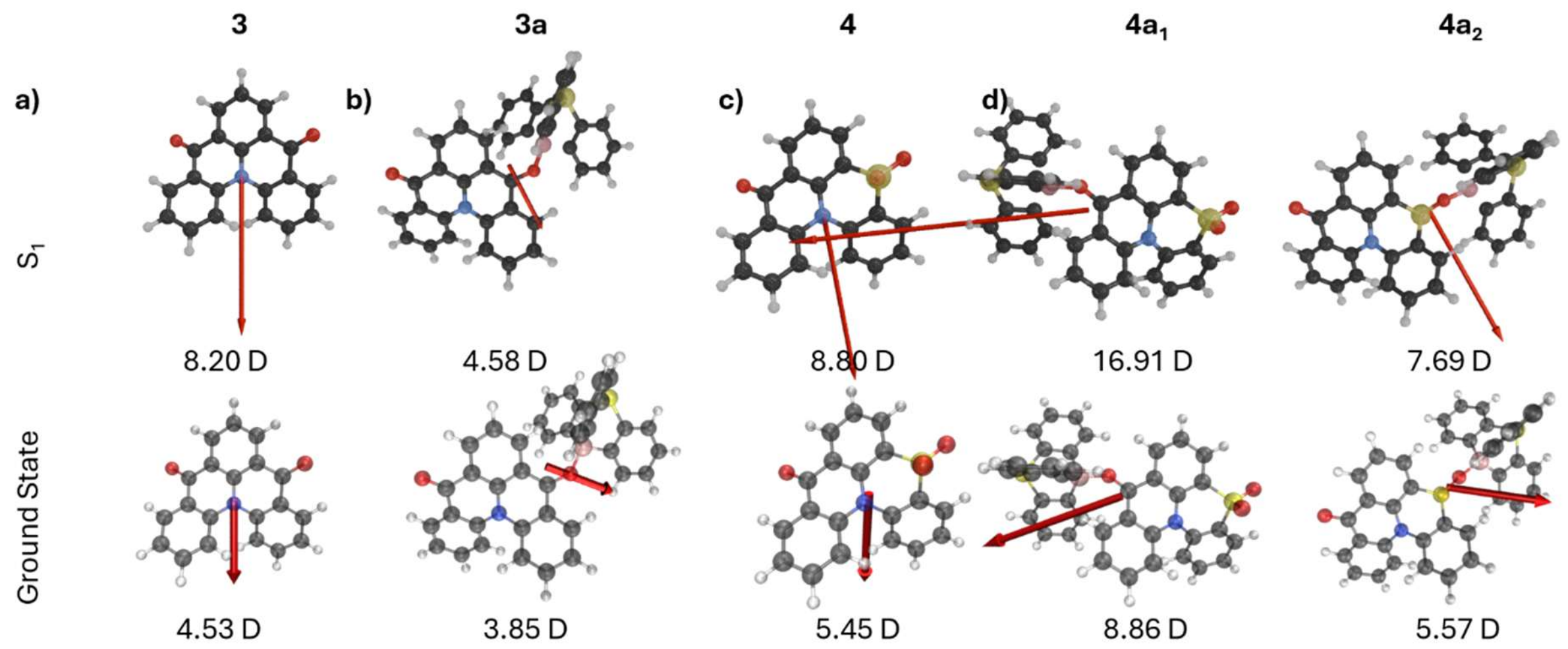

Figure S111. Ground- and excited-state dipole moments calculated at the PBE0/6-31G(d,p) (in toluene) level of theory for a) **3**, b) **3a**, c) **4**, d) **$4a_1$** e) **$4a_2$**.

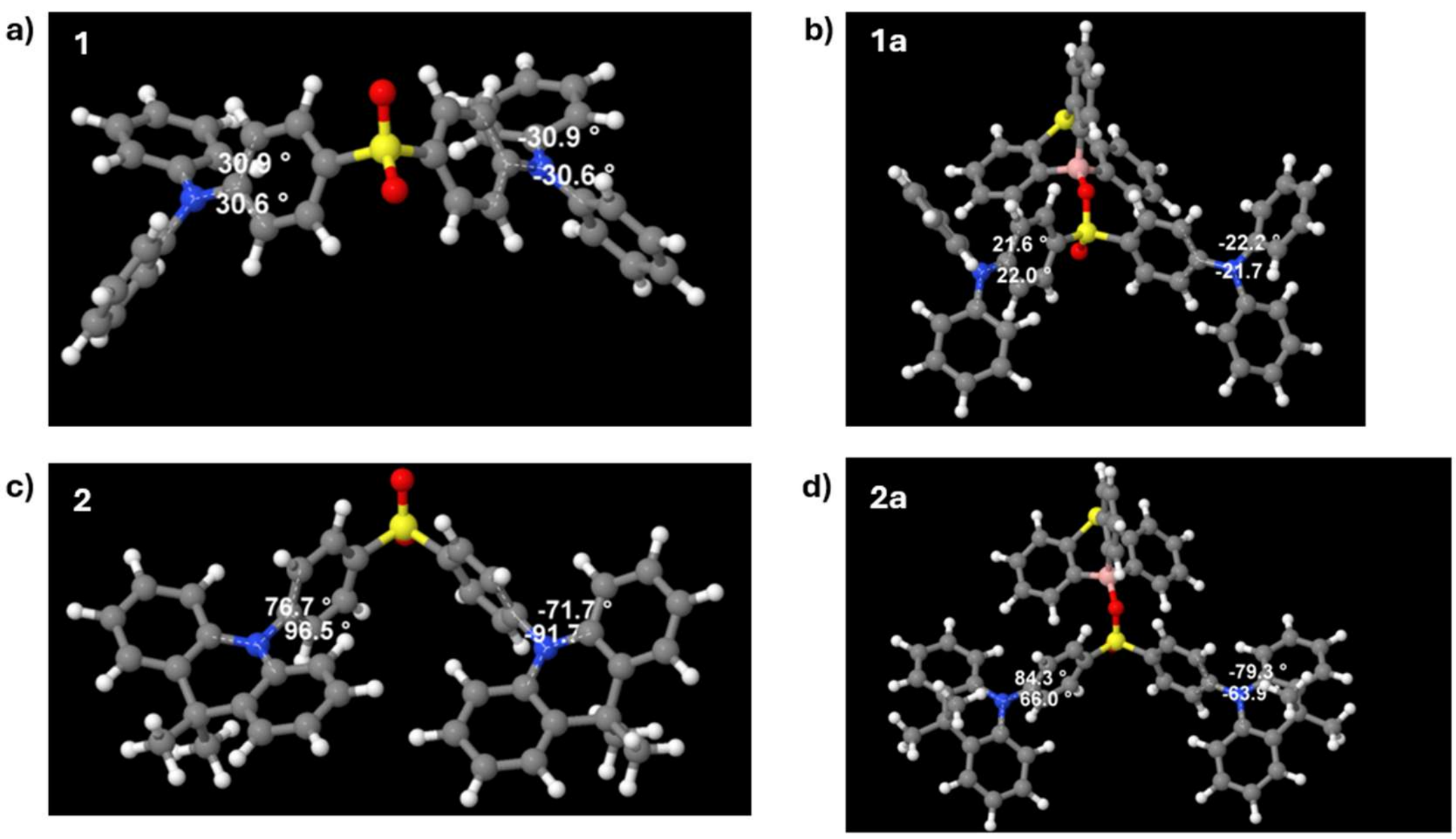

Figure **S112**. Dihedral angle (θ) between donor and acceptor moieties, calculated at the PBEO/6-31G(d,p) (in toluene), visualised using Jmol for a) **1**, b) **1a**, c) **2**, d) **2a**.[42]

1.9 Conformer Analysis for 2 and 2a and discussion of ΔE_{ST} for 2a

Regarding Figure S84d, we use the lower energy singlet band for determining the ΔE_{ST} for **2a**. Taking the higher energy singlet as the S_1 state would suggest that **2a** had a higher lying S_1 state than compound **2** (**2**: 3.06 eV, **2a higher band**: 3.33 eV), which is contrary to both the DFT calculations, other optoelectronic measurements and the trend observed in compounds **1**/**1a**. By taking the E_{onset} for the lower energy singlet state as the S_1 state for **2a** (at 2.98 eV) then the ΔE_{ST} value for the **2a** becomes 0.01 eV. This is in line with the calculations (and other measurements i.e. this S_1 state for **2a** is red-shifted relative to the S_1 state for **2**). The question is then what is the origin of the higher energy singlet state (E_{onset} at 3.33 eV) observed for **2a** in 2-MeTHF at 77 K.

Based on work by Monkman and co-workers[45] on a D-A compound containing DMAC, the presence of other conformers for **2a** was considered. As described by Monkman and co-workers, DMAC can adopt a quasi-equatorial or a quasi-axial conformation. Therefore, as **2a** (and **2**) contain two DMAC units, there are multiple possible conformations (a selection are shown in the figure below, the bent DMAC units can be angled in two different ways as shown below).

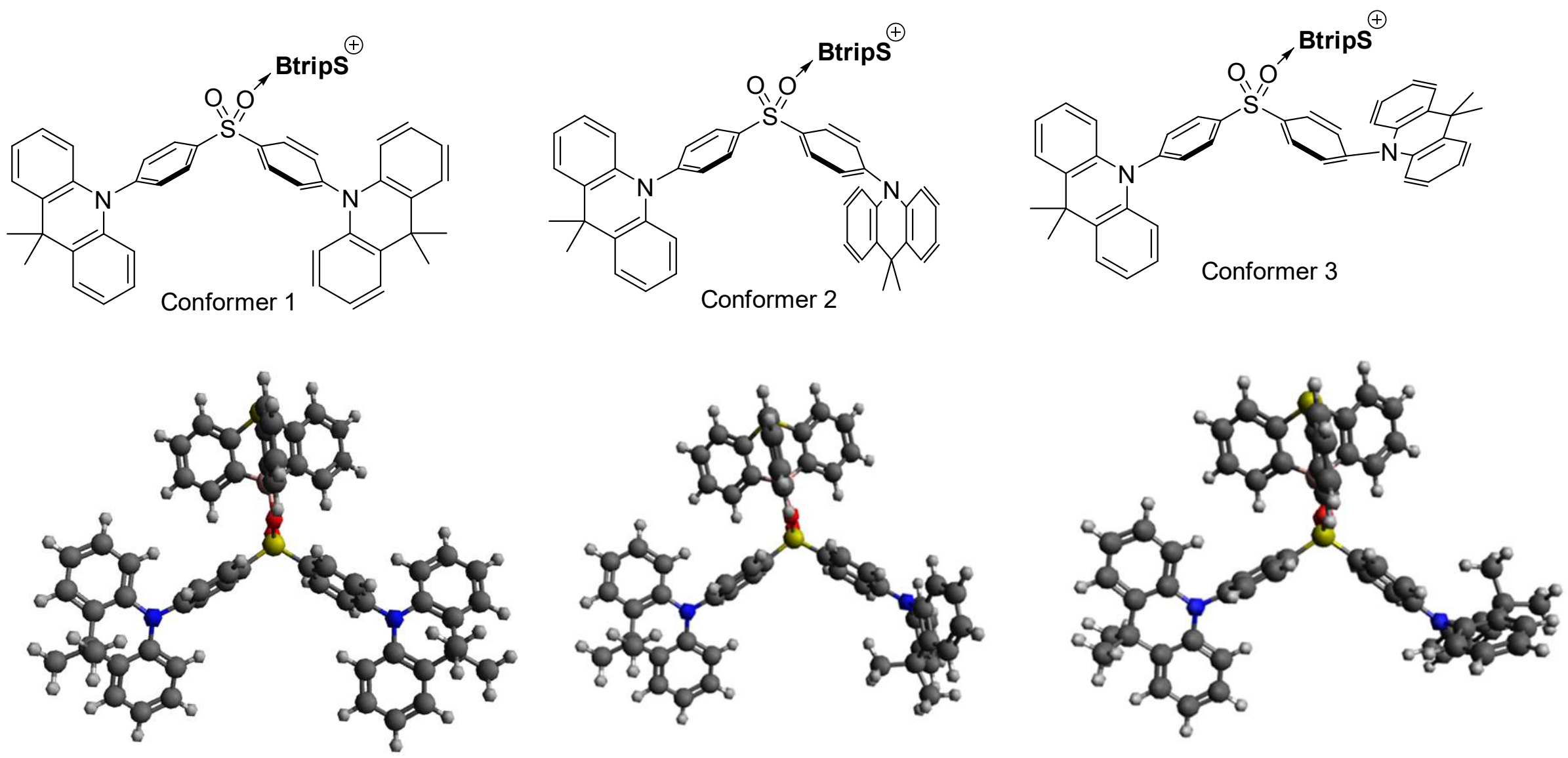

Figure **S113**. Select conformers for **2a**, including the two lowest energy conformers found (conformers **2** and **3**).

Multiple conformers were calculated for **2a**. The two lower energy conformers found had one DMAC quasi-equatorial and one quasi-axial (conformer 2 and 3 shown above). One of these conformers was found to be lower in energy by 6.2 kcal/mol compared to conformer 1, and this conformer was also more stable by 2.8 kcal/mol compared to the other mixed quasi equatorial/quasi axial conformer. Note, a

calculated ΔE of 2.8 kcal/mol corresponds to this conformer being >99% of **2a** at 298 K).

A number of different conformers also were calculated for compound **2**, and the lowest energy conformer was found to be where both DMACs are quasi equatorial. For compound **2** the calculated conformers were all close in energy (< 1.8 kcal/mol) using a toluene solvation model. Therefore, the BTripS unit has a significant impact on the relative energies of the conformers of **2a**. This is attributed to its considerable steric bulk. This can be visualised in the space filling diagram for one of the mixed DMAC conformers (where one DMAC is quasi equatorial and one quasi axial) shown in Figure S114. The quasi-equatorial DMAC (which is approximately planar across the tricyclic DMAC unit) is in closer proximity to the BTripS unit (right DMAC in figure S114). In contrast, when the DMAC unit adopts a quasi-axial form (left DMAC in figure S114), the steric crowding in this region is significantly reduced. This is presumably the origin of the difference in relative conformer energies between **2** and **2a**, with a double quasi-equatorial conformer sterically disfavoured for **2a**.

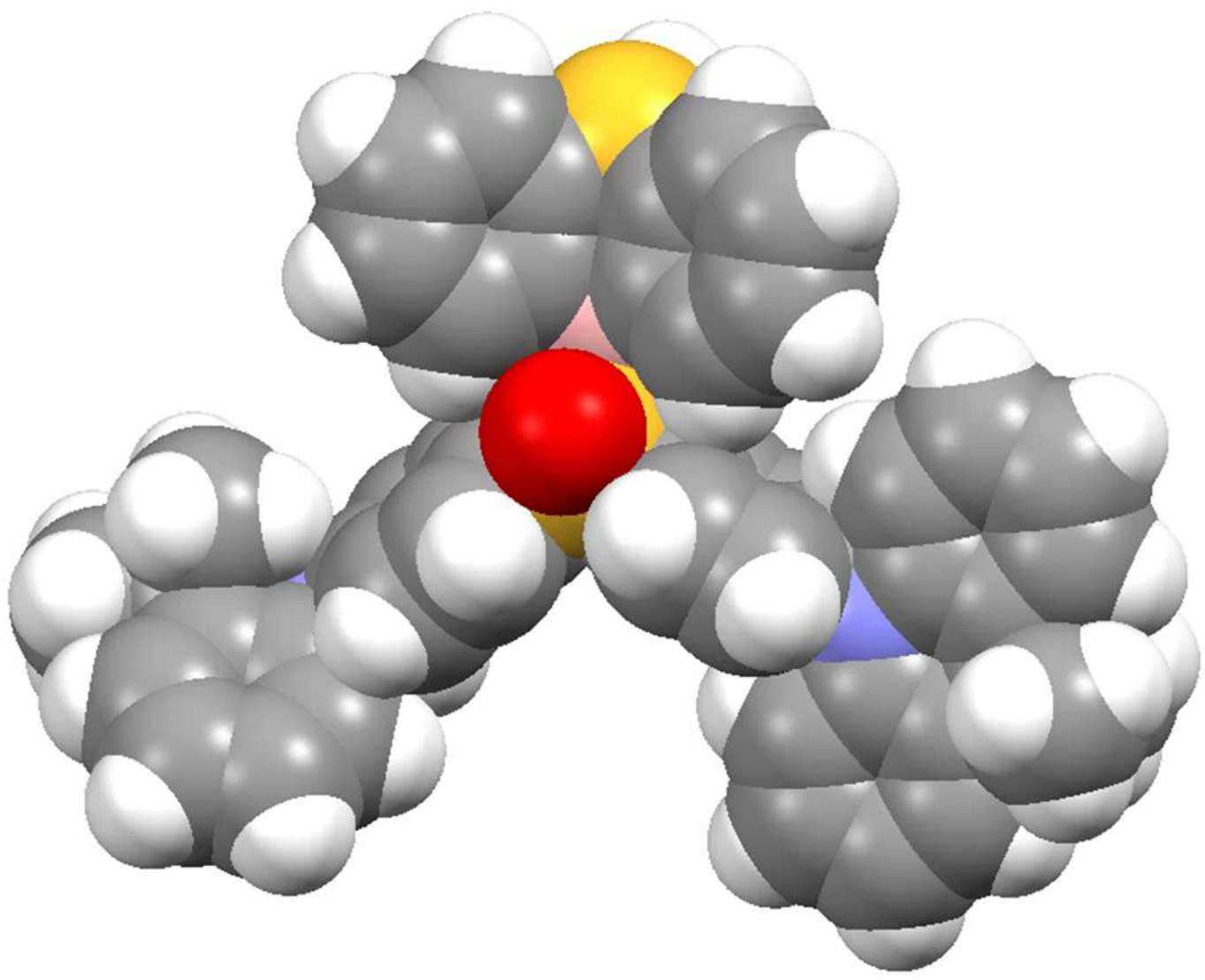

Figure **S114**. Space filling diagram (at 100% van der Waals radii) for a mixed quasi-axial / quasi equatorial conformer of **2a**. (Sulfur = yellow, oxygen = red, nitrogen = blue, carbon = grey, boron = pink).

However, these emitters are salts containing an extremely large anion therefore all calculations are performed on the cations only. Thus, the conformer energies for the salts will be impacted by cation---anion interactions, particularly in the solid-state where the anion and cation will be in close proximity. In solution, the weakly coordinating anion $[B(C_6F_5)_4]^-$ is expected to interact only weakly with the cation, but this still could perturb the relative energies of the conformers to a small degree (and possibly the nature and energy of the excited states).

With the knowledge that different conformers are possible for **2a** in solution the excited state landscape for the two lowest energy calculated conformers (both with mixed DMACs, i.e. one quasi axial and one quasi equatorial DMAC – conformers 2

and 3) was analysed using both toluene and THF solvation models (to approximate the solvents used for the measurements). Note, 2-MeTHF is not parameterised in Gaussian, thus THF was used in its place. The relative energy of the conformers of **2a** was comparable using THF solvation and toluene solvation (with the two mixed quasi axial/quasi equatorial conformers of **2a** being lower in energy in both solvents).

The lowest energy excited singlet and triplet states are shown below for both of these conformers.

Conformer 2 of **2a** in toluene

Singlet (eV)	Triplet (eV)	f
	2.5549	0
2.6186		0.05
	2.976	0
	3.3939	0
3.4742		0.008
3.5461		0.6422

Conformer 2 of **2a** in THF

Singlet (eV)	Triplet (eV)	f
	2.7069	0
2.7596		0.05
	3.0103	0
	3.5381	0
3.5506		0.7114
3.6329		0.013

Conformer 3 of **2a** in toluene

Singlet (eV)	Triplet (eV)	f
	2.5522	0
2.615		0.05
	2.992	0
	3.3905	0
3.4699		0.0008
3.5658		0.678

Conformer 3 of **2a** in THF

Singlet (eV)	Triplet (eV)	f
	2.5632	0
2.5632		0.0003
	3.0076	0
	3.3153	0
3.4307		0.011
3.5526		0.7817

Analysis of these states revealed two possible origins for the higher energy singlet band observed in the 77 K (2-Me-THF) data (Fig. S84d): (i) emission from two different conformers of **2a**, with the data using a THF solvation model revealing the S_1 energies for these two conformers are at 2.56 and 2.76 eV respectively. (ii) the presence of a single conformer of **2a** that displays dual emission (with conformer 2 of **2a** in THF having an S_1 state at 2.76 eV with a low oscillator strength, but a S_1 state at 3.55 eV with a much higher oscillator strength (f). This meets the criteria for a dual emission material, where emission is observed from the S_1 and S_2 states.

To experimentally probe this, excitation-dependent emission spectra were recorded (Figure S115). When the excitation wavelength was changed from 360 nm to 300 nm, no shift in the emission maximum of either band was observed; however, the higher energy band increased in intensity compared to the lower energy band. This is suggestive of a dual emission process.

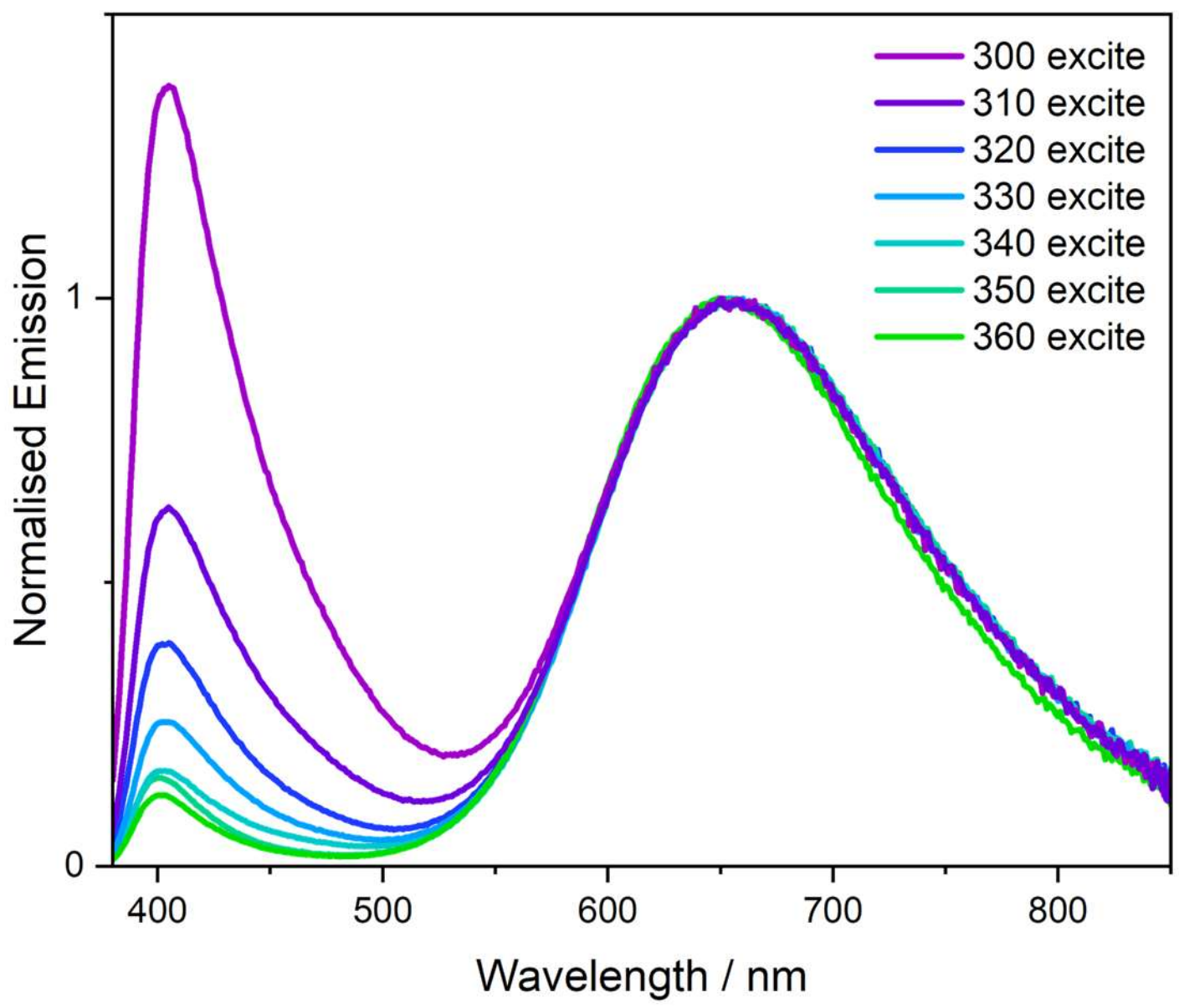

Figure **S115.** Excitation dependent PL spectra of **2a** in toluene.

The alternative hypothesis, that the emission is from two different conformers, is disfavoured due to the ca. 3 kcal/mol energy difference calculated between conformer 2 and conformer 3 for **2a**. Regardless, of whether it is dual emission or two conformers observed at 77 K in 2-Me-THF, combined the data support the conclusion that the ΔE_{ST} for **2a** is 0.01 eV.

Regarding the issue with ΔE_{ST} values for **2a** in thin films being not-extractable. Conformer distribution can be different in thin films vs. that observed in solution.[46] Therefore, we assign the issue with extracting ΔE_{ST} from our multiple (see Fig. S86) thin film measurements on **2a** to the presence of more than one conformer of **2a** in these films.

1.10 References

1. F. Forster, T. T. Metsanen, E. Irran, P. Hrobarik and M. Oestreich, “Cooperative Al-H Bond Activation in DIBAL-H: Catalytic Generation of an Alumenium-Ion-Like Lewis Acid for Hydrodefluorinative Friedel-Crafts Alkylation”, *J. Am. Chem. Soc*, 2017, **139**, 16334–16342.
2. A. Osi, D. Mahaut, N. Tumanov, L. Fusaro, J. Wouters, B. Champagne, A. Chardon and G. Berionni, “Taming the Lewis Superacidity of Non-Planar Boranes: C-H Bond Activation and Non-Classical Binding Modes at Boron”, *Angew. Chem. Int. Ed.*, 2022, **61**, e202112342.
3. Q. Zhang, J. Li, K. Shizu, S. Huang, S. Hirata, H. Miyazaki and C. Adachi, “Design of efficient thermally activated delayed fluorescence materials for pure blue organic light emitting diodes”, *J. Am. Chem. Soc.*, 2012, **134**, 14706–14709.
4. Q. Zhang, B. Li, S. Huang, H. Nomura, H. Tanaka and C. Adachi, “Efficient blue organic light-emitting diodes employing thermally activated delayed fluorescence”, *Nat. Photonics*, 2014, **8**, 326–332.
5. J. E. Field and D. Venkataraman, “Heterotriangulenes – Structure and Properties”, *Chem. Mater.*, 2002, **14**, 962–964.
6. S. Y. Yang, Q. S. Tian, X. J. Liao *et al*, “Efficient circularly polarized thermally activated delayed fluorescence hetero-[4]helicene with carbonyl-/sulfone-bridged triarylamine structures”, *J. Mater. Chem. C*, 2022, **10**, 4393–4401.
7. J. M. Blackwell, K. L. Foster, V. H. Beck and W. E. Piers, “$B(C_6F_5)_3$-Catalyzed Silation of Alcohols: A Mild, General Method for Synthesis of Silyl Ethers”, *J. Org. Chem.*, 1999, **64**, 4887–4892.
8. N. G. Connelly and W. E. Geiger, “Chemical Redox Agents for Organometallic Chemistry”, *Chem. Rev.*, 1996, **96**, 877–910.
9. G. A. Crosby and J. N. Demas, “Measurement of photoluminescence quantum yields. Review”, *J. Phys. Chem.*, 1971, **75**, 991–1024.
10. C. Si, E. F. P. Beaumont, J. Z. Y. Tan, M. J. Demchuk and Z. M. Hudson, “Hydrogen Atom Transfer Induces Photodegradation in Carbonyl-Based Multi-Resonance TADF Emitters”, *Angew. Chem. Int. Ed.*, 2026, **65**, e2490607.
11. Y. Soltani, S. J. Adams, J. Borger, L. C. Wilkins, P. D. Newman, S. J. A. Pope and R. L. Melen, “Synthesis and photophysical properties of imine borane adducts towards vapochromic materials”, *Dalton Trans.*, 2018, **47**, 12656–12660.
12. T. Beringhelli, D. Maggioni and G. D'Alfonso, “^{1}H and ^{19}F NMR Investigation of the Reaction of $B(C_6F_5)_3$ with Water in Toluene Solution”, *Organometallics*, 2001, **20**, 4927–4938.
13. V. Fasano and M. J. Ingleson, “Expanding Water/Base Tolerant Frustrated Lewis Pair Chemistry to Alkylamines Enables Broad Scope Reductive Aminations”, *Chem. Eur. J*, 2017, **23**, 2217–2224.
14. C. Bo, R. Fandos, M. Feliz, *et al*, “fac versus mer Coordination for a Tridentate Diethylene Glyclolate Ligand in Tantalum Complexes: A Combined Experimental and Theoretical Study”, *Organometallics*, 2006, **25**, 3336–3344.
15. W. Robien, SpectraBase, https://spectrabase.com/spectrum/2sb3b4asW9H, (accessed 20/11/2025).
16. J. Cosier and A. M. Glazer, “A nitrogen-gas-stream cryostat for general X-ray diffraction studies”, *J. Appl. Cryst.*, 1986, **19**, 105–107.

17. G. M. Sheldrick, “Crystal structure refinement with SHELXL”, *Acta. Crystallogr. C Struct. Chem.*, 2015, **71**, 3–8.
18. G. M. Sheldrick, “A short history of SHELX”, *Acta. Crystallogr. A*, 2008, **64**, 112–122.
19. O. V. Dolomanov, L. J. Bourhis, R. J. Gildea, J. A. K. Howard and H. Puschmann, “OLEX2: a complete structure solution, refinement and analysis program”, *J. Appl. Cryst.*, 2009, **42**, 339–341.
20. D. Kratzert and I. Krossing, “Recent improvements inDSR”, *J. Appl. Cryst*, 2018, **51**, 928–934.
21. C. M. Cardona, W. Li, A. E. Kaifer, D. Stockdale and G. C. Bazan, “Electrochemical considerations for determining absolute frontier orbital energy levels of conjugated polymers for solar cell applications”, *Adv. Mater.*, 2011, **23**, 2367–2371.
22 Y. Tsuchiya, S. Diesing, F. Bencheikh, Y. Wada, P. L. dos Santos, H. Kaji, E. Zysman-Colman, I. D. W. Samuel, C. Adachi, “Exact solution of kinetic analysis for thermally activated delayed fluorescence materials” *J. Phys. Chem. A* , **2021** 125, 8074–8089
23. M. J. Frisch, G. W. Trucks, H. B. Schlegel, *et al*, Gaussian 16, (Rev. C.01), Wallingford, CT, 2016.
24. M. Ernzerhof and G. E. Scuseria, “Assessment of the Perdew–Burke–Ernzerhof exchange-correlation functional”, *J. Chem. Phys.*, 1999, **110**, 5029–5036.
25. M. Ernzerhof, J. P. Perdew and K. Burke, “Coupling-constant dependence of atomization energies”, *Int. J. Quantum Chem.*, 1997, **64**, 285–295.
26. P. C. Hariharan and J. A. Pople, “The influence of polarization functions on molecular orbital hydrogenation energies”, *Theor. Chim. Acta*, 1973, **28**, 213–222.
27. W. J. Hehre, R. Ditchfield and J. A. Pople, “Self—Consistent Molecular Orbital Methods. XII. Further Extensions of Gaussian—Type Basis Sets for Use in Molecular Orbital Studies of Organic Molecules”, *J. Chem. Phys.*, 1972, **56**, 2257–2261.
28. R. Ditchfield, W. J. Hehre and J. A. Pople, “Self-Consistent Molecular-Orbital Methods. IX. An Extended Gaussian-Type Basis for Molecular-Orbital Studies of Organic Molecules”, *J. Chem. Phys.*, 1971, **54**, 724–728.
29. E. D. Glendening, A. E. Reed, J. E. Carpenter and F. Weinhold, NBO, (Version 3.1), Pittsburgh, TN, 2003.
30. M. D. Hanwell, D. E. Curtis, D. C. Lonie, T. Vandermeersch, E. Zurek and G. R. Hutchison, “Avogadro: an advanced semantic chemical editor, visualization, and analysis platform”, *J. Cheminform.*, 2012, **4**, 17.
31. O. S. Lee and E. Zysman-Colman, Digichem, (version 7), St. Andrews, Scotland, 2025.
32. O. S. Lee, M. C. Gather and E. Zysman-Colman, “Digichem: computational chemistry for everyone”, *Digit. Discov.*, 2024, **3**, 1695–1713.
33. N. M. O'Boyle, A. L. Tenderholt and K. M. Langner, “cclib: a library for package-independent computational chemistry algorithms”, *J. Comput. Chem.*, 2008, **29**, 839–845.
34. W. Humphrey, A. Dalke and K. Schulten, “VMD: visual molecular dynamics”, *J. Mol. Graph.*, 1996, **14**, 33–38, 27–38.
35. J. E. Stone, Masters Thesis, University of Missouri-Rolla, 1998.

36. J. D. Hunter, “Matplotlib: A 2D Graphics Environment”, *Comput. Sci. Eng.*, 2007, **9**, 90–95.
37. N. M. O'Boyle, M. Banck, C. A. James, C. Morley, T. Vandermeersch and G. R. Hutchison, “Open Babel: An open chemical toolbox”, *J. Cheminform.*, 2011, **3**, 33.
38. N. M. O'Boyle, C. Morley and G. R. Hutchison, “Pybel: a Python wrapper for the OpenBabel cheminformatics toolkit”, *Chem. Cent. J.*, 2008, **2**, 5.
39. X. Gao, S. Bai, D. Fazzi, T. Niehaus, M. Barbatti and W. Thiel, “Evaluation of Spin-Orbit Couplings with Linear-Response Time-Dependent Density Functional Methods”, *J. Chem. Theory Comput.*, 2017, **13**, 515–524.
40. C. Adamo and V. Barone, “Toward reliable density functional methods without adjustable parameters: The PBE0 model”, *J. Chem. Phys.*, 1999, **110**, 6158–6170.
41. G. A. Petersson and M. A. Al-Laham, “A complete basis set model chemistry. II. Open-shell systems and the total energies of the first-row atoms”, *J. Chem. Phys.*, 1991, **94**, 6081–6090.
42. TURBOMOLE (version 7.5), Karlsruhe, Germany, 2020.
43. D. Gezelter, Jmol: an open-source Java viewer for chemical structures in 3D, (Version 16.4.1), 2026.

44 X. Gao, S. Bai, D. Fazzi, T. Niehaus, M. Barbatti and W. Thiel, Evaluation of Spin-Orbit Couplings with Linear-Response Time-Dependent Density Functional Methods. *J. Chem. Theory Comput.* **2017,** 13, 515–524.

45 K. Stavrou, L. G. Franca, T. Böhmer, L. M. Duben, C. M. Marian and A. P. Monkman, "Unexpected Quasi-Axial Conformer in Thermally Activated Delayed Fluorescence DMAC-TRZ, Pushing Green OLEDs to Blue“, Adv. Funct. Mater. 2023, 33, 2300910

46 E. Crovini, R. Dhali, D. Sun, T. Matulaitis, T. Comerford, A. M. Z. Slawin, C. Sissa, F. Azzolin, F. Di Maiolo, A. Painelli and E. Zysman-Colman, Molecular geometry and the photophysics of thermally activated delayed fluorescence: the strange case of DMAC-py-TRZ. *J. Mater. Chem. C*, **2023,** 11, 8284–8292.
